# Synthesis, In Silico and In Vitro Evaluation for Acetylcholinesterase and BACE-1 Inhibitory Activity of Some *N*-Substituted-4-Phenothiazine-Chalcones

**DOI:** 10.3390/molecules25173916

**Published:** 2020-08-27

**Authors:** Thai-Son Tran, Minh-Tri Le, Thi-Cam-Vi Nguyen, The-Huan Tran, Thanh-Dao Tran, Khac-Minh Thai

**Affiliations:** 1 Department of Medicinal Chemistry, Faculty of Pharmacy, University of Medicine and Pharmacy at Ho Chi Minh City, Ho Chi Minh City 700000, Vietnam; ttson@huemed-univ.edu.vn (T.-S.T.); tranthanhdao@uphcm.edu.vn (T.-D.T.); 2 Department of Pharmaceutical Chemistry, Faculty of Pharmacy, College of Medicine and Pharmacy, Hue University, Hue City 530000, Vietnam; tthuan@hueuni.edu.vn; 3 School of Medicine, Vietnam National University Ho Chi Minh City, Ho Chi Minh City 700000, Vietnam; 4 Faculty of Applied Sciences, Ton Duc Thang University, Nguyen Huu Tho St., Tan Phong Ward, Dist. 7, Ho Chi Minh City 70000, Vietnam; nguyenthicamvi@tdt.edu.vn

**Keywords:** in silico, QSAR, docking, chalcone, acetylcholiesterase inhibitor, BACE-1

## Abstract

Acetylcholinesterase (AChE) and beta-secretase (BACE-1) are two attractive targets in the discovery of novel substances that could control multiple aspects of Alzheimer’s disease (AD). Chalcones are the flavonoid derivatives with diverse bioactivities, including AChE and BACE-1 inhibition. In this study, a series of *N*-substituted-4-phenothiazine-chalcones was synthesized and tested for AChE and BACE-1 inhibitory activities. In silico models, including two-dimensional quantitative structure–activity relationship (2D-QSAR) for AChE and BACE-1 inhibitors, and molecular docking investigation, were developed to elucidate the experimental process. The results indicated that 13 chalcone derivatives were synthesized with relatively high yields (39–81%). The bioactivities of these substances were examined with pIC_50_ 3.73–5.96 (AChE) and 5.20–6.81 (BACE-1). Eleven of synthesized chalcones had completely new structures. Two substances AC4 and AC12 exhibited the highest biological activities on both AChE and BACE-1. These substances could be employed for further researches. In addition to this, the present study results suggested that, by using a combination of two types of predictive models, 2D-QSAR and molecular docking, it was possible to estimate the biological activities of the prepared compounds with relatively high accuracy.

## 1. Introduction

Alzheimer disease (AD) is an irreversible disorder resulting in dementia among the elderly [1]. This neurodegenerative ailment usually involves the impairment of the cerebral cortex through a complex process leading to the progressive cognitive decline and memory loss [2,3]. Currently, about 50 million people worldwide have been affected by the disease, thus creating a heavy burden on the health care system of many countries [4,5,6]. Therefore, the discovery of new drugs for AD is urgently needed.

The exact etiology of AD is currently not fully known which might be referred to as a neurological disorder caused by multiple factors, such as (i) the low concentrations of acetylcholine (ACh) in synaptic clefts; (ii) the accumulation of extracellular amyloid plaques and intracellular neurofibrillary tangles with hyperphosphorylated Tau protein; (iii) the homeostasis dysregulation of biometals; (iv) the oxidative stress [2,7,8,9]. The multifactorial nature of AD has transformed the paradigm of AD drug development from a single target to multiple targets, either with the multitarget-directed ligands or the cocktail therapy approach [10]. Acetylcholinesterase (AChE) and beta-secretase (BACE-1) are the two drug targets that attracted much attention among others. They are two critical enzymes in Alzheimer’s pathogenesis, responsible for the defects in the cholinergic signaling pathway [11] and the formation of beta-amyloid plaques [12].

For the last few years, chalcones, belonging to flavonoid derivatives, have attracted great interest due to their diverse bioactivities, encompassing antimalarial [13,14], antimicrobial [15,16,17,18,19,20,21,22,23,24,25,26,27], antioxidant [28], antitumor [29,30,31], anti-inflammatory [32,33,34], analgesic [34,35,36], to antiulcer [37,38]. Recent studies have also indicated the abilities of chalcones in inhibiting enzymes, including alpha-glucosidase [39,40], lipoxygenase [33,41], mammalian alpha-amylase [42,43], xanthine oxidase [44], monoamine oxidase (MAO) [45,46,47], especially acetylcholinesterase [48,49,50,51,52] and beta-secretase (BACE-1) [53,54]. Therefore, the research of bioactivities of chalcone derivatives on the function of human brain, especially AChE and BACE-1 provides great promise in the discovery of new therapeutic agents for a range of neurological diseases including AD.

In the current work, a series of *N*-substituted-4-phenothiazine-chalcones was synthesized and examined for AChE and BACE-1 inhibiting effects. These compounds served as an external validation set to evaluate two-dimensional quantitative structure–activity relationship (2D-QSAR) models developed for the AChE and BACE-1 inhibitors. Molecular docking investigation was carried out to provide an insight into the molecular binding abilities of these compounds with the enzymes, through which more detailed information on structure–activity relationship (SAR) were then revealed.

## 2. Results and Discussion

### 2.1. Chemistry

**Figure 1:**
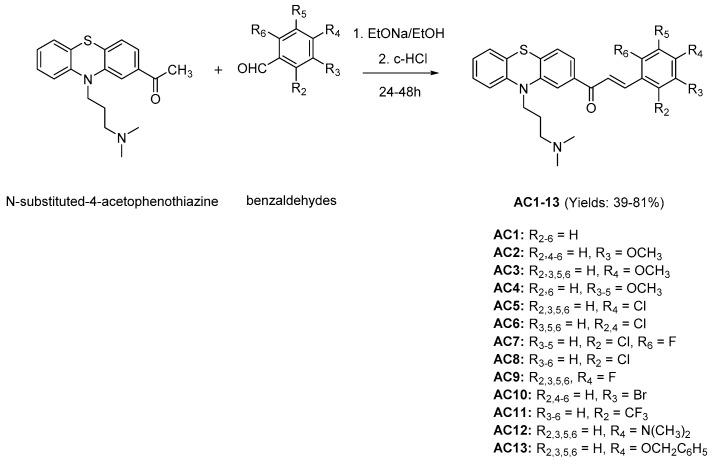
Claisen–Schmidt condensation reaction in chalcones synthesis. EtONa/EtOH: Sodium ethanolate in ethanol, c-HCl: concentrated hydrochloric acid.

Claisen–Schmidt condensation reaction [55] was applied to synthesize 13 chalcones (Figure 1) with *N*-substituted-4-acetophenothiazine and benzaldehyde derivatives as reactive agents, ethanol as solvent and sodium ethanolate as catalyst. The synthetic yields were relatively high, ranging from 39% to 81%. The structures of these substances were elucidated via IR (infrared), HR-MS (high-resolution mass spectrometry), ^1^H-NMR (proton nuclear magnetic resonance), and ^13^C-NMR (carbon-13 nuclear magnetic resonance) spectra as described in the experimental section, and 11 of them were found as completely new compounds (AC2, AC4–13).

### 2.2. In Vitro Assays

The results of in vitro assays on AChE and BACE-1 inhibition are indicated in Table 1. From the biological investigation, the inhibitory activities of synthesized substances against the two enzymes were obtained with pIC_50_ values of 3.73–5.96 (IC_50_ 186.21–1.10 µM) for AChE and 5.20–6.81 (IC_50_ 6.34–0.16 µM) for BACE-1. Two compounds AC4 and AC12 exhibited the highest bioactivities in the synthesized substances with pIC_50_ values of 5.44 ± 0.08 (IC_50_ 3.63 ± 0.61 µM, AChE) and 6.81 ± 0.09 (IC_50_ 0.16 ± 0.03 µM, BACE-1), and 5.96 ± 0.10 (IC_50_ 1.10 ± 0.24 µM, AChE) and 6.46 ± 0.05 (IC_50_ 0.35 ± 0.04 µM, BACE-1), respectively. In the present work, galatamine, and quercetin were used as reference substances. Galantamine is a well-known AChE inhibitor used for the treatment of cognitive decline in mild to moderate AD. Quercetin is a flavonoid reported to have an inhibitory activity against BACE-1. These two compounds have been used as references in many studies on AChE and BACE-1 inhibition. The results show that the biological activities of the positive controls in this study are comparative to the previously published values [56,57,58]. On the other hand, the experimental assays also specified that AC12 had a higher activity than galantamine on AChE and all the synthesized chalcones exhibited the higher inhibiting effects than quercetin on BACE-1.

### 2.3. Molecular Docking

**Figure 2:**
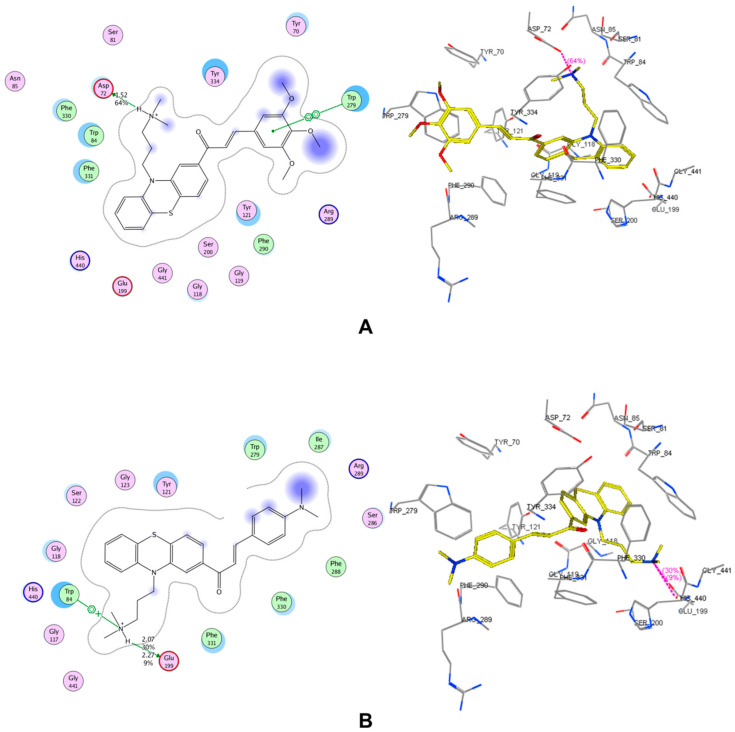
Interactions in the binding pocket of acetylcholinesterase (AChE, complex 1DX6) made by (**A**) AC4 (observed pIC_50_: 5.44 ± 0.08), (**B**) AC12 (observed pIC_50_: 5.96 ± 0.10).

**Figure 3:**
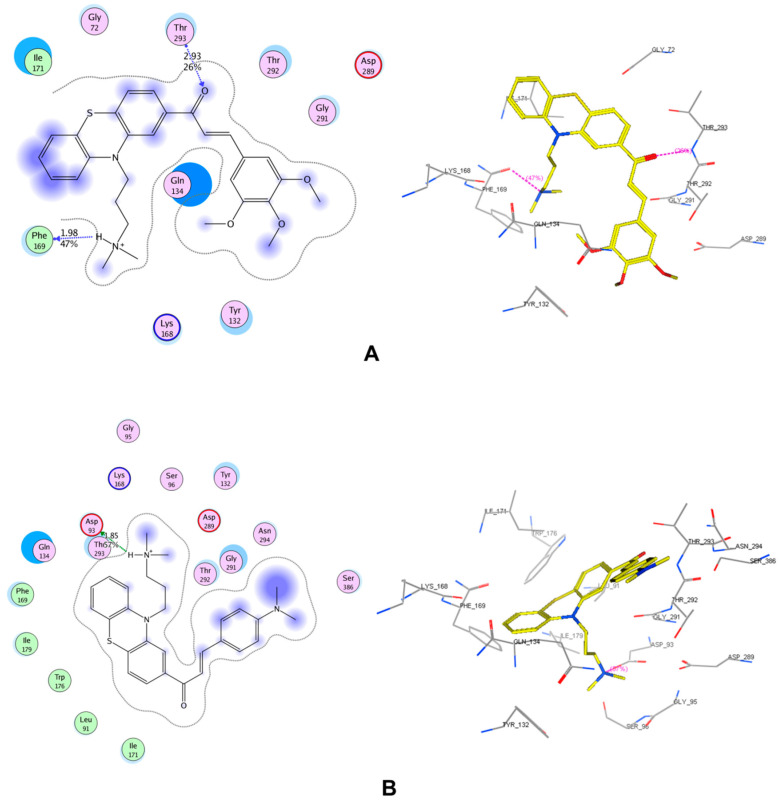
Interactions in the binding pocket of beta-secretase (BACE-1, complex 5HU1-chain A) made by (**A**) AC4 (observed pIC_50_: 6.81 ± 0.09), (**B**) AC12 (observed pIC_50_: 6.46 ± 0.05).

The results of molecular docking study are expressed in Table 2 and in Supplementary Tables S1–S3 and Figures S1–S9. The interactions of 2 compounds with the highest pIC_50_ (AC4 and AC12) on both enzyme AChE and BACE-1 are indicated in Figure 2 and Figure 3. In this study, the co-crystallized complexes employed for AChE was 1DX6 (resolution: 2.30 Å) and for BACE-1 was 5HU1 (resolution: 1.50 Å). These were the complexes with high resolutions and the co-crystallized ligands were the drugs used in clinical (1DX6: galantamine) or in clinical development (5HU1: verubecestat). With this selection, the probability of docked compounds to be reached further optimization would be high. The results indicated that all studied substances were successfully docked into the binding pockets of AChE and BACE-1 with the docking scores of (−17.71)–(−27.80) kJ·mol^−1^ (AChE) and (−11.50)–(−22.51) kJ·mol^−1^ (BACE-1). A linear correlation between docking score and pIC_50_ on AChE of synthesized chalcone derivatives were revealed (Supplementary Figure S10).

The docking scores of most derivatives into AChE were not significantly different (except for AC5 and AC7). In addition, the differences in the observed pIC_50_ values could be partly explained by the analysis of the interactions between chalcone derivatives with AChE. These interactions include (i) arene–arene interactions with Trp84, Trp279, Tyr334; (ii) arene–cation interaction with Trp84; (iii) hydrogen bonding with Asp72, Glu199. These are residues of the active site-gorge of the enzyme where Trp84 and Tyr334 are exposed on the surface, particularly Asp72 on the top, Glu199 on the bottom, and Trp279 is on the entrance of the active-site gorge [59]. Eleven out of thirteen chalcone derivatives (except for AC5 and AC11) could make a hydrogen bond to Asp72. The substituents on the benzene rings of chalcone derivatives showed no strong interaction with the enzyme. However, the presences of different groups on this ring in the chalcone structures could lead to the change in scaffold’s shape as well as its interaction mode and intensity with the enzyme. There were sixsubstances with the same interaction modes (AC3, AC4, AC6, AC9, AC10, and AC11), in which AC4 was observed with the highest activity. This could be attributed to the strongest hydrogen bond between AC4 with Asp72 (score: 64%, length: 1.52 Å) compared to the other compounds.

Compared to galantamine, all the chalcone derivatives exhibited lower bioactivities (except for AC12). This could be explained by the fact that galantamine could create an arene-cation interaction with Phe330, a strong hydrogen bond to Glu199 (score: 36%, length: 1.85 Å), and a hydrogen bond (score: 22%, length: 3.07 Å) to Ser200 (a residue in catalytic triad). AC12 had the strongest observed bioactivity among the studied substances. This can be partly explained that AC12 could yield a strong hydrogen bond to Glu199. None of the rest compounds could interact with this residue. In addition, the arene–cation interaction between AC12 and Trp84 could also lead to the increased AChE inhibitory activity of this substance in relative to the others. The interaction with Trp84 (arene–arene interaction) also made the pIC_50_ value of AC1 equivalent to AC2 although AC1 only makes very weak hydrogen bond to Asp72 (score: 9%, length: 2.14 Å), whereas this interaction created by AC2 was very strong (score: 67%, length: 1.5 Å). Differences in the biological activity of the remaining substances can be explained by the strength of the interactions formed between those derivatives and the enzyme.

Molecular docking results on BACE-1 expressed that most compounds interacted with Asp93, Thr293 in the two chains of enzyme. AC1 and AC10 afforded the same interaction mode in both chains A and B of BACE-1 enzyme similar to verubecestate in this study. The others showed different interactions in the two chains. Two substances AC4 and AC12 (the most active compounds) both interacted with three residues: Asp93, Phe169 and Thr293. AC1 had a weak interaction with Asp93 in both chains of BACE-1 (chain A: hydrogen bond, score: 16%, length 2.22 Å; chain B: hydrogen bond, score: 30%, length 1.99 Å). Most of the remaining compounds formed strong hydrogen bonds with Asp93. Thus, there could be a correlation between the ability to create strong interactions with Asp93 with high BACE-1 inhibitory activity. AC13 exhibited many interactions with amino acid residues in both chains of the enzyme, except for the hydrogen bond with Asp93. The predicted and observed pIC_50_ value of this substance was as high as the others. In this study, quercetin exhibited the least bioactivity against BACE-1, but was the substance with the second highest docking score. Actually, it is not obvious that the most active compounds will always show the highest interaction energy and vice versa. Quercetin showed higher range of interaction energy due to some insignificant interactions with other elements or amino acids in the active site which have no contribution to the biological activity.

### 2.4. 2D-QSAR Models

**Figure 4:**
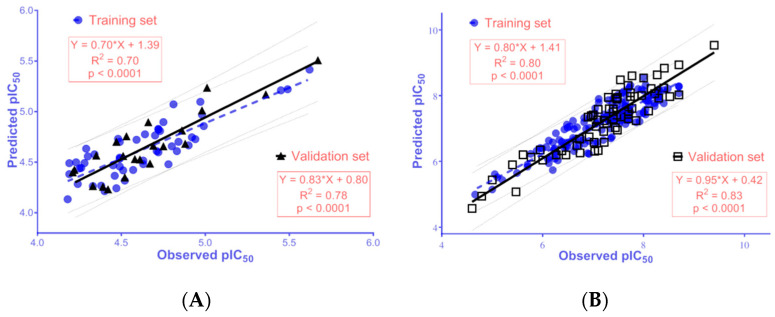
The linear regression between observed pIC_50_ and those predicted from the 2D-QSAR models for inhibitors of (**A**) AChE and (**B**) BACE-1.

The developed 2D-QSAR models for AChE and BACE-1 inhibitors were built upon the data curated from CheMBL database [60], and reported literatures [61,62,63,64,65,66,67,68,69,70,71,72]. The data was processed appropriately according to the guidelines of OECD (Organization for Economic Co-operation and Development) for an acceptable QSAR model [73] (further details are provided in the Materials and Methods section). The validation metrics for these models are showed in Table 3. The linear regressions between the observed bioactivities and those predicted from the QSAR models are expressed in Figure 4. The data sets used to build the models and the equations used for the calculations of the validation metrics are provided in Supplementary Tables S4–S6. Selected molecular descriptors used for building 2D-QSAR models are also described in detail in Supplementary Table S7. The values of selected molecular descriptors using in predicting pIC_50_ of the synthesized chalcone derivatives are provided in Supplementary Tables S8 and S9.

The results exhibited that the QSAR models meet the criteria thresholds for a good model, with R^2^ = 0.70–0.83 (>0.50), Q^2^_LOO_ = 0.57–0.77 (>0.50), RMSE = 0.16–0.41 (<0.50), RMSE_LOO_ = 0.22–0.40 (<0.50), R^2^_PRED_ = 0.78–0.81(>0.50),$\;r_{m}^{2}$ ≥ 0.65; CCC ≥ 0.85; $\bar{r_{m}^{2}}$ ≥ 0.5; and $\Delta r_{m}^{2}$ ≤ 0.2 [75,76].

A comparison of the statistical results obtained from the present QSAR models and the previously published works is indicated in Table 4 and Table 5. Based on the statistical quality in the context of both internal and external validation criteria, theFn-sub models reported in this study is statistically significant and sufficiently robust to predict the biological activities of new ligands.

**Figure 5:**
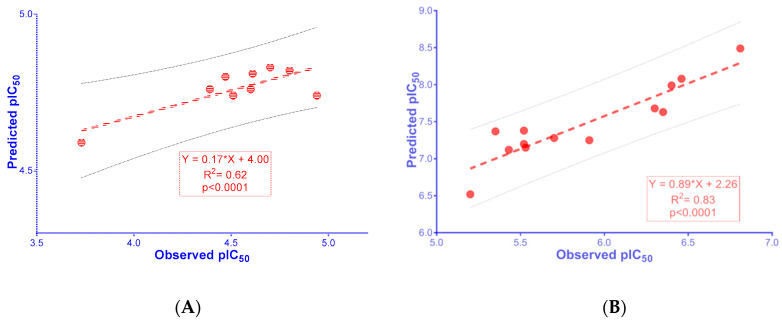
The linear regression between observed and predicted activities of synthesized chalcone derivatives against (**A**) AChE (values from AC1–3, AC6–11) and (**B**) BACE-1 (values from AC1–13).

2D-QSAR model for AChE inhibitors was developed with optimal molecular descriptors, including BCUT_SLOGP_3 (the adjacency and distance matrix), reactive (physical property), PEOE_VSA+1 and PEOE_VSA–3 (partial charge), and SlogP_VSA2 and SMR_VSA2 (subdivided surface areas). The 2D-QSAR model showed a positive correlation with BCUT_SLOGP_3, and a negative correlation with reactive, PEOE_VSA+1, PEOE_VSA–3, SlogP_VSA2, and SMR_VSA2, indicating that new ligands with high BCUT_SLOGP_3, and low reactive, PEOE_VSA+1, PEOE_VSA–3, SlogP_VSA2, SMR_VSA2 values should have higher acetylcholinesterase inhibitory activities. However, as indicated in Table 3, in the developed 2D-QSAR for AChE inhibitors, BCUT_SLOGP_3 had the highest relative importance and it played a decisive role in the predicted pIC_50_ value. As shown in Supplementary Table S8, the studied compounds exhibited a common scaffold structure as *N*-substituted-4-phenothiazine; therefore, the BCUT_SLOGP_3 values were very close to each other, resulting in the similarity among the predicted pIC_50_ of the chalcone derivatives. When the structure changed from chalcone to galantamine, the BCUT_SLOGP_3 value displayed a small increase whilst the predicted biological activity changed significantly, suggesting that the developed QSAR model could provide some prediction on the difference in bioactivity of substances with different scaffolds. It can be observed from Table 1 and Figure 5, the 2D-QSAR for AChE in the present work could predict quite accurately the biological activity most of synthetic chalcone derivatives, with nine out of 13 compounds displaying correlations in the predicted and observed pIC_50_ values (R^2^ = 0.62).

Additionally, it can be seen that the 2D-QSAR model for BACE-1 inhibitors was developed with 11 molecular descriptors, namely petitjean and BCUT_PEOE_1 (adjacency and distance matrixes), a_ICM, chiral_u, rings, and a_Nn (atom counts and bond counts), PEOE_VSA–0 and PEOE_VSA–6 (partial charges), logS (physical properties), and SlogP_VSA3 and SlogP_VSA5 (subdivided surface areas). The 2D-QSAR model reported a positive correlation between pIC_50_ for BACE-1 inhibition with the descriptors of petitjean, BCUT_PEOE_1, a_ICM, rings, a_Nn, PEOE_VSA–0, PEOE_VSA–6, SlogP_VSA3, SlogP_VSA5, and a negative correlation with chiral_u, logS. This thus suggested that new ligands with high petitjean, BCUT_PEOE_1, a_ICM, rings, a_Nn, PEOE_VSA–0, PEOE_VSA–6, SlogP_VSA3, SlogP_VSA5, and low chiral_u, or logS values should have higher BACE-1 inhibiting effect. The descriptor BCUT_PEOE_1 was positively correlated with biological activity; it had the highest relative importance with a decisive role to the predicted pIC_50_ value (Table 3). The results shown in Table 1 indicate that the two substances, AC4 and AC12, had the highest observed and predicted pIC_50_ values. This could be due to these two substances having higher BCUT_PEOE_1 values than the others (high value of BCUT_PEOE_1 was also observed in AC11, which also exhibited a high bioactivity on BACE-1). The second factor contributing to the increased pIC_50_ values of AC4 and AC12 compared to other substances is the a_ICM descriptor which was the second most important parameter in the studied model and positively correlated with the pIC_50_ value (Table 3). In addition, high values at other descriptors such as PEOE_VSA–0, SlogP_VSA5 (Supplementary Table S9) also contributed to the increase of pIC_50_ of AC4 and AC12, though not much. The correlation between the QSAR predicted and observed pIC_50_ values for BACE-1 was obtained with R^2^ value of 0.83 (Figure 5).

## 3. Materials and Methods

### 3.1. Material and Instruments

All chemicals were obtained from commercial suppliers, and used without further purification. Melting points were determined on open capillary tubes and are uncorrected (using Gallenkamp apparatus). IR spectra were recorded on a Bruker FTIR Tensor 27 instrument. MS spectra were recorded on an Agilent 6200 Series TOF and 6500 Series Q-TOF LC/MS system. ^1^H-NMR and ^13^C-NMR spectra were recorded on an AV500 Bruker (500 MHz) spectrometer. Chemical shifts were reported in parts per million (ppm) downfield relative to tetramethylsilane as an internal standard. Peak splitting patterns were abbreviated as m (multiplet), s (singlet), bs (broad singlet), d (doublet), bd (broad doublet), t (triplet), and dd (doublet of doublets).

All computation processes were performed on a computer system with the processsor of Intel® Core^TM^ i&-7700 CPU @ 3.60 Hz, 16.0 GB of RAM, the Visual Graphic Card of NVIDIA GeForce GT 1030 2GB, and the operating system of 64 bit Windows 10 (Microsoft, Redmond, WA, USA).

### 3.2. Chemistry

The chemical synthesis of the chalcone compounds AC1–AC13 is indicated in the Figure 1 and as follows. *N*-substituted-4-acetophenothiazine and benzaldehyde derivatives in equimolar amounts were dissolved in ethanol and cooled in ice. Sodium ethanolate was then added. The resulting solution was stirred using an utrasonic probe. The chemical reaction was monitored by thin layer chromatography. Final mixture was cooled and acidified by a solution of concentrated HCl to pH ≈ 5–7 and left on for 24–48 h. The crude product appeared in solid or liquid. The solid was filtered, washed with cold water, and recrystallized from appropriate solvents to give the final product. With the liquid, post-reaction mixture was evaporated and purified by column chromatography with appropriate solvent systems to obtain the final product. The structures of the target compounds were elucidated by IR, MS, ^1^H-NMR, and ^13^C-NMR spectra.

(*E*)-1-(10-(3-(dimethylamino)propyl)-10*H*-phenothiazin-2-yl)-3-phenylprop-2-en-1-one (AC1), Yield: 68%. Mp: Liquid at room temperature. IR (ν cm^–1^, KBr): 2939 (ν_C-H sp3_), 1658 (ν_C=O_), 1599 (ν_C=C Ar_), 1491 (ν_C=C Ar_), 1061 (ν_C-N_). MS*:* [M + H]^+^ *m*/*z* = 415.1814. ^1^H-NMR (500 MHz, MeOD, δ ppm): 7.76 (d, *J* = 16 Hz, 1H, CH=C), 7.71 (m, 2H, Ar-H), 7.66 (d, *J* = 16 Hz, 1H, CH=C), 7.61 (dd, *J*_1_ = 1 Hz, *J*_2_ = 8 Hz, 1H, Ar-H), 7.48 (d, 1H, Ar-H), 7.41 (m, 3H, Ar-H), 7.18 (m, 2H, Ar-H), 7.09 (dd, *J*_1_ =1 Hz, *J*_2_ = 7.5 Hz, 1H, Ar-H), 6.95 (m, 2H, Ar-H), 3.94 (t, *J* = 7 Hz, 2H, N-CH_2_), 2.51 (t, *J* = 7.5 Hz, 2H, N-CH_2_), 2.22 (s, 6H, N-CH_3_), 1.91 (m, 2H, CH_2_).^13^C-NMR (125 MHz, MeOD, δ ppm): 191.1, 146.7, 146.1, 145.5, 138.4, 136.1, 133.7, 131.8, 130.1, 129.7, 128.9, 128.3, 128.2, 125.2, 124.3, 124.1, 122.8, 117.2, 115.8, 57.6, 46.1, 45.2, 25.2.

(*E*)-1-(10-(3-(dimethylamino)propyl)-10*H*-phenothiazin-2-yl)-3-(3-methoxyphenyl)prop-2-en-1-one (AC2), Yield: 43%. Mp: Liquid at room temperature. IR (ν cm^–1^, KBr): 2970 (ν_C-H sp3_), 1658 (ν_C=O_), 1601 (ν_C=C Ar_), 1462 (ν_C=C Ar_), 1245, (ν_C-O_), 1088 (ν_C-N_). MS: [M + H]^+^ *m*/*z* = 445.1977. ^1^H-NMR (500 MHz, MeOD, δ ppm): 7.78 (d, *J* = 16 Hz, 1H, CH=C), 7.72 (d, *J* = 16 Hz, 1H, CH=C), 7.72 (dd, *J*_1_ = 1.5 Hz, *J*_2_ = 8.5 Hz, 1H, Ar-H), 7.58 (d, *J* = 1.5 Hz, 1H, Ar-H), 7.35 (m, 3H, Ar-H), 7.29 (d, *J* = 8 Hz, 1H, Ar-H), 7.24 (m, 1H, Ar-H), 7.15 (dd, *J*_1_ = 1.5 Hz, *J*_2_ = 7.5 Hz, 1H, Ar-H), 7.05 (dd, *J*_1_ = 1.5 Hz, *J*_2_ = 7.5 Hz, 1H, Ar-H), 7.03 (m, 1H, Ar-H), 7.00 (m, 1H, Ar-H), 4.06 (t, *J* = 7 Hz, 2H, N-CH_2_), 3.88 (s, 3H, OCH_3_), 2.56 (t, *J* = 7.5 Hz, 2H, N-CH_2_), 2.24 (s, 6H, N-CH_3_), 2.01 (m, 2H, CH_2_). ^13^C-NMR (125 MHz, MeOD, δ ppm): 191.5, 146.9, 146.1, 145.8, 138.2, 137.6, 133.9, 131.0, 129.0, 128.4, 128.2, 125.4, 124.4, 124.1, 123.2, 122.4, 117.7, 117.3, 115.9, 114.6, 57.8, 55.9, 46.2, 45.3, 25.4.

(*E*)-1-(10-(3-(dimethylamino)propyl)-10*H*-phenothiazin-2-yl)-3-(4-methoxyphenyl)prop-2-en-1-one (AC3), Yield: 41%. Mp: Liquid at room temperature. IR (ν cm^–1^, KBr): 2968 (ν_C-H sp3_), 1655 (ν_C=O_), 1593 (ν_C=C Ar_), 1462 (ν_C=C Ar_), 1254 (ν_C-O_), 1036 (ν_C-N_). MS: [M + H]^+^ *m*/*z* = 445.1923. ^1^H-NMR (500 MHz, MeOD, δ ppm): 7.76 (d, *J* = 15.5 Hz, 1H, CH=C), 7.69 (m, 2H, Ar-H), 7.64 (dd, *J*_1_ = 2 Hz, *J*_2_ = 8 Hz, 1H, Ar-H), 7.56 (d, *J* = 15.5 Hz, 1H, CH=C), 7.52 (d, *J* = 1.5 Hz, 1H, Ar-H), 7.23 (m, 2H, Ar-H), 7.13 (dd, *J*_1_ = 1.5 Hz, *J*_2_ = 7.5 Hz, 1H, Ar-H), 7.02 (d, *J* = 7.5 Hz, 1H, Ar-H), 6.97 (m, 3H, Ar-H), 4.03 (t, *J* = 7 Hz, 2H, N-CH_2_), 3.85 (s, 3H, OCH_3_), 2.70 (t, *J* = 7.5 Hz, 2H, N-CH_2_), 2.37 (s, 6H, N-CH_3_), 2.03 (m, 2H, CH_2_). ^13^C-NMR (125 MHz, MeOD, δ ppm): 191.4, 146.7, 146.3, 145.6, 138.9, 133.6, 131.7, 129.0, 128.8, 128.4, 128.2, 125.5, 124.3, 124.2, 120.3, 117.3, 115.8, 115.5, 57.4, 55.9, 45.9, 44.7, 24.7.

(*E)*-1-(10-(3-(dimethylamino)propyl)-10*H*-phenothiazin-2-yl)-3-(3,4,5-trimethoxyphenyl)prop-2-en-1-one (AC4), Yield: 51%. Mp: 167–168 °C. IR (ν cm^–1^, KBr): 2925 (ν_C-H sp3_), 1657 (ν_C=O_), 1582 (ν_C=C Ar_), 1461 (ν_C=C Ar_), 1280 (ν_C-O_), 1124 (ν_C-N_). MS: [M + H]^+^ *m*/*z* = 505.2132. ^1^H-NMR (500 MHz, MeOD, δ ppm): 7.75 (d, *J* = 15.5 Hz, 1H, CH=C), 7.74 (dd, *J*_1_ = 1.5 Hz, *J*_2_ = 7.5 Hz, 1H, Ar-H), 7.67 (d, *J* = 15.5 Hz, 1H, CH=C), 7.58 (d, *J* = 1.5 Hz, 1H, Ar-H), 7.28 (d, *J* = 8 Hz, 1H, Ar-H), 7.24 (td, *J*_1_ = 1.5 Hz, *J*_2_ = 8 Hz, 1H, Ar-H), 7.15 (dd, *J*_1_ = 1.5 Hz, *J*_2_ = 7.5 Hz, 1H, Ar-H), 7.09 (s, 2H, Ar-H), 7.05 (d, *J* = 7.5 Hz, 1H, Ar-H), 6.98 (td, *J* = 1Hz, *J* = 6.5 Hz, 1H, Ar-H), 4.06 (t, *J* =6.5 Hz, 2H, N-CH_2_), 3.92 (s, 6H, OCH_3_), 3.83 (s, 3H, OCH_3_), 2.28 (s, 6H, N-CH_3_), 2.02 (m, 2H, CH_2_). ^13^C-NMR (125 MHz, MeOD, δ ppm): 191.5, 154.9, 146.9, 146.4, 145.8, 141.7, 138.7, 133.9, 132.1, 129.0, 128.4, 128.2, 125.4, 124.5, 124.2, 122.3, 117.3, 115.8, 107.4, 61.2, 57.7, 56.8, 46.1, 45.2, 25.2.

(*E*)-3-(4-chlorophenyl)-1-(10-(3-(dimethylamino)propyl)-10*H*-phenothiazin-2-yl)-3-phenylprop-2-en-1-one (AC5), Yield: 78%. Mp: Liquid at room temperature. IR (ν cm^–1^, KBr): 2936 (ν_C-H sp3_), 1657 (ν_C=O_), 1590 (ν_C=C Ar_), 1461 (ν_C=C Ar_), 1089 (ν_C-N_), 744 (ν_C-Cl_). MS: [M + H]^+^ *m*/*z* = 449.1451. ^1^H-NMR (500 MHz, MeOD, δ ppm): 7.67 (d, *J* = 16 Hz, 1H, CH=C), 7.61 (m, 4H, Ar-H & CH=C), 7.44 (s, 1H, Ar-H), 7.35 (d, *J* = 7 Hz, 2H, Ar-H), 7.18 (t, *J* = 7.5 Hz, 1H, Ar-H), 7.12 (t, *J* = 7.5 Hz, 1H, Ar-H), 7.06 (d, *J* = 7.5 Hz, 1H, Ar-H), 6.93 (m, 2H, Ar-H), 3.91 (t, *J* = 5.5 Hz, 2H, N-CH_2_), 2.48 (t, *J* = 7 Hz, 2H, N-CH_2_), 2.20 (s, 6H, N-CH_3_), 1.92 (m, 2H, CH_2_). ^13^C-NMR (125 MHz, MeOD, δ ppm): 190.7, 146.7, 145.5, 144.3, 138.3, 137.4, 134.9, 133.7, 131.1, 130.2, 128.8, 128.3, 128.1, 125.1, 124.3, 124.1, 123.4, 117.2, 115.6, 57.6, 46.1, 45.2, 25.2.

(*E*)-3-(2,4-dichlorophenyl)-1-(10-(3-(dimethylamino)propyl)-10*H*-phenothiazin-2-yl)-3-phenylprop-2-en-1-one (AC6), Yield: 62%. Mp: Liquid at room temperature. IR (ν cm^–1^, KBr): 2940 (ν_C-H sp3_), 1658 (ν_C=O_), 1584 (ν_C=C Ar_), 1463 (ν_C=C Ar_), 1222 (ν_C-N_). MS: [M + H]^+^ *m*/*z* = 483.1054. ^1^H-NMR (500 MHz, DMSO, δ ppm): 8.26 (d, *J* = 8.5 Hz, 1H, CH=C), 7.98 (m, 2H, Ar-H), 7.82 (dd, *J*_1_ = 1.5 Hz, *J*_2_ = 8 Hz, 1H), 7.78 (d, *J* = 2 Hz, 1H, Ar-H), 7.57 (d, *J* = 8 Hz, 1H, Ar-H), 7.56 (d, *J* = 8.5 Hz, 1H, CH=C), 7.35 (d, *J* = 8 Hz, 1H, Ar-H), 7.25 (td, *J*_1_ = 1.5 Hz, *J*_2_ = 8.5 Hz, 1H, Ar-H), 7.18 (dd, *J* = 1.5 Hz, *J* = 6.5 Hz, 1H, Ar-H), 7.08 (d, *J* = 7.5 Hz, 1H, Ar-H), 6.98 (td, *J*_1_ = 1 Hz, *J*_2_ = 6.5 Hz, 1H, Ar-H), 4.0 (t, *J* = 7 Hz, 2H, N-CH_2_), 2.35 (t, *J* = 7 Hz, 2H, N-CH_2_), 2.11 (s, 6H, N-(CH_3_)_2_), 1.83 (m, 2H, CH_2_). ^13^C-NMR (125 MHz, DMSO, δ ppm): 188.1, 137.2, 136.5, 135.6, 135.1, 131.3, 131.0, 129.8, 129.5, 128.0, 127.9, 127.2, 127.1, 125.3, 123.4, 122.8, 122.3, 116.2, 114.3, 56.2, 45.2, 44.7, 24.1.

*(E*)-3-(2-chloro-6-fluorophenyl)-1-(10-(3-(dimethylamino)propyl)-10*H*-phenothiazin-2-yl)-3-phenylprop-2-en-1-one (AC7), Yield: 43%. Mp: 152–155 °C. IR (ν cm^–1^, KBr): 2935 (ν_C-H sp3_), 1660 (ν_C=O_), 1596 (ν_C=C Ar_), 1467 (ν_C=C Ar_), 1062 (ν_C-N_). MS: [M + H]^+^ *m*/*z* = 467.1368. ^1^H-NMR (500 MHz, DMSO, δ ppm): 7.81 (d, *J* = 16 Hz, 1H, CH=C), 7.74 (d, *J* = 16 Hz, 1H, CH=C), 7.63 (dd, *J*_1_ = 1.5 Hz, *J*_2_ = 8 Hz, 1H, Ar-H), 7.51 (m, 3H, Ar-H), 7.40 (m, 1H, Ar-H), 7.33 (d, *J* = 7.5 Hz, 1H, Ar-H), 7.23 (td, *J*_1_ = 1.5 Hz, *J*_2_ = 8.5 Hz, 1H, Ar-H), 7.16 (dd, *J*_1_ = 1.5 Hz, *J*_2_ = 7.5 Hz, 1H, Ar-H), 7.06 (d, *J* = 8 Hz, 1H, Ar-H), 6.97 (td, *J*_1_ = 1 Hz, *J*_2_ = 7.5 Hz, 1H, Ar-H), 3.97 (t, *J* = 7 Hz, 2H, N-CH_2_), 2.34 (t, *J* = 6.5 Hz, 2H, N-CH_2_), 2.10 (s, 6H, N-CH_3_), 1.82 (m, 2H, CH_2_). ^13^C-NMR (125 MHz, DMSO, δ ppm): 188.4, 144.8, 143.8, 136.4, 135.0, 133.1, 132.1, 130.9, 128.8, 128.0, 127.2, 126.2, 123.0, 122.8, 122.1, 121.4, 121.3, 116.1, 115.6, 114.2, 56.2, 45.2, 44.7, 24.0.

(*E*)-3-(2-chlorophenyl)-1-(10-(3-(dimethylamino)propyl)-10*H*-phenothiazin-2-yl)-3-phenylprop-2-en-1-one (AC8), Yield: 81%. Mp: 224–227 °C. IR (ν cm^–1^, KBr): 2957 (ν_C-H sp3_), 1658 (ν_C=O_), 1598 (ν_C=C Ar_), 1468 (ν_C=C Ar_), 1070 (ν_C-N_). MS: [M + H]^+^ *m*/*z* = 449.1475. ^1^H-NMR (500 MHz, MeOD, δ ppm): 8.23 (d, *J* = 15.5 Hz, 1H, CH=C), 8.03 (dd, *J*_1_ = 1.5 Hz, *J*_2_ = 7 Hz, 1H, Ar-H), 7.80 (d, *J* = 1.5 Hz, 1H, Ar-H), 7.78 (d, *J* = 15.5 Hz, 1H, CH=C), 7.65 (s, 1H, Ar-H), 7.52 (dd, *J*_1_ = 1.5 Hz, *J*_2_ = 7.5 Hz, 1H, Ar-H), 7.44 (m, 2H, Ar-H), 7.37 (d, *J* = 8 Hz, 1H, Ar-H), 7.30 (t, *J* = 7.5 Hz, 1H, Ar-H), 7.22 (d, *J* = 8 Hz, 1H, Ar-H), 7.12 (d, *J* = 8 Hz, 1H, Ar-H), 7.04 (t, *J* = 7.5 Hz, 1H, Ar-H), 4.19 (t, *J* = 6.5 Hz, 2H, N-CH_2_), 3.24 (t, *J* = 8 Hz, 2H, N-CH_2_), 2.81 (s, 6H, N-CH_3_), 2.24 (m, 2H, CH_2_). ^13^C-NMR (125 MHz, MeOD, δ ppm): 190.9, 146.8, 145.5, 141.3, 138.5, 136.5, 134.6, 134.2, 132.8, 131.2, 129.3, 129.2, 128.7, 128.6, 128.5, 125.9, 125.4, 124.9, 124.6, 117.5, 115.9, 56.9, 45.2, 43.8, 23.4.

(*E*)-1-(10-(3-(dimethylamino)propyl)-10*H*-phenothiazin-2-yl)-3-(4-fluorophenyl)prop-2-en-1-one (AC9), Yield: 57%. Mp: 138–140 °C. IR (ν cm^–1^, KBr): 2931 (ν_C-H sp3_), 1656 (ν_C=O_), 1593 (ν_C=C Ar_), 1463 (ν_C=C Ar_), 1061 (ν_C-N_). MS: [M + H]^+^ *m*/*z* = 433.1754. ^1^H-NMR (500 MHz, MeOD, δ ppm): 7.79 (m, 2H, Ar-H), 7.75 (d, *J* = 15.5 Hz, 1H, CH=C), 7.68 (d, *J* = 6 Hz, 1H, Ar-H), 7.64 (d, *J* = 15.5 Hz, 1H, CH=C), 7.55 (s, 1H, Ar-H), 7.24 (d, *J* = 8.5 Hz, 1H, Ar-H), 7.22 (d, *J* = 8.5 Hz, 1H, Ar-H), 7.18 (d, *J* = 8 Hz, 1H, Ar-H), 7.16 (d, *J* = 8 Hz, 1H, Ar-H), 7.14 (d, *J* = 8.5 Hz, 1H, Ar-H), 7.04 (m, 1H, Ar-H), 6.98 (m, 1H, Ar-H), 4.04 (t, *J* = 6.5, 2H, N-CH_2_), 2.73 (t, *J* = 7.5, 2H, N-CH_2_), 2.39 (s, 6H, N-CH_3_), 2.04 (m, 2H, CH_2_). ^13^C-NMR (125 MHz, MeOD, δ ppm): 191.1, 134.0, 132.7, 132.6, 132.0, 131.9, 129.0, 128.4, 128.2, 125.5, 124.4, 124.2, 122.7, 117.3, 117.1, 116.9, 115.8, 115.4, 115.2, 56.4, 45.8, 44.7, 24.7.

(*E*)-3-(3-bromophenyl)-1-(10-(3-(dimethylamino)propyl)-10*H*-phenothiazin-2-yl)-3-phenylprop-2-en-1-one (AC10), Yield: 51%. Mp: 178–180 °C. IR (ν cm^–1^, KBr): 2932 (ν_C-H sp3_), 1659 (ν_C=O_), 1598 (ν_C=C Ar_), 1462 (ν_C=C Ar_), 1059 (ν_C-N_)). MS: [M + H]^+^ *m*/*z* = 493.0949 and 495.1410. ^1^H-NMR (500 MHz, MeOD, δ ppm): 7.94 (s, 1H, Ar-H), 7.70 (m, 4H, Ar-H & CH=C), 7.57 (m, 1H, Ar-H), 7.55 (d, *J* = 16 Hz, 1H, CH=C), 7.35 (m, 1H, Ar-H), 7.23 (m, 2H, Ar-H), 7.12 (dd, *J*_1_ = 1 Hz, *J*_2_ = 6.5 Hz, 1H), 7.02 (d, *J* = 8 Hz, 1H, Ar-H), 6.97 (t, *J* = 7.5 Hz, 1H, Ar-H), 4.02 (t, *J* = 6 Hz, 2H, N-CH_2_), 2.52 (t, *J* = 7.5 Hz, 2H, N-CH_2_), 2.22 (s, 6H, N-CH_3_), 1.99 (m, 2H, CH_2_). ^13^C-NMR (125 MHz, MeOD, δ ppm): 190.9, 146.9, 145.7, 144.1, 138.6, 138.3, 134.3, 134.0, 132.2, 131.7, 129.0, 128.6, 128.4, 128.2, 125.3, 124.5, 124.4, 124.1, 124.0, 117.3, 115.8, 57.8, 46.2, 45.3, 25.4.

(*E*)-1-(10-(3-(dimethylamino)propyl)-10*H*-phenothiazin-2-yl)-3-(2-trifluoromethyl)phenyl)prop-2-en-1-one (AC11), Yield: 58%. Mp: Liquid at room temperature. IR (ν cm^–1^, KBr): 2942 (ν_C-H sp3_), 1662 (ν_C=O_), 1592 (ν_C=C Ar_), 1463 (ν_C=C Ar_), 1123 (ν_C-N_). MS: [M + H]^+^ *m*/*z* = 483.1721. ^1^H-NMR (500 MHz, MeOD, δ ppm): 8.13 (d, *J* = 15.5 Hz, 1H, CH=C), 8.10 (m, 1H, Ar-H), 7.77 (m, 1H, Ar-H), 7.73 (d, *J* = 15.5 Hz, 1H, CH=C), 7.70 (m, 2H, Ar-H), 7.59 (m, 1H, Ar-H), 7.54 (d, *J* = 10 Hz, 1H, Ar-H), 7.23 (m, 2H, Ar-H), 7.11 (m, 1H, Ar-H), 7.01 (t, *J* = 8.5 Hz, 1H, Ar-H), 6.96 (m, 1H, Ar-H), 4.00 (m, 2H, N-CH_2_), 2.51 (m, 2H, N-CH_2_), 2.22 (s, 3H, N-CH_3_), 2.21 (s, 3H, N-CH_3_), 1.96 (m, 2H, CH_2_). ^13^C-NMR (125 MHz, MeOD, δ ppm): 190.5, 146.9, 145.6, 140.5, 138.1, 134.9, 134.2, 133.7, 131.2, 129.9, 129.5, 129.0, 128.4, 128.2, 127.1, 127.0, 126.7, 125.2, 124.4, 124.1, 117.3, 115.8, 57.7, 46.2, 45.3, 25.3.

(*E*)-3-(4-(dimethylamino)phenyl)-1-(10-(3-(dimethylamino)propyl)-10*H*-phenothiazin-2-yl)-3-phenylprop-2-en-1-one (AC12), Yield: 39%. Mp: Liquid at room temperature. IR (ν cm^–1^, KBr): 2936 (ν_C-H sp3_), 1647 (ν_C=O_), 1572 (ν_C=C Ar_), 1459 (ν_C=C Ar_), 1168 (ν_C-N_). MS: [M + H]^+^ *m*/*z* = 458.2298. ^1^H-NMR (500 MHz, MeOD, δ ppm): 7.76 (d, *J* = 15.5 Hz, 1H, CH=C), 7.60 (m, 3H, Ar-H), 7.51 (s, 1H, Ar-H), 7.43 (d, *J* = 15.5 Hz, 1H, CH=C), 7.22 (d, *J* = 8 Hz, 1H, Ar-H), 7.20 (d, *J* = 8 Hz, 1H, Ar-H), 7.11 (d, *J* = 7 Hz, 1H, Ar-H), 6.99 (d, *J* = 8 Hz, 1H, Ar-H), 6.95 (t, *J* = 7.5 Hz, 1H, Ar-H), 6.72 (d, *J* = 9 Hz, 2H, Ar-H), 3.98 (t, *J* = 6.5 Hz, 2H, N-CH_2_), 3.01 (s, 6H, N-CH_3_), 2.52 (t, *J* = 7.5 Hz, 2H, N-CH_2_), 2.22 (s, 6H, N-CH_3_), 1.96 (m, 2H, CH_2_). ^13^C-NMR (125 MHz, MeOD, δ ppm): 191.5, 153.9, 147.8, 146.7, 145.8, 139.4, 132.9, 131.9, 128.9, 128.4, 128.1, 125.4, 124.1, 123.7, 117.3, 117.0, 115.8, 113.0, 57.7, 46.1, 45.3, 40.2, 25.3.

(*E*)-3-(4-(benzyloxy)phenyl)-1-(10-(3-(dimethylamino)propyl)-10*H*-phenothiazin-2-yl)-3-phenylprop-2-en-1-one (AC13), Yield: 40%. Mp: 183-185 ^o^C. IR (ν cm^–1^, KBr): 2936 (ν_C-H sp3_), 1655 (ν_C=O_), 1592 (ν_C=C Ar_), 1459 (ν_C=C Ar_), 1247 (ν_C-O_), 1060 (ν_C-N_). MS: [M + H]^+^ *m*/*z* = 521.2243. ^1^H-NMR (500 MHz, MeOD+DMSO, δ ppm): 7.80 (d, *J* = 15.5 Hz, 1H, CH=C), 7.79 (m, 2H, Ar-H), 7.74 (dd, *J*_1_ = 1.5 Hz, *J*_2_ = 8 Hz, 1H, Ar-H), 7.67 (d, *J* = 15.5 Hz, 1H, CH=C), 7.62 (d, *J* = 1.5 Hz, 1H, Ar-H), 7.51 (d, *J* = 7 Hz, 2H, Ar-H), 7.44 (m, 2H, Ar-H), 7.38 (m, 1H, Ar-H), 7.33 (d, *J* = 7.5 Hz, 1H, Ar-H), 7.28 (td, *J*_1_ = 1.5 Hz, *J*_2_ = 8.5 Hz, 1H, Ar-H), 7.20 (dd, *J* = 2 Hz, *J* = 7.5 Hz, 1H), 7.13 (m, 2H, Ar-H), 7.10 (d, *J* = 8 Hz, 1H, Ar-H), 7.00 (td, *J*_1_ = 1.5 Hz, *J*_2_ = 8 Hz, 1H, Ar-H), 5.22 (s, 2H, OCH_2_), 4.10 (t, *J* = 7 Hz, 2H, N-CH_2_), 2.55 (t, *J* = 7 Hz, 2H, N-CH_2_), 2.24 (s, 6H, N-CH_3_), 2.20 (m, 2H, CH_2_). ^13^C-NMR (125 MHz, MeOD + DMSO, δ ppm): 190.8, 162.4, 146.8, 145.9, 145.8, 138.9, 138.3, 133.1, 131.9, 129.2, 129.1, 128.8, 128.4, 128.2, 125.1, 124.3, 124.2, 120.7, 117.4, 116.6, 115.9, 71.1, 57.8, 46.2, 45.6, 25.5.

### 3.3. Acetylcholinesterase Inhibitory Activity Assay

AChE inhibitory activities of chalcones were determined by Ellman’s colourimetric method using purified acetylcolinesterase from electric eel (Type VI) and acetylthiocholine iodide (ATCI) as a substrate and galantamine as a reference. The assay was performed in 96-well microtiter plates in the same condition for both chalcones and control substance. 25 µL of 100 mM sodium phosphate buffer pH 8, 25 µL of sample and 25 µL acetylcholinesterase solutions containing 0.54 U/mL were mixed in each well of the plate and allowed to incubate for 15 min at 25 °C. Subsequently, 25 µL of a solution of ATCI (15 mM, dissolved in water) and 125 µL of 3 mM DTNB (5,5’-dithio-bis-nitro benzoic acid) were added. The absorbance at 405 nm was recorded during the first 5 min of the reaction. A control reaction, which was considered to have 100% activity, was carried out using the same volume of methanol/water instead of tested solutions. All samples and the positive control (galantamine) were assayed in triplicate. Percentages (%) of AChE inhibitions of tested compounds were calculated from the absorbance values as indicated in Equation (1):

% I = [(A_0E_ − A_0_) − (A_c_ − A_0C_)]/(A_0E_ − A_0_) (1)

where I is the percent inhibition of acetylcholinesterase; A_0E_ is the absorbance value of the control blank sample with enzyme; A_0_ is the absorbance value of blank sample; Ac is the absorbance value of the tested sample; A_0C_ is the absorbance value of blank test sample. 

The content of each sample was indicated in Table 6.

Linear recurrent equations indicating the correlation between common logarithm of the concentration of investigated compounds (µM) and their percentages of AChE inhibition (%) were built, from which the IC_50_ values (concentration that inhibits 50% AChE activity) of studied chalcones were extrapolated. The method was as described earlier [81].

### 3.4. β-secretase Inhibitory Activity Assay

β-secretase (BACE-1) Activity Detection Kit (Fluorescent) was purchased from Sigma–Aldrich and used to determine the effect of the synthesized chalcones on β-secretase activity. The assay was carried out according to the manufacturer’s protocol. The enzyme solution (0.3 units/uL, 2 μL) was reacted with the 50 μM of the substrate (7-methoxycumarin- 4-acetyl-(Asn670, Lue671)-amyloid β/A4 precursor protein 770 fragment 667–676-(2,4-dinitrophenyl))Lys-Arg-Arg amide trifluoroacetate salt and sulfated polysaccharide samples (2–5 mg/mL) in a fluorescence assay buffer in different wells. Baseline readings were measure immediately on a Hitachi F-7000 (excitation: 320 nm; emission: 405 nm) fluorescence spectrophotometer and repeated after 2 h incubation at 37 °C. Quercetin was used as the positive control. The method was as described earlier [82,83].

### 3.5. Building 2D-QSAR Model

#### 3.5.1. Data Collection and Ligand Preparation

The dataset of AChE inhibitors was obtained from CheMBL database [60], and the BACE-1 inhibitor database was collected from reported literatures [61,62,63,64,65,66,67,68,69,70,71,72]. Initial processing of input data was then carried out, including rejecting substances with similar structures (use Cluster codes tool in MOE 2008.10 [84]), retaining compounds with the same bioassay method, and correcting IC_50_ appropriately. A total of 72 derivatives, with AChE inhibitory activities which were determined using Ellman’s method on the enzyme of *Electrophorus electricus* and galantamine as a reference, were finally obtained. The total number of BACE-1 inhibitors with the same FRET (Fluorescence Resonance Energy Transfer) test method was 215 (Supplementary Tables S4 and S5). IC_50_ values were then converted into pIC_50_ for convenient calculations. The structures were built directly in Sybyl X 2.0 [85]. These final datasets were used to build 2D-QSAR models with optimal molecular descriptors.

#### 3.5.2. Molecular Descriptors Calculation and Processing

One hundred and eighty-four 2D molecular descriptors were calculated in MOE 2008.10, and then processed to eliminate redundant or irrelevant features for improving model quality and reducing computational time consumption [86]. Firstly, the molecular descriptors were filtered and removed useless or correlated attributes using RapidMiner 5.3.008 [87]. Subsequently, they were processed further by BestFirst search method of Weka 3.8 [88] to figure out parameters having the optimal values with 10-fold cross validation. Selected molecular descriptors used for building 2D-QSAR models in this study are described in detail in the Supplementary Table S7.

#### 3.5.3. Database Division into Training Set and Validation Set

The database was divided into training set and validation set in a ratio of 70% to 30% using randomization method. The Rand function in MOE 2008.10 was used to split randomly the database of compounds, each of which was assigned a random number between 0 and 1.

#### 3.5.4. Model Building and Validation

2D-QSAR model was built using the partial least square (PLS) method and then validated by the values of RMSE (root-mean-square error), R^2^ (squared correlation coefficient), RMSE_LOO_ (cross-validated root-mean-square error), Q^2^_LOO_ (cross-validated squared correlation coefficient), and more widely used metrics $r_{m}^{2}$, ${r^{\prime }}_{m}^{2}$, $\bar{r_{m}^{2}}$, $\Delta r_{m}^{2}$; R^2^_PRED_, or CCC (concordance correlation coefficient) [89]. The equation used for calculation of these metrics are provided in the Supplementary Table S6.

### 3.6. Molecular Docking Procedure

Docking was firstly performed on 3 conformations of co-crystallized ligand to validate the procedure. The RMSD (Root-Mean-Square Deviation) value between re-docked conformations and the original bound ligand in the co-crystalized complex which was ≤ 1.5 Å would indicate the reliability of the binding ability prediction of new ligands [90]. Docking process was performed using complexes 1DX6 of AChE (resolution 2.3 Å) and 5HU1 (resolution 1.5 Å) of BACE-1 downloaded form Protein Data Bank [91] and FlexX program in BioSolveIT LeadIt [92] with default settings. This program applied the flexible-based docking methodology, in which the ligand was treated as a flexible component and the protein was kept rigid during docking process. FlexX used an incremental construction algorithm for the seach of ligand conformations. The base fragment was first placed into the active site by matching interaction geometries between the ligand and protein. Then, the remains were gradually built-up in conformity with a set of predefined rotatable torsion angles to account for ligand flexibility. The FlexX utilised empirical scoring functions to score and rank the docking poses [93,94].

The interactions between chalcone molecules and their target were rendered and analyzed in MOE 2008.10 program (hydrogen bonds, π–π interactions, cation–π interactions, ionic interactions). Moreover, van der Waals surface interactions were detected by the contact of hydrophilic and lipophilic surfaces of the ligands with those of binding points.

## 4. Conclusions

In this study, 13 *N*-substituted-4-phenothiazine-chalcone derivatives were synthesized and tested for AChE and BACE-1 inhibition with the pIC_50_ values of 3.73–5.96 (AChE) and 5.20–6.81 (BACE-1). Two 2D-QSAR models were built from curated data and validated through evaluation metrics. These models could be used to predict the bioactivities of chemical compounds with high reliabilities. The synthesized substances were considered as an external validation set to evaluate the developed 2D-QSAR model for AChE and BACE-1 inhibitors with relatively high correlations between the observed and estimated bioactivities (R^2^ = 0.62 for AChE and R^2^ = 0.83 for BACE-1). Eleven of the synthesized derivatives were newly discovered (AC2, AC4–13). The molecular docking model developed in the present work could be used to explain the difference in the observed biological activity compared to the predicted value as the 2D-QSAR models could not clearly interpreted. Thus, the combination of 2D and 3D models, between ligand- and structure-based drug designs, could allow for the prediction of the biological activities of chemical substances more accurately. Among the studied *N*-substituted-4-phenothiazine-chalcones, AC4 and AC12 were two derivatives with the strongest inhibitory activities against both AChE and BACE-1. These substances could be used in further studies. In addition, many substances need to be synthesized and tested for AChE and BACE-1 inhibitory activities to further validate the performance of the developed 2D-QSAR models.

## Figures and Tables

**Table 1:** Acetylcholinesterase (AChE) and beta-secretase (BACE-1) inhibitory activities of synthesized *N*-substituted-4-phenothiazine-chalcones.

	AChE			BACE-1		
Compound	IC_50_ (µM) *	pIC_50_		IC_50_ (µM) *	pIC_50_	
		Observed *	Predicted		Observed *	Predicted
AC1	33.88 ± 1.45	4.47 ± 0.02	4.80	6.34 ± 0.46	5.20 ± 0.03	6.52
AC2	30.90 ± 2.10	4.51 ± 0.03	4.74	3.00 ± 0.00	5.52 ± 0.00	7.38
AC3	11.48 ± 1.29	4.94 ± 0.05	4.74	4.48 ± 0.40	5.35 ± 0.04	7.37
**AC4**	**3.63** **± 0.61**	**5.44** **± 0.08**	**4.71**	**0.16** **± 0.03**	**6.81** **± 0.09**	**8.49**
AC5	60.26 ± 1.84	4.22 ± 0.01	4.82	1.24 ± 0.12	5.91 ± 0.04	7.25
AC6	19.95 ± 1.57	4.70 ± 0.03	4.83	0.50 ± 0.00	6.30 ± 0.00	7.68
AC7	40.74 ± 2.48	4.39 ± 0.03	4.76	0.45 ± 0.04	6.35 ± 0.04	7.63
AC8	15.85 ± 1.08	4.80 ± 0.03	4.82	2.97 ± 0.05	5.53 ± 0.01	7.15
AC9	25.12 ± 0.83	4.60 ± 0.01	4.76	3.72 ± 0.21	5.43 ± 0.02	7.12
AC10	24.55 ± 1.09	4.61 ± 0.02	4.81	1.99 ± 0.21	5.70 ± 0.05	7.28
AC11	186.21 ± 4.52	3.73 ± 0.01	4.59	0.40 ± 0.00	6.40 ± 0.00	7.99
**AC12**	**1.10** **± 0.24**	**5.96** **± 0.10**	**4.81**	**0.35** **± 0.04**	**6.46 ± 0.05**	**8.08**
AC13	11.75 ± 0.63	4.93 ± 0.02	4.63	3.03 ± 0.21	5.52 ± 0.03	7.20
Galanthamine	1.26 ± 0.12	5.90 ± 0.04	5.14	-	-	-
Quercetin	-	-	-	9.55 ± 0.37	5.02 ± 0.02	5.24

IC_50_: the half maximal inhibitory concentration, pIC_50_ = −logIC_50_. * Reported with standard deviation (SD).

**Table 2:** Results of molecular docking study of the synthesized *N*-substituted-4-phenothiazine-chalcones on AChE (1DX6) and BACE-1 (5HU1).

Comp.	pIC_50_ (AChE)		pIC_50_ (BACE-1)		Docking Score (kJ·mol^−1^) (AChE: 1DX6)	Docking Score (kJ·mol^−1^) (BACE-1: 5HU1 chain A, B)
	Obs. *	Pred.	Obs. *	Pred.		
AC1	4.47 ± 0.02	4.80	5.20 ± 0.03	6.52	−25.83	−17.82; −17.69
AC2	4.51 ± 0.03	4.74	5.52 ± 0.00	7.38	−26.03	−17.22; −17.81
AC3	4.94 ± 0.05	4.74	5.35 ± 0.04	7.37	−27.67	−16.28; −15.37
AC4	5.44 ± 0.08	4.71	6.81 ± 0.09	8.49	−24.49	−16.71; −13.92
AC5	4.22 ± 0.01	4.82	5.91 ± 0.04	7.25	−17.71	−20.77; −16.87
AC6	4.70 ± 0.03	4.83	6.30 ± 0.00	7.68	−25.45	−18.79; −16.73
AC7	4.39 ± 0.03	4.76	6.35 ± 0.04	7.63	−17.94	−19.51; −16.85
AC8	4.80 ± 0.03	4.82	5.53 ± 0.01	7.15	−26.27	−20.36; −16.81
AC9	4.60 ± 0.01	4.76	5.43 ± 0.02	7.12	−27.80	−20.97; −18.06
AC10	4.61 ± 0.02	4.81	5.70 ± 0.05	7.28	−27.30	−22.51; −20.81
AC11	3.73 ± 0.01	4.59	6.40 ± 0.00	7.99	−25.33	−19.35; −16.41
AC12	5.96 ± 0.10	4.81	6.46 ± 0.05	8.08	−22.15	−18.25; −16.18
AC13	4.93 ± 0.02	4.63	5.52 ± 0.03	7.20	−23.30	−11.50; −14.09
Galantamine	5.90 ± 0.04	5.14	-	-	−28.53	-
Verubecestat	-	-	-	7.66	-	−24.95; −22.43
Quercetin	-	-	5.02 ± 0.02	5.24	-	−22.23; −23.95

Comp.: Compound, Obs.: Observed, Pred.: Predicted. * Reported with standard deviation (SD).

**Table 3:** 2D-QSAR models for AChE and BACE-1 inhibitors.

AChE												
pIC_50_ = −0.928 + (2.348 × BCUT_SLOGP_3) − (0.150 × reactive) − (0.004 × PEOE_VSA + 1) − (0.005 × PEOE_VSA−3) − (0.002 × SlogP_VSA2) − (0.004 × SMR_VSA2)												
**Internal Validation**					**External Validation**							
N	RMSE	R^2^	RMSE_LOO_	Q^2^_LOO_	N	RMSE	R^2^	R^2^_(PRED)_	$r_{m}^{2}$	$\bar{r_{m}^{2}}$	$\Delta r_{m}^{2}$	CCC
50	0.18	0.70	0.22	0.57	22	0.16	0.78	0.78	0.64	0.69	0.11	0.88
**BACE-1**												
pIC_50_ = 1.268 + (0.870 × petitjean) + (6.370 × BCUT_PEOE_1) + (3.305 × a_ICM) − (0.478 × chiral_u) + (0.085 × rings) + (0.157 × a_Nn) + (0.006 × PEOE_VSA − 0) + (0.022 × PEOE_VSA − 6) − (0.260 × logS) + (0.009 × SlogP_VSA3) + (0.009 × SlogP_VSA5)												
**Internal Validation**					**External Validation**							
N	RMSE	R^2^	RMSE_LOO_	Q^2^_LOO_	N	RMSE	R^2^	R^2^_(PRED)_	$r_{m}^{2}$	$\bar{r_{m}^{2}}$	$\Delta r_{m}^{2}$	CCC
150	0.37	0.80	0.40	0.77	65	0.41	0.83	0.81	0.79	0.76	0.05	0.91

N: number of compounds; RMSE (root-mean-square error), R^2^ (squared correlation coefficient), RMSE_LOO_ (cross-validated root-mean-square error), Q^2^_LOO_ (cross-validated squared correlation coefficient), CCC (concordance correlation coefficient), and $r_{m}^{2},\;\bar{r_{m}^{2}},\;\Delta r_{m\;}^{2}$ (validation metrics suggested by Roy et al. [74]).

**Table 4:** Comparison of this study with previous published works on 2D-QSAR model for AChE.

Source	Model	Training Set			Validation Set	
		N	R^2^	Q^2^	N	R^2^_PRED_
**This study**	**PLS**	**55**	**0.70**	**0.57**	**22**	**0.78**
Roy et al. 2018 [77]	MLR	284	0.52–0.74	0.50–0.71	142	0.50–0.63
Niraj et al. 2015 [78]	PLS	24	0.78	0.70	11	0.66

PLS: Partial least squares; MLR: Multiple linear regression; GFA: Genetic Function Approximation; N: number of compounds.

**Table 5:** Comparison of this study with previous published works on 2D-QSAR model for BACE1.

Source	Model	Training Set			Validation Set	
		N	R^2^	Q^2^	n	R^2^_PRED_
**This study**	**PLS**	**150**	**0.80**	**0.77**	**65**	**0.81**
Ambure et al. 2016 [79]	PLS	52	0.83	0.76	22	0.81
Ambure et al. 2016 [79]	MLR	51	0.83	0.76	22	0.80
Hossain et al. 2013 [80]	CoMFA	71	1.00	0.77	35	0.77
Hossain et al. 2013 [80]	CoMSIA	71	1.00	0.73	35	0.71
Hossain et al. 2013 [80]	PLS	71	0.94	0.79	35	0.71
Roy et al. 2018 [77]	MLR	51	0.76–0.83	0.71–0.76	23	0.75–0.91

PLS: Partial least squares; MLR: Multiple linear regression; CoMFA: Comparative molecular field analysis; CoMSIA: Comparative similarity indices analysis; LHM: Linear heuristic method; N: number of compounds.

**Table 6:** The content of each sample in AChE in vitro assay.

Samples	ATCI	DTNB	Buffer	Chalcone	AChE
Control blank sample with enzyme (A_0E_)	+	+	+	−	+
Blank sample (A_0_)	+	+	+	−	−
Tested sample (A_C_)	+	+	+	+	+
Blank test sample (A_0C_)	+	+	+	+	−

+: present; −: absent.

## Supplementary Materials

The following are available online. Table S1. Results of re-docking (RMSD in Å), Table S2: Docking results and ligand interaction (Co-crystallized 1DX6), Table S3. Docking results and ligand interaction (Co-crystallized 5HU1 chain A and B), Table S4. Dataset of 72 compounds used in the building of 2D-QSAR model for AChE inhibitors, Table S5. Dataset of 215 compounds used in the building of 2D-QSAR model for BACE-1 inhibitors, Table S6. Equations for calculation of 2D-QSAR validation metrics, Table S7. Selected descriptors used for building 2D-QSAR models, Table S8. Values of selected descriptors used in prediction of pIC_50_ of the synthesized chalcone derivatives (AChE), Table S9. Values of selected descriptors used in prediction of pIC_50_ of the synthesized chalcone derivatives (BACE-1, Table S10. Spectra of synthesized chalcone derivatives, Figure S1. Interactions of co-crystallized ligand in the protein complex 1DX6 (2D), Figure S2. Interactions of co-crystallized ligand in the protein complex 1DX6 (3D), Figure S3. Alignment of re-dock ligands with the native one in the binding pocket of of 1DX6, Figure S4. Interactions of co-crystallized ligand in the protein complex 5HU1-chain A (2D), Figure S5. Interactions of co-crystallized ligand in the protein complex 5HU1-chain A (3D), Figure S6. Alignment of re-dock ligands with the native one in the binding pocket of of 5HU1-chain A, Figure S7. Interactions of co-crystallized ligand in the protein complex 5HU1-chain B (2D), Figure S8. Interactions of co-crystallized ligand in the protein complex 5HU1-chain B (3D), Figure S9. Alignment of re-dock ligands with the native one in the binding pocket of of 5HU1-chain B, Figure S10. The linear regression between docking score and pIC_50_ on AChE of synthesized chalcone derivatives (A. observed values from **AC1–3** and **AC5–10**, B. predicted values from **AC1**, **AC4**, **AC6**, **AC8**, **AC10**, **AC13**).
