# Supplementary material for: Synthesis, In Silico and In Vitro Evaluation for Acetylcholinesterase and BACE-1 Inhibitory Activity of Some N-Substituted-4-Phenothiazine-Chalcones
Source: Molecules. 2020 Aug 27;25(17):3916. doi: 10.3390/molecules25173916 (PMC7504348; doi:10.3390/molecules25173916)
Supplement: Supplementary file 1 [file molecules-25-03916-s001.pdf]

SUPPORTING INFORMATION

Synthesis, In Silico and In Vitro Evaluation for Acetylcholinesterase and BACE-1 Inhibitory Activity of Some *N*-Substituted-4-Phenothiazine-Chalcones

Thai-Son Tran [1,2,a], Minh-Tri Le [1,3,a],* Thi-Cam-Vi Nguyen [4], The-Huan Tran [2], Thanh-Dao Tran [1] and Khac-Minh Thai [1,*]

[1] Department of Medicinal Chemistry, Faculty of Pharmacy, University of Medicine and Pharmacy at Ho Chi Minh City, Ho Chi Minh City 700000, Vietnam; ttson@huemed-univ.edu.vn; daott@ump.edu.vn
[2] Department of Pharmaceutical Chemistry, Faculty of Pharmacy, College of Medicine and Pharmacy, Hue University, Hue City 530000, Vietnam; tthuan@hueuni.edu.vn
[3] School of Medicine, Vietnam National University Ho Chi Minh City, 700000 Ho Chi Minh City, Vietnam
[4] Ton Duc Thang University, Nguyen Huu Tho St., Tan Phong Ward, Dist. 7, Ho Chi Minh City 70000, Viet Nam; nguyenthicamvi@tdtu.edu.vn
[a] T-S T and M-T. L contributed equally to this work and are co-first authors.
* Correspondence: thaikhacminh@gmail.com, thaikhacminh@ump.edu.vn (K-M. T) and leminhtri@ump.edu.vn (M-T L). Tel.: +84-903-718-190 (M-T.L.); +84-28-3855-2225 or +84-909-680-385 (K-M.T.); Fax: +84-28-3822-5435 (K-M.T.)

Table S1. Results of re-docking (RMSD in Å)

Table S2: Docking results and ligand interaction (Co-crystallized 1DX6)

Table S3. Docking results and ligand interaction (Co-crystallized 5HU1 chain A and B)

Table S4. Dataset of 72 compounds used in the building of 2D-QSAR model for AChE inhibitors

Table S5. Dataset of 215 compounds used in the building of 2D-QSAR model for BACE-1 inhibitors

Table S6. Equations for calculation of 2D-QSAR validation metrics

Table S7. Selected descriptors used for building 2D-QSAR models

Table S8. Values of selected descriptors used in prediction of pIC_{50} of the synthesized chalcone derivatives (AChE)

Table S9. Values of selected descriptors used in prediction of pIC_{50} of the synthesized chalcone derivatives (BACE-1)

Table S10. Spectra of synthesized chalcone derivatives

Figure S1. Interactions of co-crystallized ligand in the protein complex 1DX6 (2D)

Figure S2. Interactions of co-crystallized ligand in the protein complex 1DX6 (3D)

Figure S3. Alignment of re-dock ligands with the native one in the binding pocket of of 1DX6

Figure S4. Interactions of co-crystallized ligand in the protein complex 5HU1-chain A (2D)

Figure S5. Interactions of co-crystallized ligand in the protein complex 5HU1-chain A (3D)

Figure S6. Alignment of re-dock ligands with the native one in the binding pocket of of 5HU1-chain A

Figure S7. Interactions of co-crystallized ligand in the protein complex 5HU1-chain B (2D)

Figure S8. Interactions of co-crystallized ligand in the protein complex 5HU1-chain B (3D)

Figure S9. Alignment of re-dock ligands with the native one in the binding pocket of of 5HU1-chain B

Figure S10. The linear regression between docking score and pIC_{50} on AChE of synthesized chalcone derivatives (A. observed values from **AC1-3** and **AC5-10**, B. predicted values from **AC1, AC4, AC6, AC8, AC10, AC13**)

Table S1. Results of re-docking (RMSD in Å)

Ligand	AChE	BACE-1	
	1DX6	5HU1 (chain A)	5HU1 (chain B)
1	0.49	0.71	0.53
2	0.50	0.52	0.87
3	0.89	0.89	0.96

- *Ligand 1: separated from the complex (native form, not prepared).*
- *Ligand 2: separated from the complex and re-prepared using appropriate procedure indicated in the section of Materials and Methods*
- *Ligand 3: built and prepared from the beginning.*

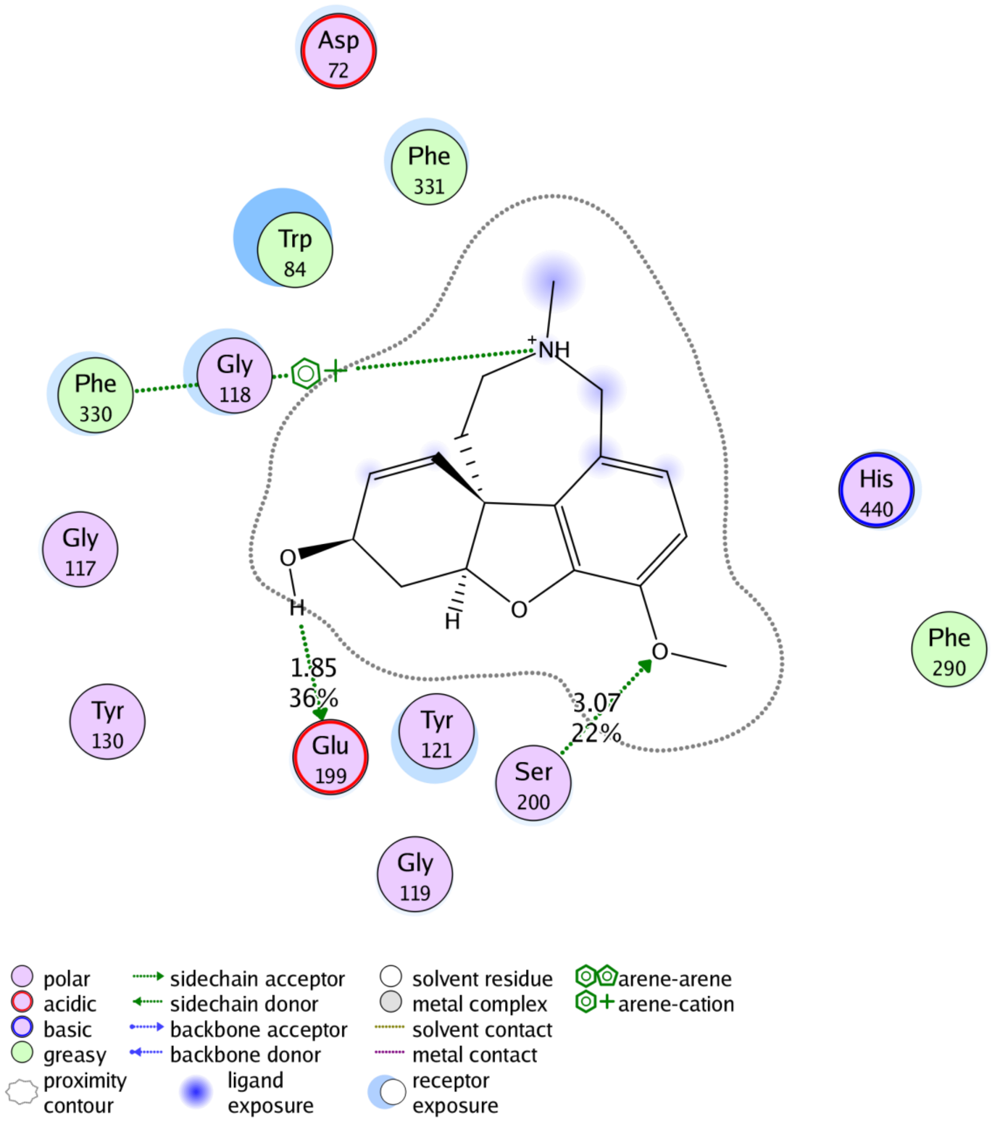

Figure S1. Interactions of co-crystallized ligand in the protein complex 1DX6 (2D)

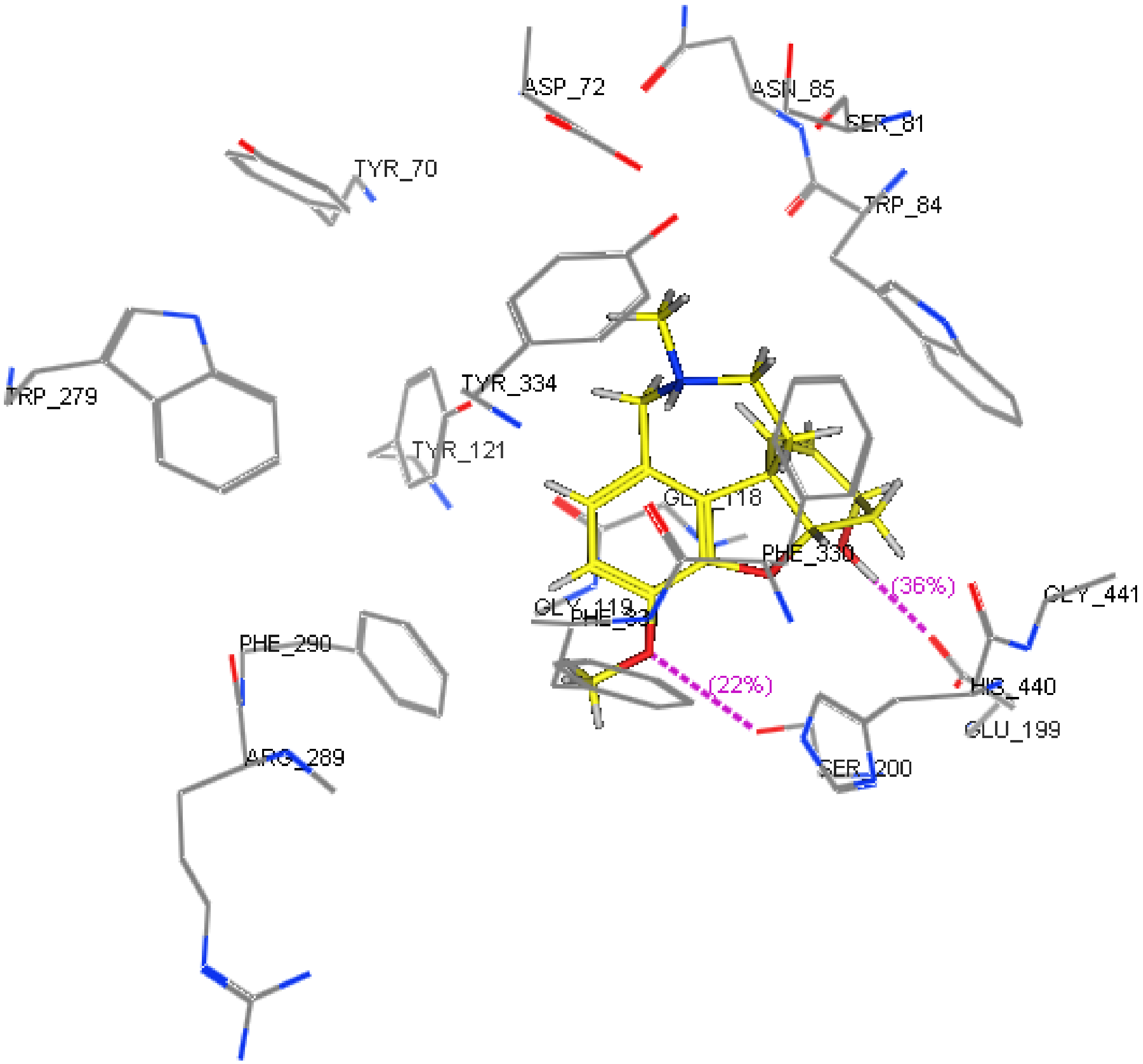

Figure S2. Interactions of co-crystallized ligand in the protein complex 1DX6 (3D)

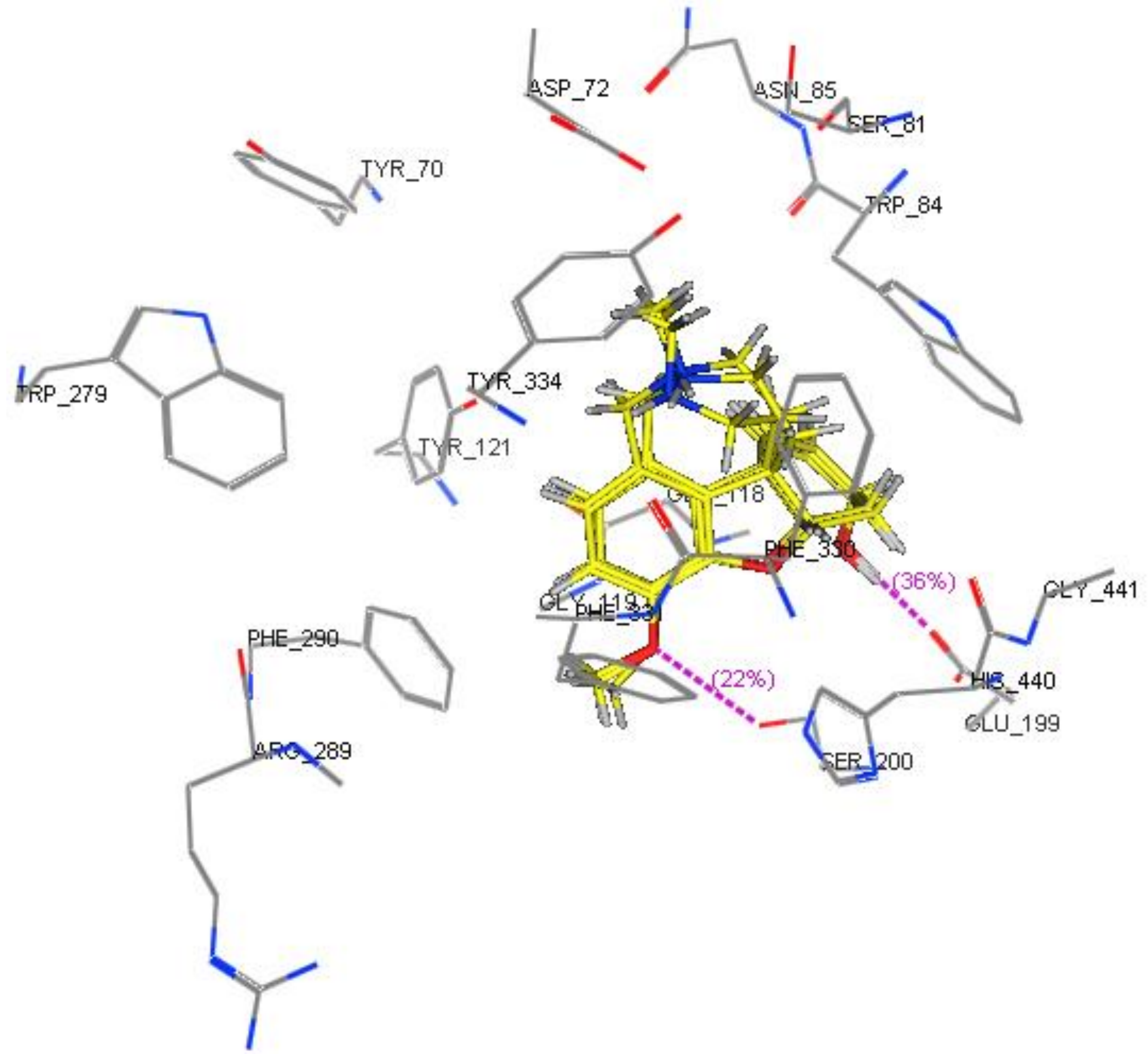

Figure S3. Alignment of re-dock ligands with the native one in the binding pocket of of 1DX6

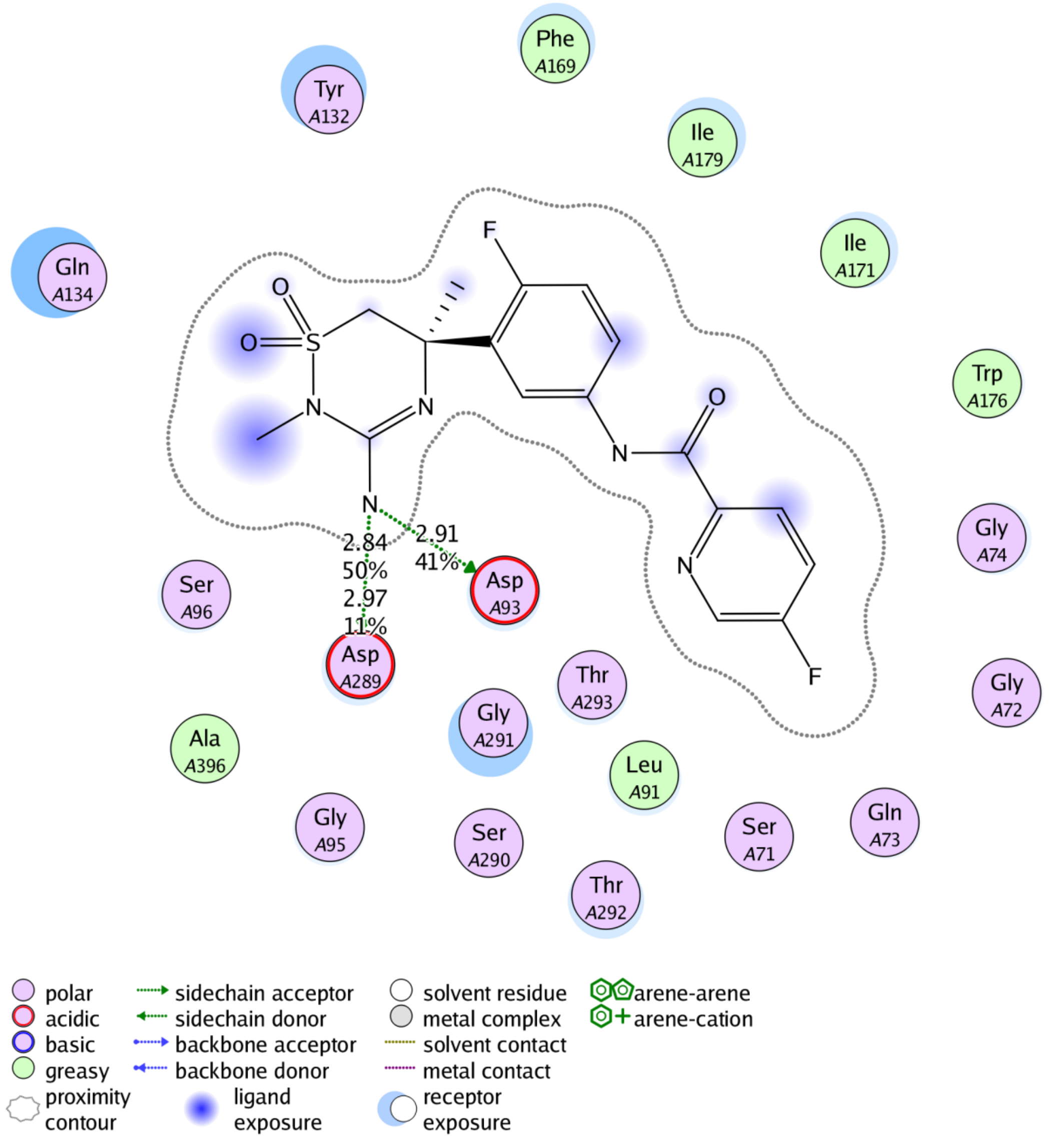

Figure S4. Interactions of co-crystallized ligand in the protein complex 5HU1-chain A (2D)

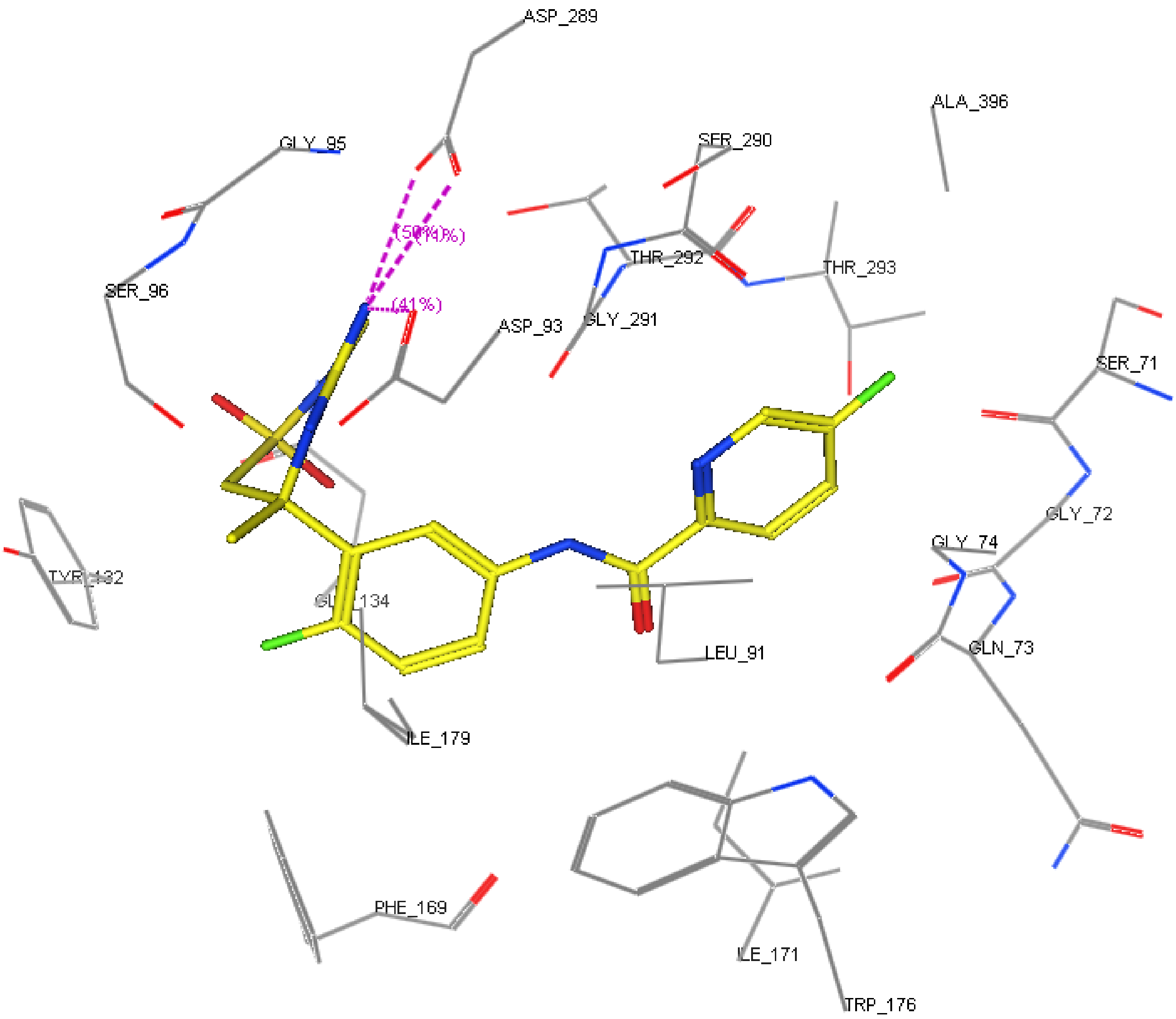

Figure S5. Interactions of co-crystallized ligand in the protein complex 5HU1-chain A (3D)

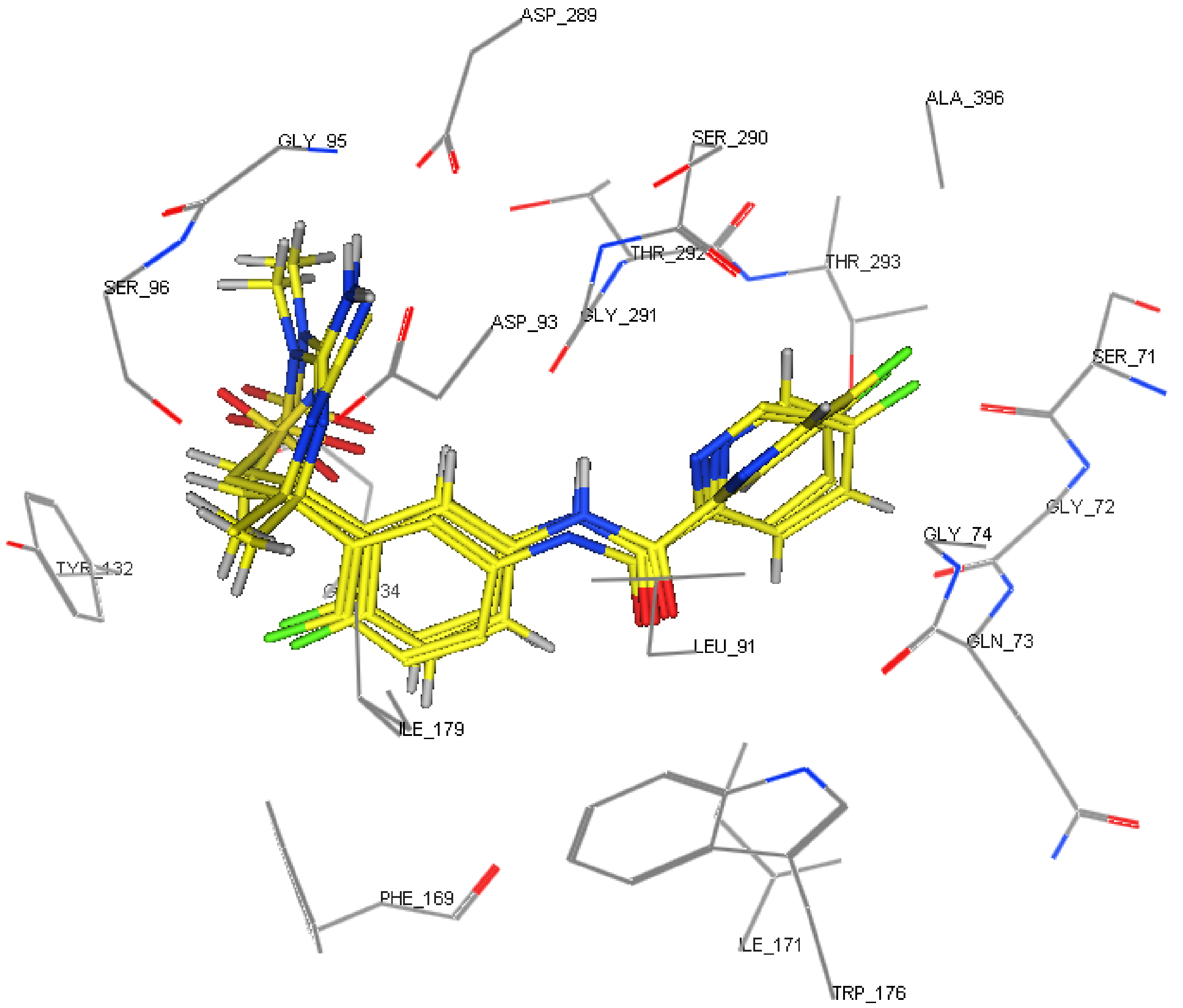

Figure S6. Alignment of re-dock ligands with the native one in the binding pocket of of 5HU1-chain A

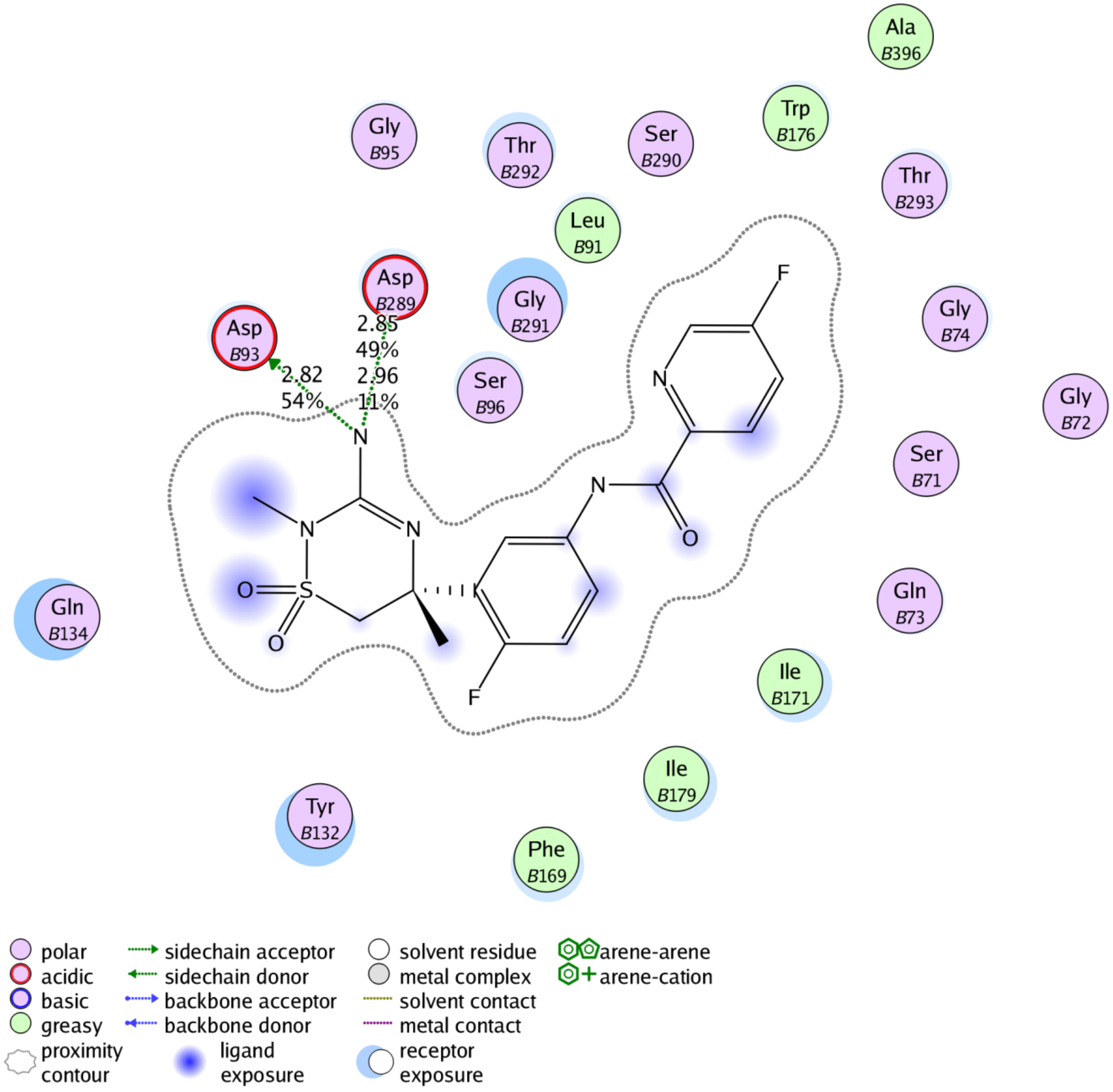

Figure S7. Interactions of co-crystallized ligand in the protein complex 5HU1-chain B (2D)

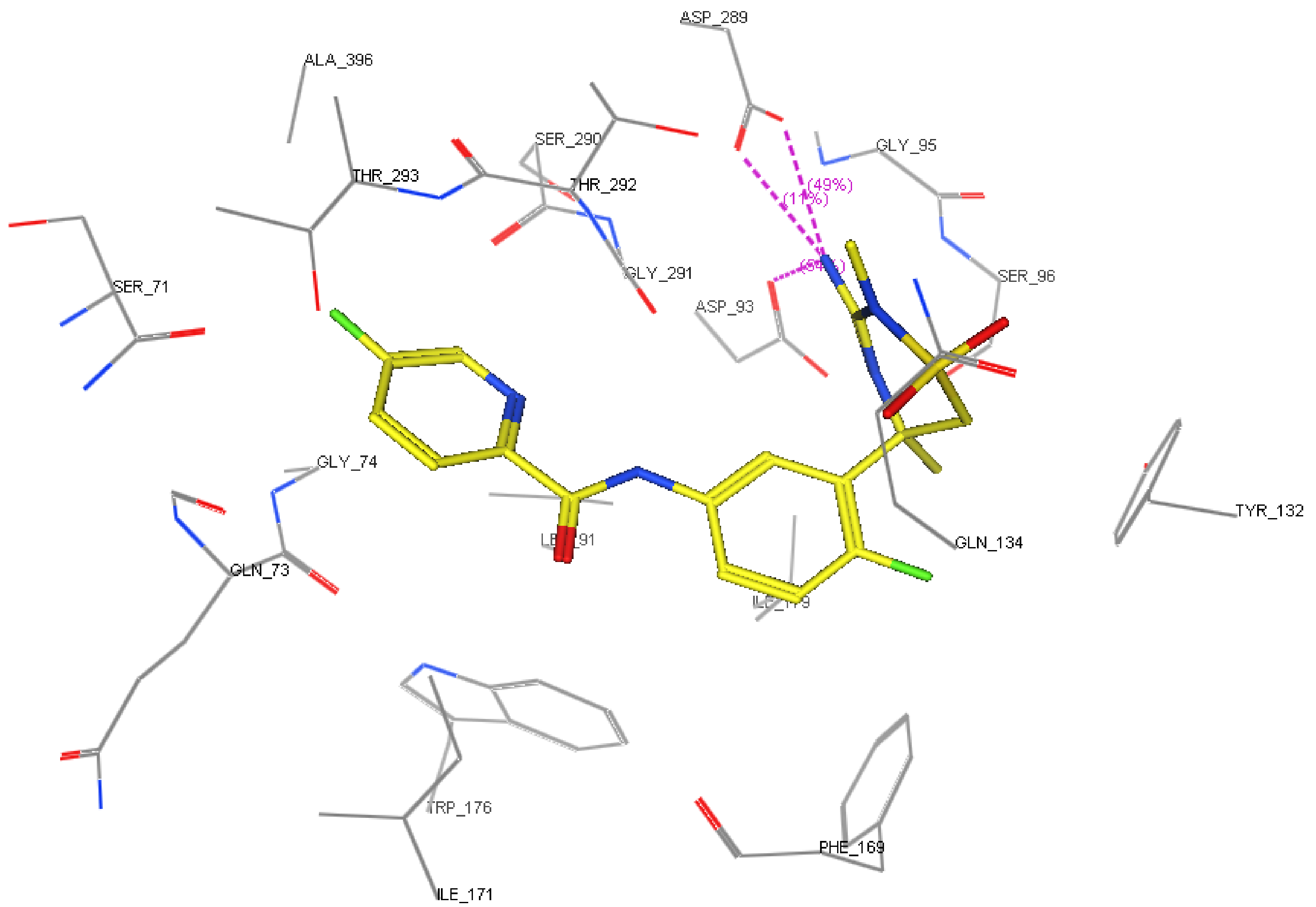

Figure S8. Interactions of co-crystallized ligand in the protein complex 5HU1-chain B (3D)

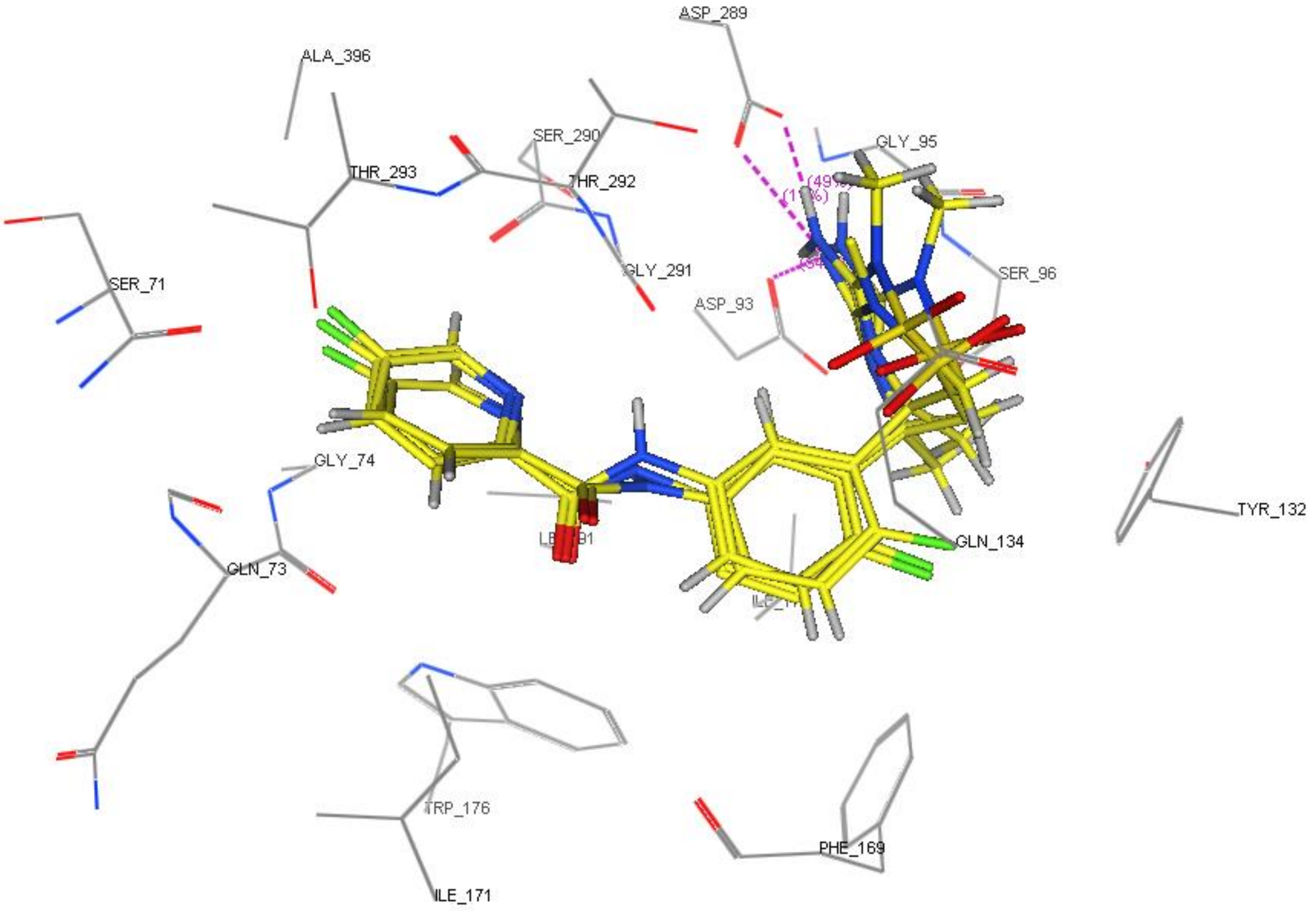

Figure S9. Alignment of re-dock ligands with the native one in the binding pocket of of 5HU1-chain B

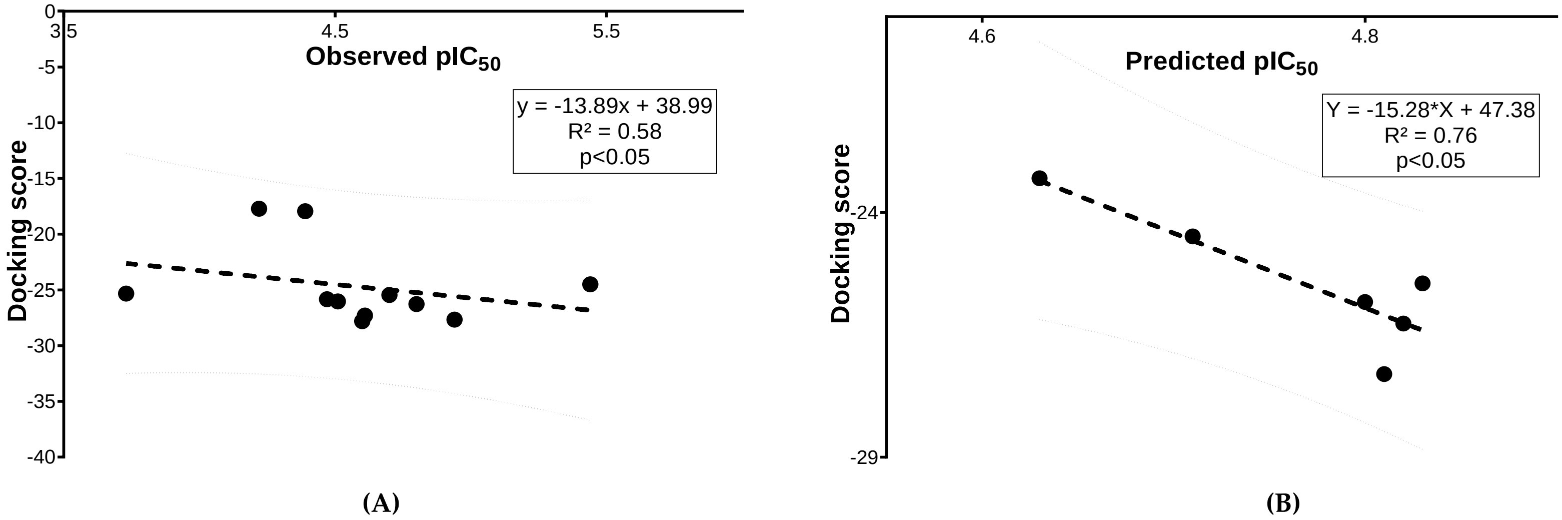

Figure S10. The linear regression between docking score and pIC_{50} on AChE of synthesized chalcone derivatives (A. observed values from **AC1-3** and **AC5-10**, B. predicted values from **AC1, AC4, AC6, AC8, AC10, AC13**)

Table S2: Docking results and ligand interaction (Co-crystallized 1DX6)

Compound	pIC_{50}		Docking score ($kJ.mol^{-1}$)	Ligand interaction
	Observed	Predicted		
AC1	4.47	4.80	-25.83	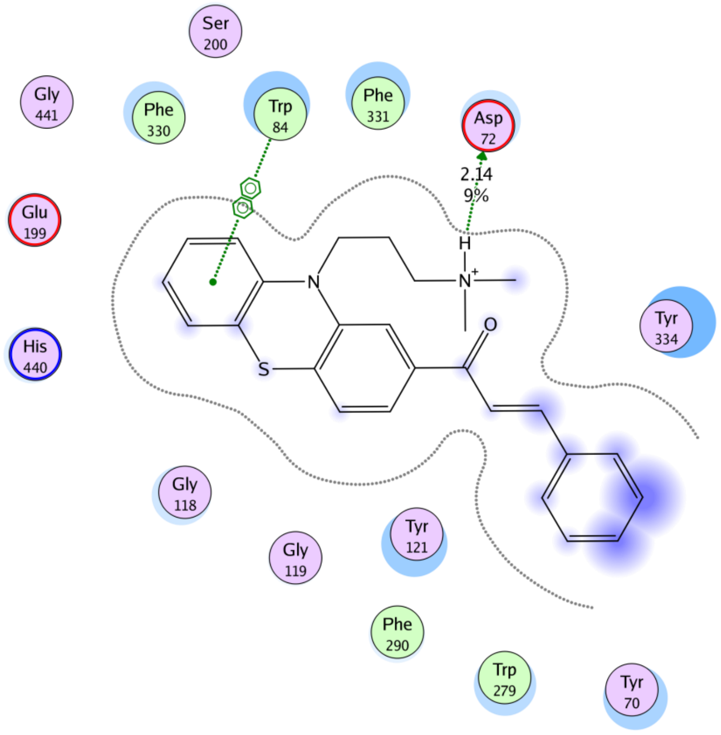 Trp84 (arene-arene), Asp72 (hydrogen bond, score: 9%, length: 2.14 Å)

<table>
<tr><th></th><th></th><th></th><th></th><th></th></tr>
<tr><td>AC2</td><td>4.51</td><td>4.74</td><td>-26.03</td><td>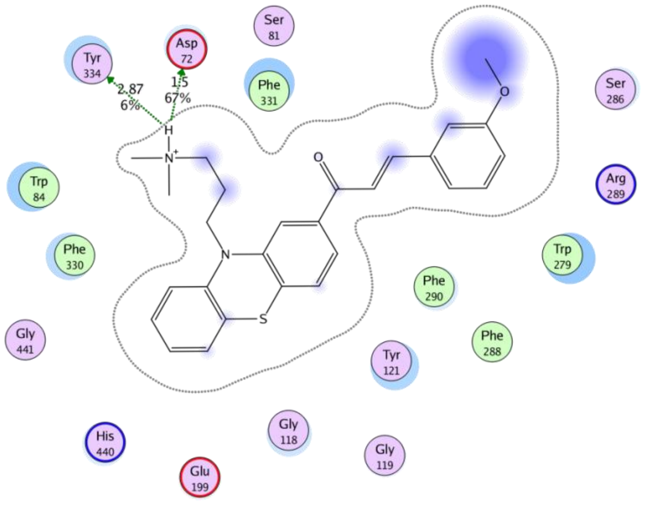

Tyr334 (hydrogen bond, score: 6%, length: 2.87 Å), Asp72 (hydrogen bond, score: 67%, length: 1.5 Å)</td></tr>
<tr><td>AC3</td><td>4.94</td><td>4.74</td><td>-27.67</td><td>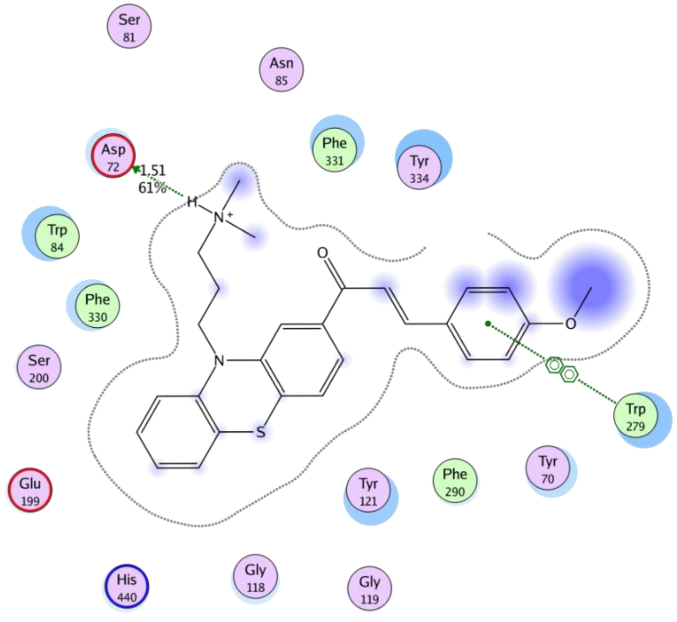

Trp279 (arene-arene), Asp72 (hydrogen bond, score: 61%, length: 1.51 Å)</td></tr>
</table>

AC4	5.44	4.71	-24.49	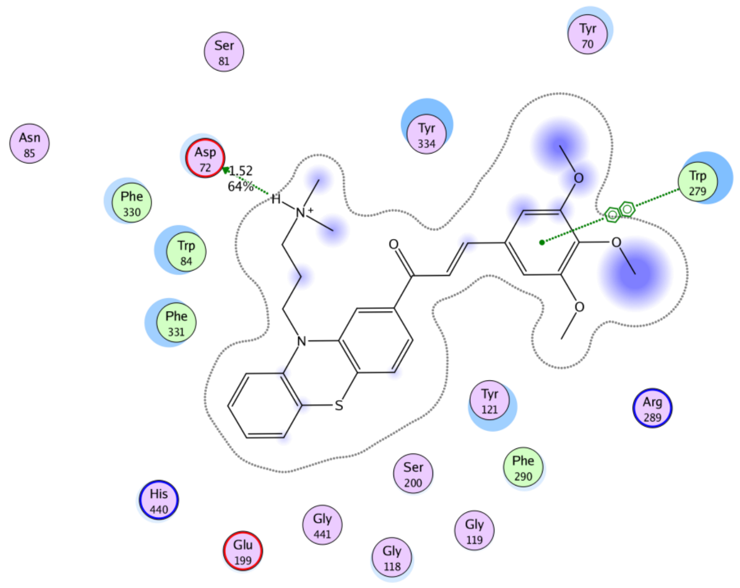 Trp279 (arene-arene), Asp72 (hydrogen bond, score: 64%, length: 1.52 Å)
AC5	4.22	4.82	-17.71	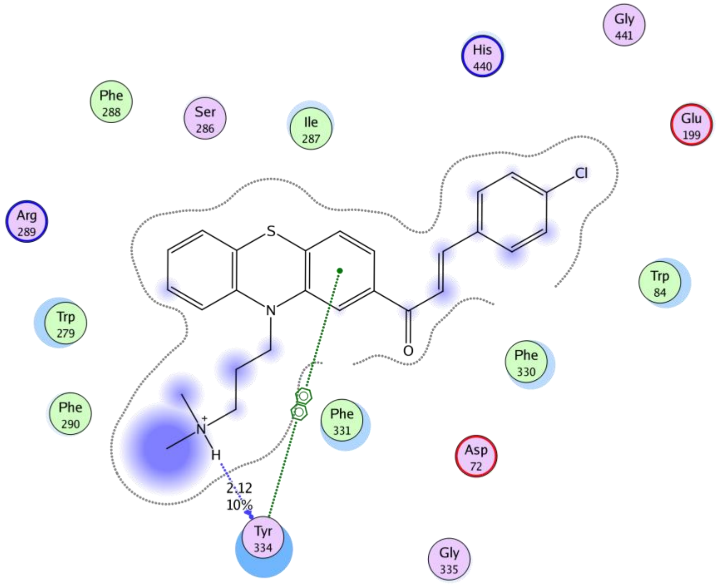 Tyr334 (arene-arene; hydrogen bond, score 10%, length: 2.12 Å)

AC6	4.70	4.83	-25.45	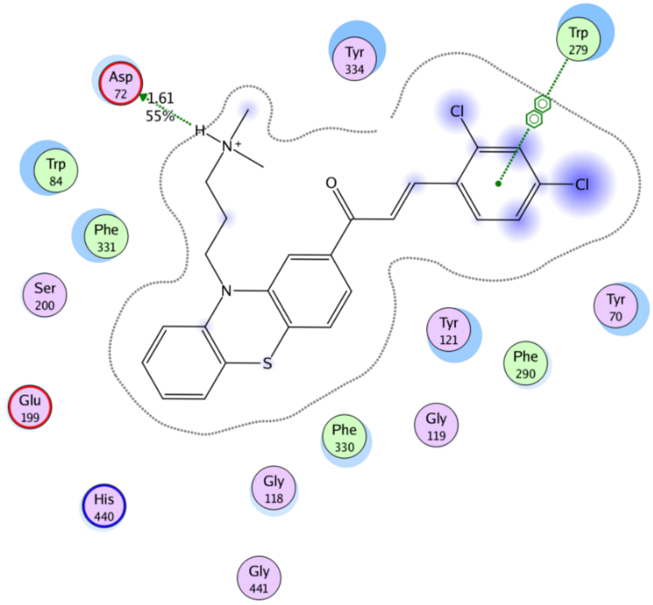 Trp279 (arene-arene), Asp72 (hydrogen bond, score: 55%, length: 1.61 Å)
AC7	4.39	4.76	-17.94	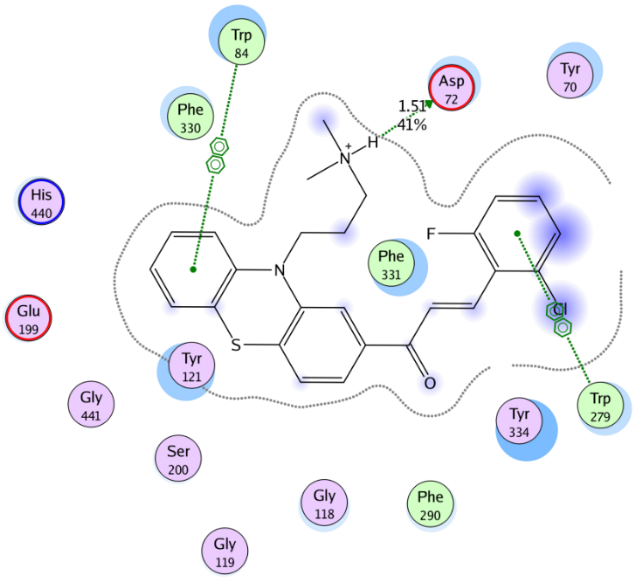 Trp279 (arene-arene), Trp84 (arene-arene), Asp72 (hydrogen bond, score: 41%, length: 1.51 Å)

AC8	4.80	4.82	-26.27	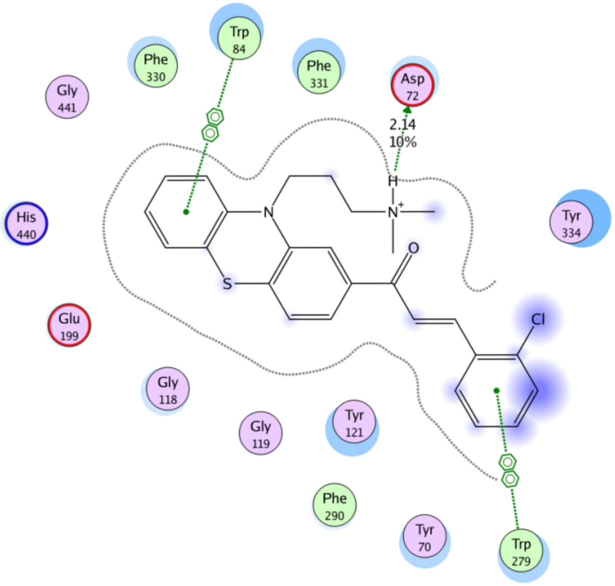 Trp279 (arene-arene), Trp84 (arene-arene), Asp72 (hydrogen bond, score: 10%, length: 2.14 Å)
AC9	4.60	4.76	-27.80	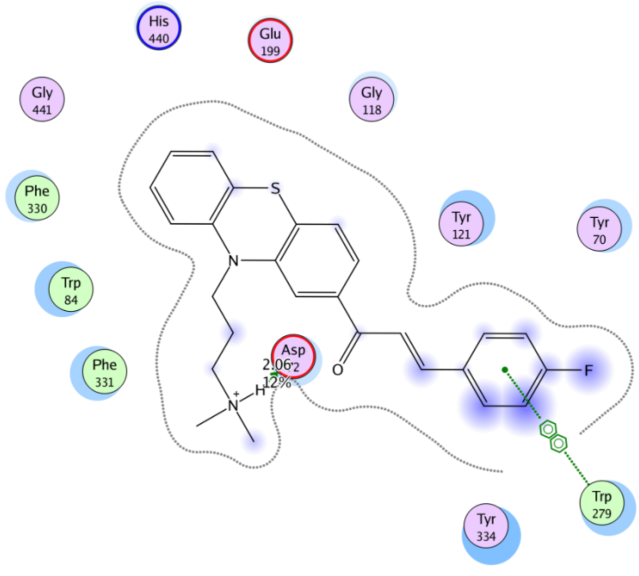 Trp279 (arene-arene), Asp72 (hydrogen bond, score: 12%, length: 2.06 Å)

AC10	4.61	4.81	-27.30	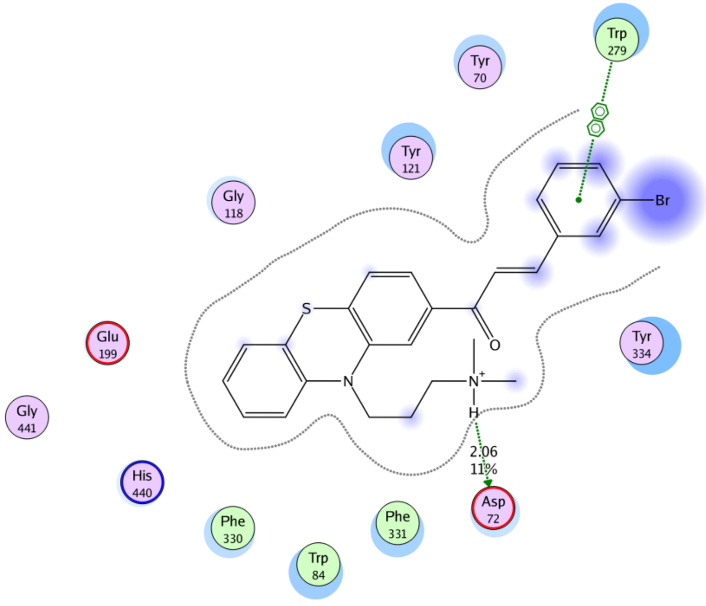Trp279 (arene-arene), Asp72 (hydrogen bond, score: 11%, length: 2.06 Å)
AC11	3.73	4.59	-25.33	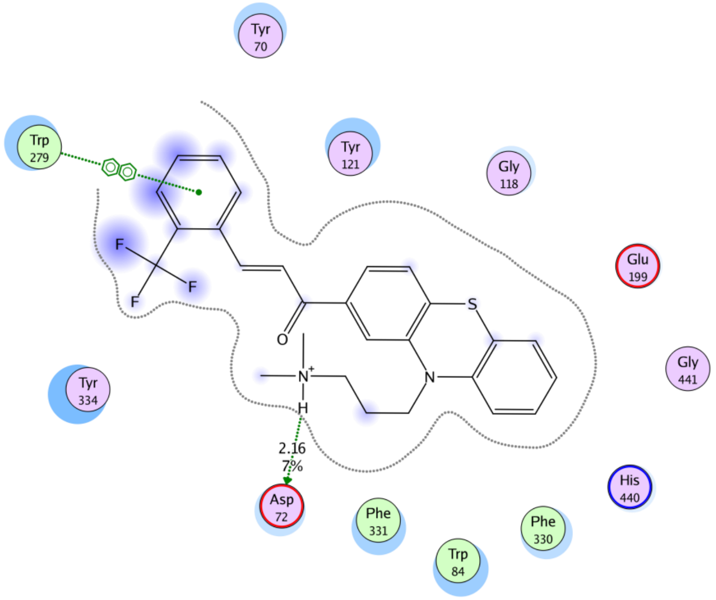Trp279 (arene-arene), Asp72 (hydrogen bond, score: 7%, length: 2.16 Å)

AC12	5.96	4.81	-22.15	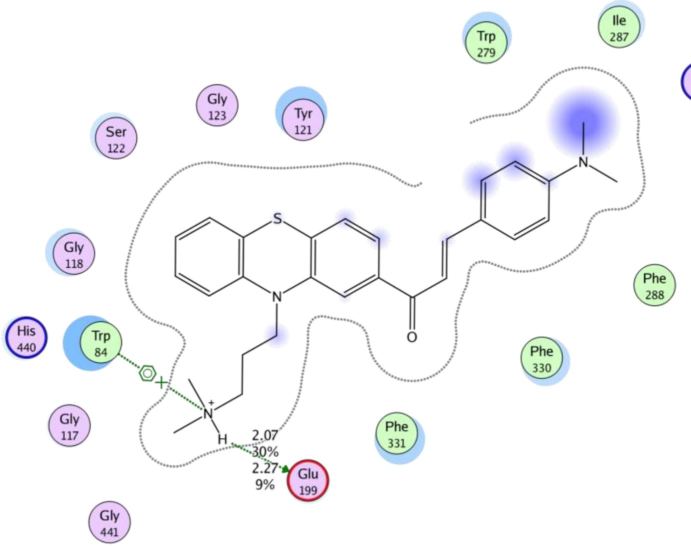<br>Trp84 (arene-cation), Glu199 (hydrogens, score: 9%; 30%; length: 2.27; 2.07 Å)
AC13	4.93	4.63	-23.30	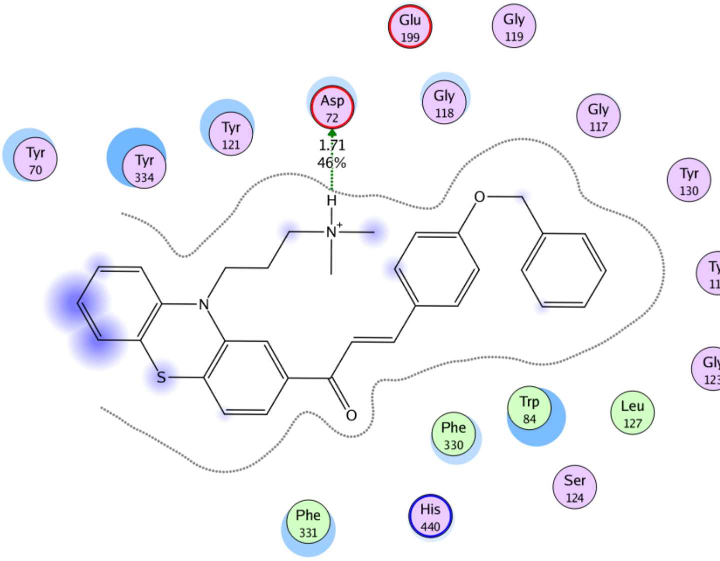<br>Asp72 (hydrogen bond, score: 46%, length: 1.71 Å)

Galanthamine	5.9	5.14	-28.53	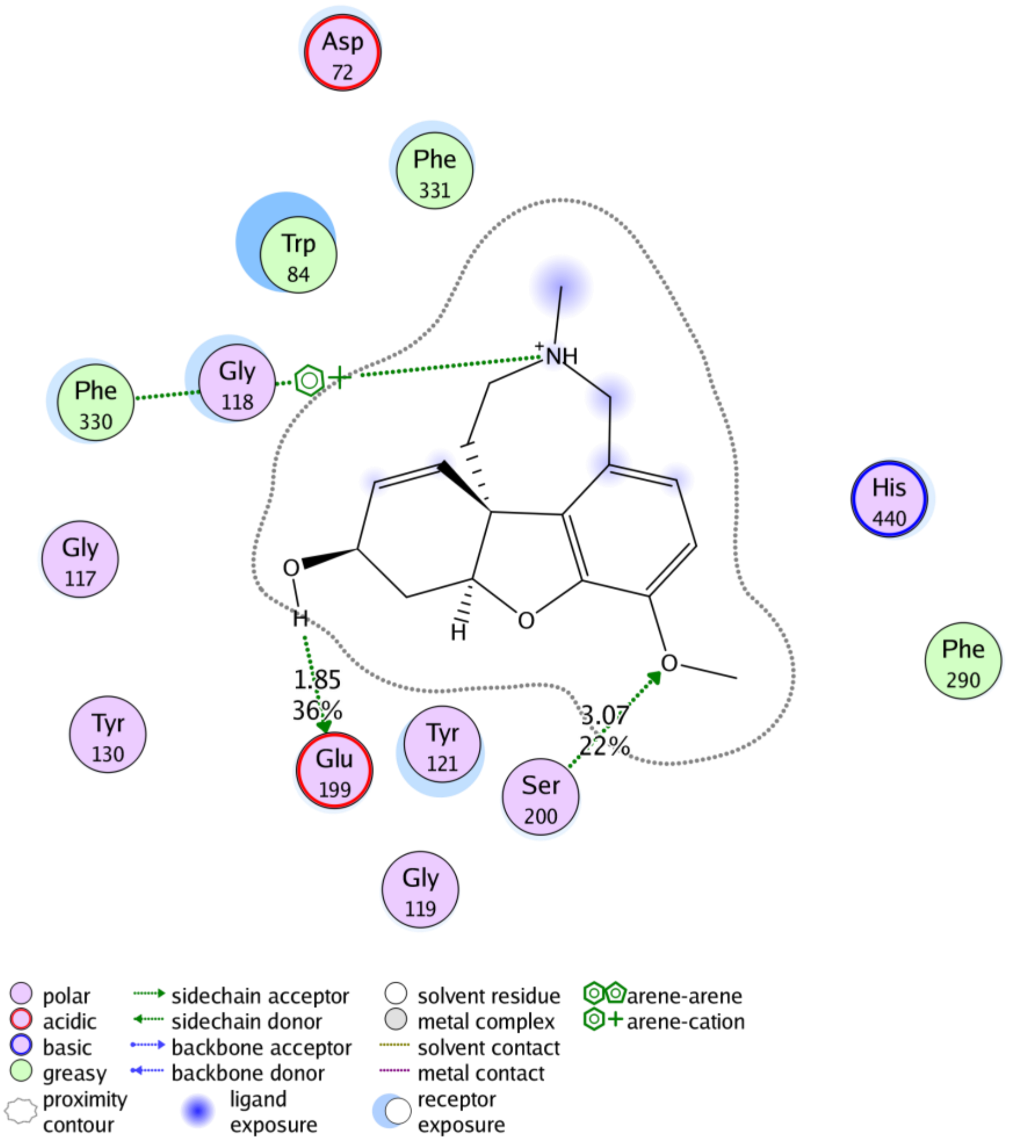 Phe330 (arene-cation), Glu199 (hydrogen, score: 36%, length: 1.85 Å), Ser200 (hydrogen, score: 22%, length: 3.07 Å)

Table S3. Docking results and ligand interaction (Co-crystallized 5HU1 chain A and B)

Compound	pIC_{50}		Chain	Docking score ($kJ.mol^{-1}$)	Interactions
	Observed	Predicted			
AC1	5.20	6.52	A	-17.82	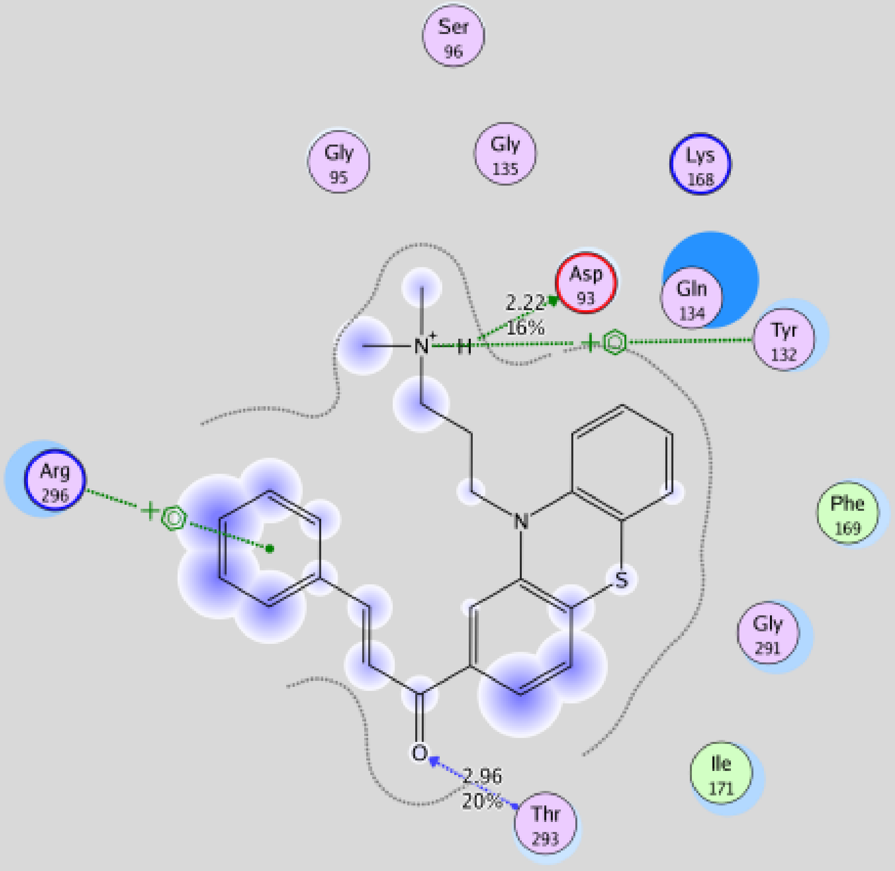 TyrA132 (arene-cation), ArgA296 (arene-cation), AspA93 (hydrogen bond, score: 16%, length 2.22 Å), ThrA293 (hydrogen bond, score: 20%, length 2.96 Å)

			B	-17.69	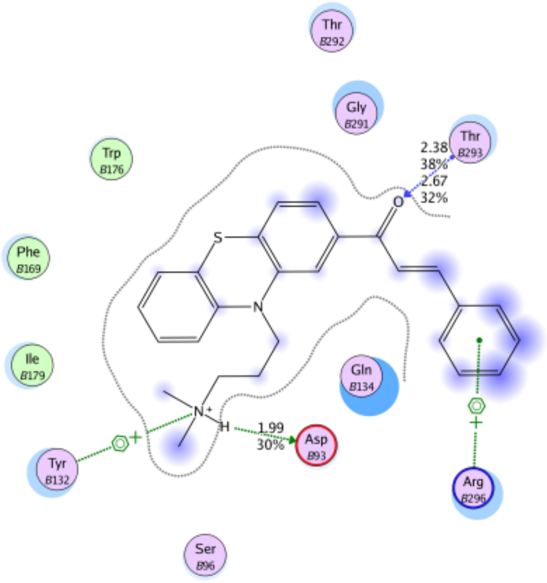<br>TyrB132 (arene-cation, ArgB296 (arene-cation), AspB93 (hydrogen bond, score: 30%, length 1.99 Å), ThrB293 (hydrogen bonds, score: 32%; 38%, length: 2.67 Å; 2.38 Å)
AC2	5.52	7.38	A	-17.22	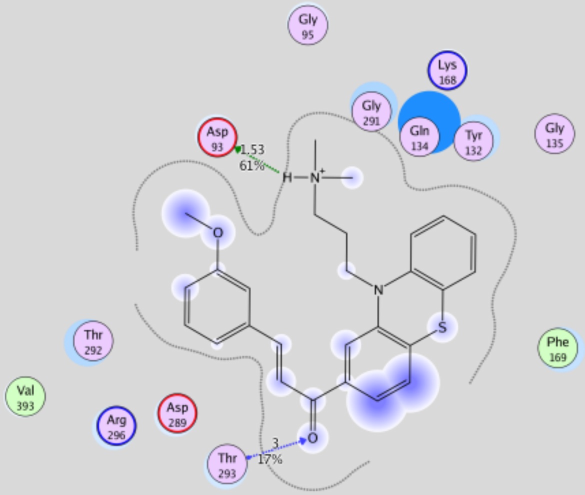<br>AspA93 (hydrogen bond, score: 61%, length 1.53 Å), ThrA293 (hydrogen bond, score: 17%, length 3 Å)

			B	-17.81	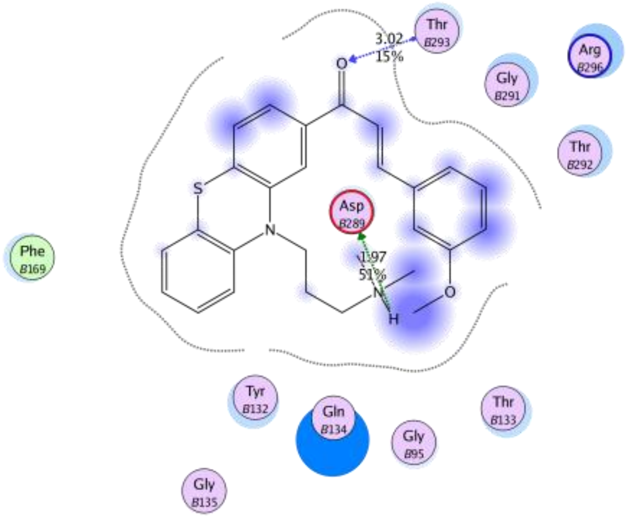<br>ThrB293 (hydrogen bond, score: 15%, length 3.02 Å), AspB289 (hydrogen bond, score: 51%, length 1.97 Å)
AC3	5.35	7.37	A	-16.28	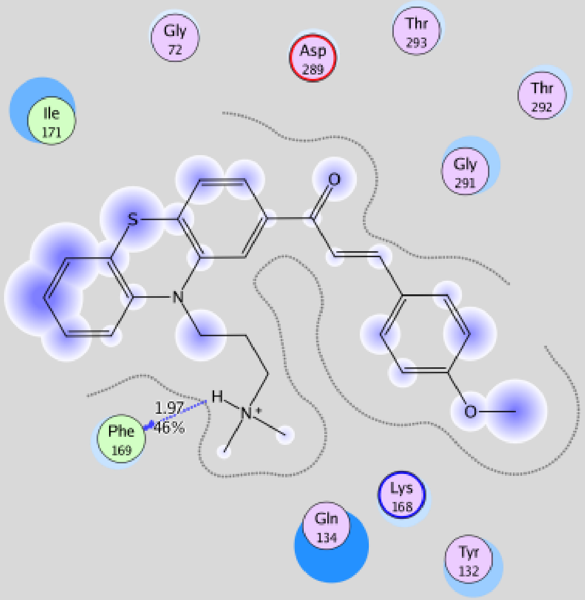<br>PheA169 (hydrogen bond, score: 46%, length 1.97 Å)

			B	-15.37	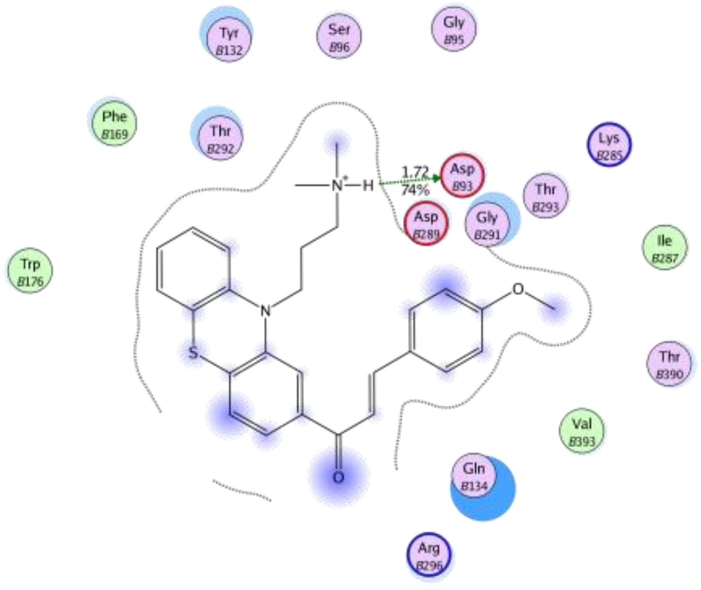 AspB93 (hydrogen bond, score: 74%, length 1.72 Å)
AC4	6.81	8.49	A	-16.71	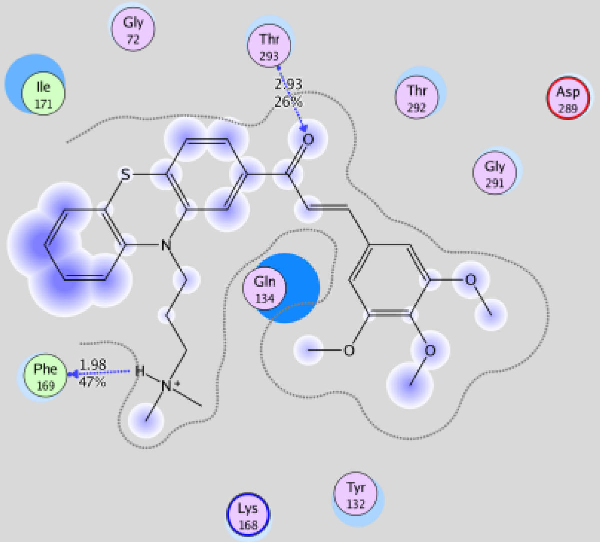 ThrA293 (hydrogen bond, score: 26%, length 2.93 Å), PheA169 (hydrogen bond, score: 47%, length 1.98 Å)

			B	-13.92	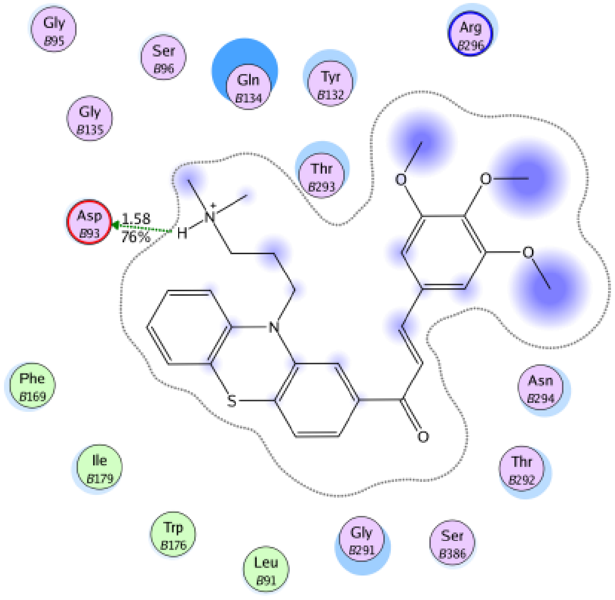 AspB93 (hydrogen bond, score: 76%, length 1.58 Å)
AC5	5.91	7.25	A	-20.77	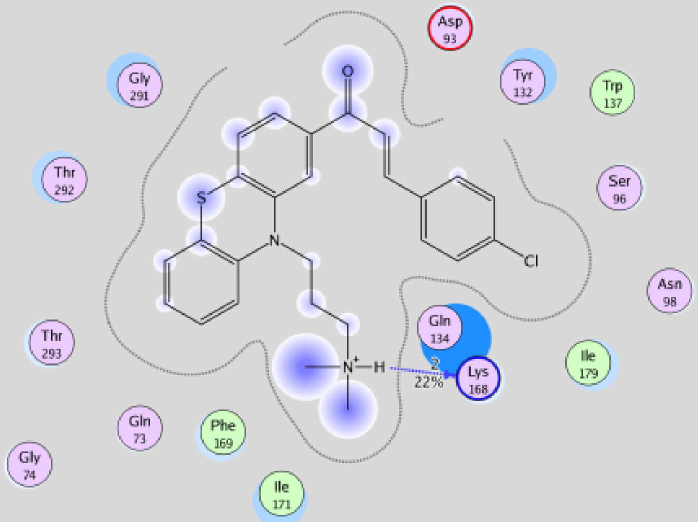 LysA168 (hydrogen bond, score: 22%, length 2 Å)

			B	-16.87	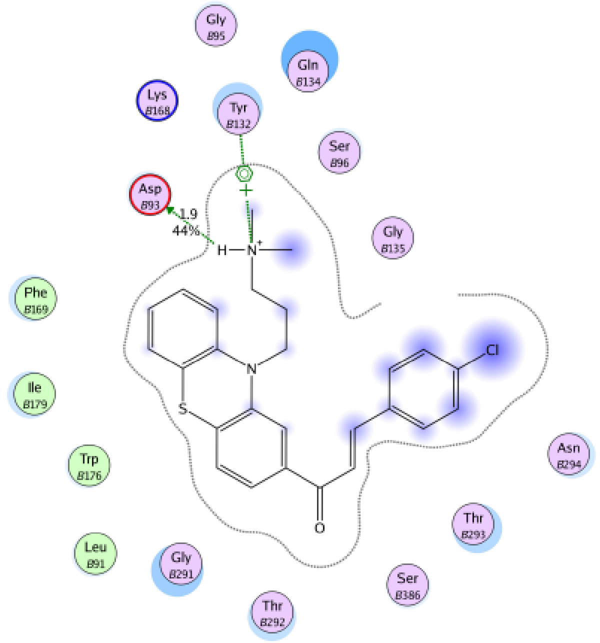<br>AspB93 (hydrogen bond, score: 44%, length 1.9 Å), TyrB132 (arene-cation)
AC6	6.30	7.68	A	-18.79	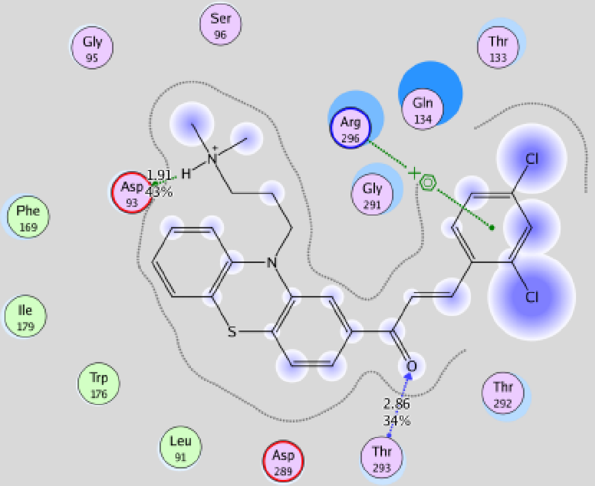<br>AspA93 (hydrogen bond, score: 43%, length 1.91 Å), ThrA293 (hydrogen bond, score: 34%, length 2.86 Å) ArgA296 (arene-cation)

			B	-16.73	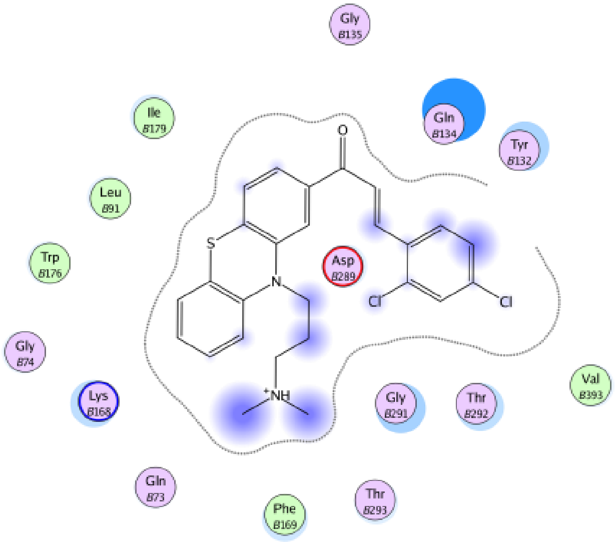<br>No strong interaction
AC7	6.35	7.63	A	-19.51	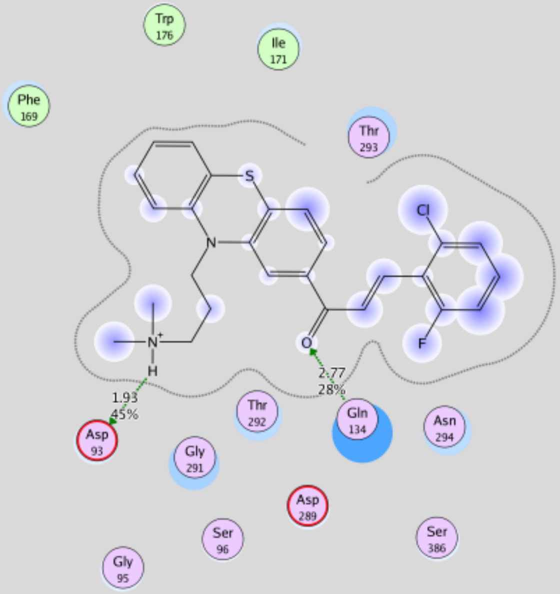<br>AspA93 (hydrogen bond, score: 45%, length 1.93 Å), GlnA134 (hydrogen bond, score: 28%, length 2.77 Å)

			B	-16.85	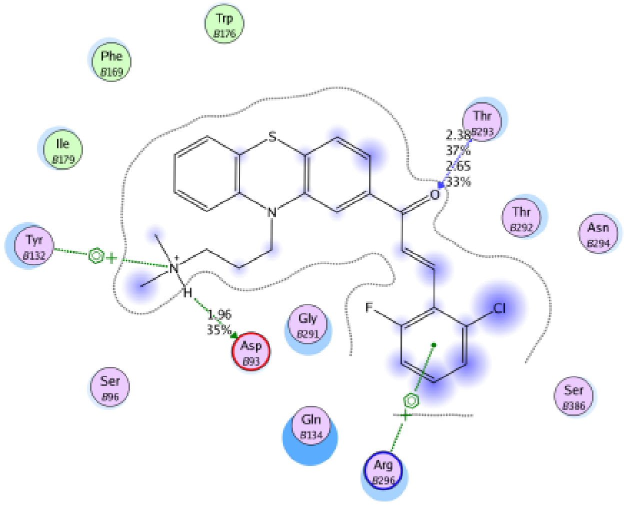<br>AspB93 (hydrogen bond, score: 35%, length 1.96 Å), ThrB293 (hydrogen bonds, score: 33%; 37%, length: 2.65 Å; 2.38 Å), TyrB132 (arene-cation), ArgB296 (arene-cation)
AC8	5.53	7.15	A	-20.36	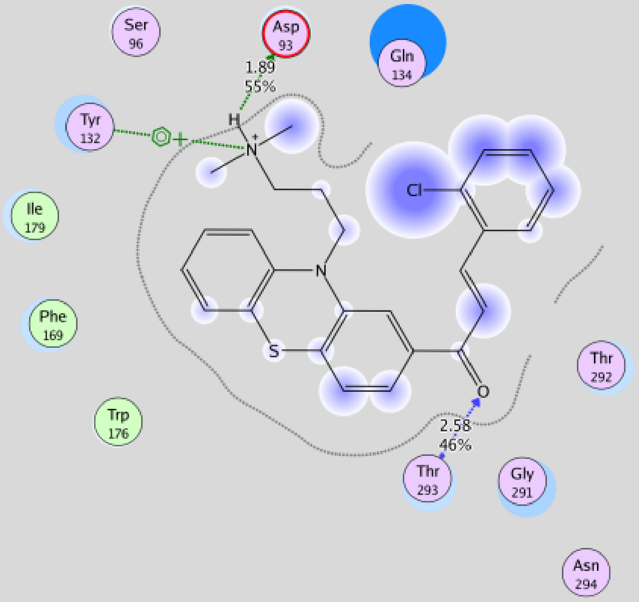<br>AspA93 (hydrogen bond, score: 55%, length 1.89 Å), ThrA293 (hydrogen bond, score: 46%, length 2.58 Å), TyrA132 (arene-cation)

			B	-16.81	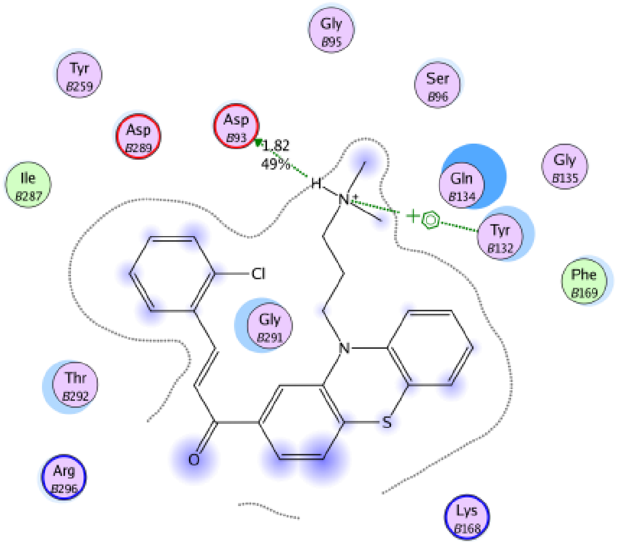<br>AspB93 (hydrogen bond, score: 49%, length 1.82 Å), TyrB132 (arene-cation)
AC9	5.43	7.12	A	-20.97	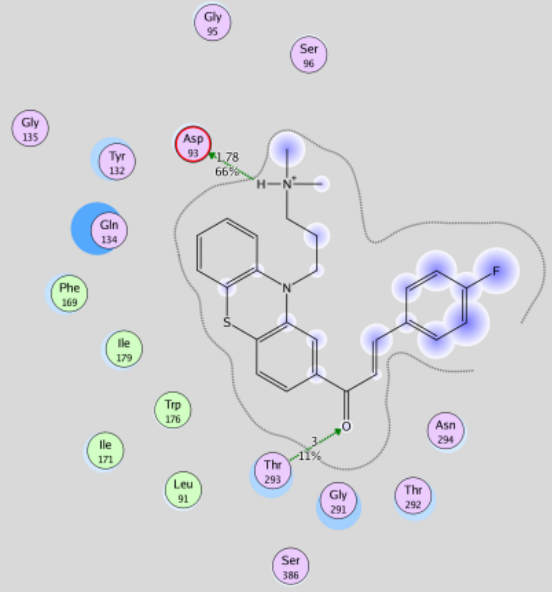<br>AspA93 (hydrogen bond, score: 66%, length 1.78 Å), ThrA293 (hydrogen bond, score: 11%, length 3 Å)

			B	-18.06	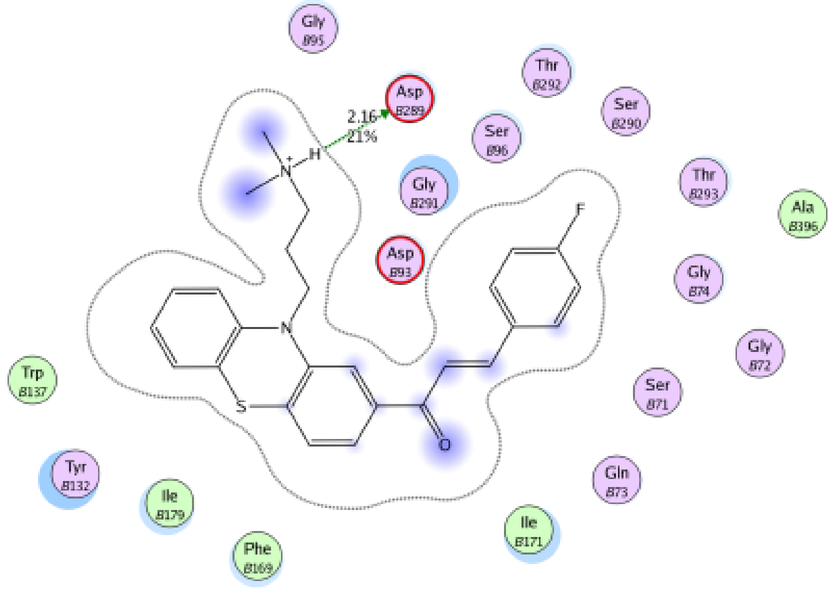 AspB289 (hydrogen bond, score: 21%, length 2.16 Å)
AC10	5.70	7.28	A	-22.51	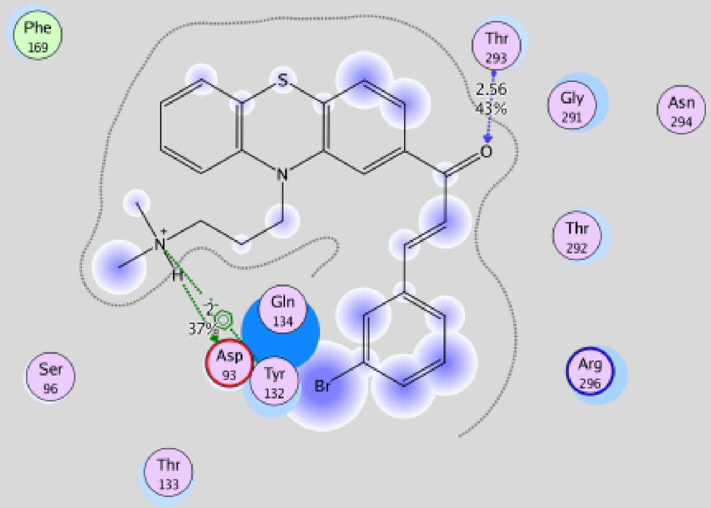 AspA93 (hydrogen bond, score: 37%, length 2 Å), ThrA293 (hydrogen bond, score: 43%, length 2.56 Å), TyrA132 (arene-cation)

			B	-20.81	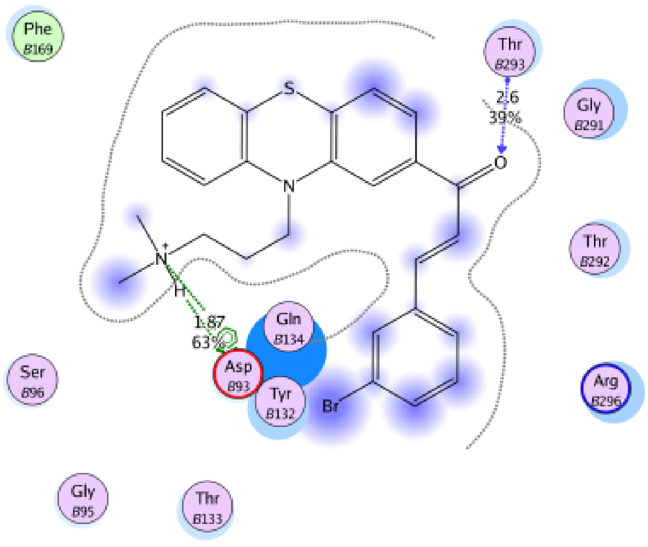 AspB93 (hydrogen bond, score: 63%, length 1.87 Å), ThrB293 (hydrogen bond, score: 39%, length 2.6 Å), TyrB132 (arene-cation)
AC11	6.40	7.99	A	-19.35	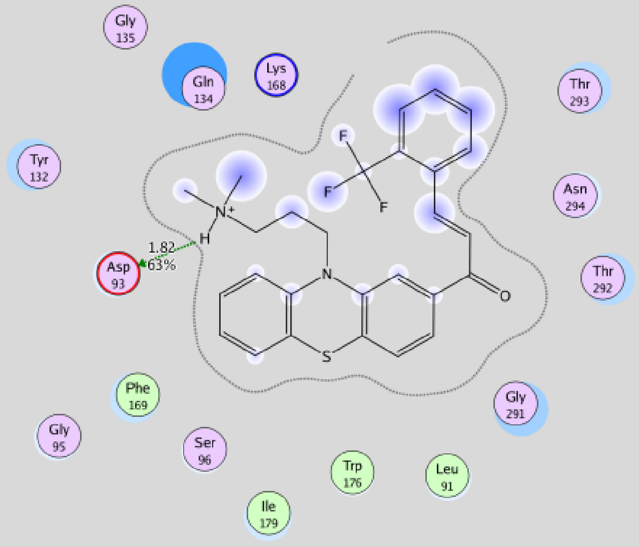 AspA93 (hydrogen bond, score: 63%, length 1.82 Å)

			B	-16.41	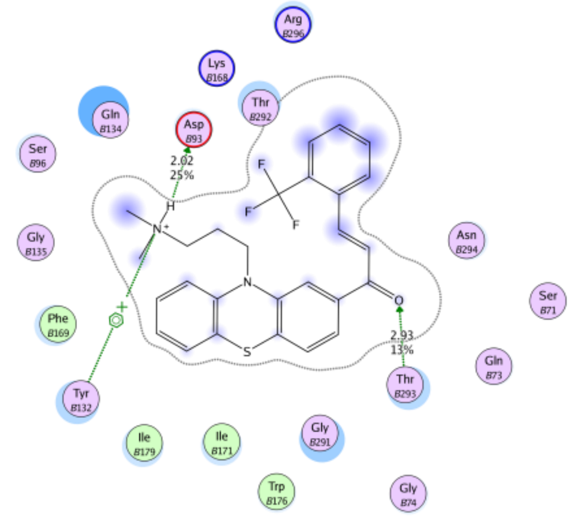 AspB93 (hydrogen bond, score: 25%, length 2.02 Å), ThrB293 (hydrogen bond, score: 13%, length 2.93 Å), TyrB132 (arene-cation)
AC12	6.46	8.08	A	-18.25	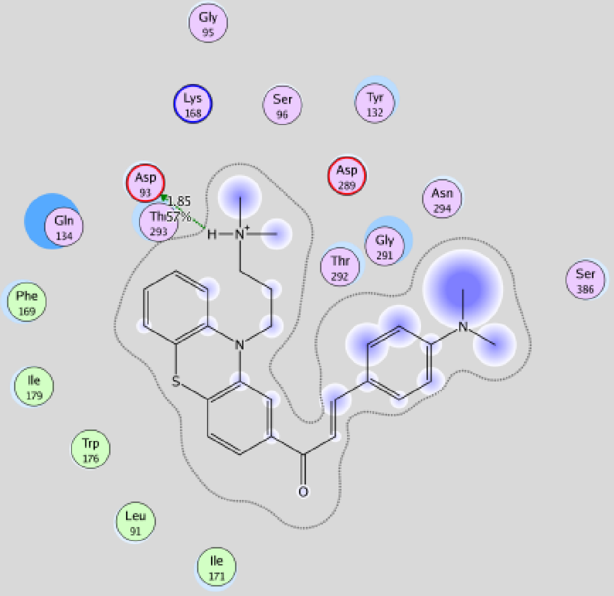 AspA93 (hydrogen bond, score: 57%, length 1.85 Å)

			B	-16.18	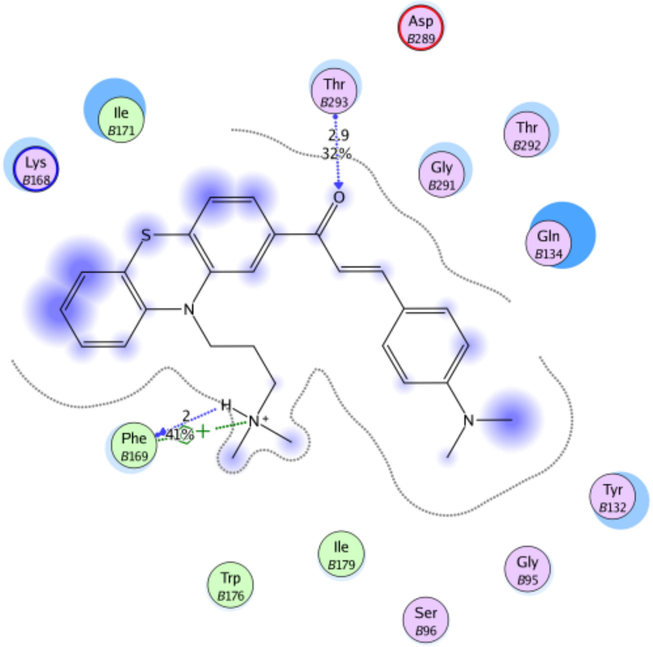<br>PheB169 (hydrogen bond, score: 41%, length 2 Å), (arene-cation), ThrB293 (hydrogen bond, score: 32%, length 2.9 Å)
AC13	5.52	7.20	A	-11.50	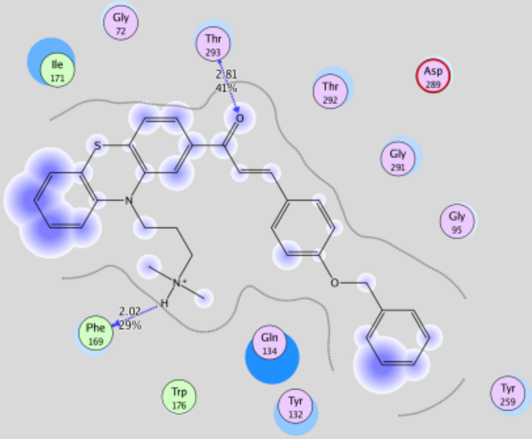<br>ThrA293 (hydrogen bond, score: 41%, length 2.81 Å), PheA169 (hydrogen bond, score: 29%, length 2.02 Å)

			B	-14.09	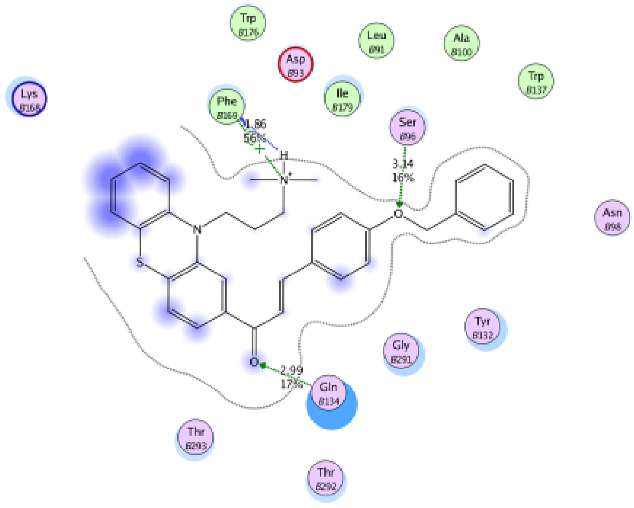 SerB96 (hydrogen bond, score: 16%, length 3.14 Å), PheB169 (hydrogen bond, score: 56%, length 1.86 Å), (arene-cation), GlnB134 (hydrogen bond, score: 17%, length: 2.99 Å)
Verubecestat	-	7.66	A		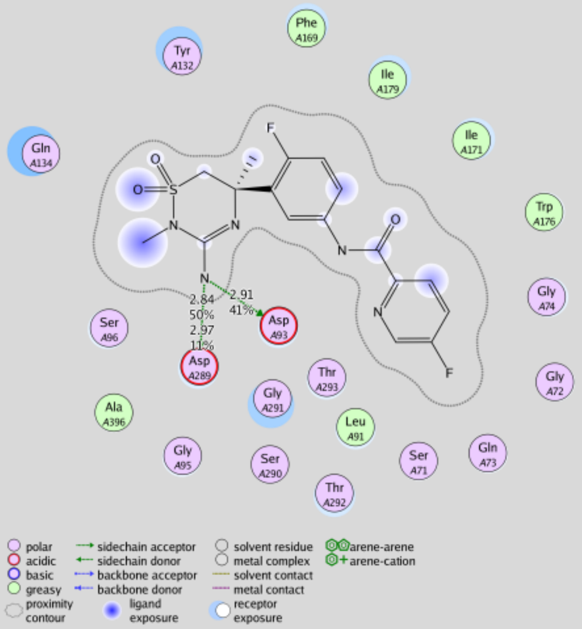 AspA93 (hydrogen bond, score: 41%, length 2.91Å), AspA289 (hydrogen bonds, score: 50%; 11%, length 2.84 Å, 2.97 Å)

			B		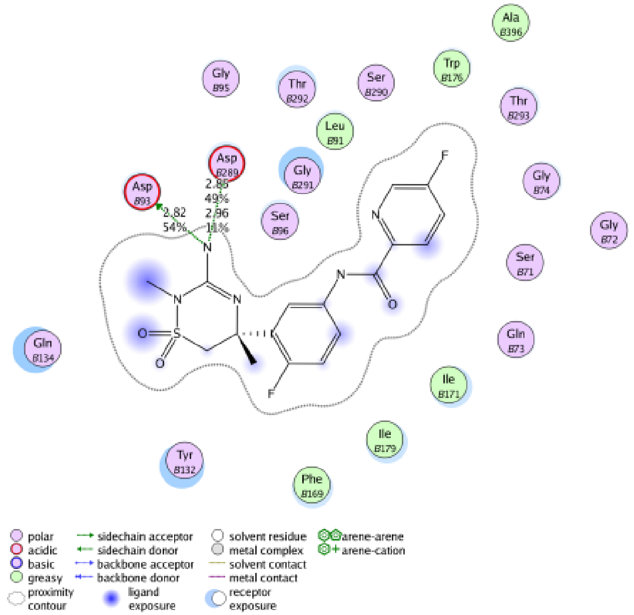 AspB93 (hydrogen bond, score: 54%, length 2.82 Å), AspB289 (hydrogen bonds, score: 49%; 11%, length 2.85 Å, 2.96 Å)
Quercetin	5.02	5.24	A	-22.23	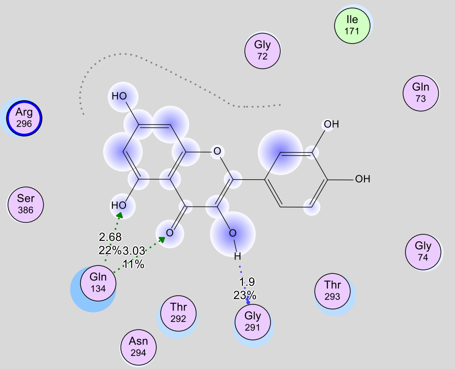 GlnA134 (hydrogen bonds, score: 22%, length: 2.68 Å; score: 11%, length 3.03 Å), GlyA291 (hydrogen bond, score: 23%, length: 1.9 Å)

			B	-23.95	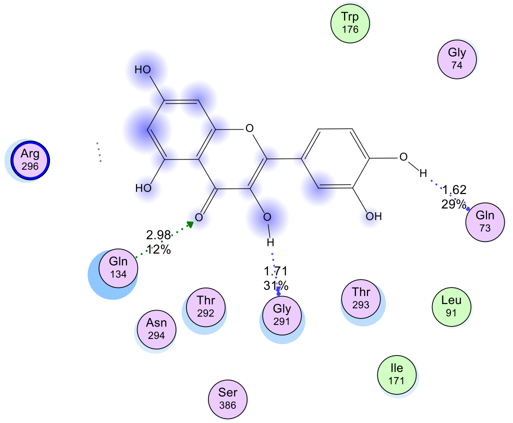 GlnB134 (hydrogen bonds, score: 12%, length: 2.98 Å; score), GlyB291 (hydrogen bond, score: 31%, length: 1.71 Å), GlnB73 (hydrogen bond, score: 29%, length: 1.62 Å)

Table S4. Dataset of 72 compounds used in the building of 2D-QSAR model for AChE inhibitors

No.	Name	pIC50	predicted pIC50
1	CHEMBL3133246	5.45	5.21
2	CHEMBL2296124	4.33	4.26
3	CHEMBL237223	4.59	4.72
4	CHEMBL2334727	4.72	4.60
5	CHEMBL3617370	4.51	4.42
6	CHEMBL3617380	4.49	4.46
7	CHEMBL2334728	4.24	4.28
8	CHEMBL416526	4.94	4.73
9	CHEMBL2393074	4.75	4.90
10	CHEMBL2385772	4.48	4.73
11	CHEMBL253386	5.49	5.22
12	CHEMBL3617382	4.63	4.52
13	CHEMBL3125443	4.90	4.67
14	CHEMBL3617375	4.28	4.49
15	CHEMBL3127216	4.84	4.70
16	CHEMBL3617367	4.38	4.27
17	CHEMBL457684	4.78	4.48
18	CHEMBL340807	4.80	4.66
19	CHEMBL2334726	4.49	4.55
20	CHEMBL659	5.62	5.42
21	CHEMBL2334730	4.64	4.78
22	CHEMBL3133247	4.81	5.07
23	CHEMBL517342	4.19	4.38
24	CHEMBL2296125	4.46	4.37
25	CHEMBL3617393	4.47	4.24
26	CHEMBL3617388	4.45	4.47
27	CHEMBL2385773	4.83	4.61
28	CHEMBL2385777	4.34	4.58
29	CHEMBL3127219	4.92	4.75
30	CHEMBL3127043	4.98	4.97
31	CHEMBL1269160	4.57	4.42
32	CHEMBL2334725	4.70	4.76
33	CHEMBL1270150	4.73	4.81
34	CHEMBL500712	4.40	4.21
35	CHEMBL2334736	4.72	4.83
36	CHEMBL2018160	4.63	4.48
37	CHEMBL2334737	4.71	4.61
38	CHEMBL269538	4.19	4.49
39	CHEMBL3617397	4.52	4.32
40	CHEMBL2334745	4.49	4.70
41	CHEMBL2296112	4.25	4.44

42	CHEMBL3617381	4.49	4.53
43	CHEMBL3133250	4.29	4.63
44	CHEMBL12014	4.23	4.50
45	CHEMBL3617372	4.26	4.44
46	CHEMBL3133243	4.97	5.10
47	CHEMBL510090	4.72	4.82
48	CHEMBL2385771	4.30	4.56
49	CHEMBL2296117	4.18	4.13
50	CHEMBL3127217	4.99	4.86
51	CHEMBL2334738	4.69	4.66
52	CHEMBL2296113	4.21	4.40
53	CHEMBL2381433	4.58	4.53
54	CHEMBL3132866	4.98	5.01
55	CHEMBL2385768	4.75	4.66
56	CHEMBL2385780	4.88	4.68
57	CHEMBL2334739	4.52	4.35
58	CHEMBL3133245	5.36	5.17
59	CHEMBL1270255	5.01	5.24
60	CHEMBL3133248	4.66	4.90
61	CHEMBL3617362	4.22	4.42
62	CHEMBL2381434	4.67	4.49
63	CHEMBL1535235	4.61	4.53
64	CHEMBL129177	4.47	4.70
65	CHEMBL2385784	4.53	4.76
66	CHEMBL2385779	4.42	4.23
67	CHEMBL3617396	4.39	4.26
68	CHEMBL1085869	5.67	5.51
69	CHEMBL2334747	4.51	4.56
70	CHEMBL2385766	4.35	4.57
71	CHEMBL463856	4.33	4.26
72	CHEMBL2334732	4.86	4.81

Table S5. Dataset of 215 compounds used in the building of 2D-QSAR model for BACE-1 inhibitors

No.	Name	pIC50	Predicted pIC50
1	BMC-2009-3671-17	6.92	7.02
2	BMC-2004-247-8	6.46	6.57
3	BMC-2007-1023-8	8.30	7.97
4	JMC-2012-27-30	5.49	5.65
5	BMC-2004-248-21	7.17	6.96
6	BMC-2009-3675-11	7.96	8.19
7	BMC-2009-3671-20	6.82	7.33
8	BMC-2009-3677-26	7.49	7.93
9	BMC-2008-1019-14	6.26	6.86
10	BMC-2014-2038-39	7.74	7.51
11	BMC-2013-4677-30	6.83	6.54
12	BMC-2004-248-19	7.37	6.74
13	BMC-2013-4677-37	5.88	6.33
14	BMC-2013-4241-10	6.46	6.45
15	BMC-2014-2036-24	7.49	8.02
16	BMC-2013-4675-7	7.35	6.70
17	BMC-2009-3667-16	7.72	7.63
18	BMC-2014-2039-43	7.28	7.39
19	EODD-2013-723-102	6.62	6.99
20	JMC-2012-26-15	5.00	5.15
21	JMC-2012-27-37	5.70	5.84
22	EODD-2013-718-42c	8.00	8.56
23	JMC-2012-9013-24	7.11	7.90
24	BMC-2014-2041-65	6.24	6.08
25	BMC-2008-1021-30	6.48	7.12
26	BMC-2009-3672-21	8.10	7.85
27	JMC-2012-9013-26	7.58	7.52
28	EODD-2013-720-53	6.82	7.20
29	BMC-2008-1021-31	6.64	6.66
30	BMC-2008-1021-28	7.08	6.58
31	JMC-2012-27-41	6.20	6.58
32	BMC-2009-3675-7	8.40	8.17
33	BMC-2009-3673-39	7.70	7.35
34	BMC-2014-2042-71	6.62	6.31
35	EODD-2013-713-11	7.55	7.25
36	BMC-2009-3672-27	7.04	7.46
37	BMC-2014-2042-90	8.07	7.85
38	EODD-2013-719-48	8.48	8.21
39	JMC-2012-27-47	6.49	6.45
40	BMC_2014-2042-81	7.60	8.12
41	BMC-2009-3665-4	8.04	7.43
42	BMC-2009-3673-36	7.64	7.69

43	BMC-2013-4676-17	6.93	7.33
44	BMC-2014-2038-41	7.70	7.60
45	BMC-2013-4677-38	6.07	6.44
46	BMC-2009-3667-20	7.47	7.71
47	EODD-2013-720-58	7.70	7.39
48	JMC-2012-26-24b	6.20	5.74
49	BMC-2014-2036-23	7.70	7.56
50	BMC-2013-4677-53	7.05	7.14
51	BMC-2007-1023-2	6.60	7.23
52	BMC-2009-3667-17	7.64	7.27
53	BMC-2008-1020-23	6.22	6.37
54	BMC-2014-2041-66	6.15	6.14
55	BMC-2013-4241-4	5.99	6.39
56	BMC-2009-3673-34	7.85	7.19
57	BMC-2014-2036-17	7.32	7.35
58	BMC-2008-1020-18	6.12	5.95
59	BMC-2014-2038-37	4.66	5.00
60	BMC-2009-3672-24	8.30	7.96
61	BMC-2007-1024-18	7.40	7.93
62	BMC-2008-1020-24	6.80	6.66
63	JMC-2012-28-64	6.10	6.08
64	BMC-2014-2035-13	8.00	7.51
65	BMC-2014-2040-53	8.15	8.02
66	BMC-2009-3667-18a	7.58	7.41
67	BMC-2009-3666-10	7.64	8.03
68	JMC-2012-9010-17	8.10	7.96
69	BMC-2014-2036-20	7.11	7.79
70	EODD-2013-721-64	6.33	6.65
71	BMC-2013-4677-35	6.38	6.60
72	BMC-2009-3671-12	7.04	7.62
73	EODD-2013-718-44	8.70	8.08
74	EODD-2013-721-65	7.20	6.65
75	BMC-2013-4242-23	7.34	7.00
76	BMC-2013-4241-9	7.92	7.22
77	BMC-2014-2040-62	7.69	7.61
78	BMC-2007-1023-5	7.30	7.87
79	BMC-2004-247-9	6.26	6.30
80	EODD-2013-718-42b	8.04	8.33
81	BMC-2009-3676-18	8.10	7.76
82	BMC-2007-1023-6	8.22	8.01
83	BMC-2009-3668-25	8.22	7.78
84	EODD-2013-718-45	7.68	7.34
85	BMC-2004-246-4	6.72	6.46
86	BMC-2013-4241-14	7.51	7.15
87	BMC-2009-3667-19	7.55	7.59

88	BMC-2007-1025-22	8.22	7.68
89	EODD-2013-716-31	8.70	8.27
90	BMC-2014-2042-85	6.53	7.06
91	BMC-2007-1023-11	8.30	8.05
92	BMC-2009-3671-16	7.58	8.09
93	BMC-2014-2039-42	7.08	7.22
94	BMC-2009-3666-11	7.74	8.32
95	BMC-2014-2038-40	7.70	7.27
96	BMC-2004-248-13	6.68	7.04
97	BMC-2014-2039-47	7.57	7.82
98	BMC-2009-3672-25	7.22	7.43
99	BMC-2009-3677-23	7.30	7.25
100	BMC-2009-3666-7	7.20	7.33
101	JMC-2012-28-83	5.10	5.52
102	BMC-2014-2035-14	7.70	8.20
103	BMC-2007-1023-10	8.30	8.19
104	BMC-2014-2035-15	8.00	8.16
105	BMC-2014-2036-22	7.04	6.54
106	EODD-2013-718-42a	7.89	8.20
107	BMC-2014-2042-72	7.58	7.34
108	BMC-2013-4242-24	6.43	6.98
109	BMC-2009-3672-26	8.04	7.58
110	BMC-2009-3676-21	6.60	6.71
111	BMC-2009-3677-24	7.40	7.21
112	BMC-2013-4242-20	6.92	6.62
113	BMC-2009-3675-9	8.00	7.47
114	BMC-2013-4241-5	6.26	6.22
115	JMC-2012-26-19b	5.20	5.44
116	BMC-2014-2042-87	7.31	7.24
117	BMC-2014-2042-84	6.49	7.03
118	BMC-2013-4676-19	7.38	7.76
119	BMC-2013-4241-3	5.05	5.61
120	BMC-2009-3668-27	7.40	7.26
121	BMC-2009-3677-25	7.70	7.26
122	BMC-2009-3675-10	7.66	7.50
123	BMC-2007-1025-21	8.40	7.77
124	BMC-2007-1024-17	8.30	8.17
125	BMC-2004-246-5	6.89	6.25
126	BMC-2009-3676-19	7.06	7.16
127	BMC-2013-4675-4	6.24	6.39
128	BMC-2014-2041-64	6.92	6.23
129	BMC-2004-246-6	5.95	6.41
130	BMC-2013-4242-22	7.64	7.14
131	BMC-2007-1024-15	7.92	7.44
132	BMC-2013-4241-12	6.86	6.99

133	BMC-2009-3673-35	8.30	7.86
134	BMC-2008-1019-13	6.52	6.58
135	BMC-2013-4241-16	6.12	6.63
136	BMC-2014-2042-73	7.52	7.45
137	JMC-2012-27-34	5.70	5.91
138	BMC-2013-4677-36	6.65	6.43
139	BMC-2004-248-20	6.75	6.64
140	BMC-2009-3672-23	7.64	7.81
141	JMC-2012-9010-18	7.64	7.67
142	BMC-2009-3675-13	7.64	7.55
143	JMC-2012-28-65	6.30	6.03
144	BMC-2013-4241-15	7.35	7.51
145	BMC-2004-246-7	5.76	6.23
146	BMC-2013-4677-48	8.04	7.86
147	BMC-2013-4241-13	7.41	7.34
148	BMC-2013-4675-6	6.42	6.42
149	BMC-2009-3671-9	8.70	8.29
150	BMC-2009-3671-11	7.72	8.26
151	BMC-2008-1019-12	7.48	6.96
152	BMC-2008-1021-27	7.70	7.30
153	BMC-2014-2039-46	7.27	7.16
154	BMC-2014-2035-9	7.10	6.33
155	JMC-2012-9010-15	7.74	7.98
156	BMC-2007-1024-13	7.47	7.41
157	BMC-2014-2042-79	7.40	7.59
158	EODD-2013-716-32	8.40	8.23
159	BMC-2008-1021-29	7.00	7.42
160	JMC-2012-9013-25	7.48	7.95
161	BMC-2004-246-3	7.09	7.26
162	BMC-2014-2042-80	7.47	7.90
163	BMC-2004-247-10	6.46	6.20
164	JMC-2012-9013-23	7.54	8.48
165	BMC-2008-1020-26	7.85	7.24
166	BMC-2009-3667-18b	7.40	7.51
167	EODD-2013-713-12a	8.41	8.84
168	BMC-2009-3677-27	7.10	7.58
169	BMC-2004-248-14	6.68	6.49
170	BMC-2007-1024-12	6.92	6.75
171	EODD-2013-718-41	7.30	7.95
172	BMC-2009-3675-14	7.32	7.60
173	BMC-2008-1019-8	5.61	6.20
174	BMC-2008-1020-22	5.77	6.13
175	BMC-2008-1019-6	6.74	6.26
176	BMC-2009-3671-15	8.15	8.22
177	BMC-2013-4242-19	7.21	6.91

178	BMC-2014-2042-82	7.55	7.36
179	EODD-2013-721-61	6.96	7.39
180	BMC-2008-1020-16	7.40	7.28
181	BMC-2014-2042-76	8.70	8.94
182	BMC-2009-3675-5	8.30	7.93
183	BMC-2009-3675-16	7.70	7.51
184	BMC-2007-1023-9	7.96	8.00
185	BMC-2014-2042-78	7.70	7.43
186	BMC-2008-1019-7	5.95	6.35
187	BMC-2013-4675-3	6.27	6.20
188	JMC-2012-26-8b	4.60	4.57
189	EODD-2013-713-10	7.06	7.07
190	JMC-2012-26-19a	5.00	5.44
191	BMC-2014-2035-12	5.47	5.08
192	BMC-2013-4676-18	6.90	7.35
193	JMC-2012-9013-19	8.27	8.63
194	BMC-2004-248-17	7.22	6.58
195	BMC-2014-2040-54	8.22	8.06
196	BMC-2009-3671-13	7.82	7.98
197	JMC-2012-26-24a	6.00	6.04
198	BMC-2008-1020-19	7.77	7.61
199	EODD-2013-719-50	7.77	8.60
200	JMC-2012-27-54a	5.40	5.90
201	EODD-2013-717-39a	9.40	9.53
202	BMC-2007-1024-14	7.74	8.09
203	BMC-2013-4677-31	6.73	6.68
204	BMC-2009-3672-22	8.70	8.02
205	BMC-2009-3667-23	6.38	6.32
206	BMC-2013-4675-5	7.03	6.31
207	BMC-2013-4677-32	7.22	7.24
208	BMC-2014-2042-88	7.44	7.03
209	BMC-2007-1023-7	8.52	7.97
210	JMC-2012-26-8a	4.79	4.94
211	JMC-2012-9013-22	7.69	8.31
212	BMC-2013-4241-18	7.02	6.58
213	BMC-2014-2042-75	8.00	8.54
214	JMC-2012-9010-16	7.45	8.05
215	BMC-2007-1024-16	8.10	7.53

Table S6. Equations for calculation of 2D-QSAR validation metrics

These metrics were calculated according to the equations (Eq.) (1-10).

$$\text{RMSE} = \sqrt{\frac{\sum_{i=1}^{n_{TR}}(\hat{y}_i - y_i)^2}{n}} \quad (1)$$

$$\text{R}^2 = 1 - \frac{\sum_{i=1}^{n_{TR}}(\hat{y}_i - y_i)^2}{\sum_{i=1}^{n_{TR}}(y_i - \bar{y}_i)^2} \quad (2)$$

$$\text{RMSE}_{\text{LOO}} = \sqrt{\frac{\sum_{i=1}^{n_{TR}}(\hat{y}'_i - y_i)^2}{n}} \quad (3)$$

$$\text{R}^2_{\text{LOO}} = 1 - \frac{\sum_{i=1}^{n_{TR}}(\hat{y}'_i - y_i)^2}{\sum_{i=1}^{n_{TR}}(y_i - \bar{y}_i)^2} \quad (4)$$

$$\text{R}^2_{\text{PRED}} = 1 - \frac{\sum_{I=1}^{n_{VAL}}\left(y_{pred(validation)} - y_{validation}\right)^2}{\sum_{I=1}^{n_{VAL}}\left(y_{validation} - \bar{y}_{training}\right)^2} \quad (5)$$

$$r_m^2 = r^2(1 - \sqrt{r^2 - r_0^2} \quad (6)$$

$$r'^2_m = r'^2\,(1 - \sqrt{r'^2 - r'^2_0}) \quad (7)$$

$$\overline{r_m^2} = \frac{r_m^2 + r'^2_m}{2} \quad (8)$$

$$\Delta r_m^2 = |r_m^2 - r'^2_m| \quad (9)$$

$$CCC = \frac{2\sum_{i=1}^{n_{VAL}}(y_i - \bar{y})(\hat{y}_i - \bar{\hat{y}})}{\sum_{i=1}^{n_{VAL}}(y_i - \bar{y})^2 + \sum_{i=1}^{n_{VAL}}(\hat{y}_i - \bar{\hat{y}})^2 + n_{VAL}(\bar{y} - \bar{\hat{y}})^2} \quad (10)$$

In Eq. (1), (2), and (10), y_i and $\hat{y}_i$ are, respectively, the observed and predicted activity values, while $\bar{y}$ and $\bar{\hat{y}}$ are, respectively, the mean values of y_i and $\hat{y}_i$. In Eq.(3) and (4), y_i and $\hat{y}'_i$ are, respectively, the observed and predicted activity values in LOO cross-validation. Eq. (6) and (7) were utilized to calculate correlation coefficients between observed and predicted activity values of the compounds of training set with (r^2) or without intercept (r_0^2) in case of using predicted data on the y-axis and experimental data on the x-axis, while (r'^2) and (r'^2_0) are, respectively, the same coefficients in the opposite case. The most stringent validation criteria thresholds including $\text{r}_\text{m}^2 \geq 0.65$; CCC ≥ 0.85; $\overline{\text{r}_\text{m}^2} \geq 0.5$; and $\Delta\text{r}_\text{m}^2 \leq 0.2$ were applied to verify the external predictivity of good models [1,2]

Table S7. Selected descriptors used for building 2D-QSAR models

Code	Category	Description
BCUT_SlogP_3	Adjacency and distance matrix	A Burden's parameter using atomic contribution to logP (using the Wildman and Crippen SlogP method [3]) instead of partial charge.
BCUT_PEOE_1	Adjacency and distance matrix	A descriptor relating topological shape and partial charges
petitjean	Adjacency and distance matrix	Value of (diameter - radius) / diameter.
reactive	Physical property	An indicator of the presence of reactive groups. A non-zero value indicates that the molecule contains a reactive group. The table of reactive groups is based on the Oprea set [4] and includes metals, phospho-, N/O/S-N/O/S single bonds, thiols, acyl halides, Michael Acceptors, azides, esters, etc.
logS	Physical property	The log of the aqueous solubility (mol/L).
PEOE_VSA-0, PEOE_VSA+1, PEOE_VSA-3, PEOE_VSA-6	Partial charge	Sum of the proximate accessible *van der Waals* surface area (Å^2), v_i, calculation for each atom over all the atoms i, such that partial charge of atom i is in a specified range.
SlogP_VSA2, SlogP_VSA3, SlogP_VSA5	Subdivided surface areas	Sum of the proximate accessible *van der Waals* surface area (Å^2), v_i, calculated for each atom over all the atoms, such that partition coefficient for atom i is in a specified range
SMR_VSA2	Subdivided surface areas	Sum of the proximate accessible *van der Waals* surface area (Å^2), v_i, calculation for each atom over all the atoms i, such that molar refractivity for atom i is in a specified range.
a_ICM	Atom counts and bond counts	The entropy of the element distribution in the molecule (including implicit hydrogens but not lone pair pseudo-atoms)
chiral_u	Atom counts and bond counts	The number of unconstrained chiral centers.
rings	Atom counts and bond counts	The number of rings
a_Nn	Atom counts and bond counts	The number of nitrogen atoms.

Table S8. Values of selected descriptors used in prediction of pIC_{50} of the synthesized chalcone derivatives (AChE)

Compound	BCUT_SLOGP_3	reactive	PEOE_VSA+1	PEOE_VSA-3	SlogP_VSA2	SMR_VSA2	Predicted pIC_{50}
AC1	2.61	1	59.72	0.00	0.00	6.23	**4.80**
AC2	2.61	1	76.70	0.00	0.00	6.23	**4.74**
AC3	2.61	1	76.70	0.00	0.00	6.23	**4.74**
AC4	2.61	1	85.31	0.00	0.00	6.23	**4.71**
AC5	2.61	1	55.45	0.00	0.00	6.23	**4.82**
AC6	2.61	1	51.18	0.00	0.00	6.23	**4.83**
AC7	2.61	1	55.70	0.00	9.12	15.35	**4.76**
AC8	2.61	1	55.45	0.00	0.00	6.23	**4.82**
AC9	2.61	1	55.45	0.00	9.12	15.35	**4.76**
AC10	2.61	1	55.45	0.00	0.00	9.28	**4.81**
AC11	2.61	1	57.66	35.73	0.00	13.99	**4.59**
AC12	2.61	1	55.45	0.00	0.00	9.35	**4.81**
AC13	2.61	1	108.72	0.00	0.00	6.23	**4.63**
Galantamine	2.77	0	114.49	0.00	12.94	0.00	**5.14**

Table S9. Values of selected descriptors used in prediction of pIC_{50} of the synthesized chalcone derivatives (BACE-1)

Compound	petitjean	BCUT_PEOE_1	a_ICM	chiral_u	rings	a_nN	PEOE_VSA-0	PEOE_VSA-6	logS	SlogP_VSA3	SlogP_VSA5	Predicted pIC_{50}
AC1	0.47	-0.63	1.41	0	4	2	144.11	0.00	-6.42	36.88	68.90	**6.52**
AC2	0.50	-0.61	1.46	0	4	2	168.62	2.50	-6.47	36.88	104.28	**7.38**
AC3	0.47	-0.61	1.46	0	4	2	168.62	2.50	-6.47	36.88	104.28	**7.37**
AC4	0.47	-0.59	1.52	0	4	2	168.62	7.51	-6.57	36.88	175.05	**8.49**
AC5	0.50	-0.63	1.52	0	4	2	168.62	0.00	-7.15	36.88	68.90	**7.25**
AC6	0.50	-0.64	1.59	0	4	2	180.87	0.00	-7.89	36.88	68.90	**7.68**
AC7	0.47	-0.64	1.62	0	4	2	178.67	0.00	-7.45	36.88	68.90	**7.63**
AC8	0.47	-0.64	1.52	0	4	2	168.62	0.00	-7.15	36.88	68.90	**7.15**
AC9	0.50	-0.63	1.52	0	4	2	168.62	0.00	-6.71	36.88	68.90	**7.12**
AC10	0.47	-0.63	1.52	0	4	2	168.62	0.00	-7.51	36.88	71.95	**7.28**
AC11	0.47	-0.56	1.62	0	4	2	156.36	0.00	-7.48	36.88	68.90	**7.99**
AC12	0.47	-0.58	1.42	0	4	3	215.10	0.00	-6.35	36.88	134.69	**8.08**
AC13	0.48	-0.64	1.41	0	5	2	170.83	2.50	-8.24	36.88	68.90	**7.20**
Verubecestat	0.50	-0.58	2.00	1	3	5	112.04	12.47	-3.62	17.23	49.68	**7.66**
Quercetin	0.50	-0.63	-1.52	0	3	0	61.27	41.34	-2.77	25.39	5.24	**5.24**

Table S10. Spectra of synthesized chalcone derivatives

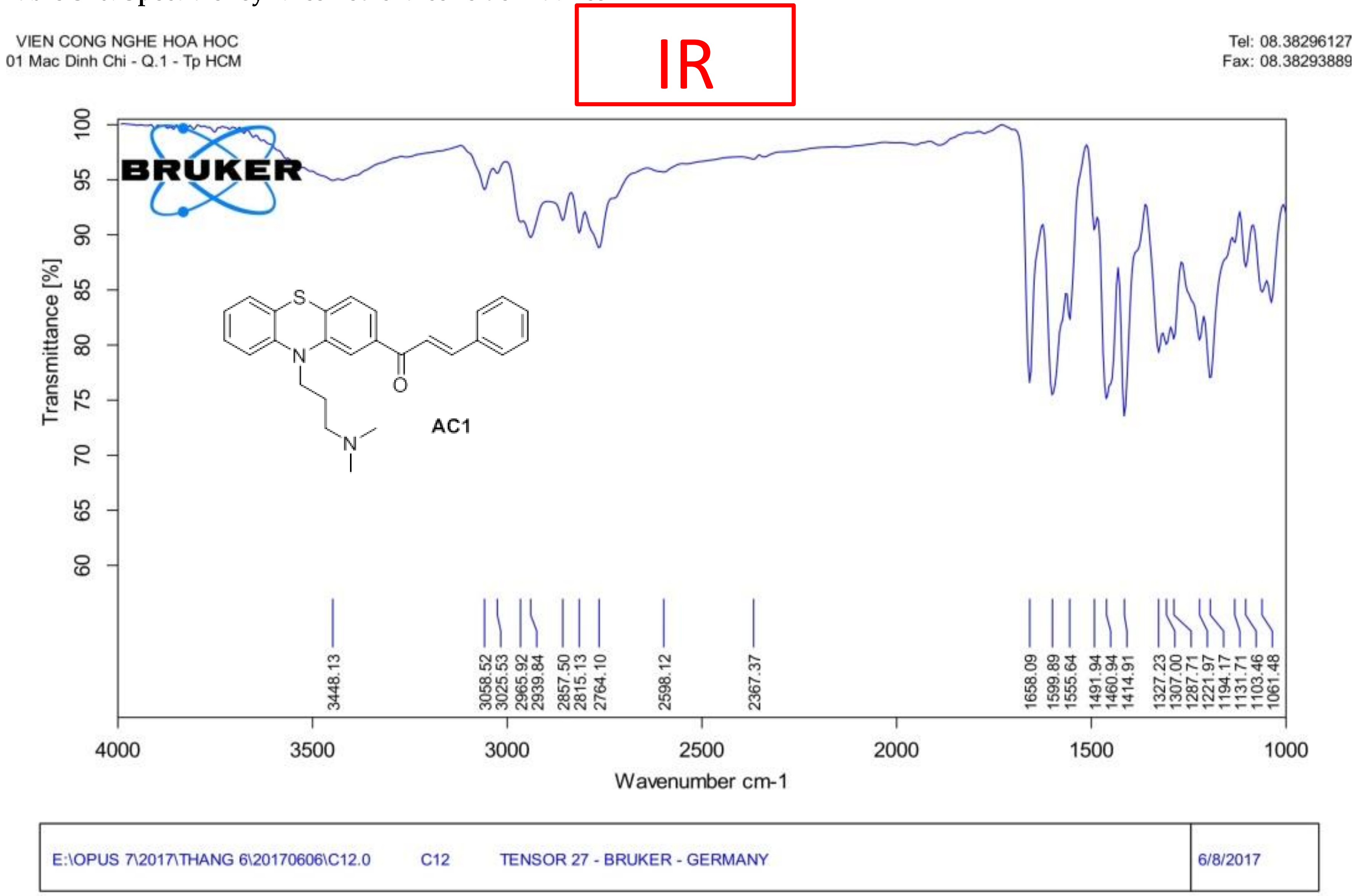

MS

Qualitative Analysis Report

**Data Filename**	01-AC1.d	**Sample Name**	01-AC1
**Sample Type**	Sample	**Position**	P2-A9
**Instrument Name**	Instrument 1	**User Name**	
**Acq Method**	Cot ngan - MSMS_Pos.m	**Acquired Time**	22/08/2020 10:06:30 AM
**IRM Calibration Status**	Success	**DA Method**	COTNGAN.M.m
**Comment**			

**Sample Group**		**Info.**	
**Stream Name**	LC 1	**Acquisition SW Version**	6200 series TOF/6500 series Q-TOF B.06.01 (B6172 SP1)

User Chromatograms

Fragmentor Voltage 140 **Collision Energy** 0 **Ionization Mode** ESI

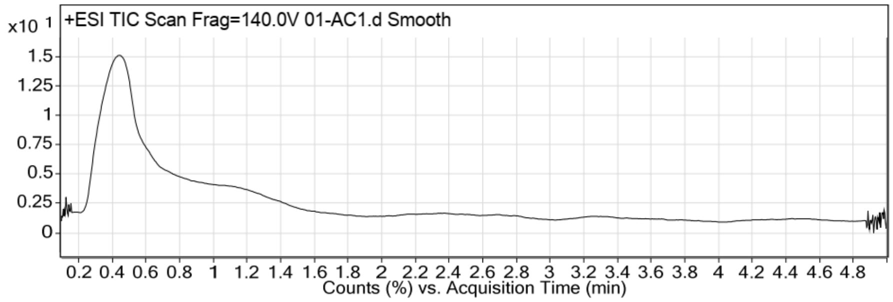

User Spectra

Fragmentor Voltage	Collision Energy	Ionization Mode
140	0	ESI

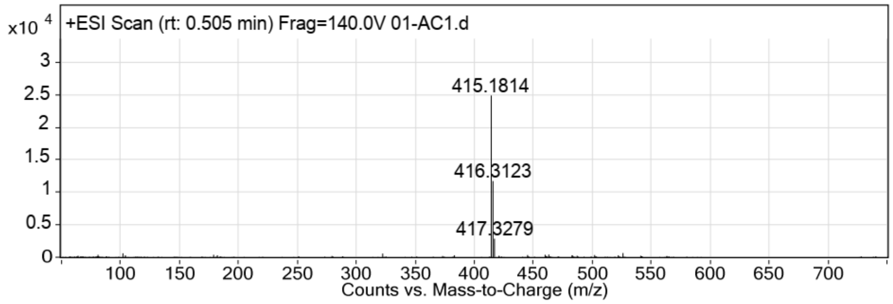

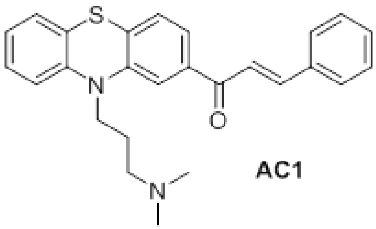

AC1

--- End Of Report ---

MS

**Sample Name**	01-AC1	**Position**	P2-A9	**Instrument Name**	Instrument 1	**User Name**	
**Inj Vol**	2	**InjPosition**		**SampleType**	Sample	**IRM Calibration Status**	Success
**Data Filename**	01-AC1.d	**ACQ Method**	Cot ngan - MSMS_Pos.	**Comment**		**Acquired Time**	22/08/2020 10:06:30 AM

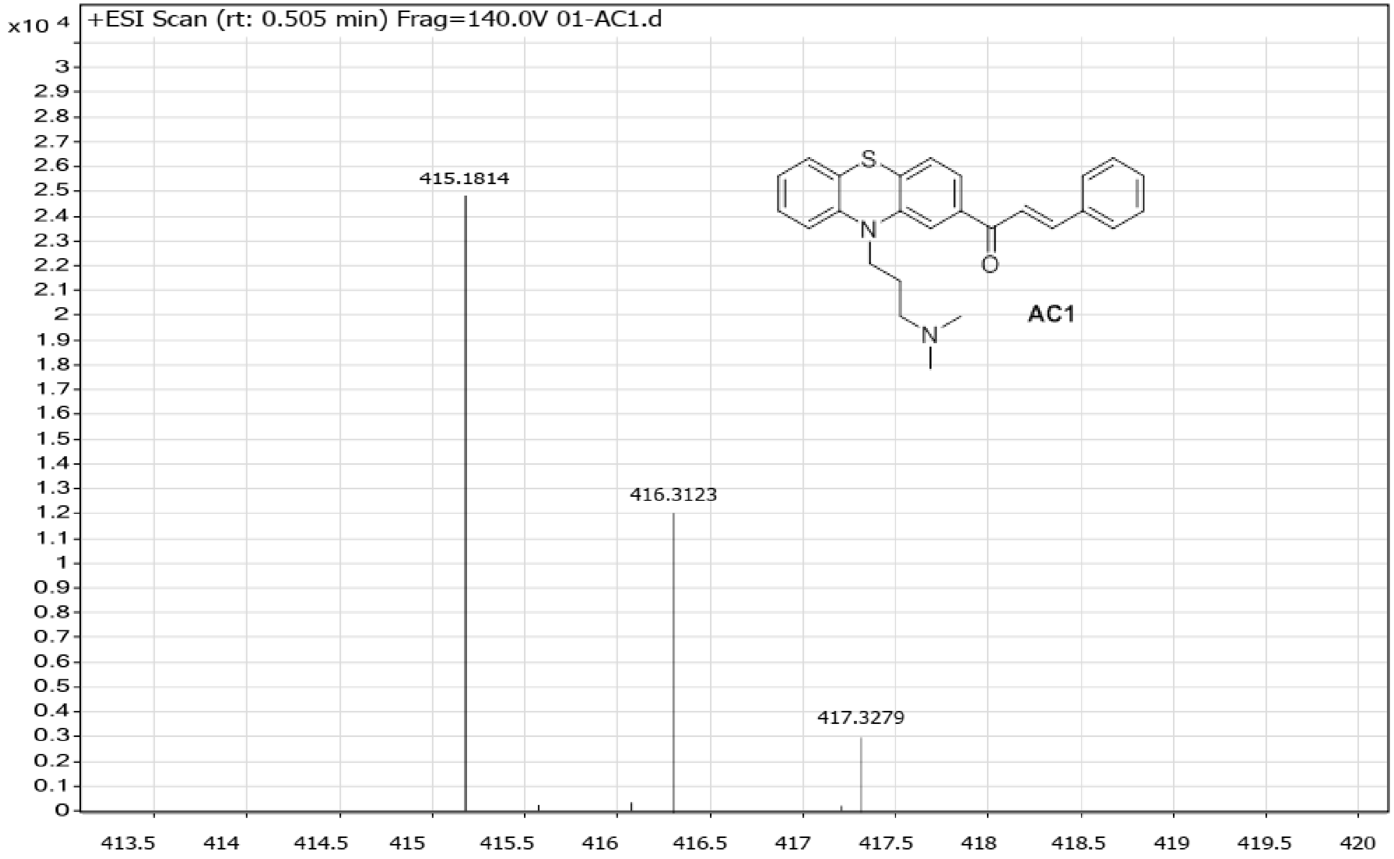

^{1}H-NMR

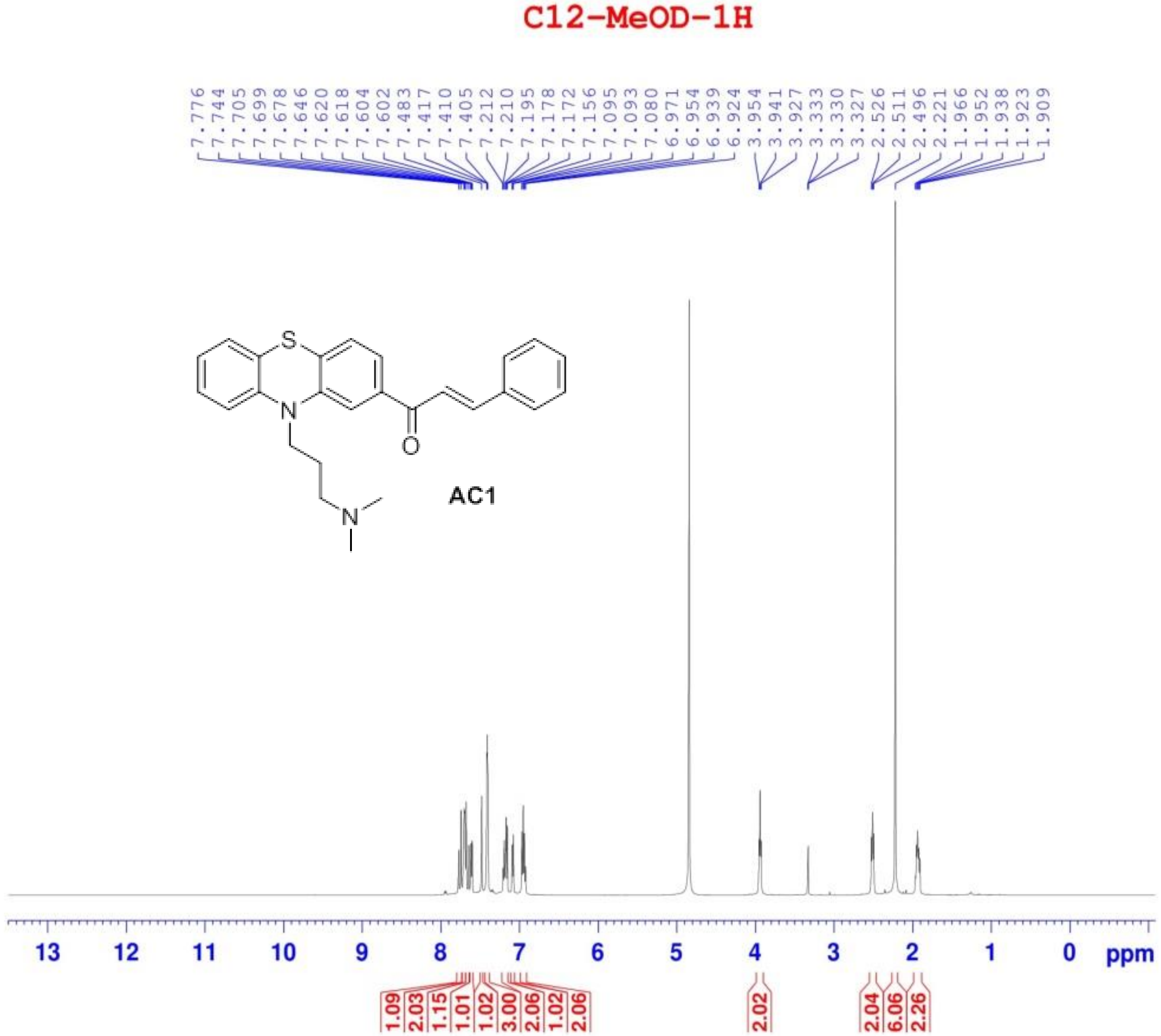


```
Current Data Parameters
NAME              114DAO_C12
EXPNO                      1
PROCNO                     1

F2 - Acquisition Parameters
Date_               20170704
Time                   11.31
INSTRUM                spect
PROBHD     5 mm PABBO BB/
PULPROG                 zg30
TD                     65536
SOLVENT                 MeOD
NS                        16
DS                         2
SWH            10000.000 Hz
FIDRES          0.152588 Hz
AQ             3.2767999 sec
RG                     50.73
DW                50.000 usec
DE                  6.50 usec
TE                   303.0 K
D1            1.00000000 sec
TD0                        1

======== CHANNEL f1 ========
SFO1         500.2030889 MHz
NUC1                      1H
P1                 10.00 usec
PLW1         22.00000000 W

F2 - Processing parameters
SI                     65536
SF           500.2000017 MHz
WDW                       EM
SSB        0
LB                   0.30 Hz
GB         0
PC                      1.00
```

^{1}H-NMR

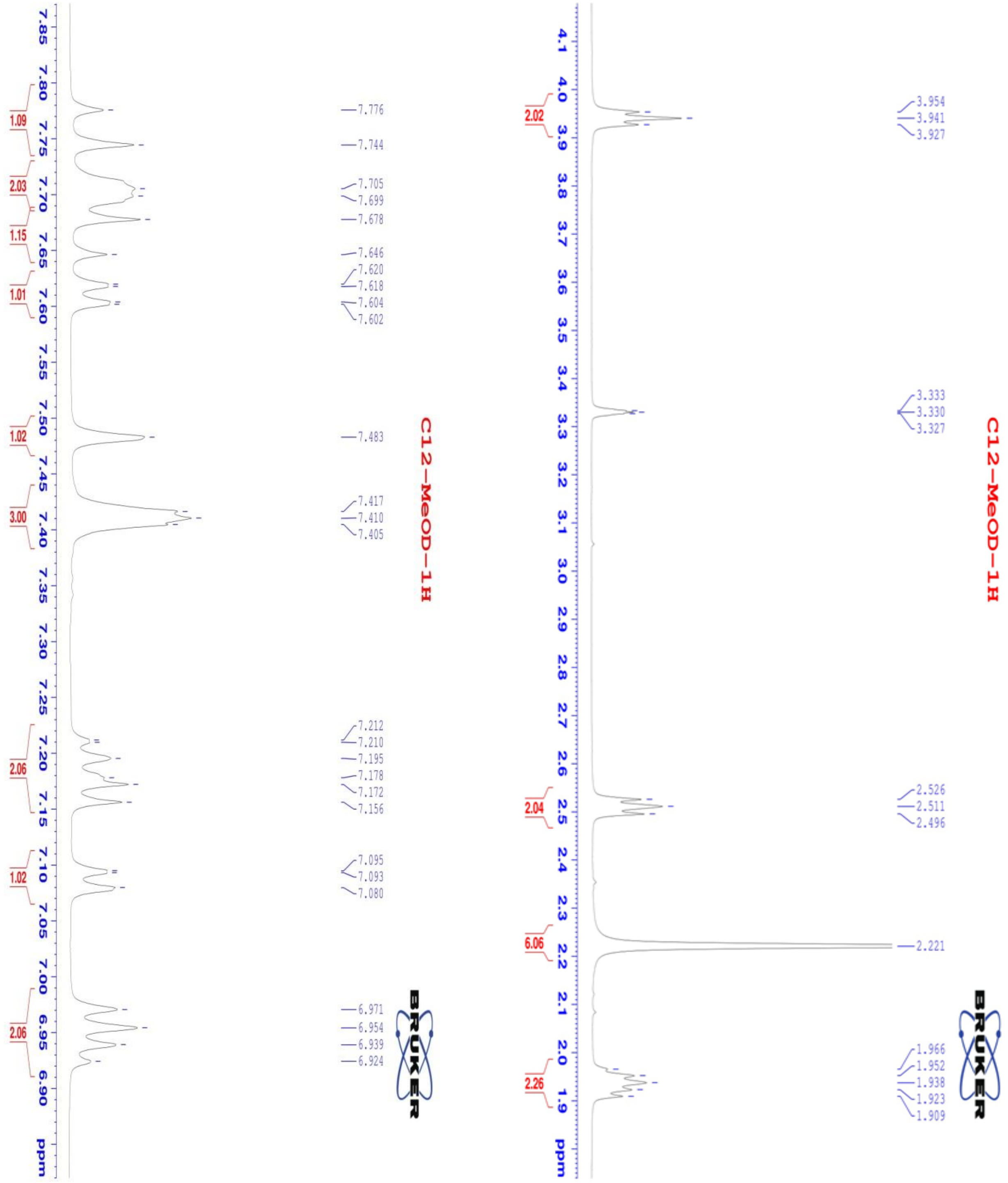
C12-MeOD-1H
7.776
7.744
7.705
7.699
7.678
7.646
7.620
7.618
7.604
7.602
7.483
7.417
7.410
7.405
7.212
7.210
7.195
7.178
7.172
7.156
7.095
7.093
7.080
6.971
6.954
6.939
6.924
3.954
3.941
3.927
3.333
3.330
3.327
2.526
2.511
2.496
2.221
1.966
1.952
1.938
1.923
1.909
ppm

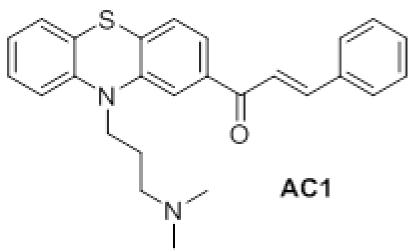
S
N
O
N

AC1

^{13}C-NMR

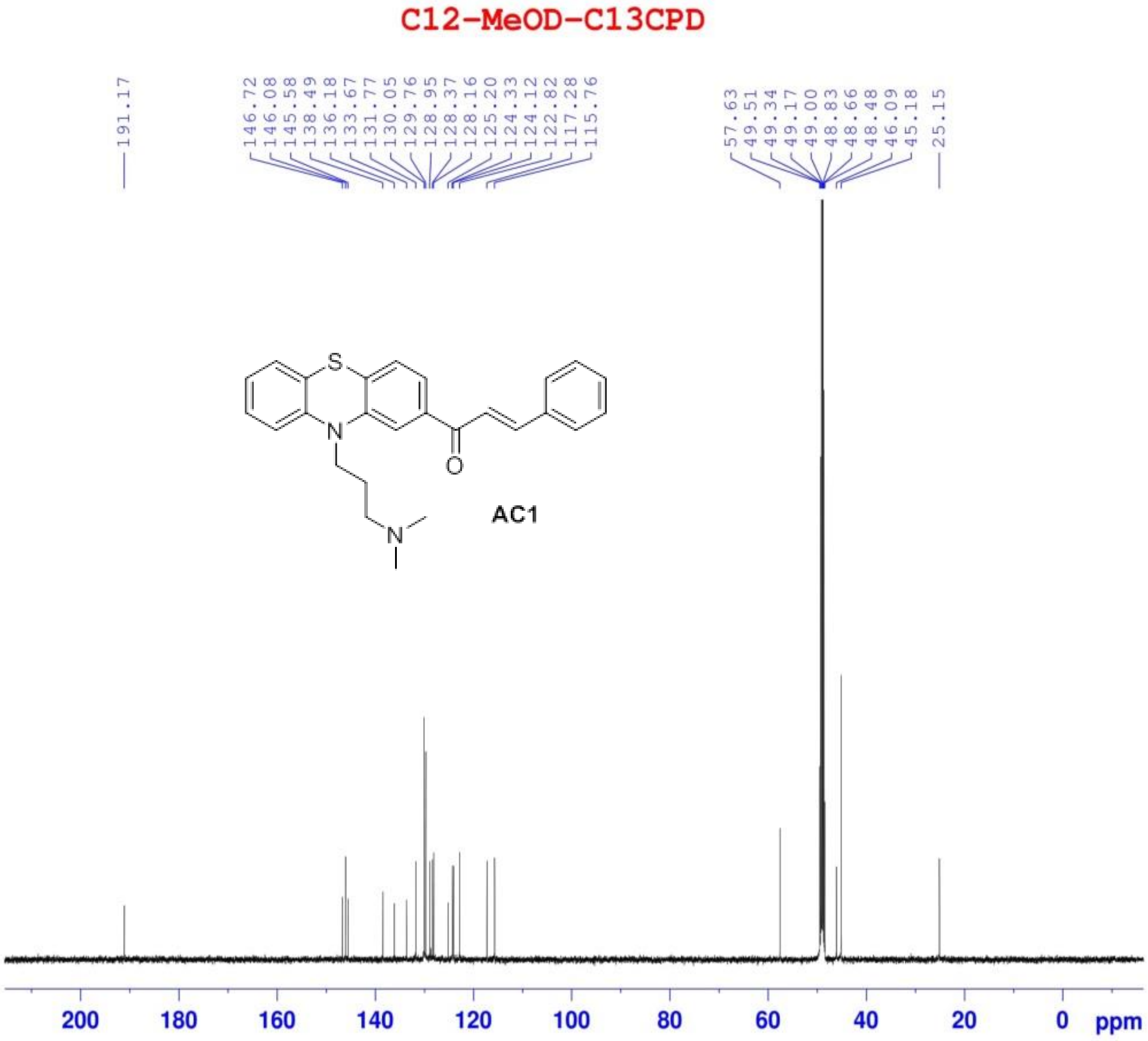

BRUKER

Current Data Parameters
NAME 114DAO_C12
EXPNO 2
PROCNO 1

F2 - Acquisition Parameters
Date_ 20170705
Time 14.51
INSTRUM spect
PROBHD 5 mm PABBO BB/
PULPROG zgpg30
TD 65536
SOLVENT MeOD
NS 128
DS 4
SWH 29761.904 Hz
FIDRES 0.454131 Hz
AQ 1.1010048 sec
RG 198.57
DW 16.800 usec
DE 6.50 usec
TE 303.0 K
D1 2.00000000 sec
D11 0.03000000 sec
TD0 1

======== CHANNEL f1 ========
SFO1 125.7879670 MHz
NUC1 13C
P1 10.00 usec
PLW1 88.00000000 W

======== CHANNEL f2 ========
SFO2 500.2020008 MHz
NUC2 1H
CPDPRG[2 waltz16
PCPD2 80.00 usec
PLW2 22.00000000 W
PLW12 0.34375000 W
PLW13 0.22000000 W

F2 - Processing parameters
SI 32768
SF 125.7752211 MHz
WDW EM
SSB 0
LB 1.00 Hz
GB 0
PC 1.40

^{13}C-NMR

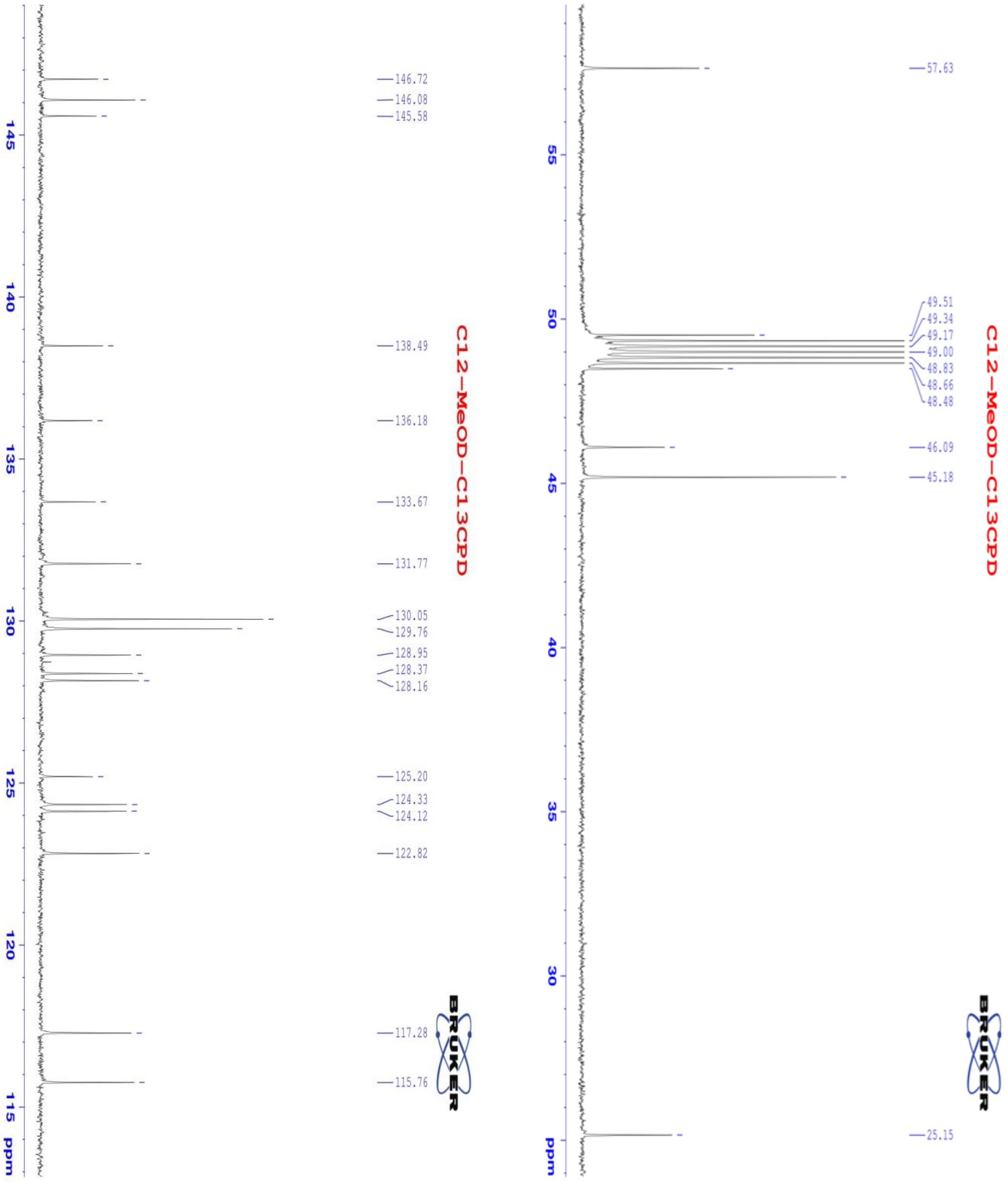

C12–MeOD–C13CPD
146.72
146.08
145.58
138.49
136.18
133.67
131.77
130.05
129.76
128.95
128.37
128.16
125.20
124.33
124.12
122.82
117.28
115.76
145
140
135
130
125
120
115 ppm
BRUKER
C12–MeOD–C13CPD
57.63
49.51
49.34
49.17
49.00
48.83
48.66
48.48
46.09
45.18
25.15
55
50
45
40
35
30
ppm
BRUKER

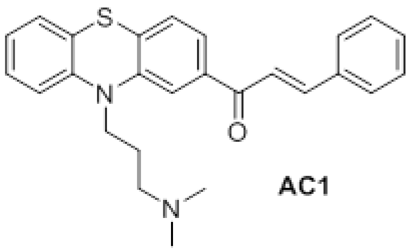

S
N
O
N

AC1

VIEN CONG NGHE HOA HOC
01 Mac Dinh Chi - Q.1 - Tp HCM

Tel: 08.38296127
Fax: 08.38293889

IR

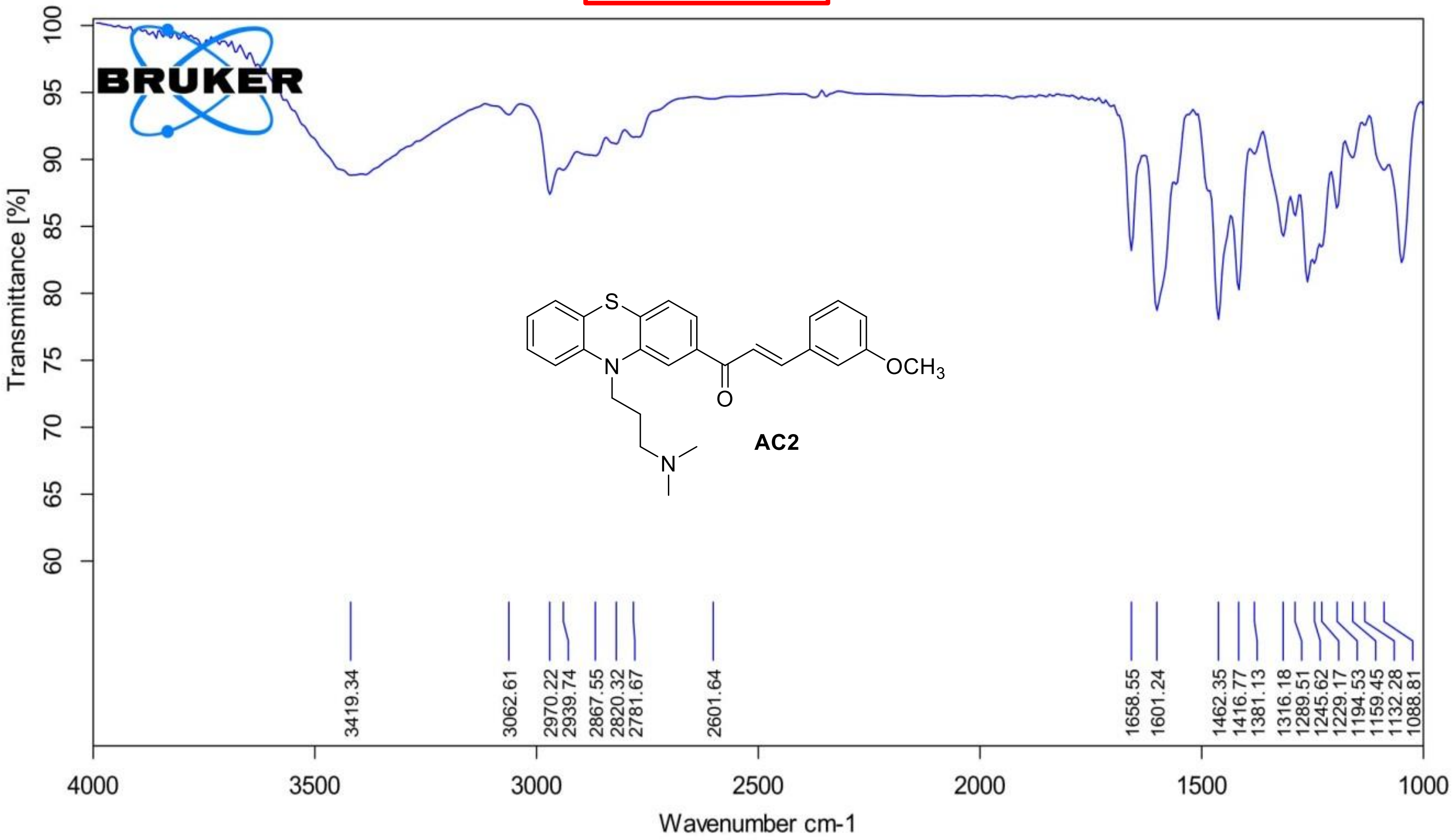

E:\OPUS 7\2017\THANG 6\20170606\C3.0	C3	TENSOR 27 - BRUKER - GERMANY	6/6/2017

MS

Qualitative Analysis Report

**Data Filename**	02-AC2.d	**Sample Name**	02-AC2
**Sample Type**	Sample	**Position**	P2-C6
**Instrument Name**	Instrument 1	**User Name**	
**Acq Method**	Cot ngan - MSMS_Pos.m	**Acquired Time**	22/08/2020 10:13:59 AM
**IRM Calibration Status**	Success	**DA Method**	COTNGAN.M.m
**Comment**			

**Sample Group**		**Info.**	
**Stream Name**	LC 1	**Acquisition SW Version**	6200 series TOF/6500 series Q-TOF B.06.01 (B6172 SP1)

User Chromatograms

**Fragmentor Voltage**	140	**Collision Energy**	0	**Ionization Mode**	ESI

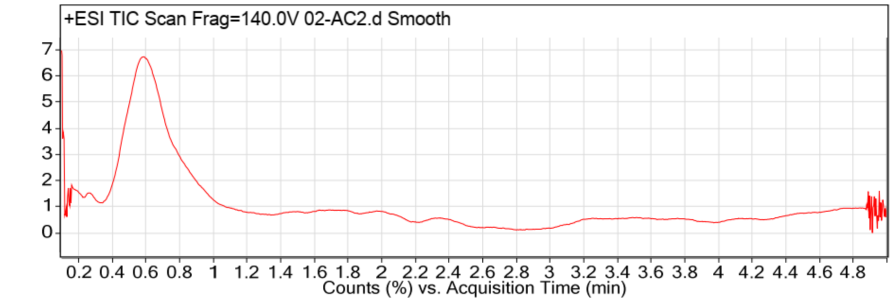

User Spectra

**Fragmentor Voltage**	**Collision Energy**	**Ionization Mode**
140	0	ESI

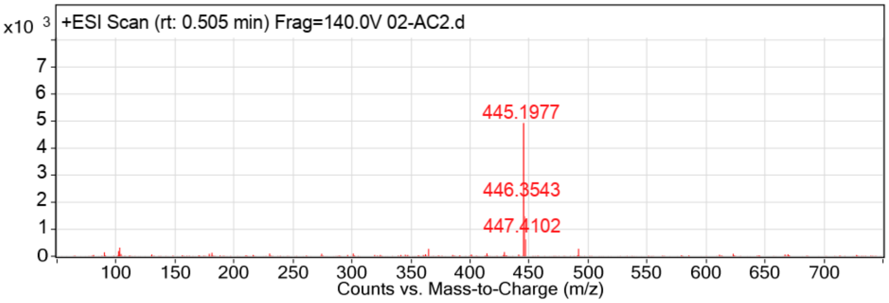

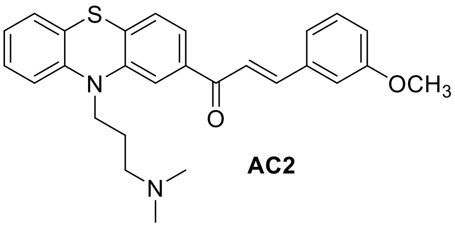

AC2

--- End Of Report ---

MS

**Sample Name**	02-AC2	**Position**	P2-C6	**Instrument Name**	Instrument 1	**User Name**	
**Inj Vol**	2	**InjPosition**		**SampleType**	Sample	**IRM Calibration Status**	Success
**Data Filename**	02-AC2.d	**ACQ Method**	Cot ngan - MSMS_Pos.	**Comment**		**Acquired Time**	22/08/2020 10:13:59 AM

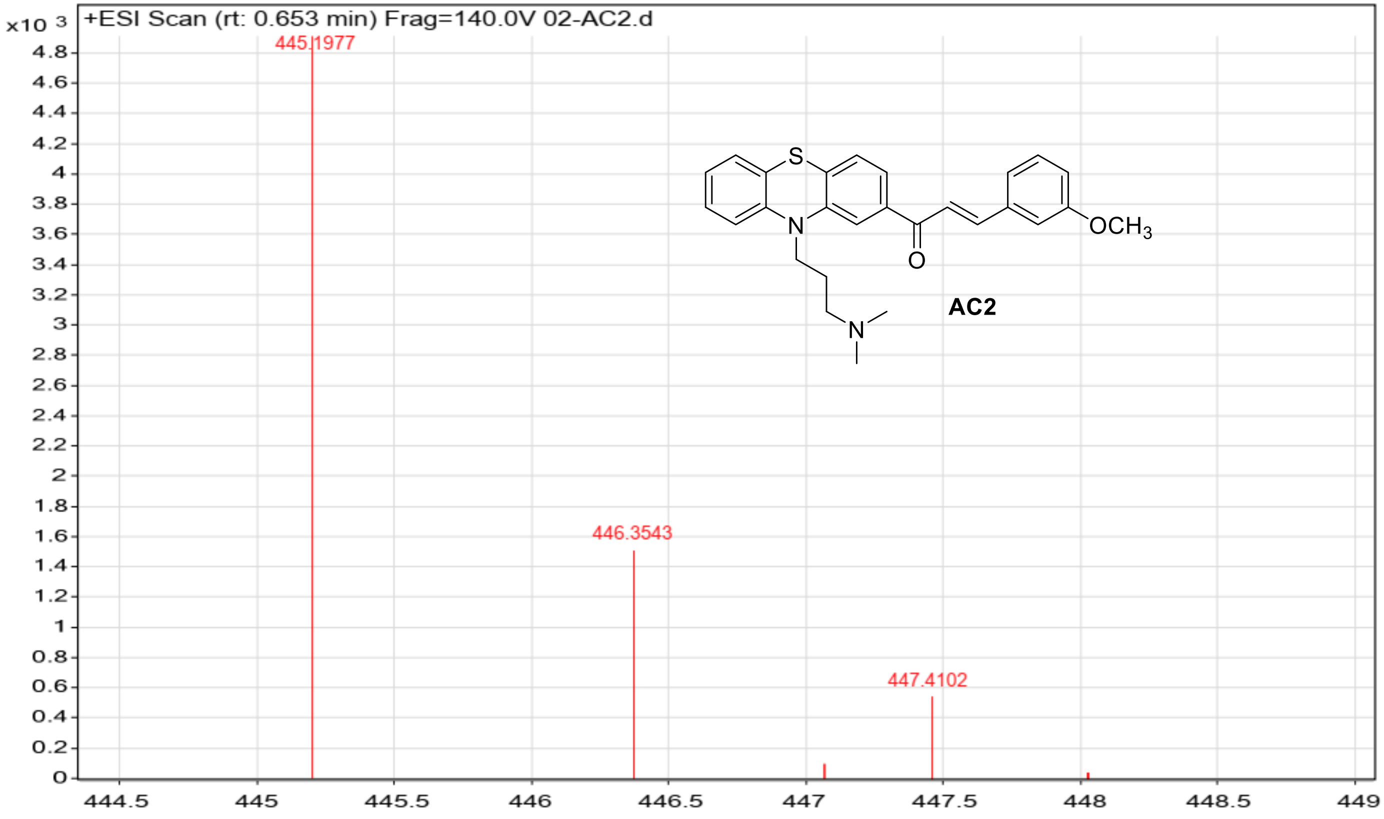

^{1}H-NMR

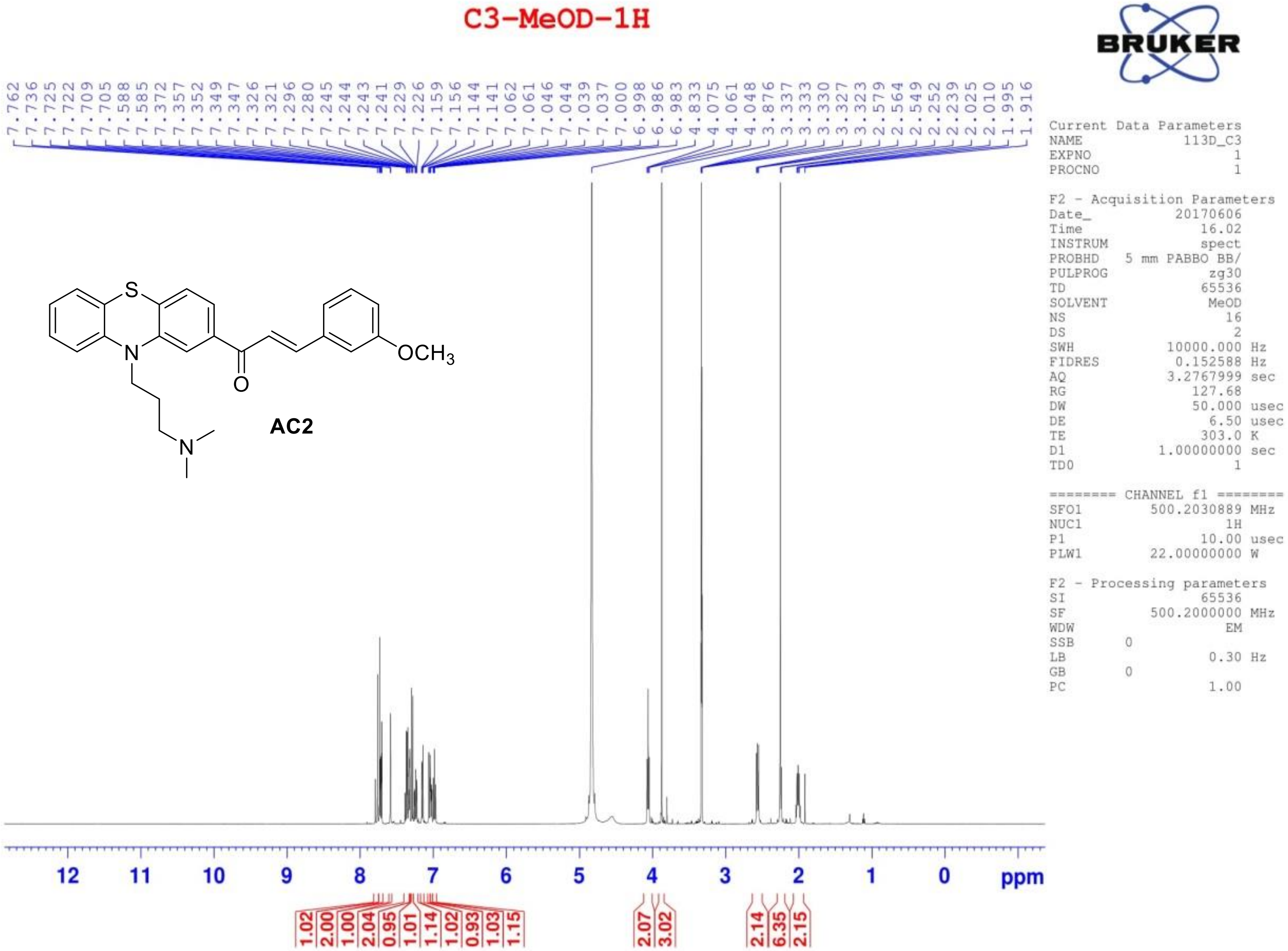

C3-MeOD-1H
BRUKER
7.762 7.736 7.725 7.722 7.709 7.705 7.588 7.585 7.372 7.357 7.352 7.349 7.347 7.326 7.321 7.296 7.280 7.245 7.244 7.243 7.241 7.229 7.226 7.159 7.156 7.144 7.141 7.062 7.061 7.046 7.044 7.039 7.037 7.000 6.998 6.986 6.983 4.833 4.075 4.061 4.048 3.876 3.337 3.333 3.330 3.327 3.323 2.579 2.564 2.549 2.252 2.239 2.025 2.010 1.995 1.916
S
N
O
OCH3
N
AC2
Current Data Parameters
NAME 113D_C3
EXPNO 1
PROCNO 1
F2 - Acquisition Parameters
Date_ 20170606
Time 16.02
INSTRUM spect
PROBHD 5 mm PABBO BB/
PULPROG zg30
TD 65536
SOLVENT MeOD
NS 16
DS 2
SWH 10000.000 Hz
FIDRES 0.152588 Hz
AQ 3.2767999 sec
RG 127.68
DW 50.000 usec
DE 6.50 usec
TE 303.0 K
D1 1.00000000 sec
TD0 1
======== CHANNEL f1 ========
SFO1 500.2030889 MHz
NUC1 1H
P1 10.00 usec
PLW1 22.00000000 W
F2 - Processing parameters
SI 65536
SF 500.2000000 MHz
WDW EM
SSB 0
LB 0.30 Hz
GB 0
PC 1.00
12 11 10 9 8 7 6 5 4 3 2 1 0 ppm
1.02 2.00 1.00 2.04 0.95 1.01 1.14 1.02 0.93 1.03 1.15 2.07 3.02 2.14 6.35 2.15

^{1}H-NMR

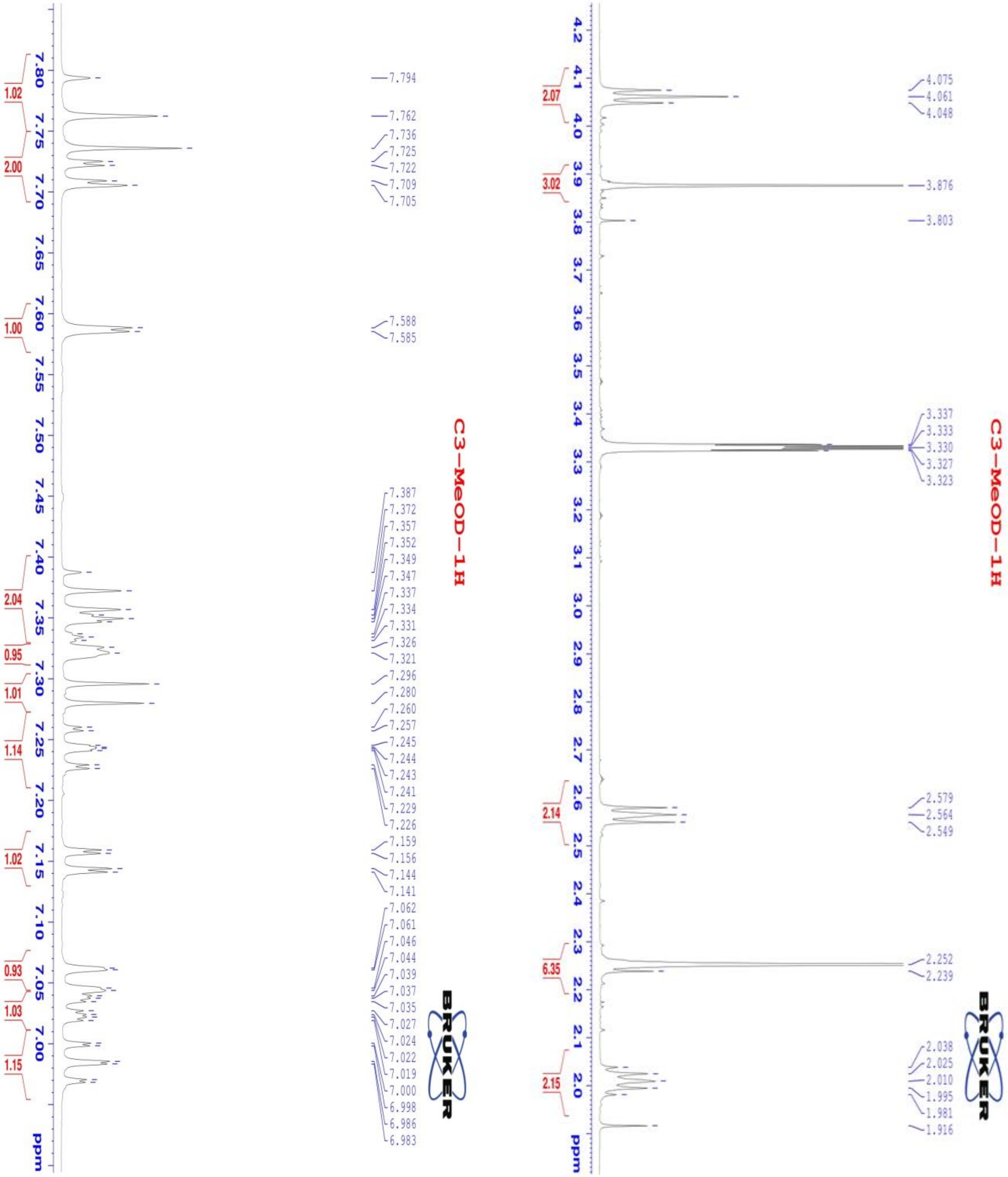
7.794
7.762
7.736
7.725
7.722
7.709
7.705
7.588
7.585
7.387
7.372
7.357
7.352
7.349
7.347
7.337
7.334
7.331
7.326
7.321
7.296
7.280
7.260
7.257
7.245
7.244
7.243
7.241
7.229
7.226
7.159
7.156
7.144
7.141
7.062
7.061
7.046
7.044
7.039
7.037
7.035
7.027
7.024
7.022
7.019
7.000
6.998
6.986
6.983
C3–MeOD–1H
4.075
4.061
4.048
3.876
3.803
3.337
3.333
3.330
3.327
3.323
2.579
2.564
2.549
2.252
2.239
2.038
2.025
2.010
1.995
1.981
1.916
C3–MeOD–1H

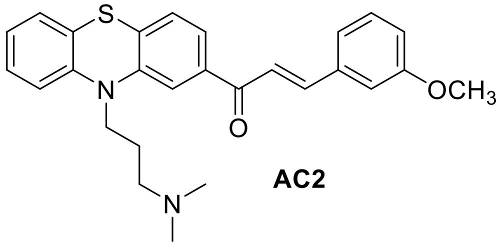
S
N
O
OCH3
N

AC2

^{13}C-NMR

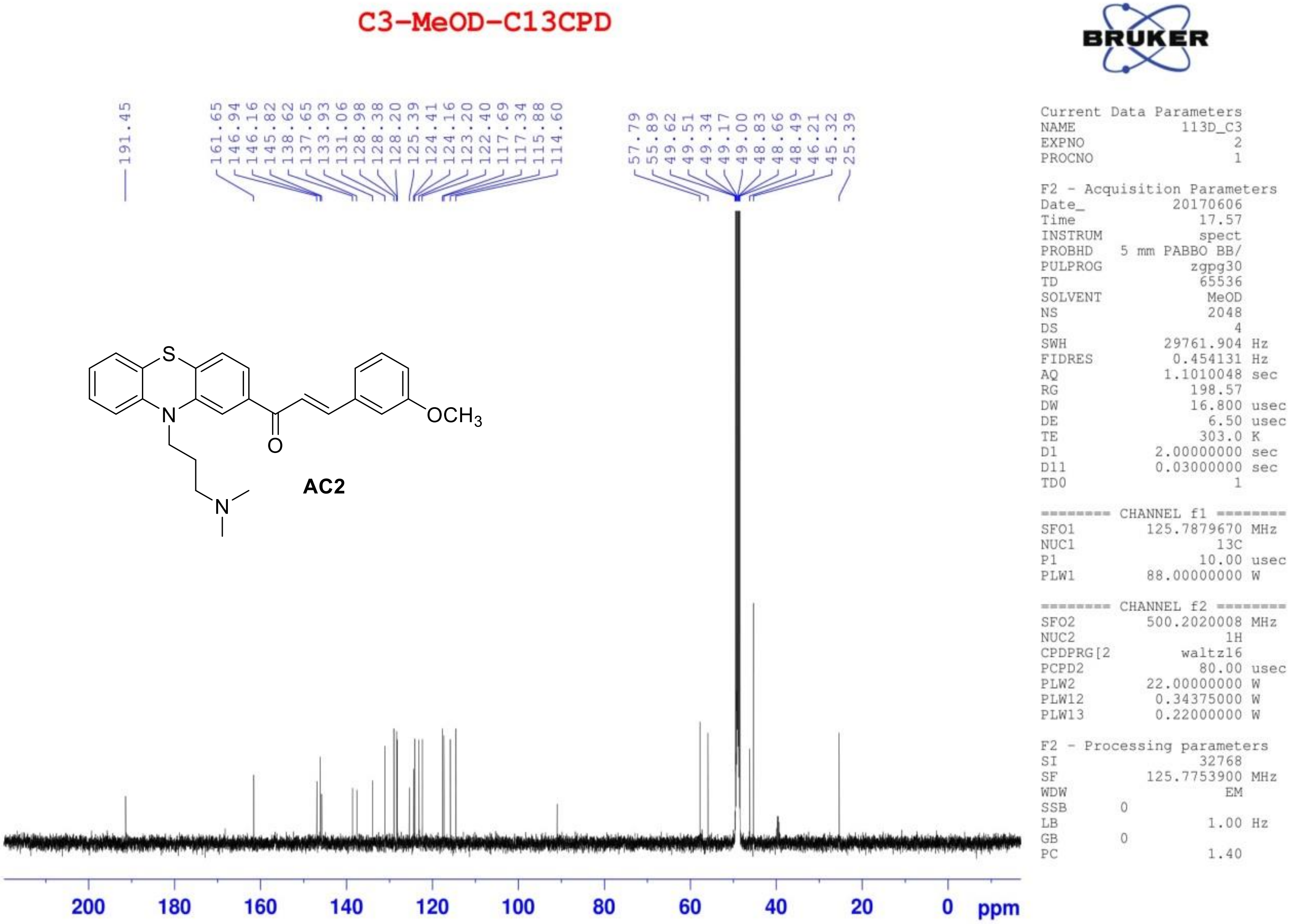

C3-MeOD-C13CPD
191.45
161.65
146.94
146.16
145.82
138.62
137.65
133.93
131.06
128.98
128.38
128.20
125.39
124.41
124.16
123.20
122.40
117.69
117.34
115.88
114.60
57.79
55.89
49.62
49.51
49.34
49.17
49.00
48.83
48.66
48.49
46.21
45.32
25.39
200 180 160 140 120 100 80 60 40 20 0 ppm
BRUKER
Current Data Parameters
NAME 113D_C3
EXPNO 2
PROCNO 1
F2 - Acquisition Parameters
Date_ 20170606
Time 17.57
INSTRUM spect
PROBHD 5 mm PABBO BB/
PULPROG zgpg30
TD 65536
SOLVENT MeOD
NS 2048
DS 4
SWH 29761.904 Hz
FIDRES 0.454131 Hz
AQ 1.1010048 sec
RG 198.57
DW 16.800 usec
DE 6.50 usec
TE 303.0 K
D1 2.00000000 sec
D11 0.03000000 sec
TD0 1
======== CHANNEL f1 ========
SFO1 125.7879670 MHz
NUC1 13C
P1 10.00 usec
PLW1 88.00000000 W
======== CHANNEL f2 ========
SFO2 500.2020008 MHz
NUC2 1H
CPDPRG[2 waltz16
PCPD2 80.00 usec
PLW2 22.00000000 W
PLW12 0.34375000 W
PLW13 0.22000000 W
F2 - Processing parameters
SI 32768
SF 125.7753900 MHz
WDW EM
SSB 0
LB 1.00 Hz
GB 0
PC 1.40
S
N
O
OCH3
N

AC2

^{13}C-NMR

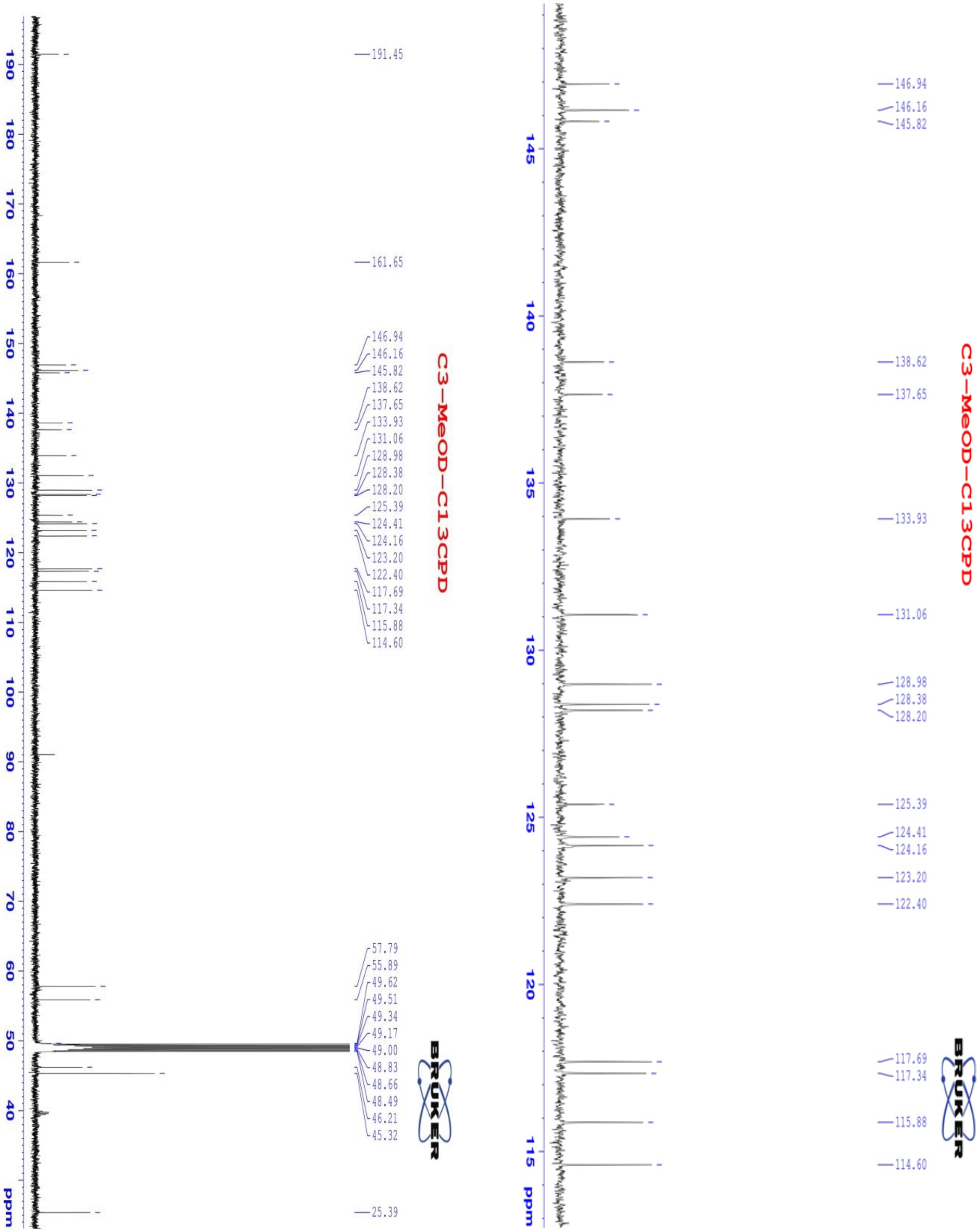
191.45
161.65
146.94
146.16
145.82
138.62
137.65
133.93
131.06
128.98
128.38
128.20
125.39
124.41
124.16
123.20
122.40
117.69
117.34
115.88
114.60
57.79
55.89
49.62
49.51
49.34
49.17
49.00
48.83
48.66
48.49
46.21
45.32
25.39
C3-MeOD-C13CPD
BRUKER
ppm

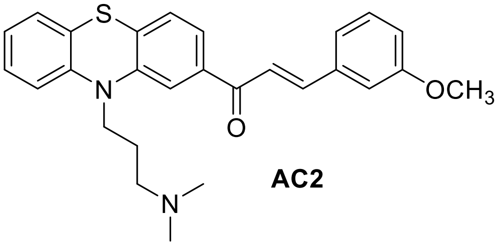
S
N
O
OCH3
N

AC2

^{13}C-NMR

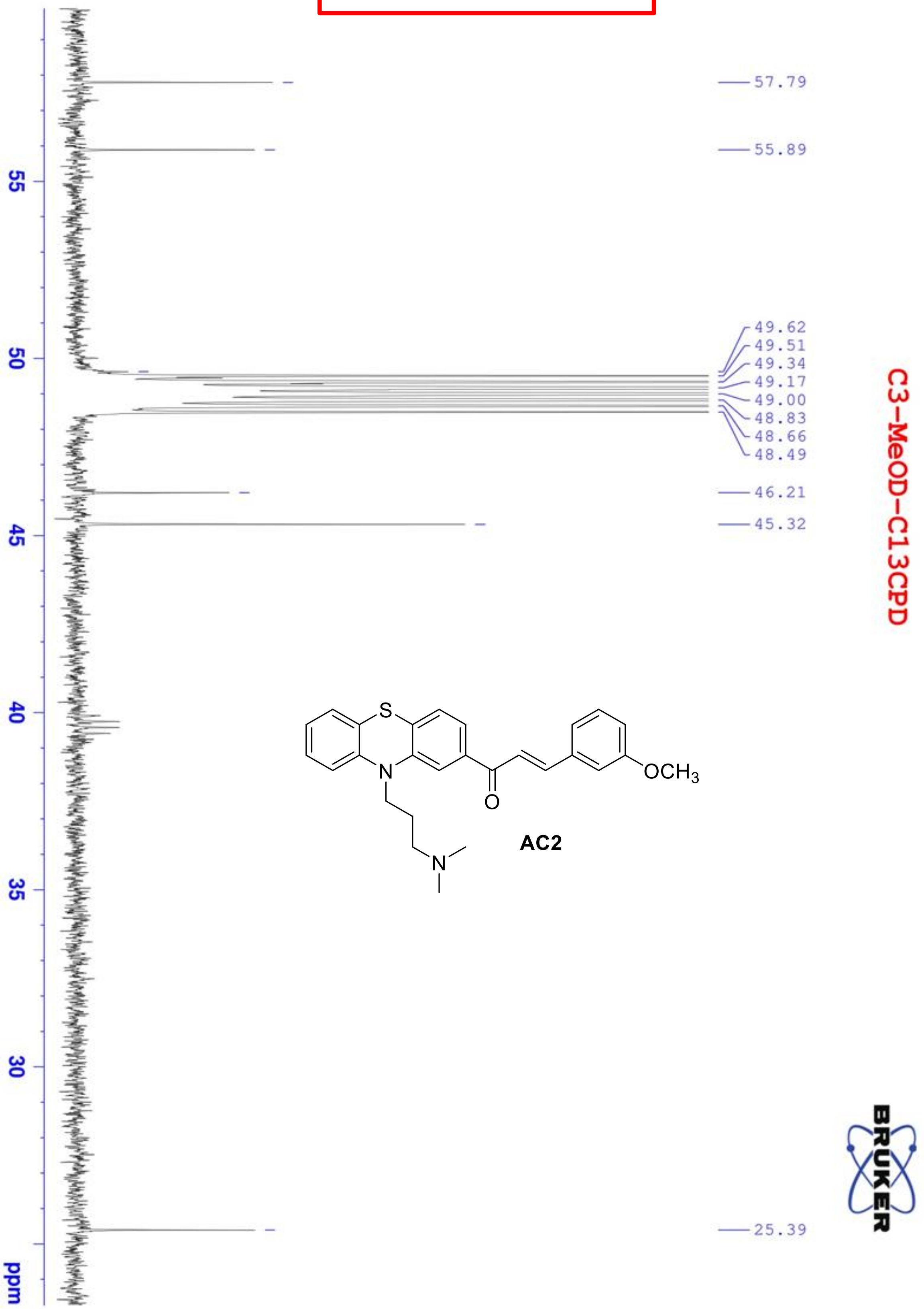
57.79
55.89
49.62
49.51
49.34
49.17
49.00
48.83
48.66
48.49
46.21
45.32
25.39
55
50
45
40
35
30
ppm
C3-MeOD-C13CPD
S
N
O
OCH3
N
AC2
BRUKER

VIEN CONG NGHE HOA HOC
01 Mac Dinh Chi - Q.1 - Tp HCM

IR

Tel: 08.38296127
Fax: 08.38293889

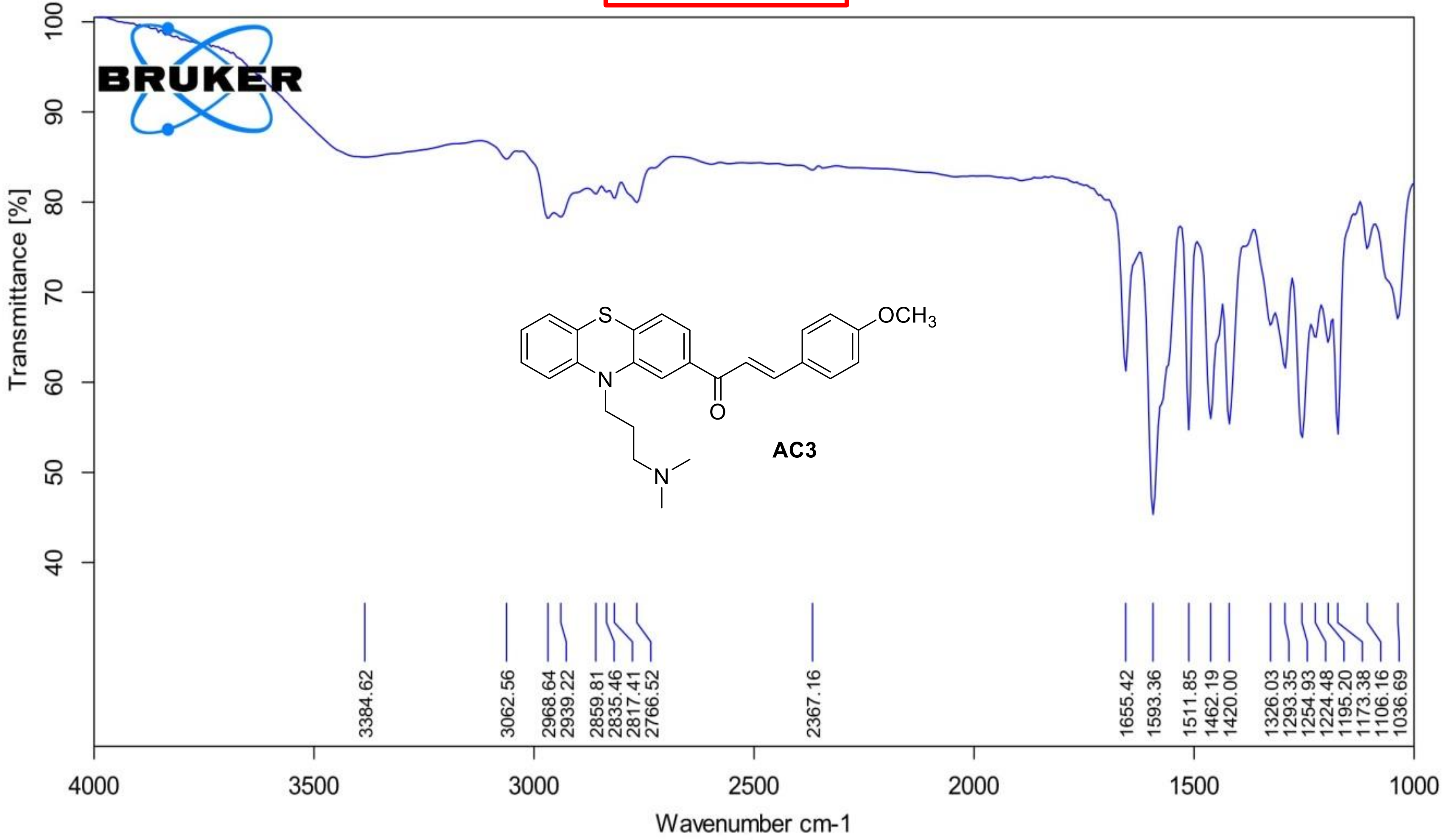

E:\OPUS 7\2017\THANG 6\20170606\C2.0	C2	TENSOR 27 - BRUKER - GERMANY	6/6/2017

MS

Qualitative Analysis Report

**Data Filename**	03-AC3.d	**Sample Name**	03-AC3
**Sample Type**	Sample	**Position**	P2-C4
**Instrument Name**	Instrument 1	**User Name**	
**Acq Method**	Cot ngan - MSMS_Pos.m	**Acquired Time**	22/08/2020 10:21:51 AM
**IRM Calibration Status**	Success	**DA Method**	COTNGAN.M.m
**Comment**			

**Sample Group**		**Info.**	
**Stream Name**	LC 1	**Acquisition SW Version**	6200 series TOF/6500 series Q-TOF B.06.01 (B6172 SP1)

User Chromatograms

Fragmentor Voltage 140 **Collision Energy** 0 **Ionization Mode** ESI

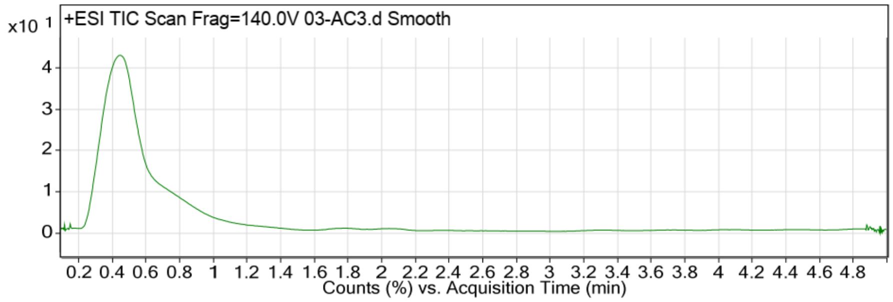

User Spectra

Fragmentor Voltage	Collision Energy	Ionization Mode
140	0	ESI

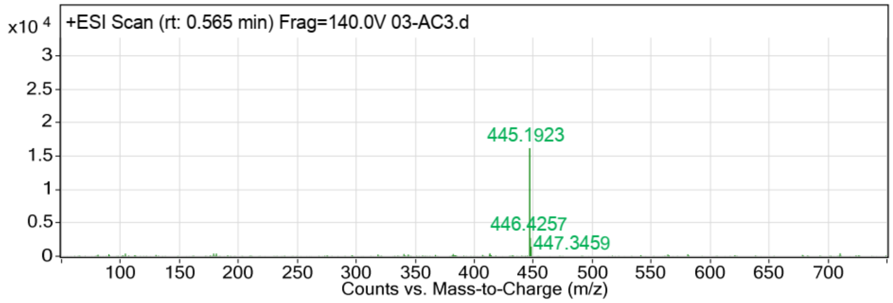

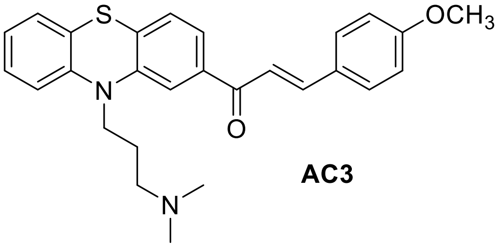

AC3

--- End Of Report ---

MS

Sample Name	03-AC3	Position	P2-C4	Instrument Name	Instrument 1	User Name	
Inj Vol	2	InjPosition		SampleType	Sample	IRM Calibration Status	Success
Data Filename	03-AC3.d	ACQ Method	Cot ngan - MSMS_Pos.	Comment		Acquired Time	22/08/2020 10:21:51 PM

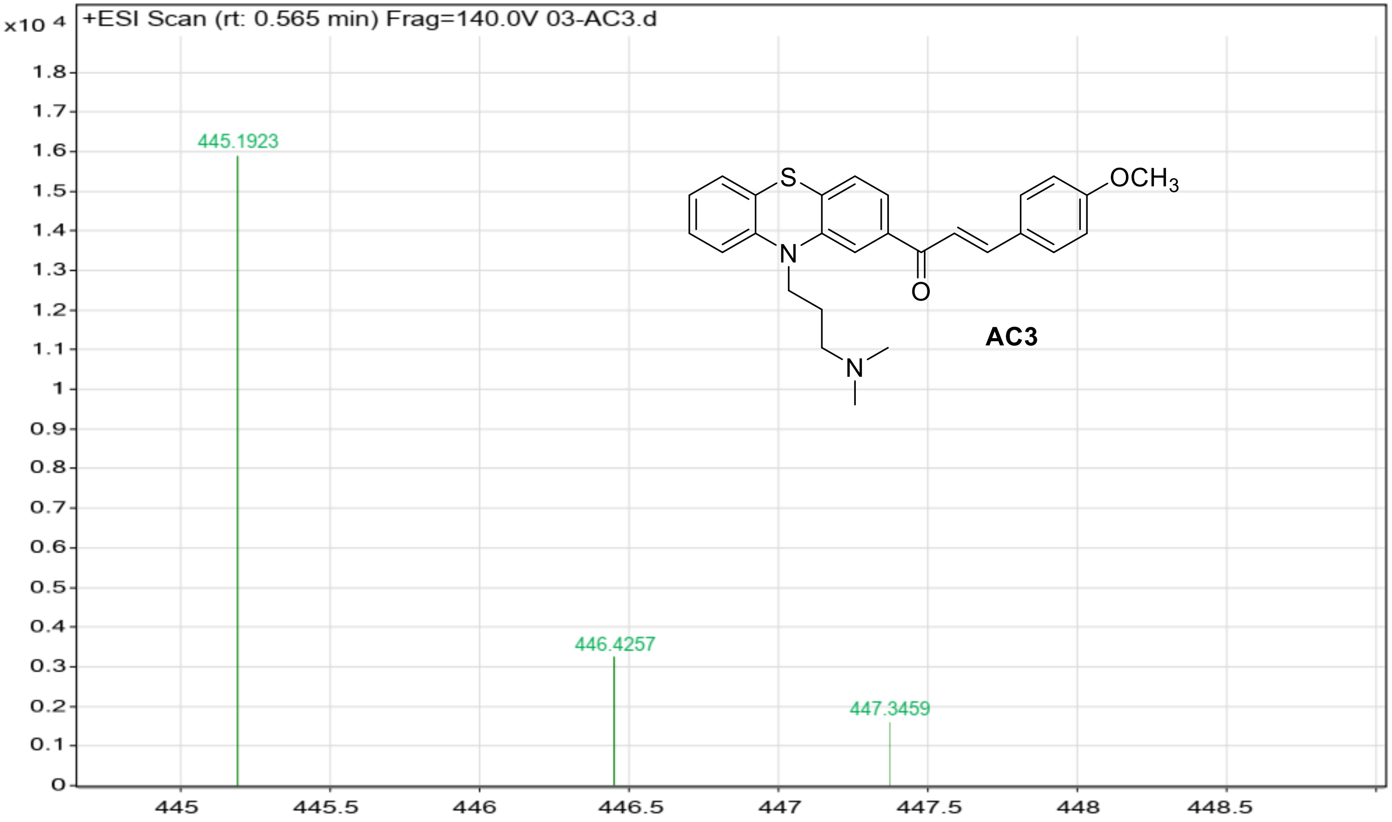

^{1}H-NMR

C2-MeOD-1H

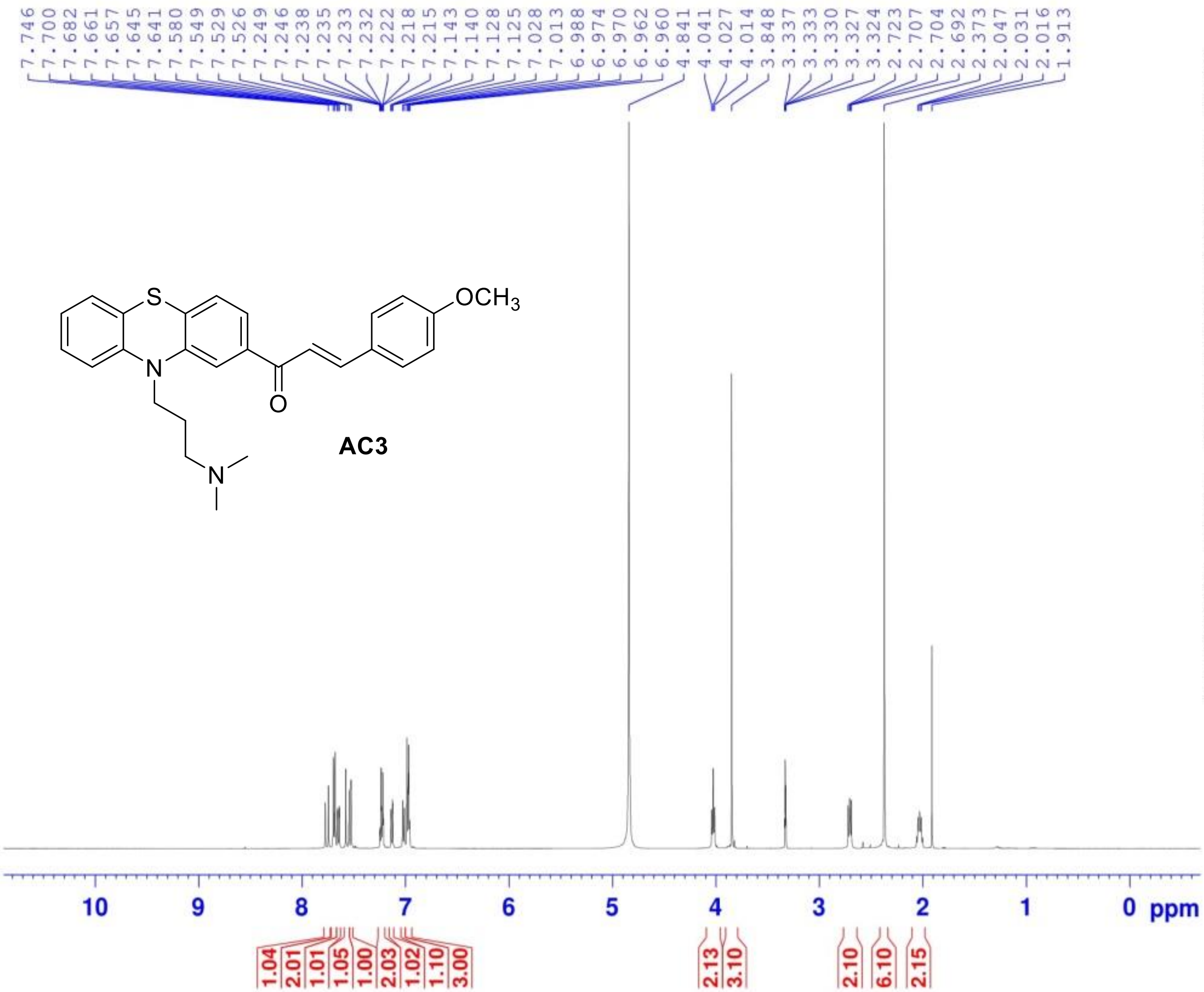

AC3

Current Data Parameters
NAME 113D_C2
EXPNO 1
PROCNO 1

F2 - Acquisition Parameters
Date_ 20170606
Time 15.13
INSTRUM spect
PROBHD 5 mm PABBO BB/
PULPROG zg30
TD 65536
SOLVENT MeOD
NS 16
DS 2
SWH 10000.000 Hz
FIDRES 0.152588 Hz
AQ 3.2767999 sec
RG 79.36
DW 50.000 usec
DE 6.50 usec
TE 303.0 K
D1 1.00000000 sec
TD0 1

======== CHANNEL f1 ========
SFO1 500.2030889 MHz
NUC1 1H
P1 10.00 usec
PLW1 22.00000000 W

F2 - Processing parameters
SI 65536
SF 500.2000002 MHz
WDW EM
SSB 0
LB 0.30 Hz
GB 0
PC 1.00

^{1}H-NMR

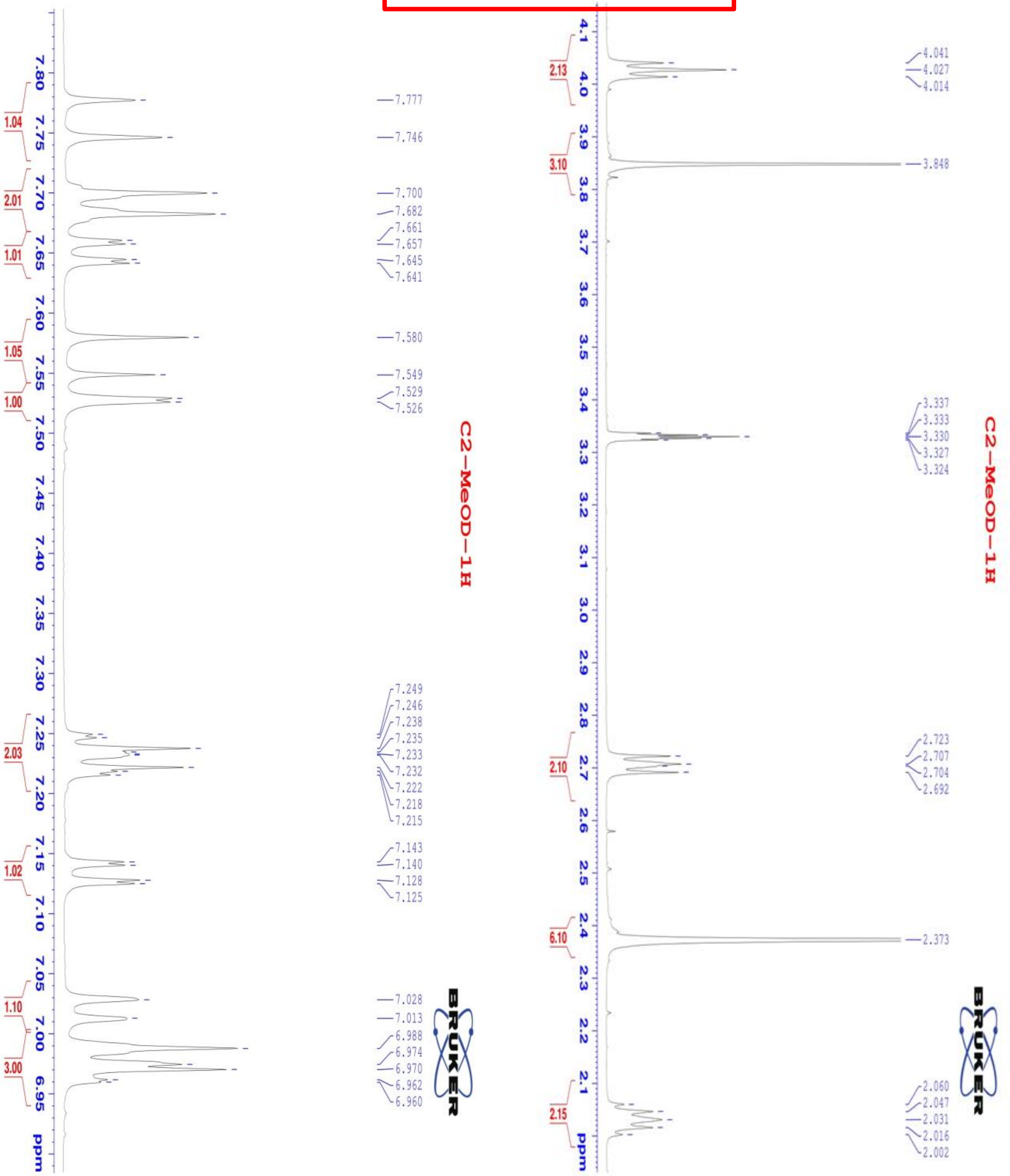

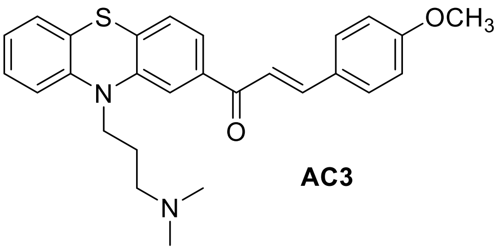

AC3

^{13}C-NMR

C2-MeOD-C13CPD

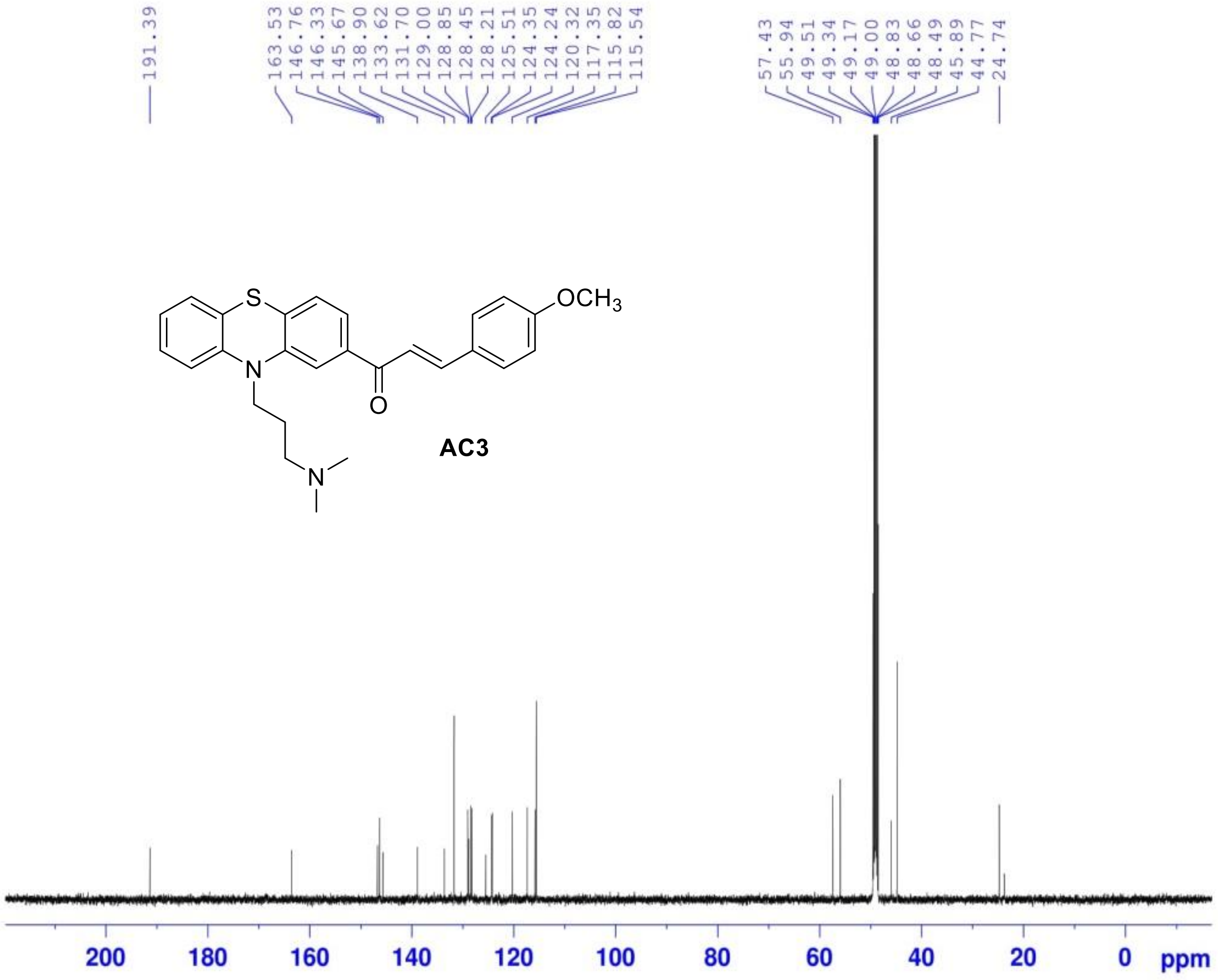

AC3

BRUKER

Current Data Parameters
NAME 113D_C2
EXPNO 2
PROCNO 1

F2 - Acquisition Parameters
Date_ 20170606
Time 15.57
INSTRUM spect
PROBHD 5 mm PABBO BB/
PULPROG zgpg30
TD 65536
SOLVENT MeOD
NS 256
DS 4
SWH 29761.904 Hz
FIDRES 0.454131 Hz
AQ 1.1010048 sec
RG 198.57
DW 16.800 usec
DE 6.50 usec
TE 303.0 K
D1 2.00000000 sec
D11 0.03000000 sec
TD0 1

======== CHANNEL f1 ========
SFO1 125.7879670 MHz
NUC1 13C
P1 10.00 usec
PLW1 88.00000000 W

======== CHANNEL f2 ========
SFO2 500.2020008 MHz
NUC2 1H
CPDPRG[2 waltz16
PCPD2 80.00 usec
PLW2 22.00000000 W
PLW12 0.34375000 W
PLW13 0.22000000 W

F2 - Processing parameters
SI 32768
SF 125.7753900 MHz
WDW EM
SSB 0
LB 1.00 Hz
GB 0
PC 1.40

^{13}C-NMR

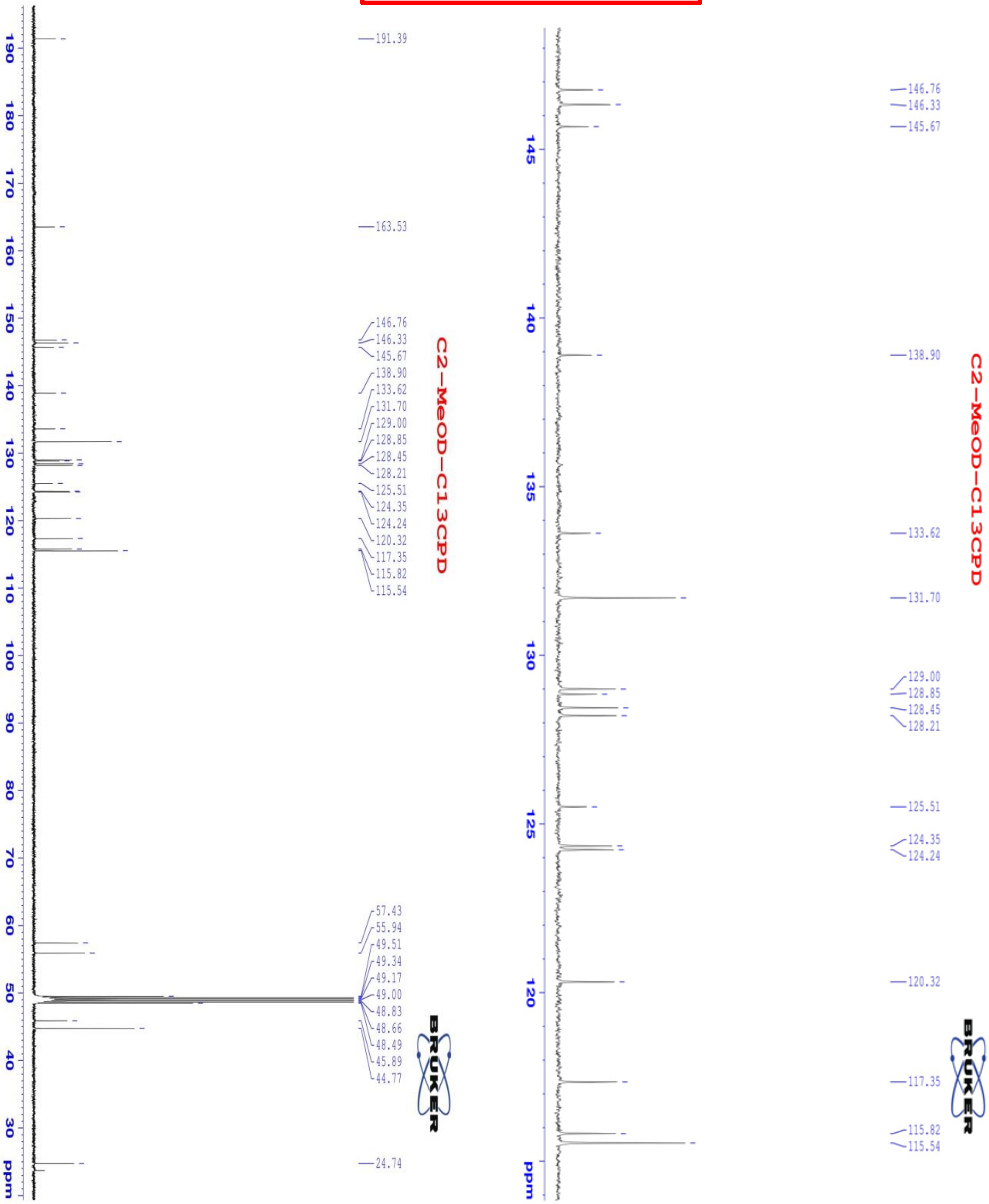
191.39
163.53
146.76
146.33
145.67
138.90
133.62
131.70
129.00
128.85
128.45
128.21
125.51
124.35
124.24
120.32
117.35
115.82
115.54
57.43
55.94
49.51
49.34
49.17
49.00
48.83
48.66
48.49
45.89
44.77
24.74
C2-MeOD-C13CPD
BRUKER
ppm

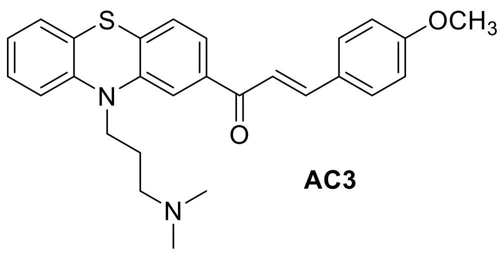
S
N
O
OCH3

AC3

^{13}C-NMR

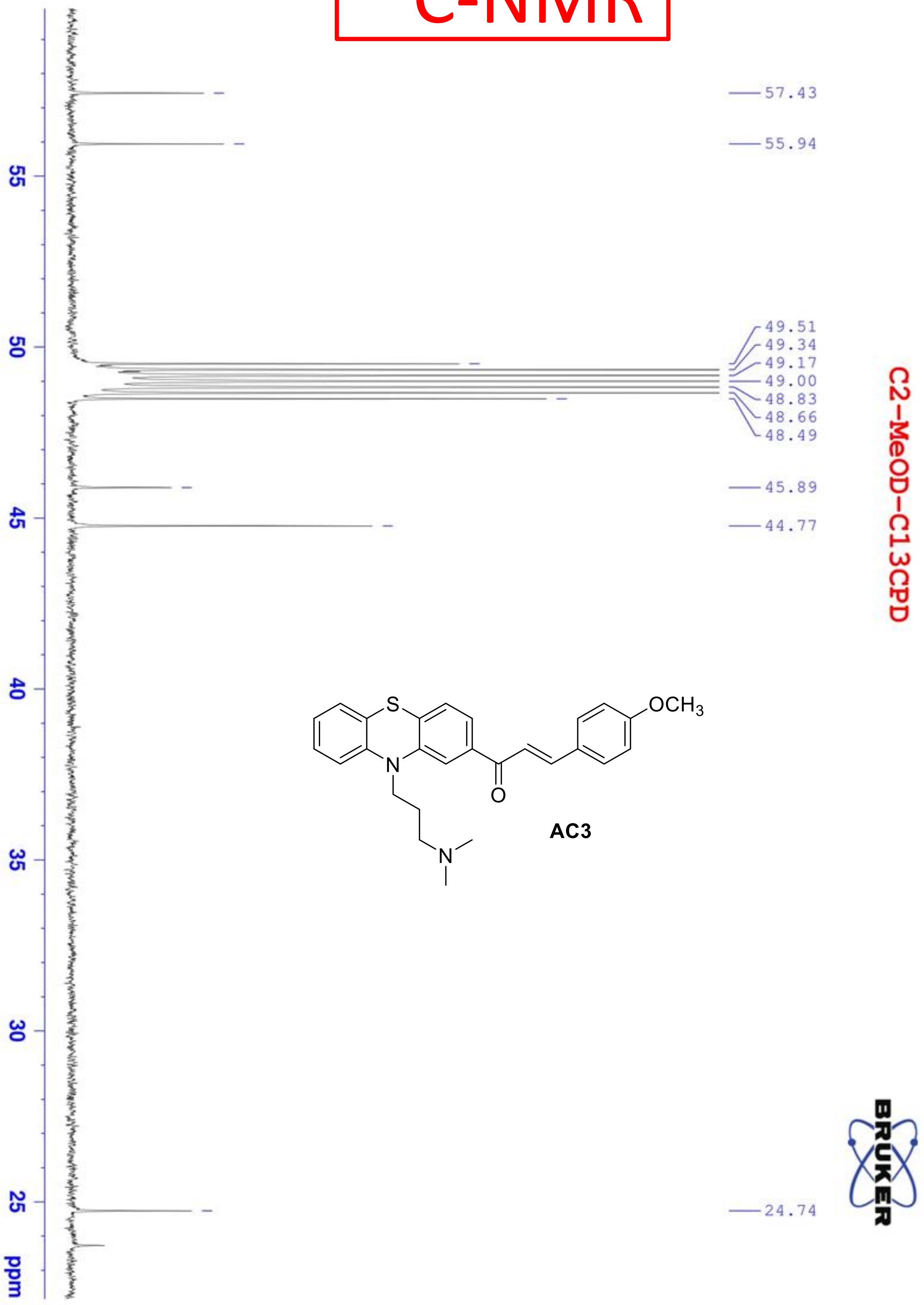

57.43
55.94
49.51
49.34
49.17
49.00
48.83
48.66
48.49
45.89
44.77
24.74
55
50
45
40
35
30
25
ppm
C2–MeOD–C13CPD
S
N
O
OCH3
N
AC3
BRUKER

IR

VIEN CONG NGHE HOA HOC
01 Mac Dinh Chi - Q.1 - Tp HCM

Tel: 08.38296127
Fax: 08.38293889

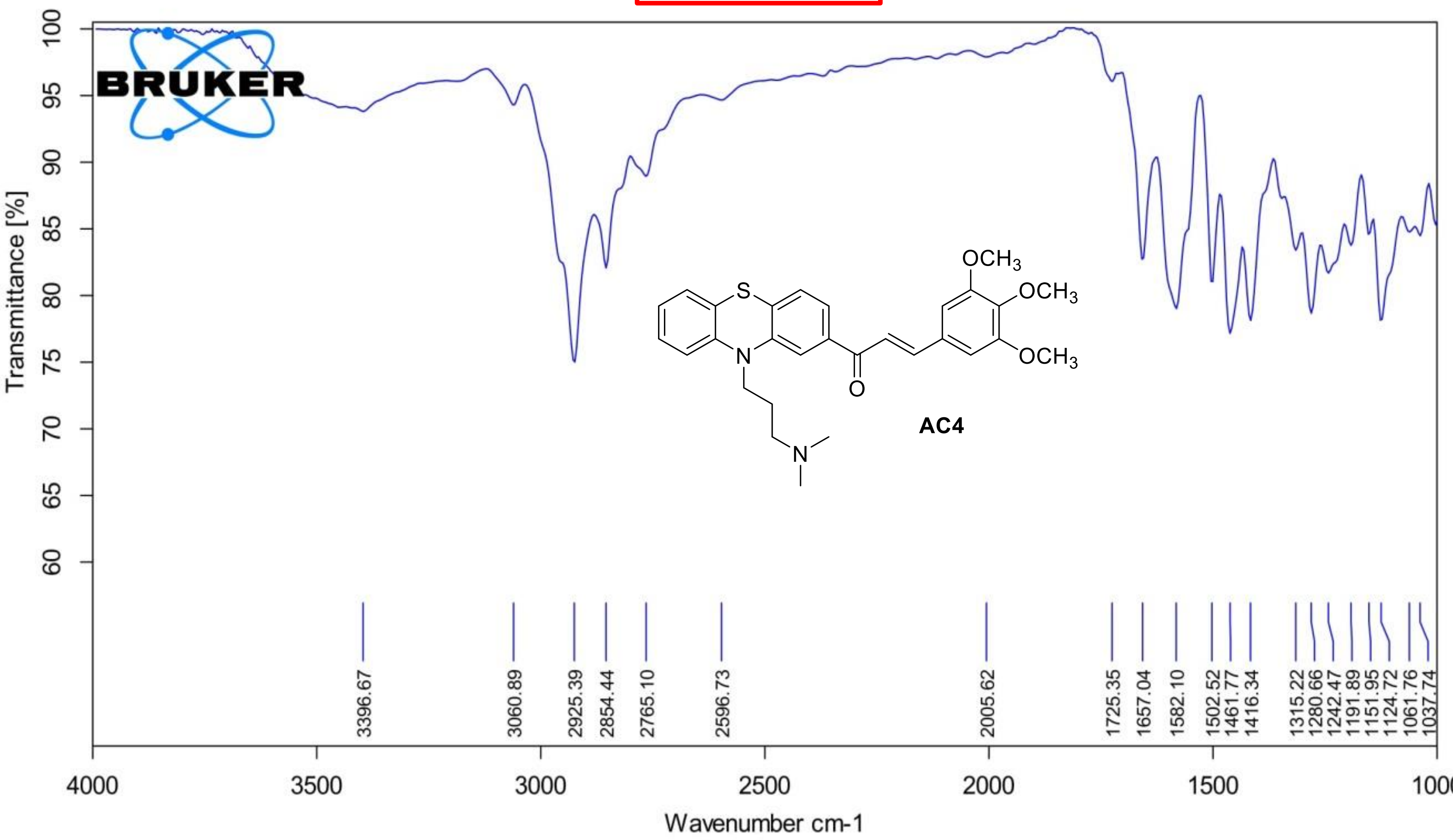

E:\OPUS 7\2017\THANG 6\20170606\C4.0	C4	TENSOR 27 - BRUKER - GERMANY	6/6/2017

MS

Qualitative Analysis Report

**Data Filename**	04-AC4.d	**Sample Name**	04-AC4
**Sample Type**	Sample	**Position**	P2-B5
**Instrument Name**	Instrument 1	**User Name**	
**Acq Method**	Cot ngan - MSMS_Pos.m	**Acquired Time**	22/08/2020 10:30:20 AM
**IRM Calibration Status**	Success	**DA Method**	COTNGAN.M.m
**Comment**			

**Sample Group**		**Info.**	
**Stream Name**	LC 1	**Acquisition SW Version**	6200 series TOF/6500 series Q-TOF B.06.01 (B6172 SP1)

User Chromatograms

Fragmentor Voltage 140 **Collision Energy** 0 **Ionization Mode** ESI

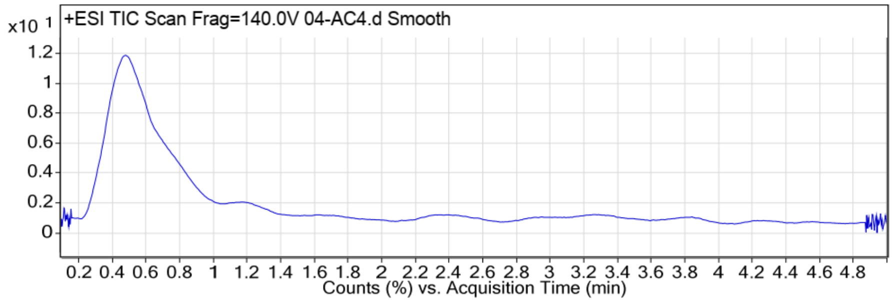

User Spectra

Fragmentor Voltage	Collision Energy	Ionization Mode
140	0	ESI

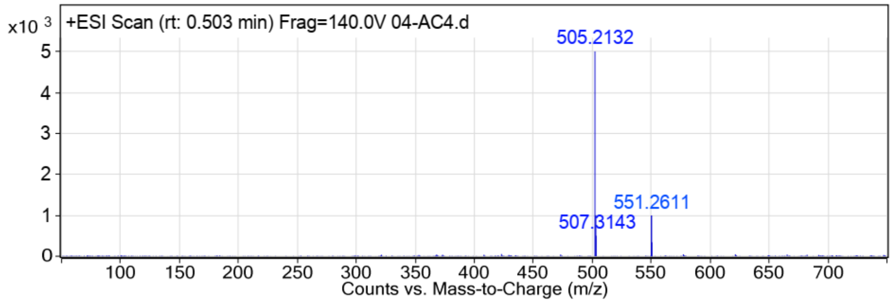

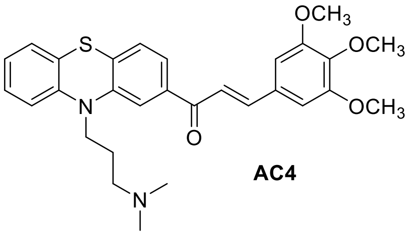

AC4

--- End Of Report ---

MS

**Sample Name**	04-AC4	**Position**	P2-B5	**Instrument Name**	Instrument 1	**User Name**	
**Inj Vol**	2	**InjPosition**		**SampleType**	Sample	**IRM Calibration Status**	Success
**Data Filename**	04-AC4.d	**ACQ Method**	Cot ngan - MSMS_Pos.	**Comment**		**Acquired Time**	22/08/2020 10:30:20 AM

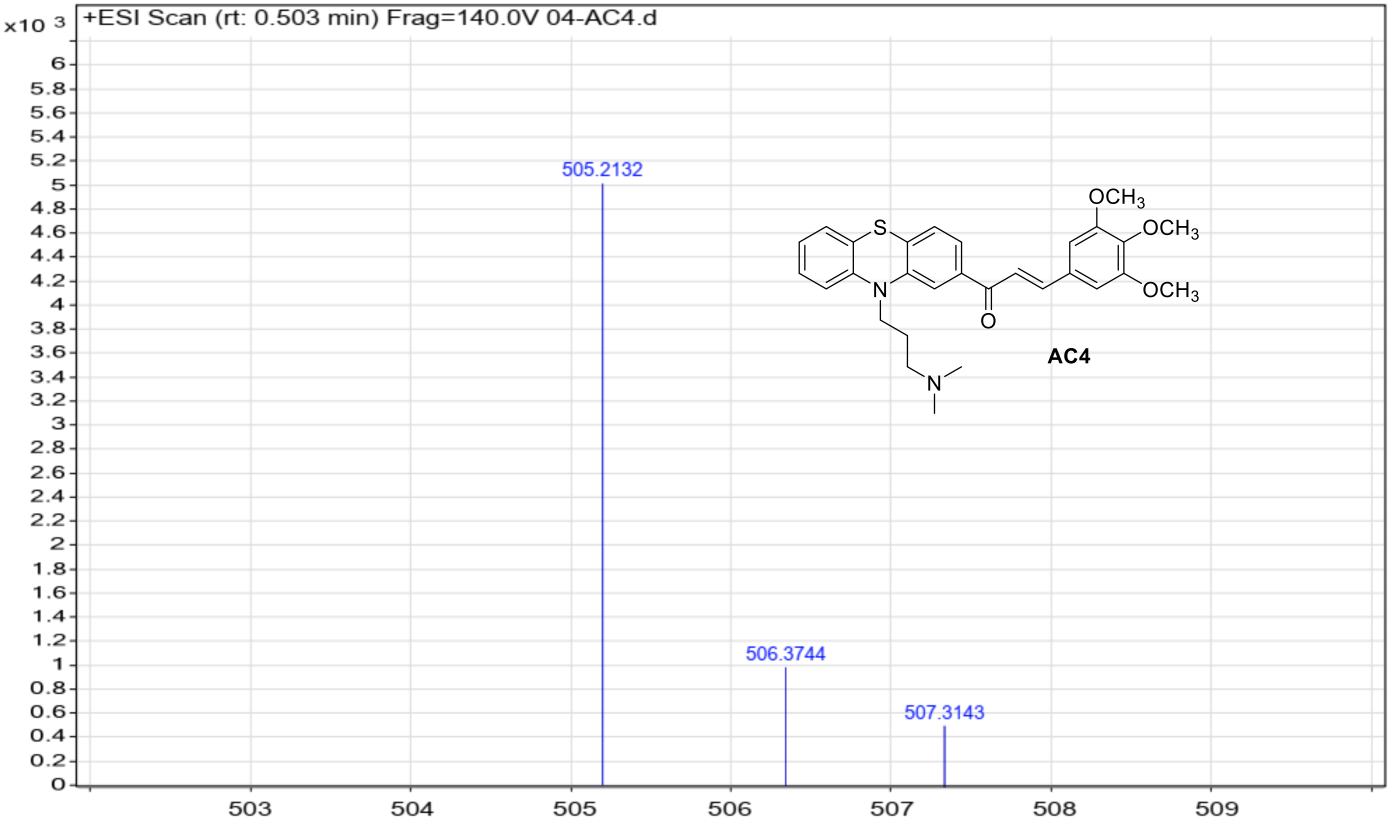

^{1}H-NMR

C4-MeOD-1H

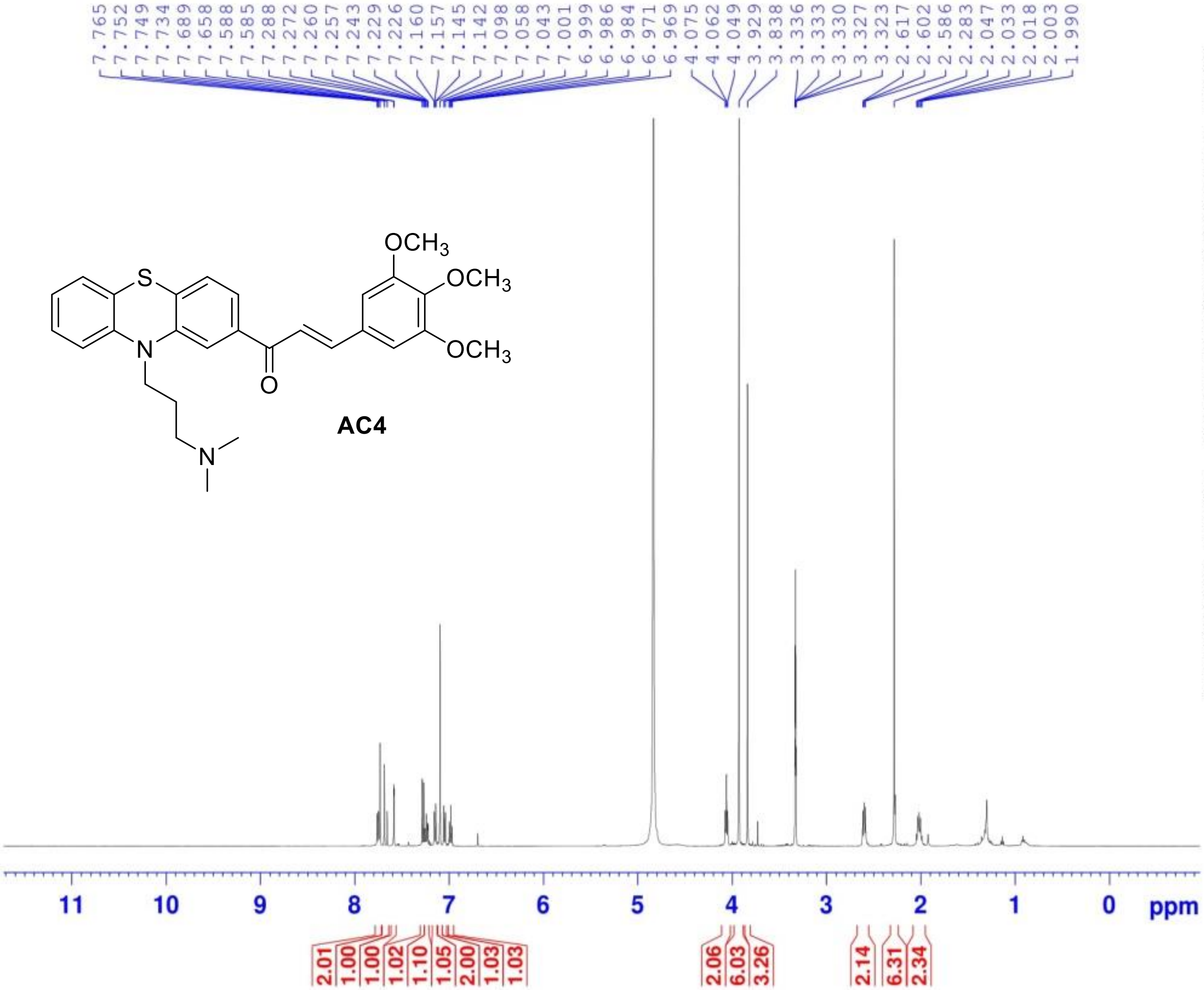

AC4

BRUKER

Current Data Parameters
NAME 113D_C4
EXPNO 1
PROCNO 1

F2 - Acquisition Parameters
Date_ 20170606
Time 16.07
INSTRUM spect
PROBHD 5 mm PABBO BB/
PULPROG zg30
TD 65536
SOLVENT MeOD
NS 16
DS 2
SWH 10000.000 Hz
FIDRES 0.152588 Hz
AQ 3.2767999 sec
RG 127.68
DW 50.000 usec
DE 6.50 usec
TE 303.0 K
D1 1.00000000 sec
TD0 1

======== CHANNEL f1 ========
SFO1 500.2030889 MHz
NUC1 1H
P1 10.00 usec
PLW1 22.00000000 W

F2 - Processing parameters
SI 65536
SF 500.2000001 MHz
WDW EM
SSB 0
LB 0.30 Hz
GB 0
PC 1.00

^{1}H-NMR

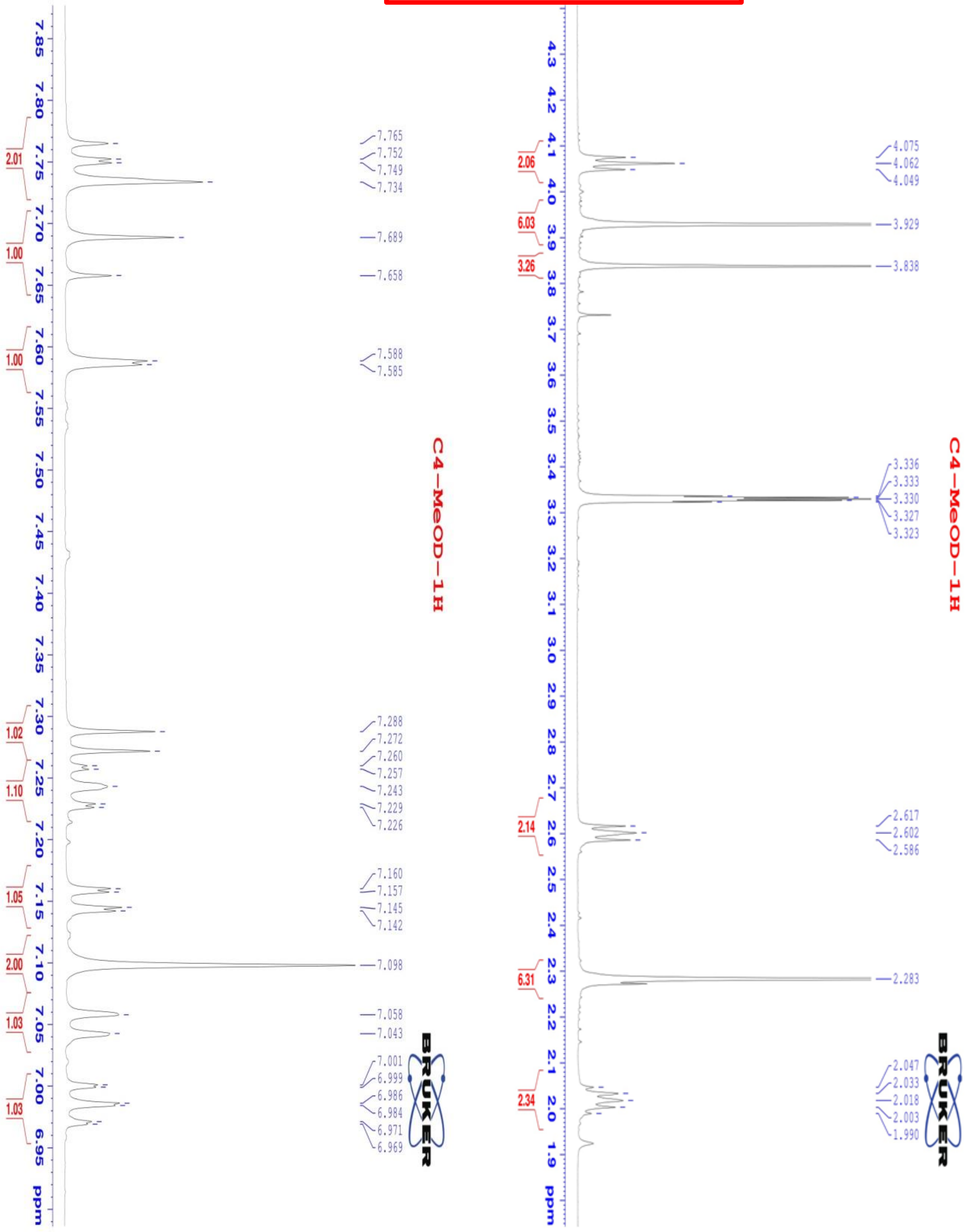

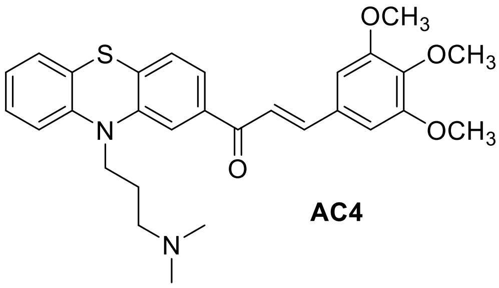

AC4

^{13}C-NMR

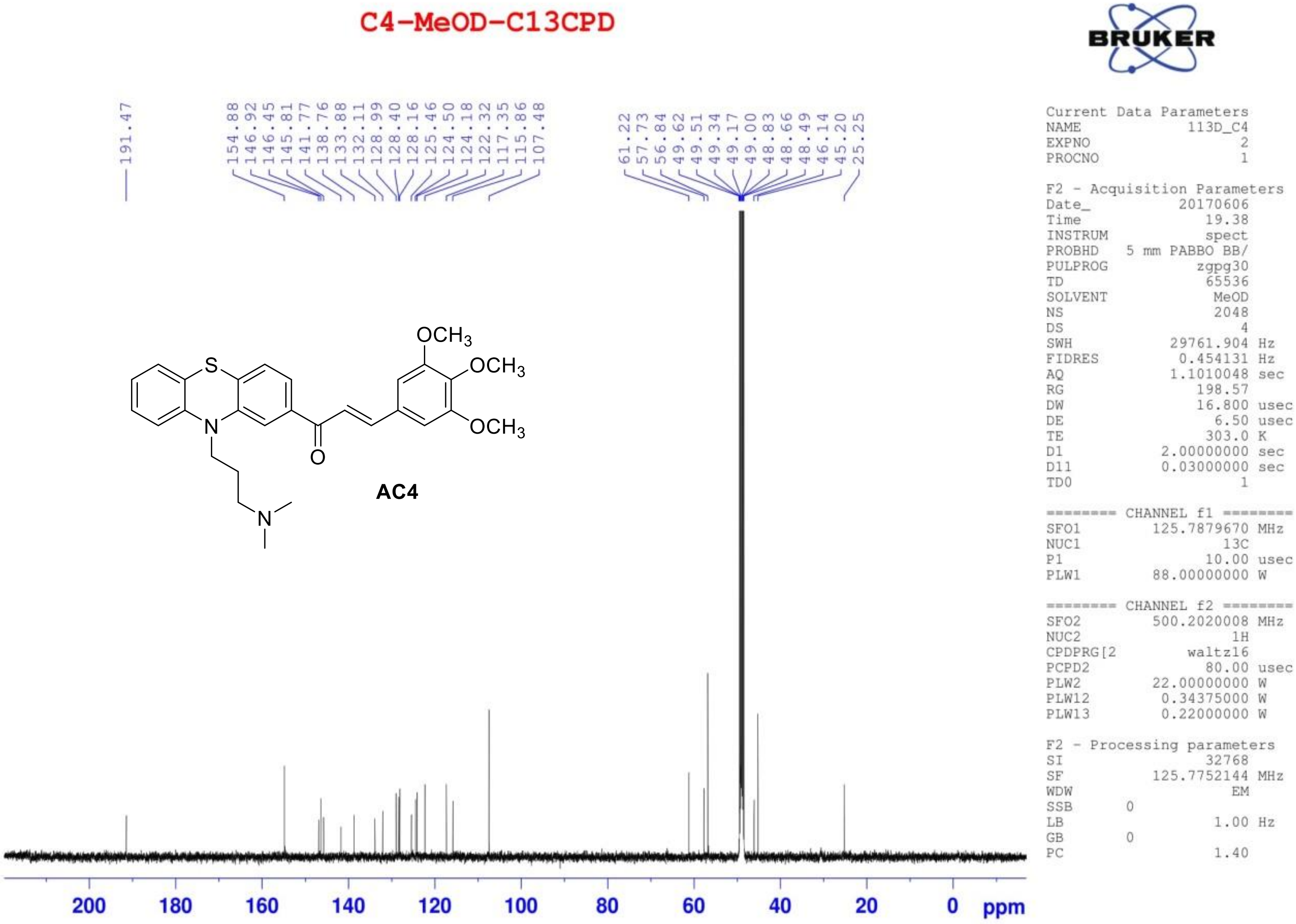
C4-MeOD-C13CPD
191.47
154.88
146.92
146.45
145.81
141.77
138.76
133.88
132.11
128.99
128.40
128.16
125.46
124.50
124.18
122.32
117.35
115.86
107.48
61.22
57.73
56.84
49.62
49.51
49.34
49.17
49.00
48.83
48.66
48.49
46.14
45.20
25.25
OCH3
OCH3
OCH3
S
N
O
N
AC4
200
180
160
140
120
100
80
60
40
20
0
ppm
Current Data Parameters
NAME 113D_C4
EXPNO 2
PROCNO 1
F2 - Acquisition Parameters
Date_ 20170606
Time 19.38
INSTRUM spect
PROBHD 5 mm PABBO BB/
PULPROG zgpg30
TD 65536
SOLVENT MeOD
NS 2048
DS 4
SWH 29761.904 Hz
FIDRES 0.454131 Hz
AQ 1.1010048 sec
RG 198.57
DW 16.800 usec
DE 6.50 usec
TE 303.0 K
D1 2.00000000 sec
D11 0.03000000 sec
TD0 1
======== CHANNEL f1 ========
SFO1 125.7879670 MHz
NUC1 13C
P1 10.00 usec
PLW1 88.00000000 W
======== CHANNEL f2 ========
SFO2 500.2020008 MHz
NUC2 1H
CPDPRG[2 waltz16
PCPD2 80.00 usec
PLW2 22.00000000 W
PLW12 0.34375000 W
PLW13 0.22000000 W
F2 - Processing parameters
SI 32768
SF 125.7752144 MHz
WDW EM
SSB 0
LB 1.00 Hz
GB 0
PC 1.40

^{13}C-NMR

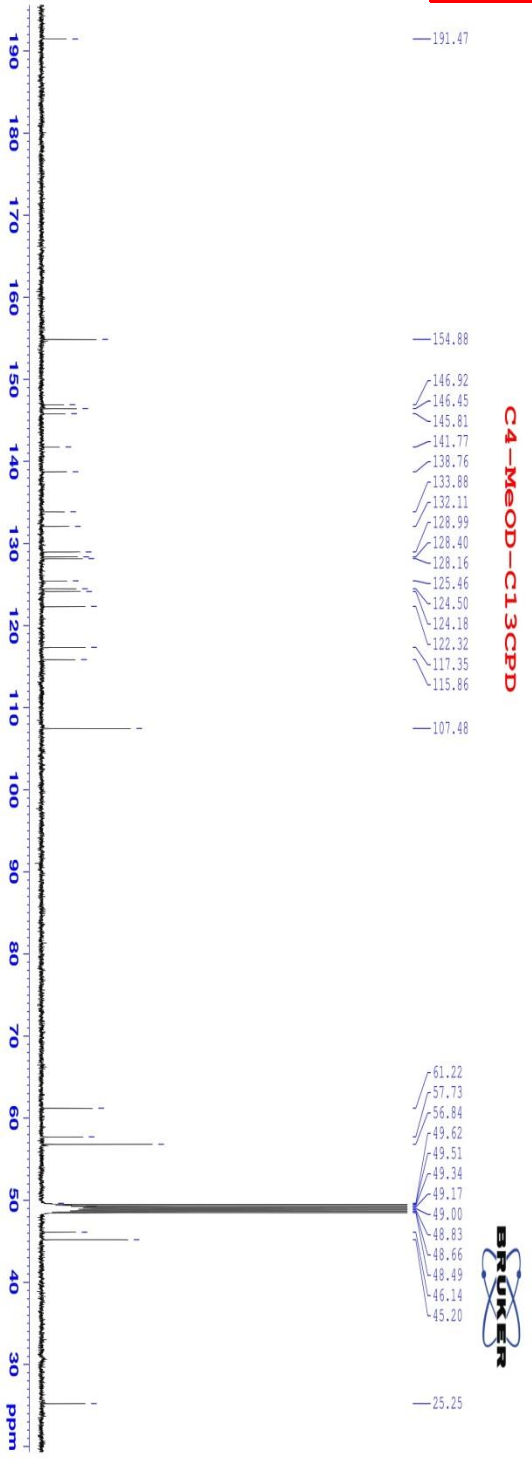
C4-MeOD-C13CPD
191.47
154.88
146.92
146.45
145.81
141.77
138.76
133.88
132.11
128.99
128.40
128.16
125.46
124.50
124.18
122.32
117.35
115.86
107.48
61.22
57.73
56.84
49.62
49.51
49.34
49.17
49.00
48.83
48.66
48.49
46.14
45.20
25.25
ppm

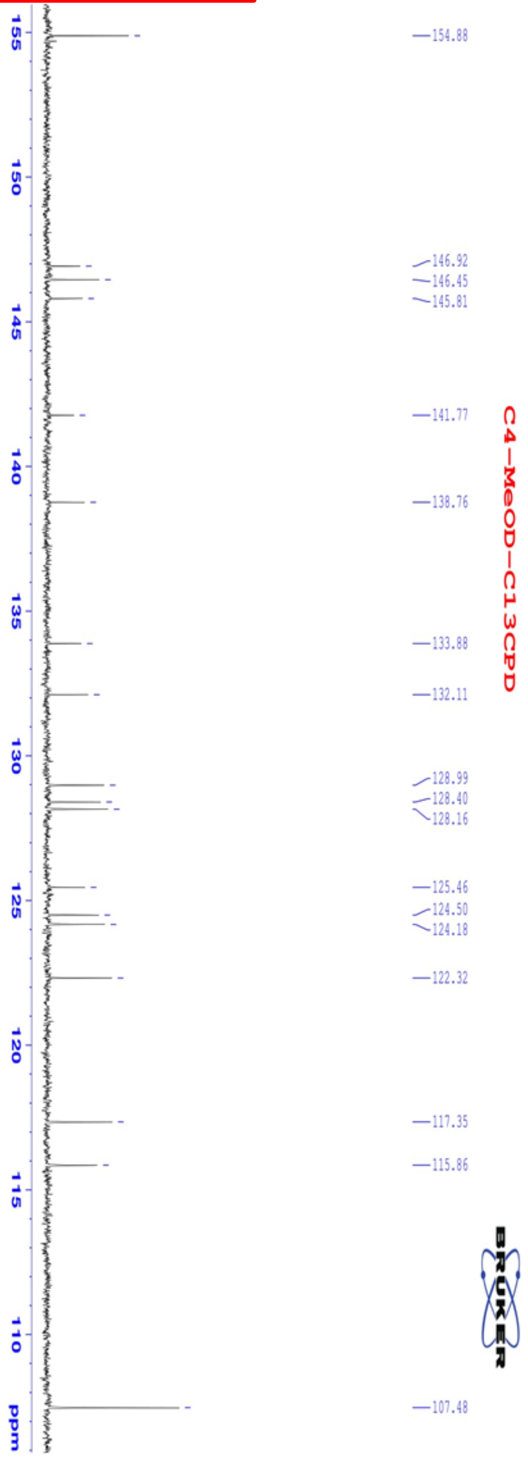
C4-MeOD-C13CPD
154.88
146.92
146.45
145.81
141.77
138.76
133.88
132.11
128.99
128.40
128.16
125.46
124.50
124.18
122.32
117.35
115.86
107.48
ppm

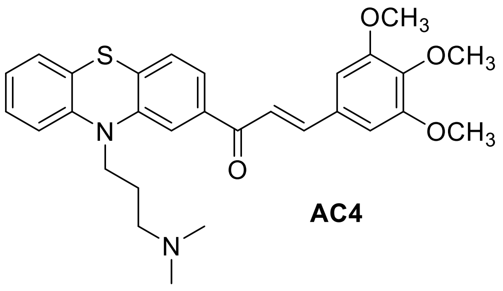
S
N
O
N
OCH_3
OCH_3
OCH_3

AC4

^{13}C-NMR

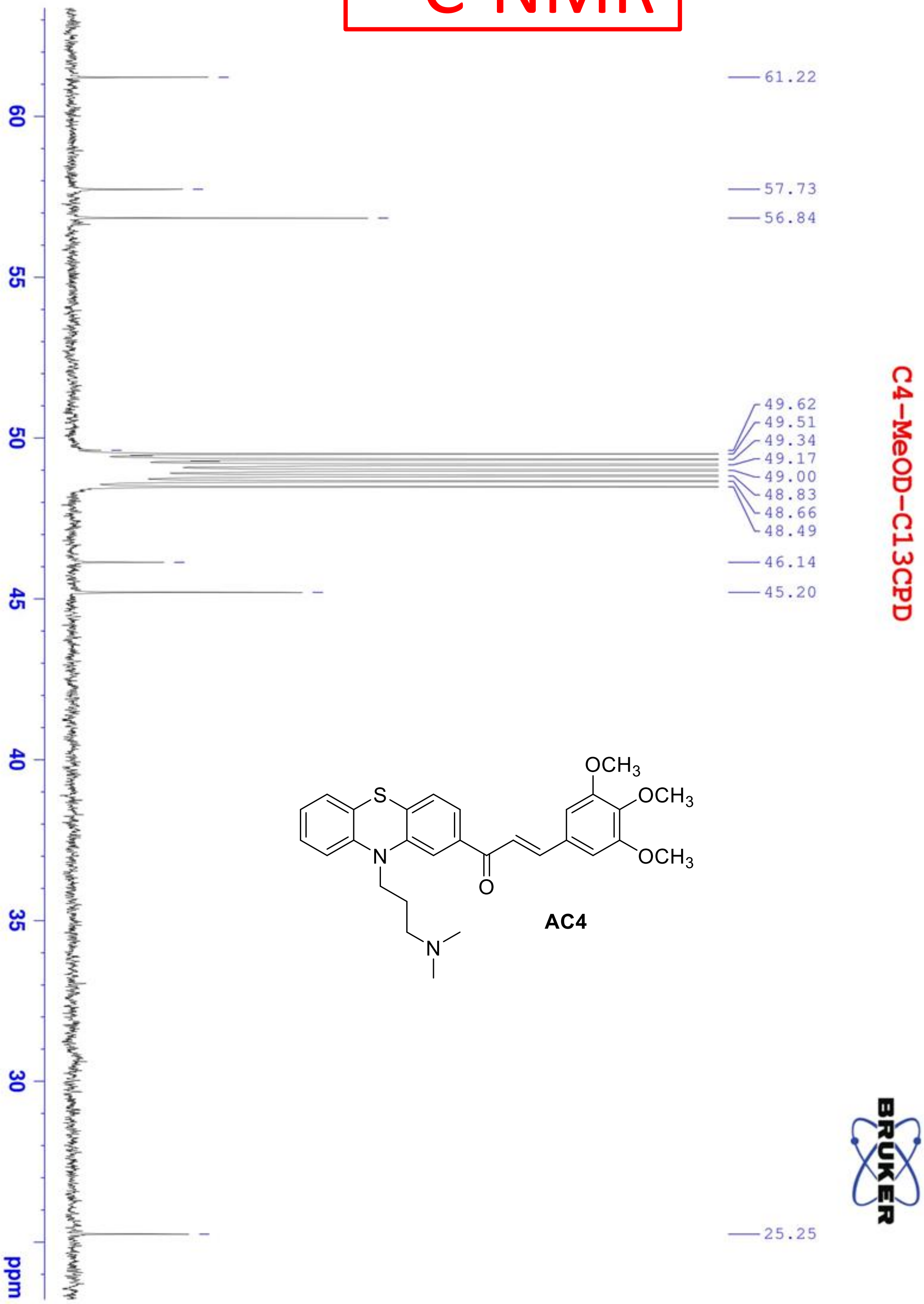
61.22
57.73
56.84
49.62
49.51
49.34
49.17
49.00
48.83
48.66
48.49
46.14
45.20
25.25
60
55
50
45
40
35
30
ppm
C4–MeOD–C13CPD
S
N
N
O
OCH3
OCH3
OCH3

AC4

IR

VIEN CONG NGHE HOA HOC
01 Mac Dinh Chi - Q.1 - Tp HCM

Tel: 08.38296127
Fax: 08.38293889

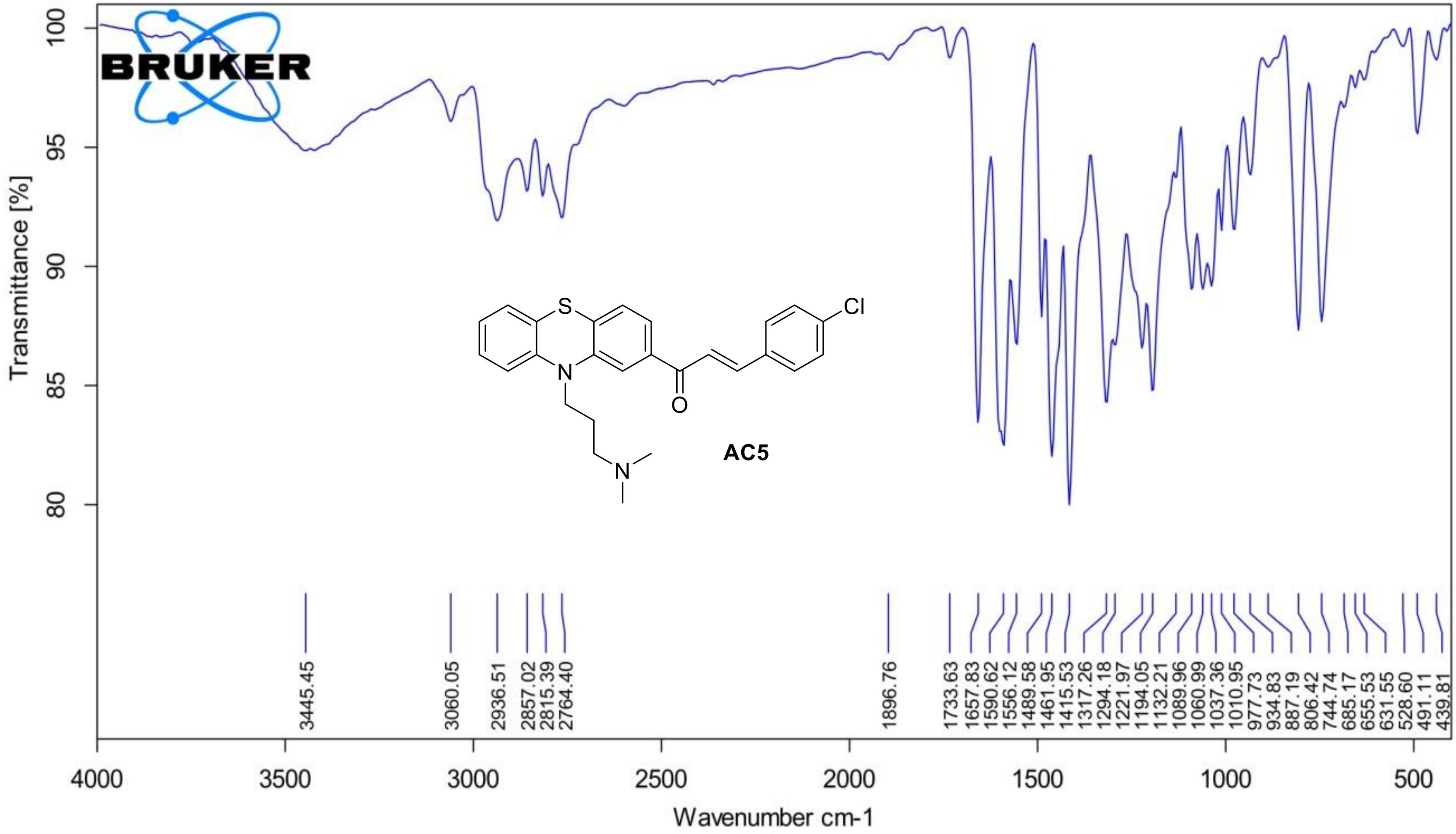

E:\OPUS 7\2017\THANG 7\20170714\C9.0	C9	TENSOR 27 - BRUKER - GERMANY	7/14/2017

MS

Qualitative Analysis Report

**Data Filename**	05-AC5.d	**Sample Name**	05-AC5
**Sample Type**	Sample	**Position**	P2-B4
**Instrument Name**	Instrument 1	**User Name**	
**Acq Method**	Cot ngan - MSMS_Pos.m	**Acquired Time**	22/08/2020 10:38:45 AM
**IRM Calibration Status**	Success	**DA Method**	COTNGAN.M.m
**Comment**			

**Sample Group**		**Info.**	
**Stream Name**	LC 1	**Acquisition SW Version**	6200 series TOF/6500 series Q-TOF B.06.01 (B6172 SP1)

User Chromatograms

Fragmentor Voltage 140 **Collision Energy** 0 **Ionization Mode** ESI

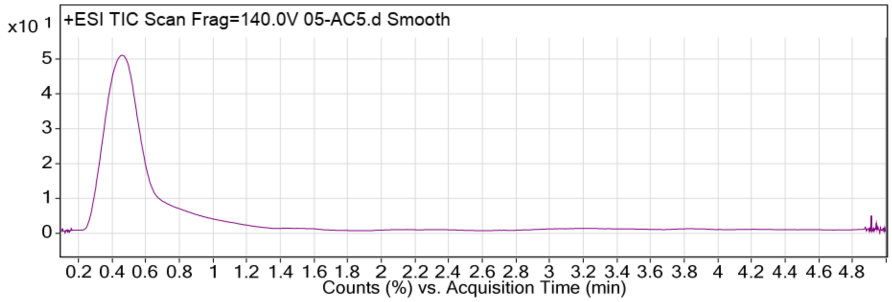

User Spectra

Fragmentor Voltage	Collision Energy	Ionization Mode
140	0	ESI

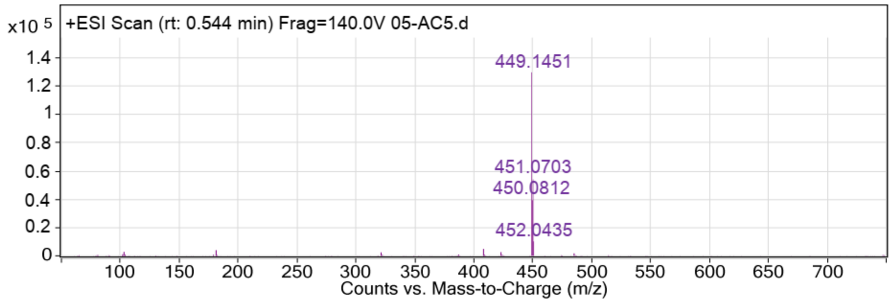

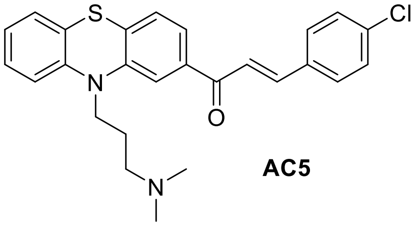

AC5

--- End Of Report ---

MS

**Sample Name**	05-AC5	**Position**	P2-B4	**Instrument Name**	Instrument 1	**User Name**	
**Inj Vol**	2	**InjPosition**		**SampleType**	Sample	**IRM Calibration Status**	Success
**Data Filename**	05-AC5.d	**ACQ Method**	Cot ngan - MSMS_Pos.	**Comment**		**Acquired Time**	22/08/2020 10:38:45 AM

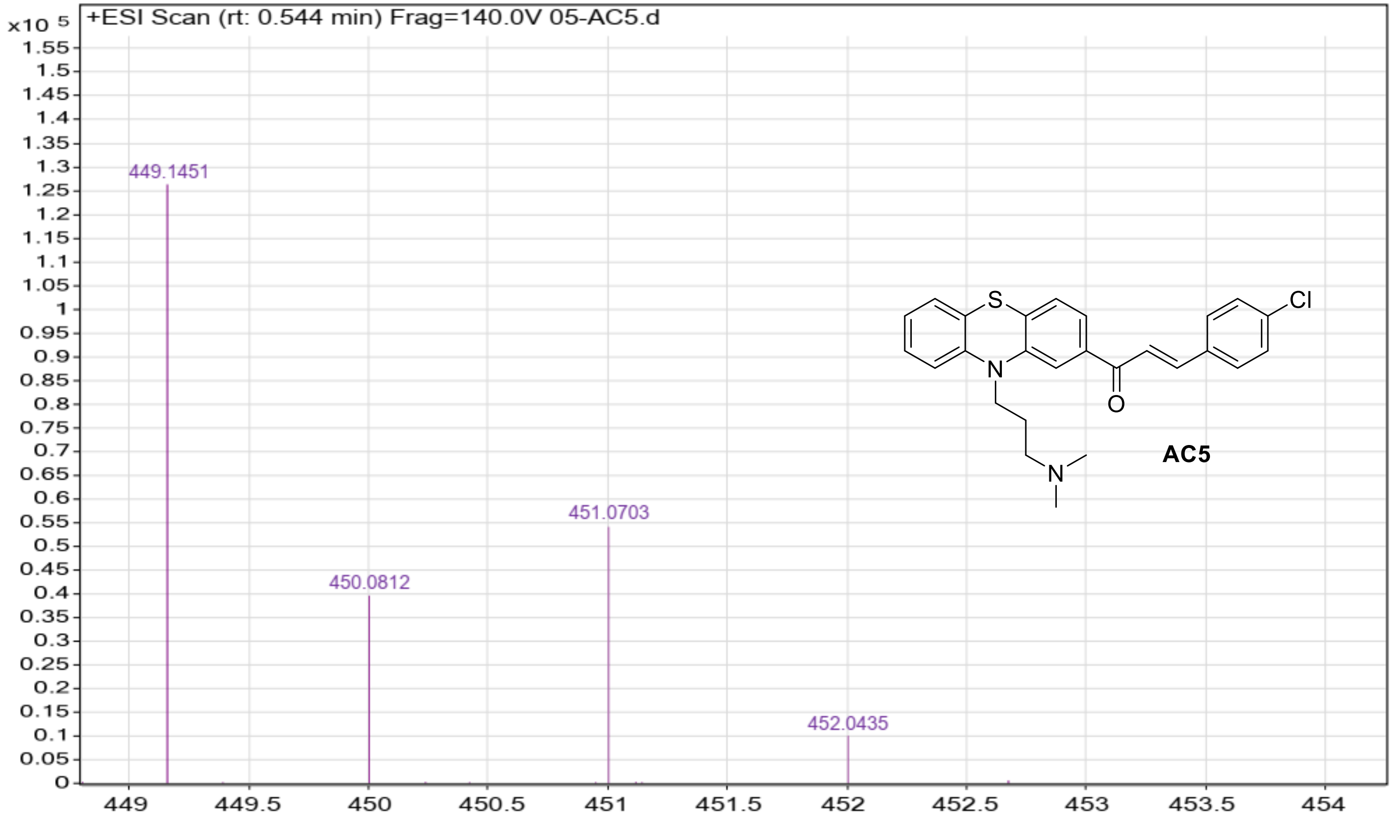

^{1}H-NMR

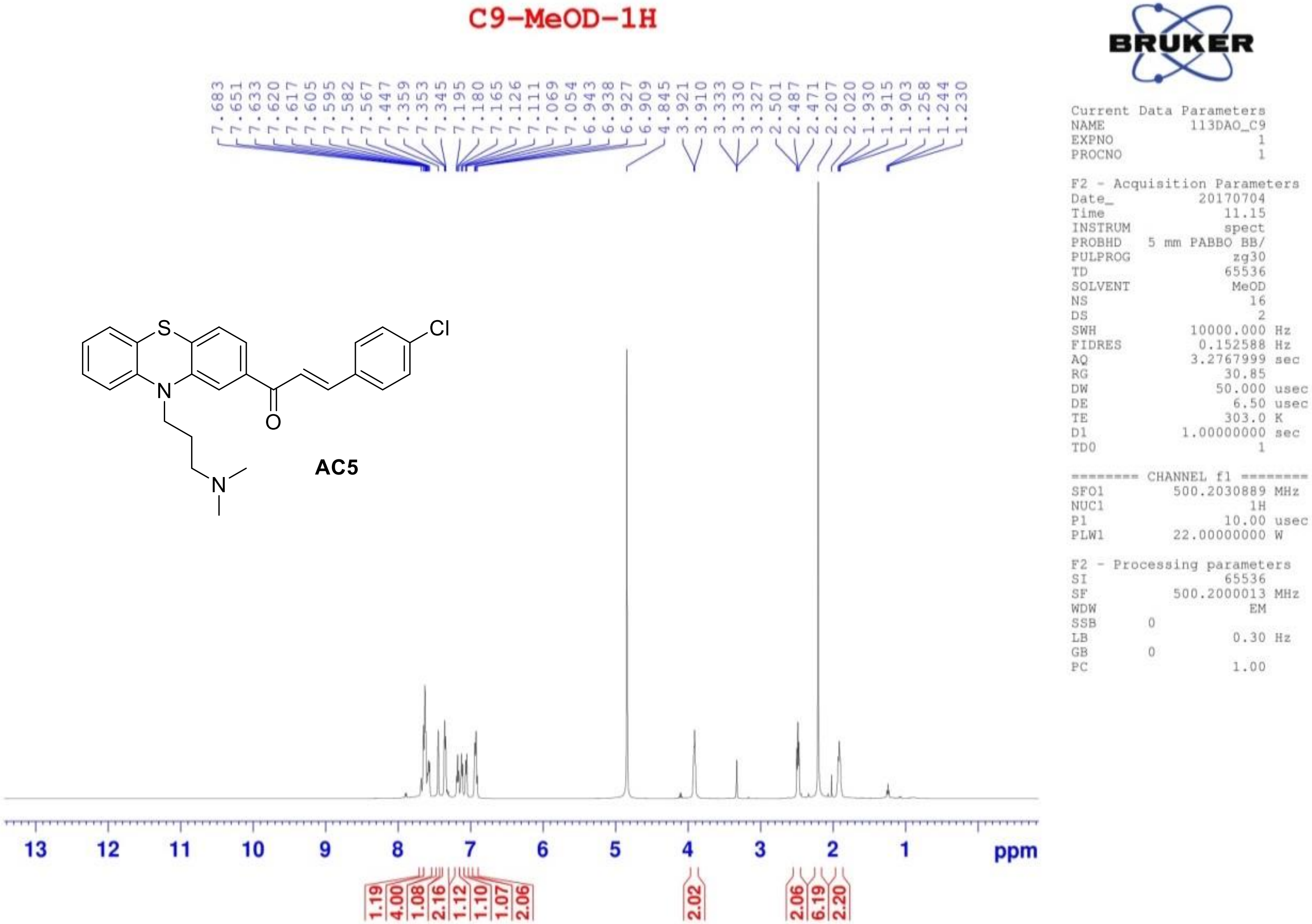

C9-MeOD-1H
7.683 7.651 7.633 7.620 7.617 7.605 7.595 7.582 7.567 7.447 7.359 7.353 7.345 7.195 7.180 7.165 7.126 7.111 7.069 7.054 6.943 6.938 6.927 6.909 4.845 3.921 3.910 3.333 3.330 3.327 2.501 2.487 2.471 2.207 2.020 1.930 1.915 1.903 1.258 1.244 1.230
S
N
O
N
Cl
AC5
13 12 11 10 9 8 7 6 5 4 3 2 1 ppm
1.19 4.00 1.08 2.16 1.12 1.10 1.07 2.06 2.02 2.06 6.19 2.20
BRUKER
Current Data Parameters
NAME 113DAO_C9
EXPNO 1
PROCNO 1
F2 - Acquisition Parameters
Date_ 20170704
Time 11.15
INSTRUM spect
PROBHD 5 mm PABBO BB/
PULPROG zg30
TD 65536
SOLVENT MeOD
NS 16
DS 2
SWH 10000.000 Hz
FIDRES 0.152588 Hz
AQ 3.2767999 sec
RG 30.85
DW 50.000 usec
DE 6.50 usec
TE 303.0 K
D1 1.00000000 sec
TD0 1
======== CHANNEL f1 ========
SFO1 500.2030889 MHz
NUC1 1H
P1 10.00 usec
PLW1 22.00000000 W
F2 - Processing parameters
SI 65536
SF 500.2000013 MHz
WDW EM
SSB 0
LB 0.30 Hz
GB 0
PC 1.00

^{1}H-NMR

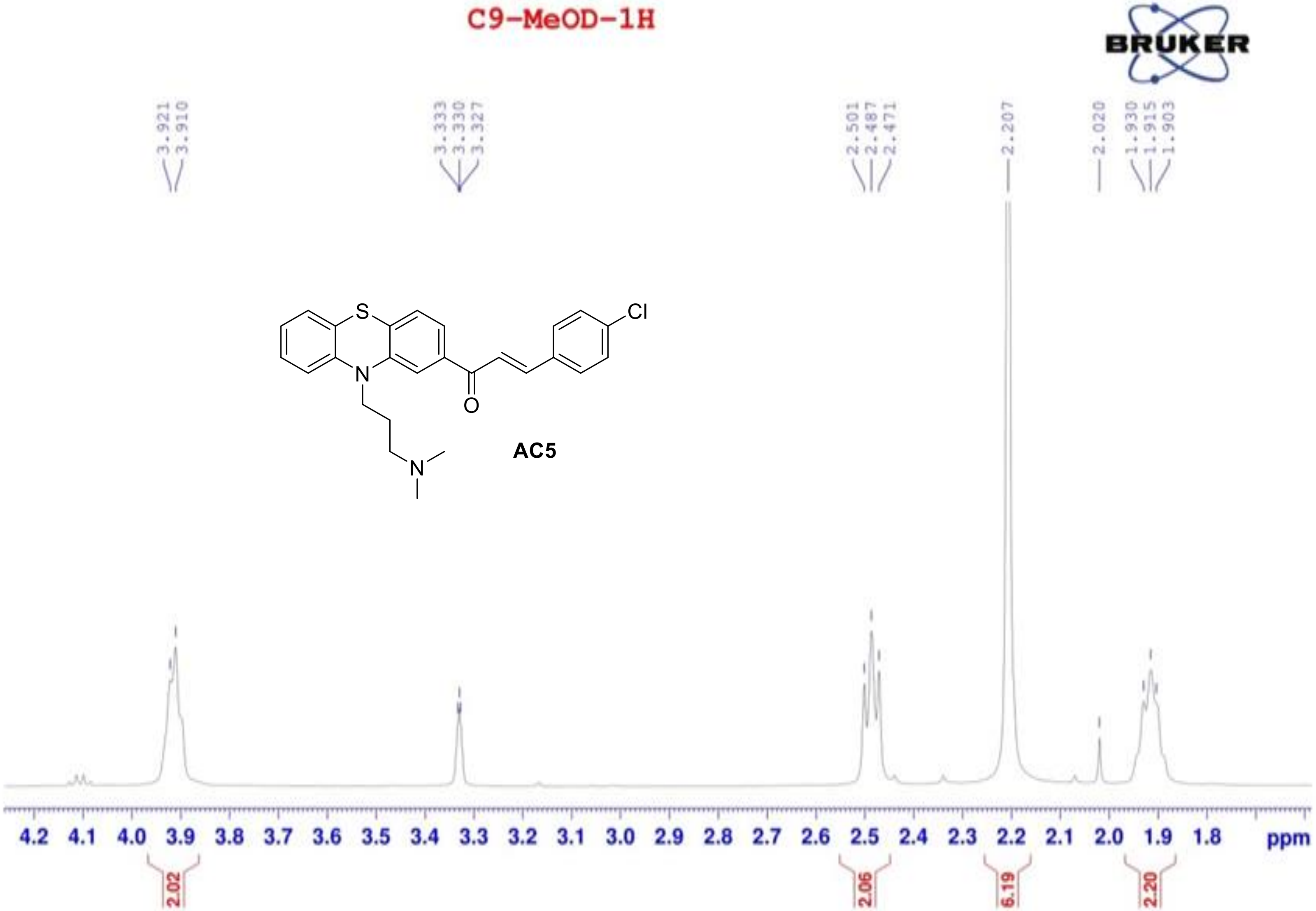
C9-MeOD-1H
BRUKER
3.921
3.910
3.333
3.330
3.327
2.501
2.487
2.471
2.207
2.020
1.930
1.915
1.903
S
N
O
Cl
N
AC5
4.2 4.1 4.0 3.9 3.8 3.7 3.6 3.5 3.4 3.3 3.2 3.1 3.0 2.9 2.8 2.7 2.6 2.5 2.4 2.3 2.2 2.1 2.0 1.9 1.8 ppm
2.02
2.06
6.19
2.20

^{1}H-NMR

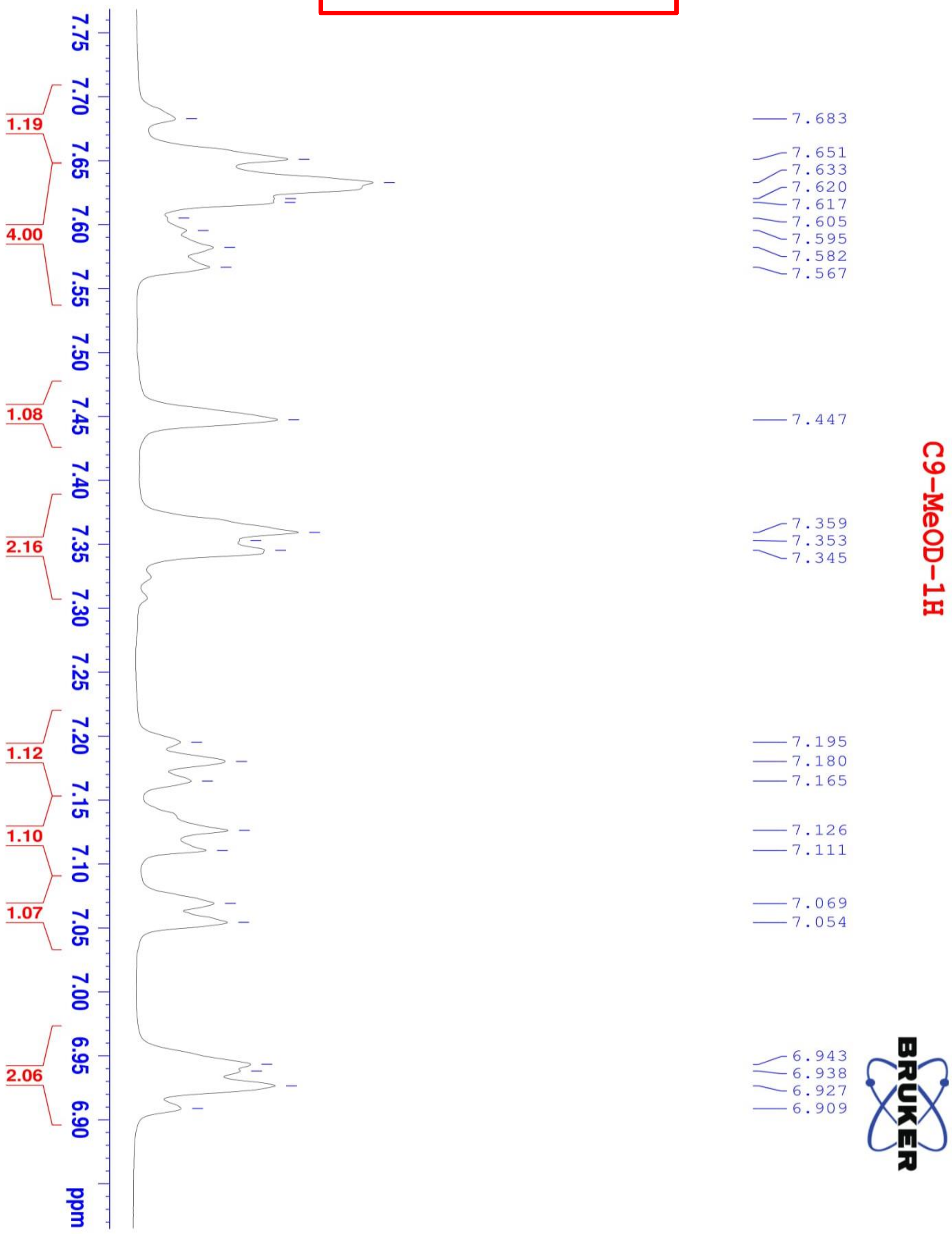
C9-MeOD-1H
7.683
7.651
7.633
7.620
7.617
7.605
7.595
7.582
7.567
7.447
7.359
7.353
7.345
7.195
7.180
7.165
7.126
7.111
7.069
7.054
6.943
6.938
6.927
6.909
1.19
4.00
1.08
2.16
1.12
1.10
1.07
2.06
ppm

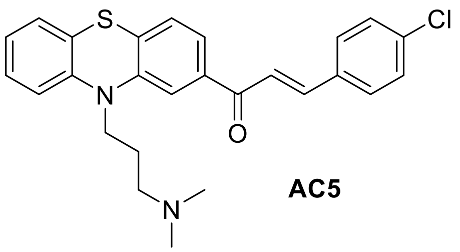
S
N
O
Cl
N

AC5

^{13}C-NMR

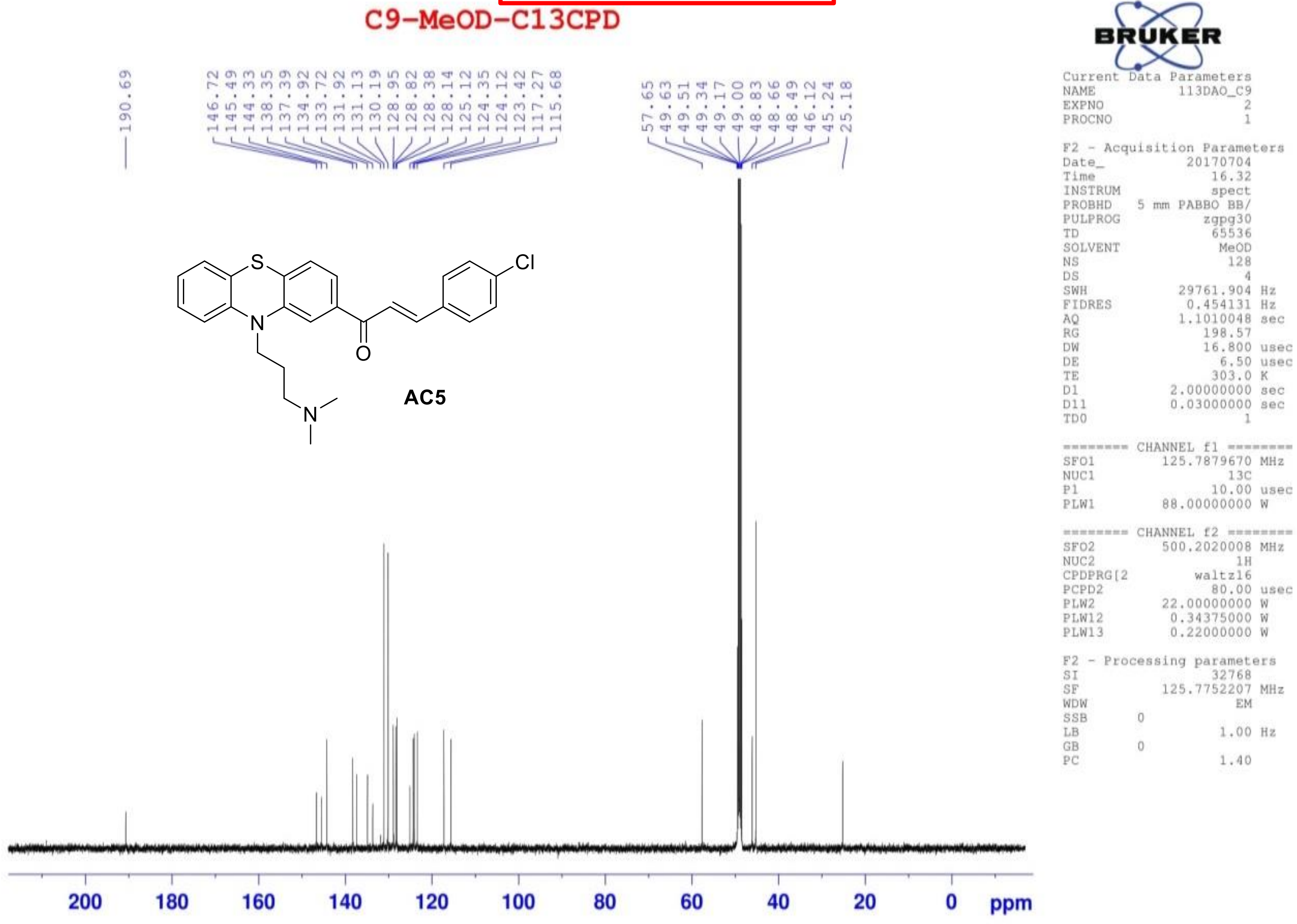
C9-MeOD-C13CPD
190.69
146.72
145.49
144.33
138.35
137.39
134.92
133.72
131.92
131.13
130.19
128.95
128.82
128.38
128.14
125.12
124.35
124.12
123.42
117.27
115.68
57.65
49.63
49.51
49.34
49.17
49.00
48.83
48.66
48.49
46.12
45.24
25.18
S
N
O
Cl
N
AC5
200
180
160
140
120
100
80
60
40
20
0
ppm
BRUKER
Current Data Parameters
NAME 113DAO_C9
EXPNO 2
PROCNO 1
F2 - Acquisition Parameters
Date_ 20170704
Time 16.32
INSTRUM spect
PROBHD 5 mm PABBO BB/
PULPROG zgpg30
TD 65536
SOLVENT MeOD
NS 128
DS 4
SWH 29761.904 Hz
FIDRES 0.454131 Hz
AQ 1.1010048 sec
RG 198.57
DW 16.800 usec
DE 6.50 usec
TE 303.0 K
D1 2.00000000 sec
D11 0.03000000 sec
TD0 1
======== CHANNEL f1 ========
SFO1 125.7879670 MHz
NUC1 13C
P1 10.00 usec
PLW1 88.00000000 W
======== CHANNEL f2 ========
SFO2 500.2020008 MHz
NUC2 1H
CPDPRG[2 waltz16
PCPD2 80.00 usec
PLW2 22.00000000 W
PLW12 0.34375000 W
PLW13 0.22000000 W
F2 - Processing parameters
SI 32768
SF 125.7752207 MHz
WDW EM
SSB 0
LB 1.00 Hz
GB 0
PC 1.40

^{13}C-NMR

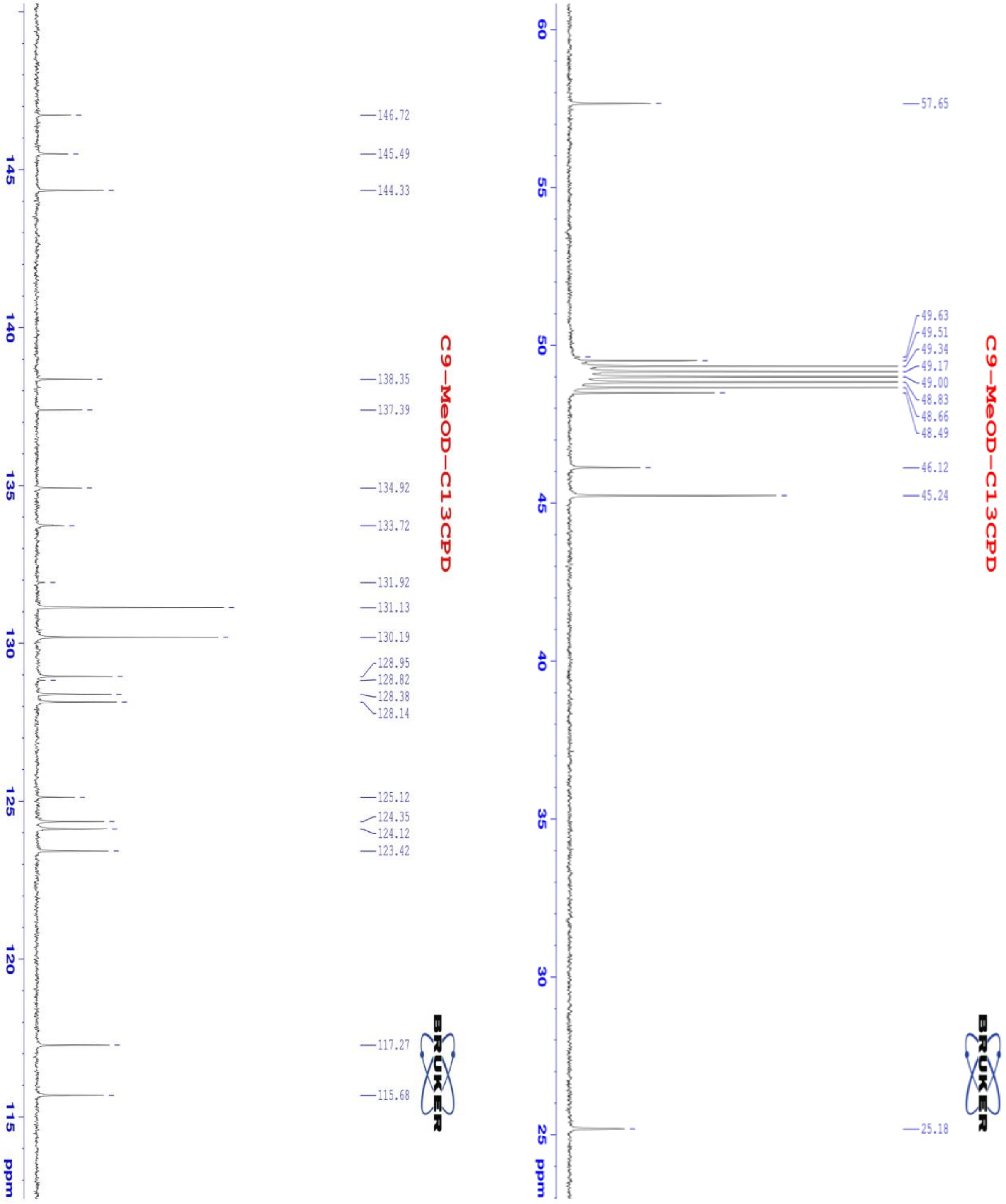
146.72
145.49
144.33
138.35
137.39
134.92
133.72
131.92
131.13
130.19
128.95
128.82
128.38
128.14
125.12
124.35
124.12
123.42
117.27
115.68
C9-MeOD-C13CPD
ppm
57.65
49.63
49.51
49.34
49.17
49.00
48.83
48.66
48.49
46.12
45.24
25.18
C9-MeOD-C13CPD
ppm

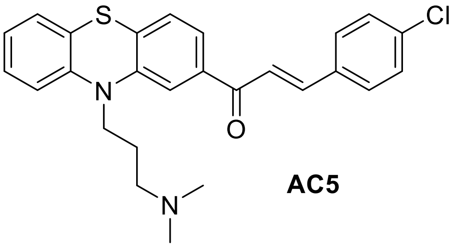
S
N
O
Cl
N

AC5

VIEN CONG NGHE HOA HOC
01 Mac Dinh Chi - Q.1 - Tp HCM

IR

Tel: 08.38296127
Fax: 08.38293889

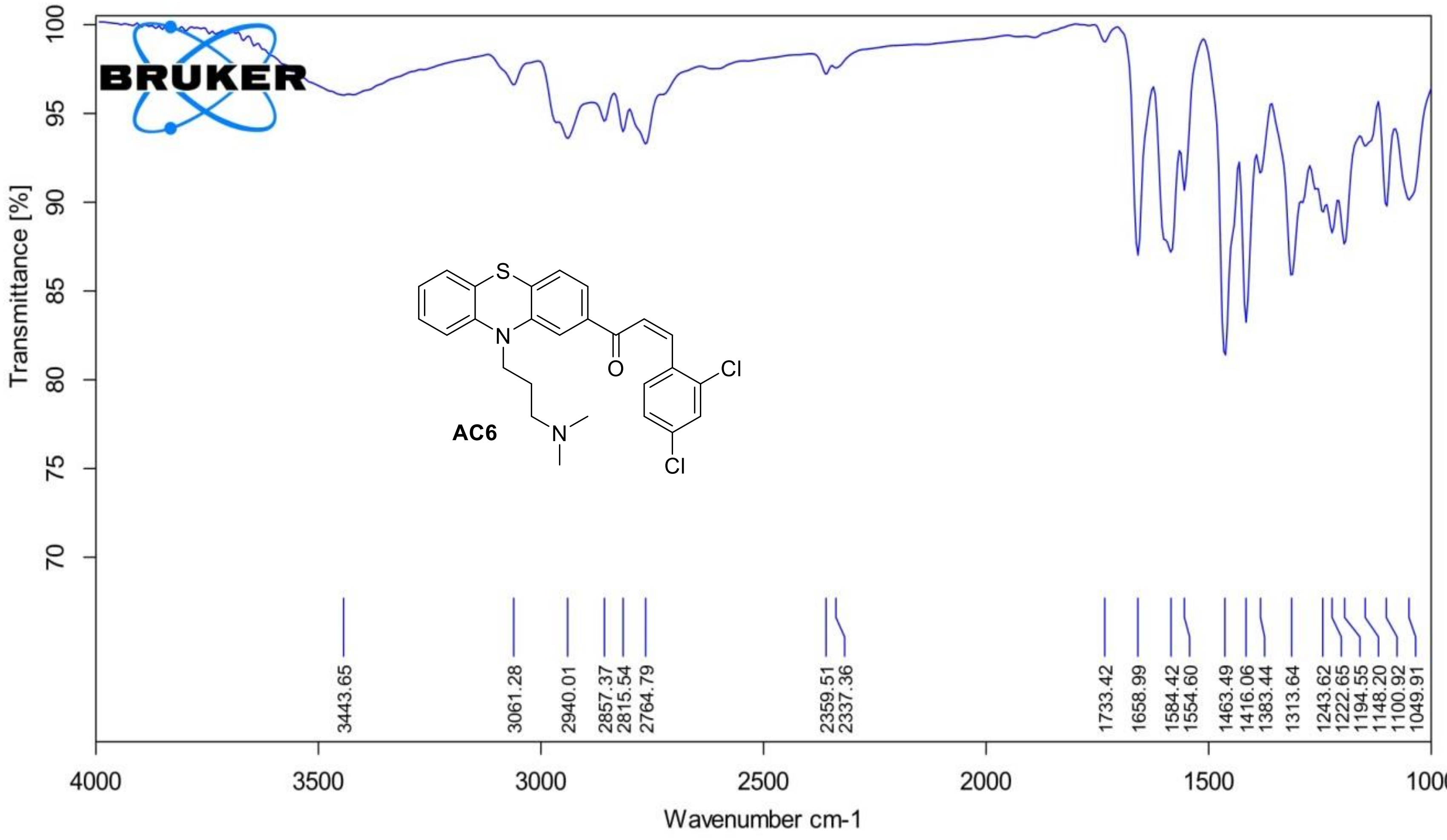

E:\OPUS 7\2017\THANG 6\20170606\C8.0	C8	TENSOR 27 - BRUKER - GERMANY	6/8/2017

MS

Qualitative Analysis Report

**Data Filename**	06-AC6.d	**Sample Name**	06-AC6
**Sample Type**	Sample	**Position**	P2-B3
**Instrument Name**	Instrument 1	**User Name**	
**Acq Method**	Cot ngan - MSMS_Pos.m	**Acquired Time**	22/08/2020 10:43:11 AM
**IRM Calibration Status**	Success	**DA Method**	COTNGAN.M.m
**Comment**			

**Sample Group**		**Info.**	
**Stream Name**	LC 1	**Acquisition SW Version**	6200 series TOF/6500 series Q-TOF B.06.01 (B6172 SP1)

User Chromatograms

Fragmentor Voltage 140 **Collision Energy** 0 **Ionization Mode** ESI

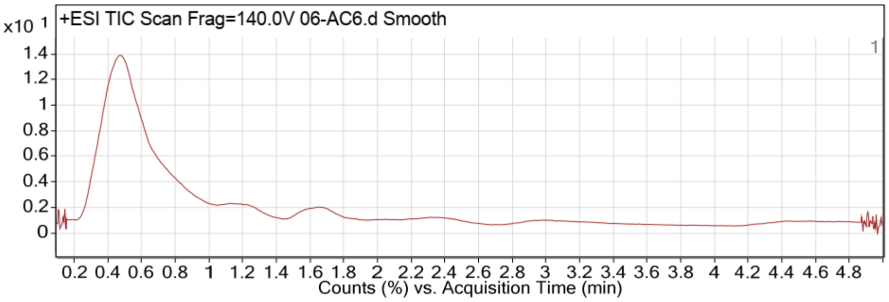

User Spectra

Fragmentor Voltage	Collision Energy	Ionization Mode
140	0	ESI

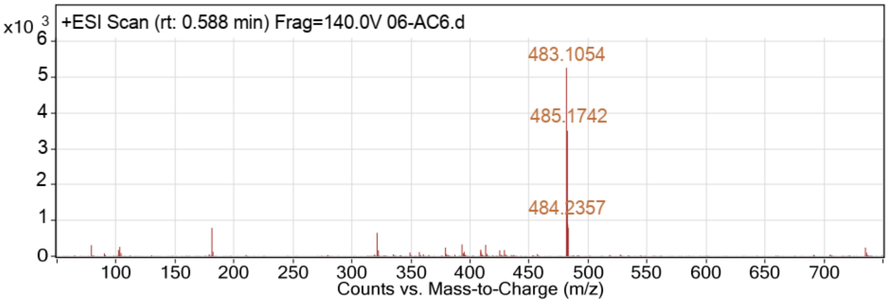

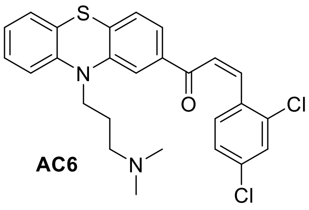

AC6

--- End Of Report ---

MS

**Sample Name**	06-AC6	**Position**	P2-B3	**Instrument Name**	Instrument 1	**User Name**	
**Inj Vol**	2	**InjPosition**		**SampleType**	Sample	**IRM Calibration Status**	Success
**Data Filename**	06-AC6.d	**ACQ Method**	Cot ngan - MSMS_Pos.	**Comment**		**Acquired Time**	22/08/2020 10:43:11 AM

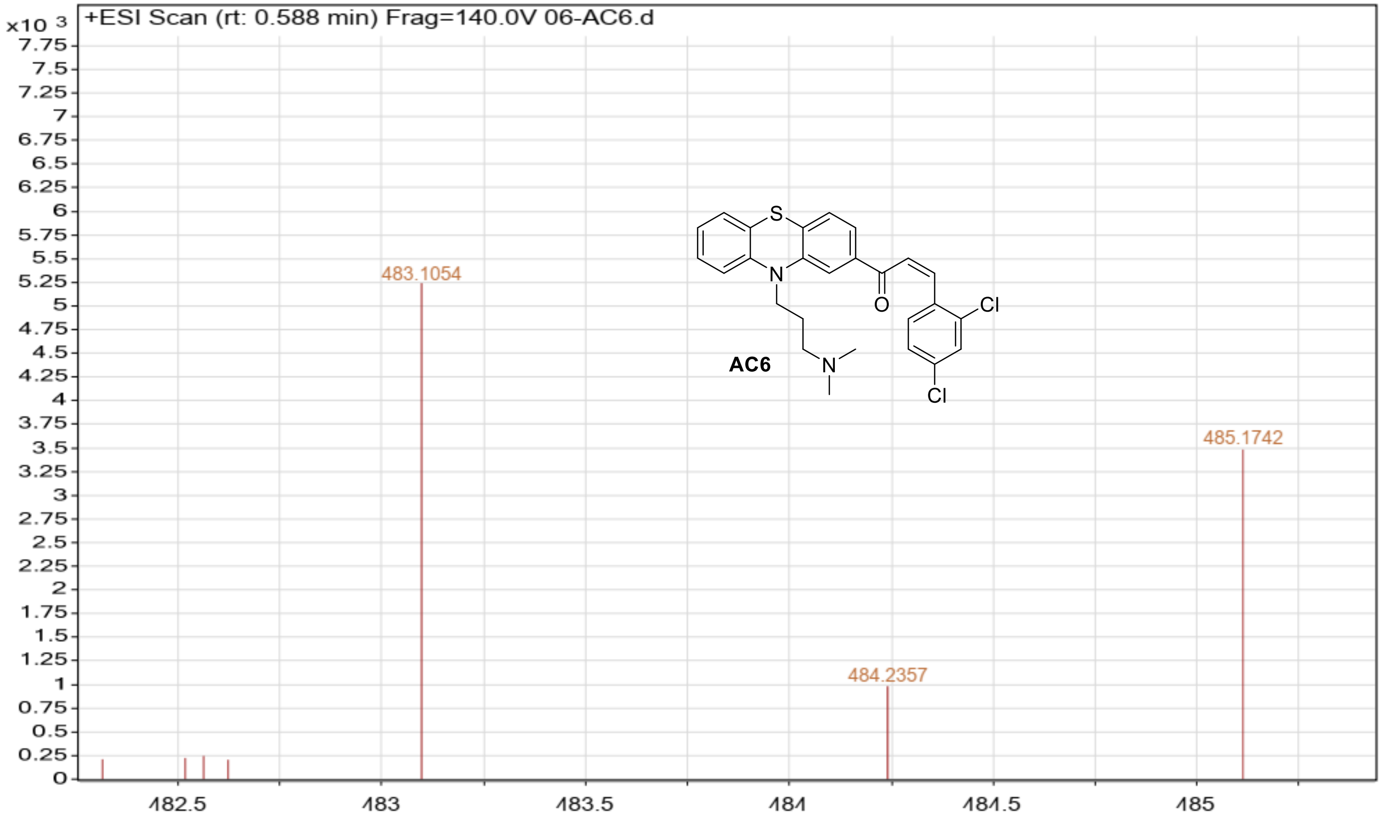

^{1}H-NMR

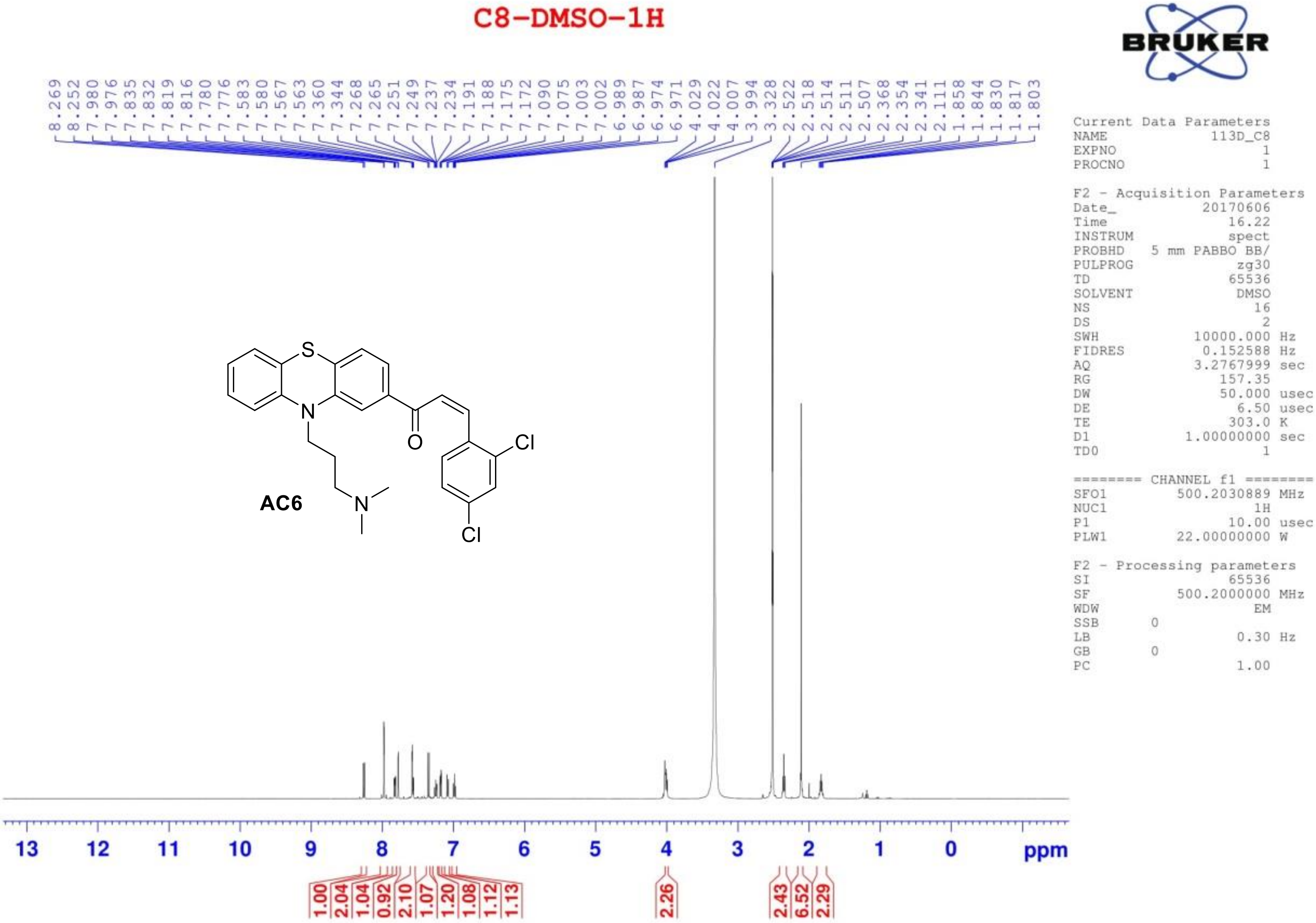
C8-DMSO-1H
BRUKER
8.269 8.252 7.980 7.976 7.835 7.832 7.819 7.816 7.780 7.776 7.583 7.580 7.567 7.563 7.360 7.344 7.268 7.265 7.251 7.249 7.237 7.234 7.191 7.188 7.175 7.172 7.090 7.075 7.003 7.002 6.989 6.987 6.974 6.971 4.029 4.022 4.007 3.994 3.328 2.522 2.518 2.514 2.511 2.507 2.368 2.354 2.341 2.111 1.858 1.844 1.830 1.817 1.803
S
N
O
Cl
Cl
N
AC6
13 12 11 10 9 8 7 6 5 4 3 2 1 0 ppm
1.00 2.04 1.04 0.92 2.10 1.07 1.20 1.08 1.12 1.13 2.26 2.43 6.52 2.29
Current Data Parameters
NAME 113D_C8
EXPNO 1
PROCNO 1
F2 - Acquisition Parameters
Date_ 20170606
Time 16.22
INSTRUM spect
PROBHD 5 mm PABBO BB/
PULPROG zg30
TD 65536
SOLVENT DMSO
NS 16
DS 2
SWH 10000.000 Hz
FIDRES 0.152588 Hz
AQ 3.2767999 sec
RG 157.35
DW 50.000 usec
DE 6.50 usec
TE 303.0 K
D1 1.00000000 sec
TD0 1
======== CHANNEL f1 ========
SFO1 500.2030889 MHz
NUC1 1H
P1 10.00 usec
PLW1 22.00000000 W
F2 - Processing parameters
SI 65536
SF 500.2000000 MHz
WDW EM
SSB 0
LB 0.30 Hz
GB 0
PC 1.00

^{1}H-NMR

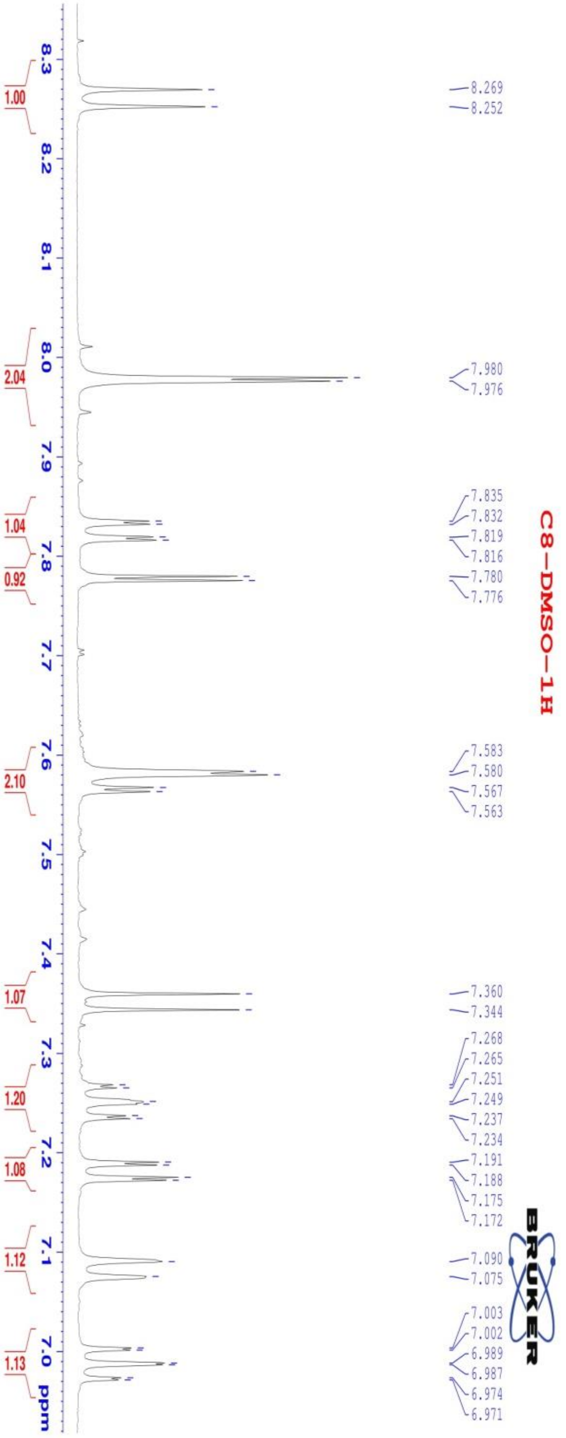

1.00
2.04
1.04
0.92
2.10
1.07
1.20
1.08
1.12
1.13
8.269
8.252
7.980
7.976
7.835
7.832
7.819
7.816
7.780
7.776
7.583
7.580
7.567
7.563
7.360
7.344
7.268
7.265
7.251
7.249
7.237
7.234
7.191
7.188
7.175
7.172
7.090
7.075
7.003
7.002
6.989
6.987
6.974
6.971
C8-DMSO-1H
ppm

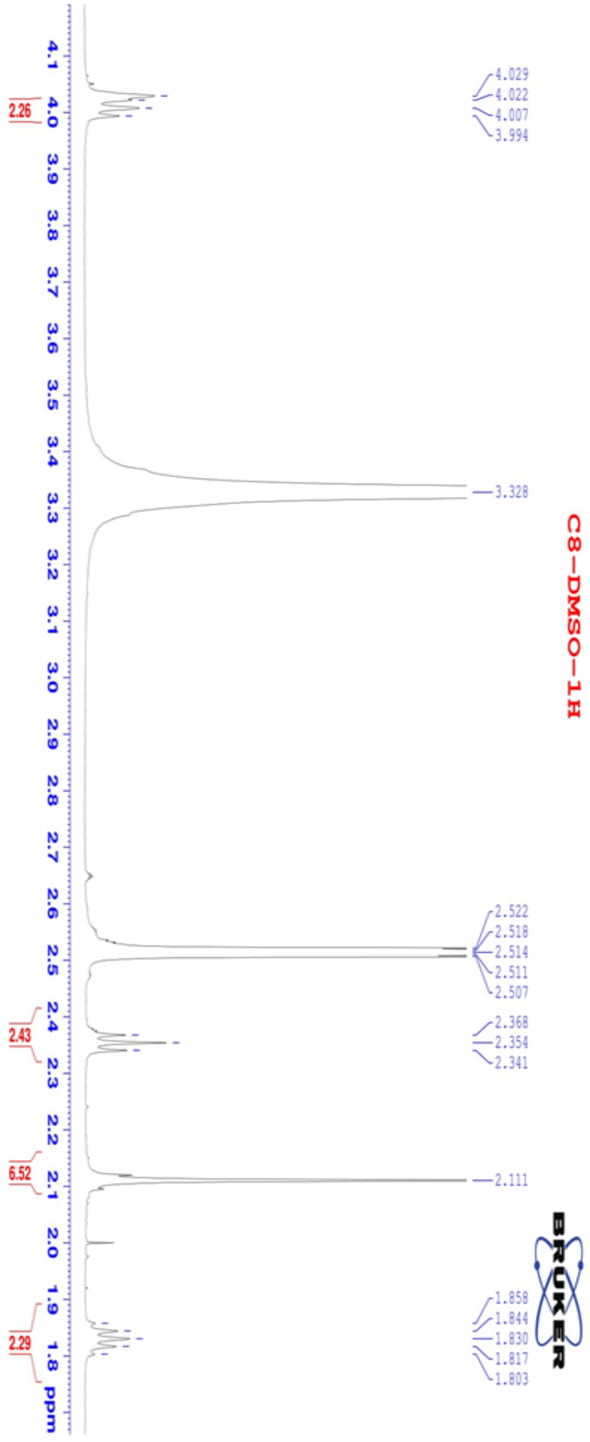

2.26
2.43
6.52
2.29
4.029
4.022
4.007
3.994
3.328
2.522
2.518
2.514
2.511
2.507
2.368
2.354
2.341
2.111
1.858
1.844
1.830
1.817
1.803
C8-DMSO-1H
ppm

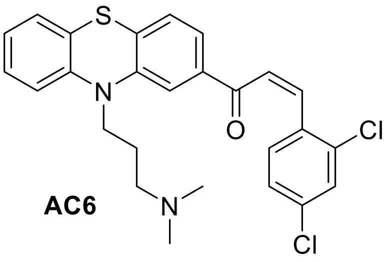

S
N
O
Cl
Cl
N

AC6

^{13}C-NMR

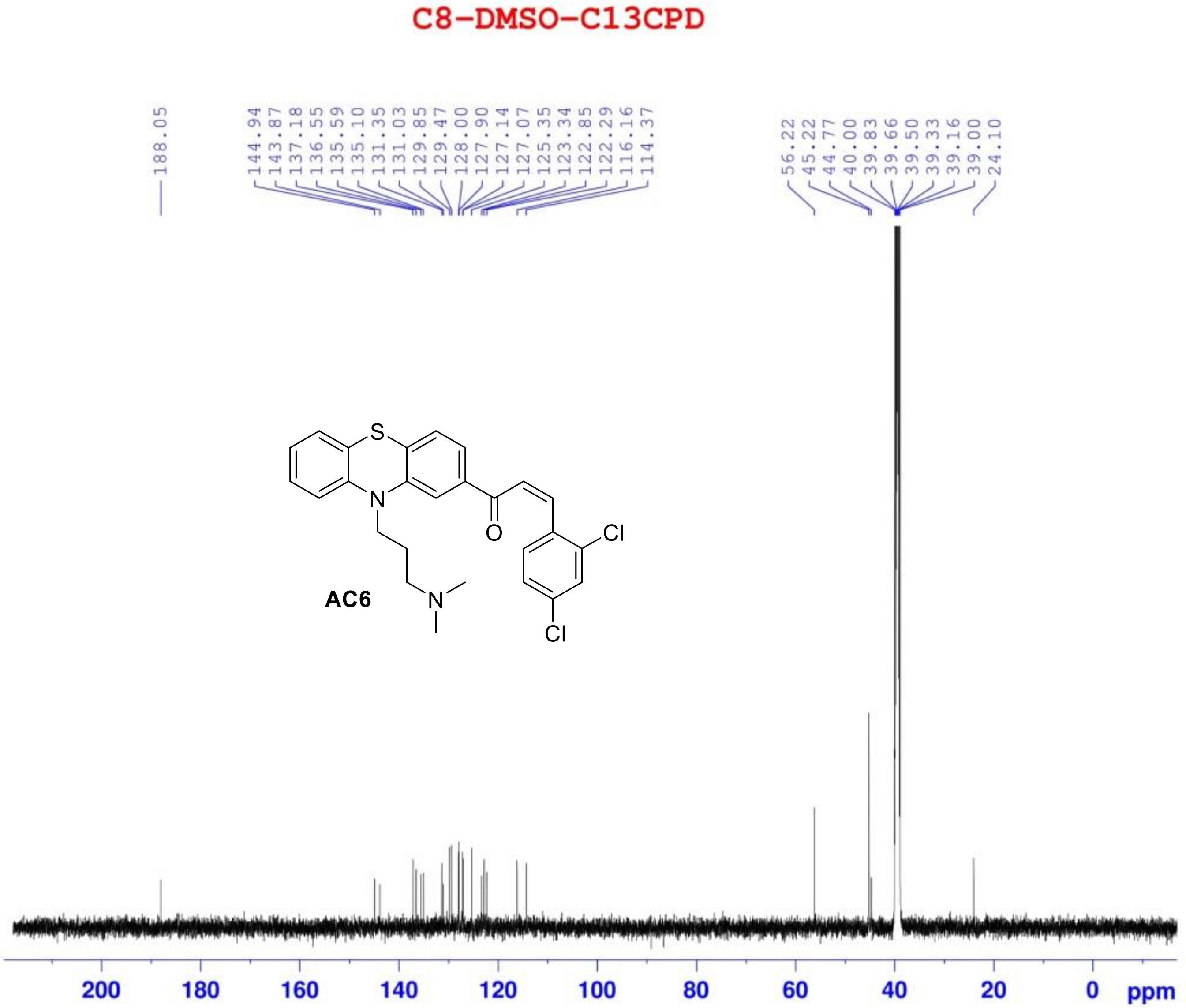

Current Data Parameters
NAME 113D_C8
EXPNO 2
PROCNO 1

F2 - Acquisition Parameters
Date_ 20170607
Time 4.54
INSTRUM spect
PROBHD 5 mm PABBO BB/
PULPROG zgpg30
TD 65536
SOLVENT DMSO
NS 4096
DS 4
SWH 29761.904 Hz
FIDRES 0.454131 Hz
AQ 1.1010048 sec
RG 198.57
DW 16.800 usec
DE 6.50 usec
TE 303.0 K
D1 2.00000000 sec
D11 0.03000000 sec
TD0 1

======== CHANNEL f1 ========
SFO1 125.7879670 MHz
NUC1 13C
P1 10.00 usec
PLW1 88.00000000 W

======== CHANNEL f2 ========
SFO2 500.2020008 MHz
NUC2 1H
CPDPRG[2 waltz16
PCPD2 80.00 usec
PLW2 22.00000000 W
PLW12 0.34375000 W
PLW13 0.22000000 W

F2 - Processing parameters
SI 32768
SF 125.7754565 MHz
WDW EM
SSB 0
LB 1.00 Hz
GB 0
PC 1.40

^{13}C-NMR

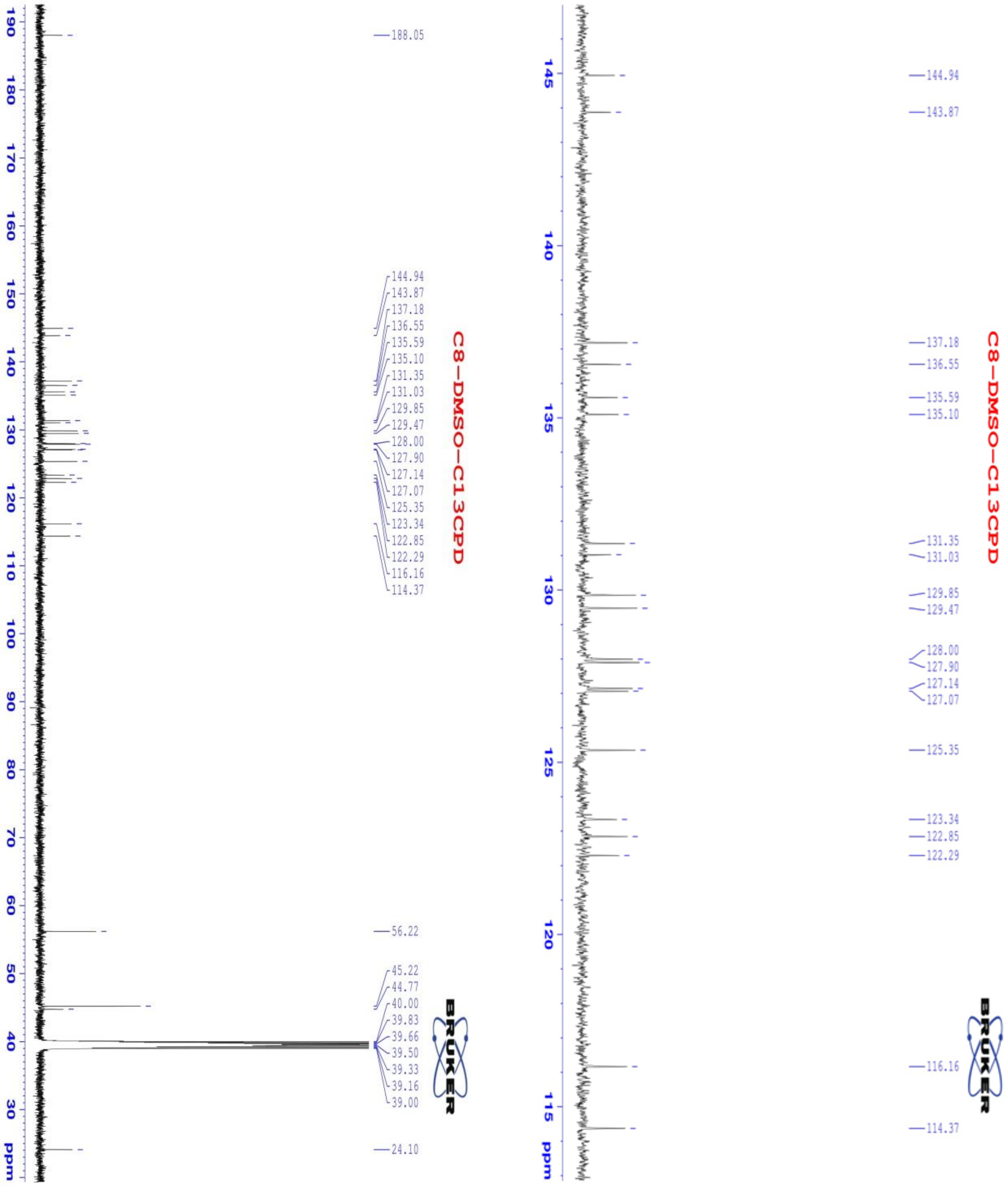
C8-DMSO-C13CPD
188.05
144.94
143.87
137.18
136.55
135.59
135.10
131.35
131.03
129.85
129.47
128.00
127.90
127.14
127.07
125.35
123.34
122.85
122.29
116.16
114.37
56.22
45.22
44.77
40.00
39.83
39.66
39.50
39.33
39.16
39.00
24.10
ppm

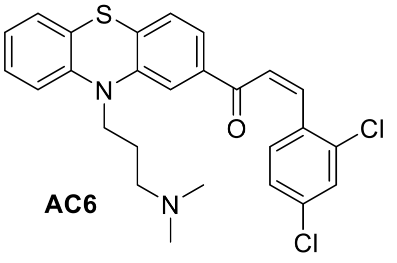

AC6

^{13}C-NMR

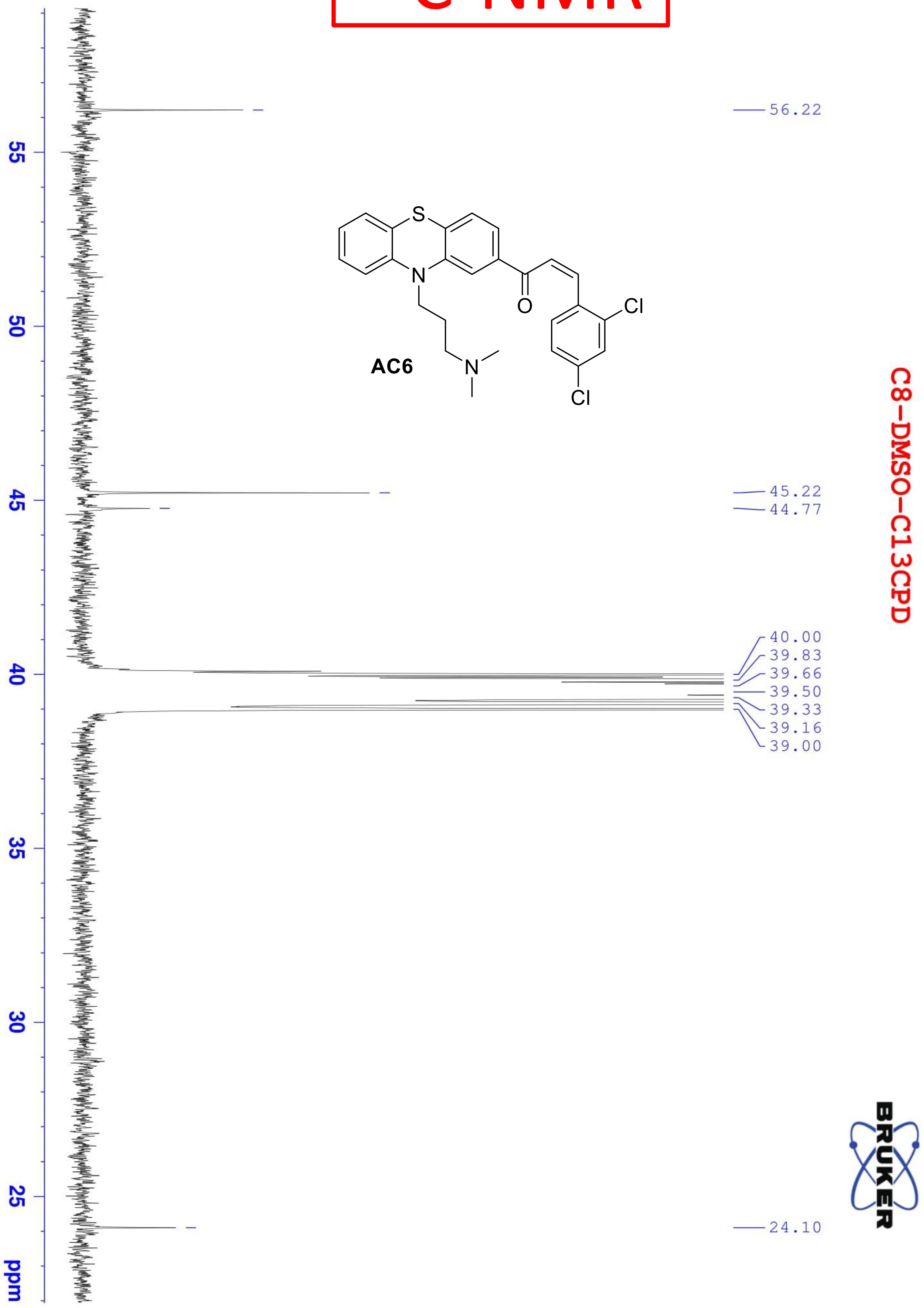

56.22
45.22
44.77
40.00
39.83
39.66
39.50
39.33
39.16
39.00
24.10
AC6
S
N
O
Cl
Cl
N
C8–DMSO–C13CPD
55
50
45
40
35
30
25
ppm
BRUKER

VIEN CONG NGHE HOA HOC
01 Mac Dinh Chi - Q.1 - Tp HCM

IR

Tel: 08.38296127
Fax: 08.38293889

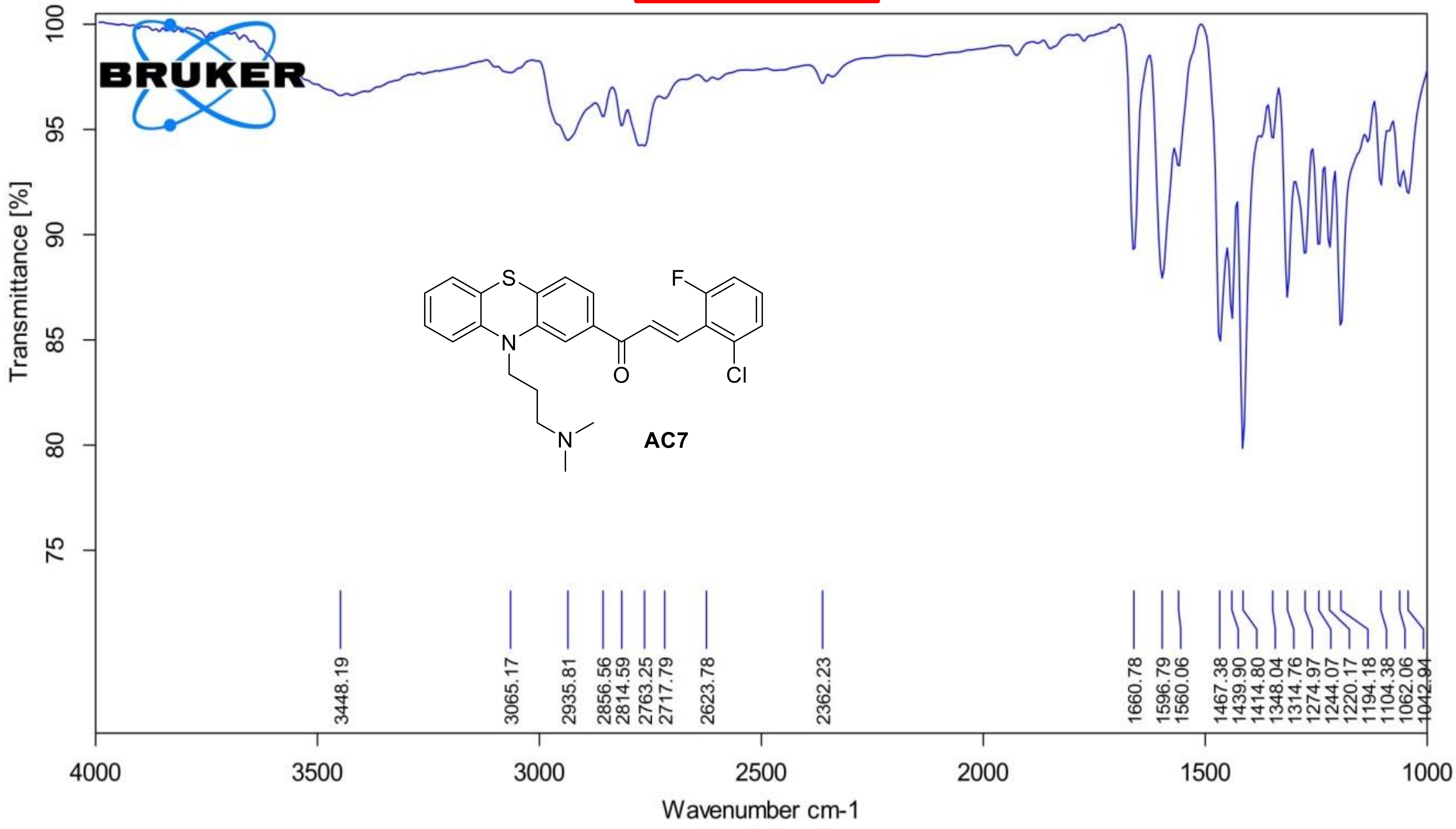

E:\OPUS 7\2017\THANG 6\20170606\C6.0	C6	TENSOR 27 - BRUKER - GERMANY	6/8/2017

MS

Qualitative Analysis Report

**Data Filename**	07-AC7.d	**Sample Name**	07-AC7
**Sample Type**	Sample	**Position**	P2-B7
**Instrument Name**	Instrument 1	**User Name**	
**Acq Method**	Cot ngan - MSMS_Pos.m	**Acquired Time**	22/08/2020 10:55:28 AM
**IRM Calibration Status**	Success	**DA Method**	COTNGAN.M.m
**Comment**			

**Sample Group**		**Info.**	
**Stream Name**	LC 1	**Acquisition SW Version**	6200 series TOF/6500 series Q-TOF B.06.01 (B6172 SP1)

User Chromatograms

Fragmentor Voltage 140 **Collision Energy** 0 **Ionization Mode** ESI

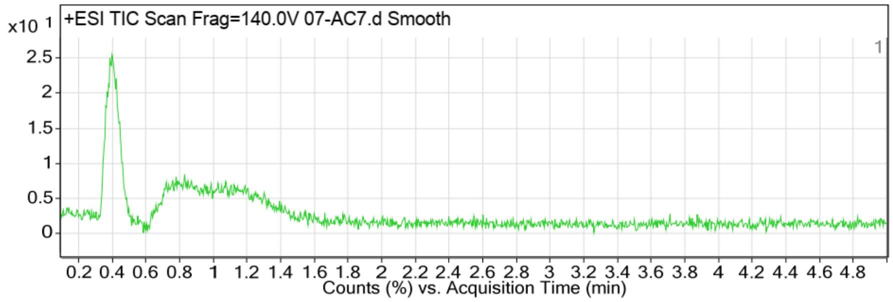

User Spectra

Fragmentor Voltage	Collision Energy	Ionization Mode
140	0	ESI

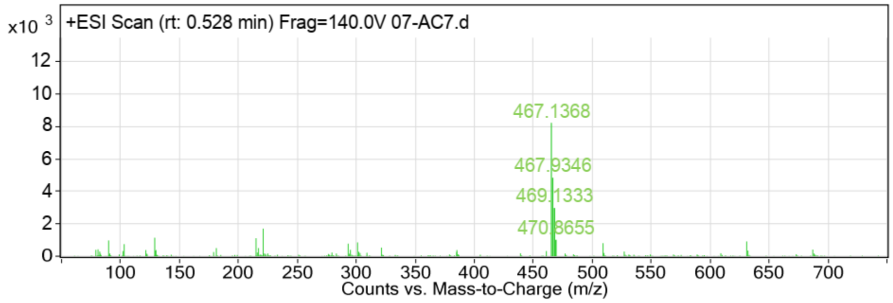

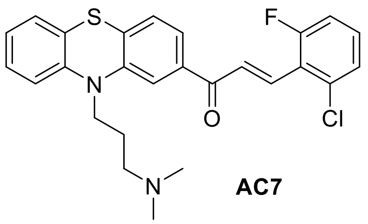

AC7

--- End Of Report ---

MS

**Sample Name**	07-AC7	**Position**	P2-B7	**Instrument Name**	Instrument 1	**User Name**	
**Inj Vol**	2	**InjPosition**		**SampleType**	Sample	**IRM Calibration Status**	Success
**Data Filename**	07-AC7.d	**ACQ Method**	Cot ngan - MSMS_Pos.	**Comment**		**Acquired Time**	22/08/2020 10:55:28 AM

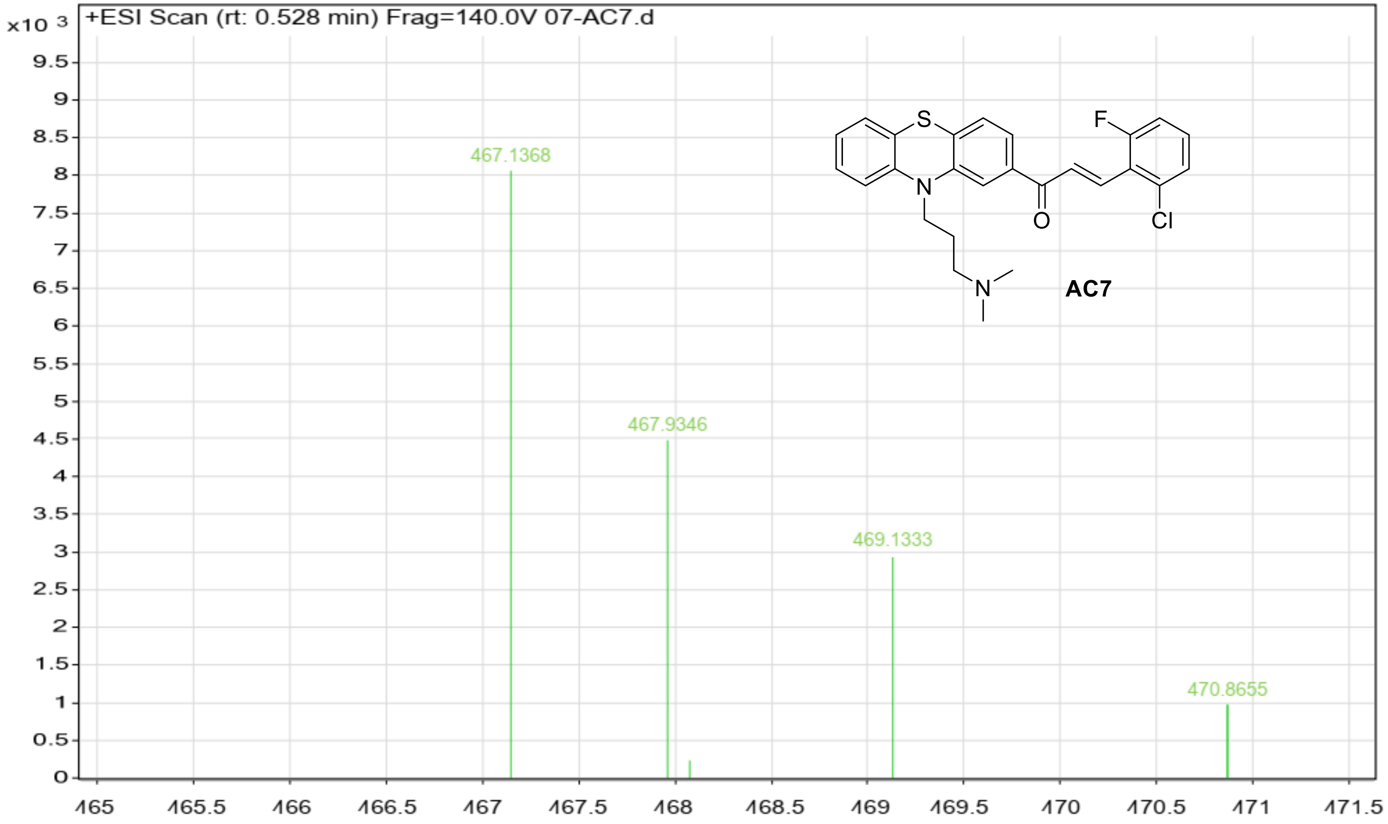

^{1}H-NMR

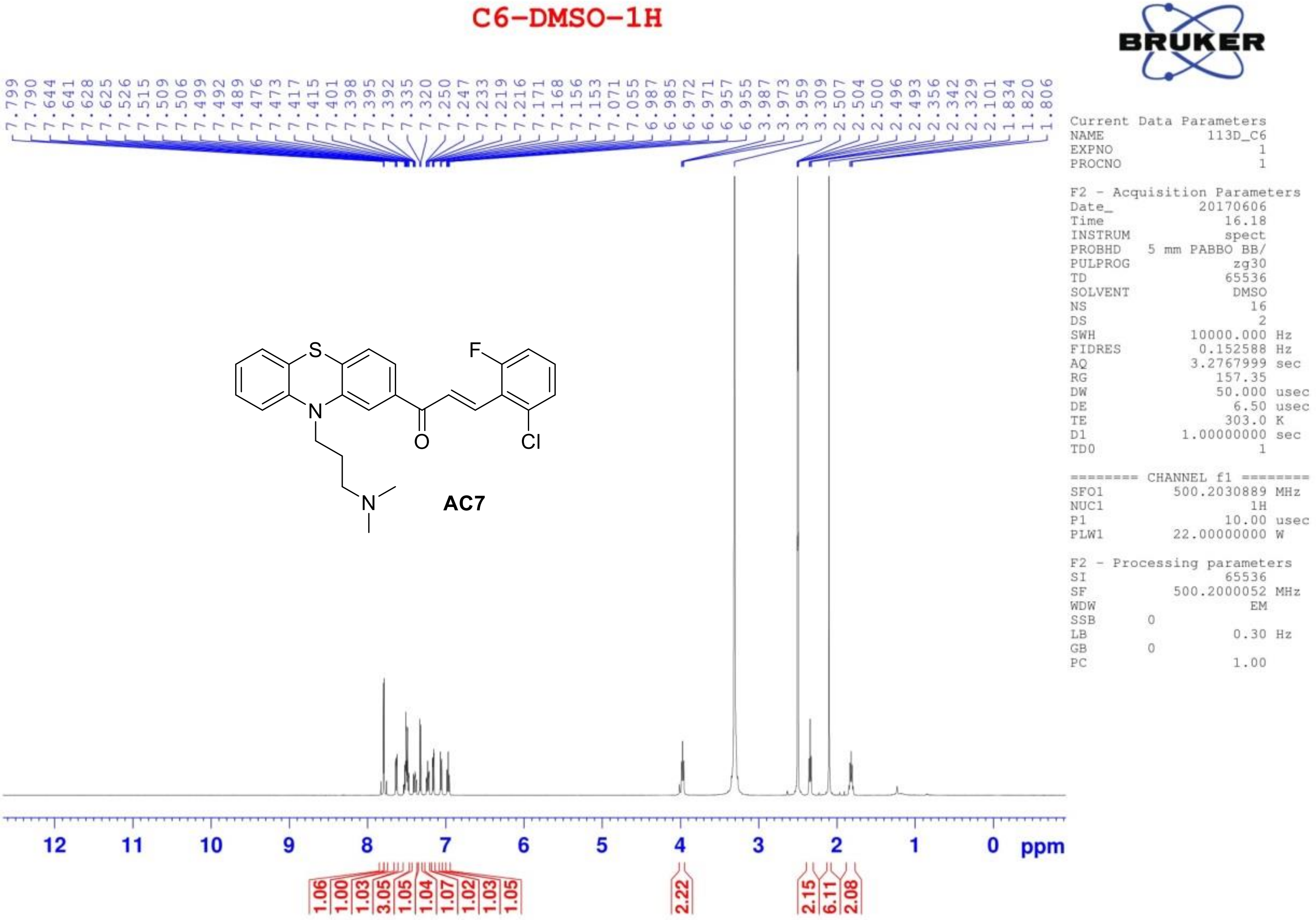

C6-DMSO-1H
BRUKER
7.799 7.790 7.644 7.641 7.628 7.625 7.526 7.515 7.509 7.506 7.499 7.492 7.489 7.476 7.473 7.417 7.415 7.401 7.398 7.395 7.392 7.335 7.320 7.250 7.247 7.233 7.219 7.216 7.171 7.168 7.156 7.153 7.071 7.055 6.987 6.985 6.972 6.971 6.957 6.955 3.987 3.973 3.959 3.309 2.507 2.504 2.500 2.496 2.493 2.356 2.342 2.329 2.101 1.834 1.820 1.806
Current Data Parameters
NAME 113D_C6
EXPNO 1
PROCNO 1
F2 - Acquisition Parameters
Date_ 20170606
Time 16.18
INSTRUM spect
PROBHD 5 mm PABBO BB/
PULPROG zg30
TD 65536
SOLVENT DMSO
NS 16
DS 2
SWH 10000.000 Hz
FIDRES 0.152588 Hz
AQ 3.2767999 sec
RG 157.35
DW 50.000 usec
DE 6.50 usec
TE 303.0 K
D1 1.00000000 sec
TD0 1
======== CHANNEL f1 ========
SFO1 500.2030889 MHz
NUC1 1H
P1 10.00 usec
PLW1 22.00000000 W
F2 - Processing parameters
SI 65536
SF 500.2000052 MHz
WDW EM
SSB 0
LB 0.30 Hz
GB 0
PC 1.00
S
F
N
O
Cl
N
AC7
12 11 10 9 8 7 6 5 4 3 2 1 0 ppm
1.06 1.00 1.03 3.05 1.05 1.04 1.07 1.02 1.03 1.05 2.22 2.15 6.11 2.08

^{1}H-NMR

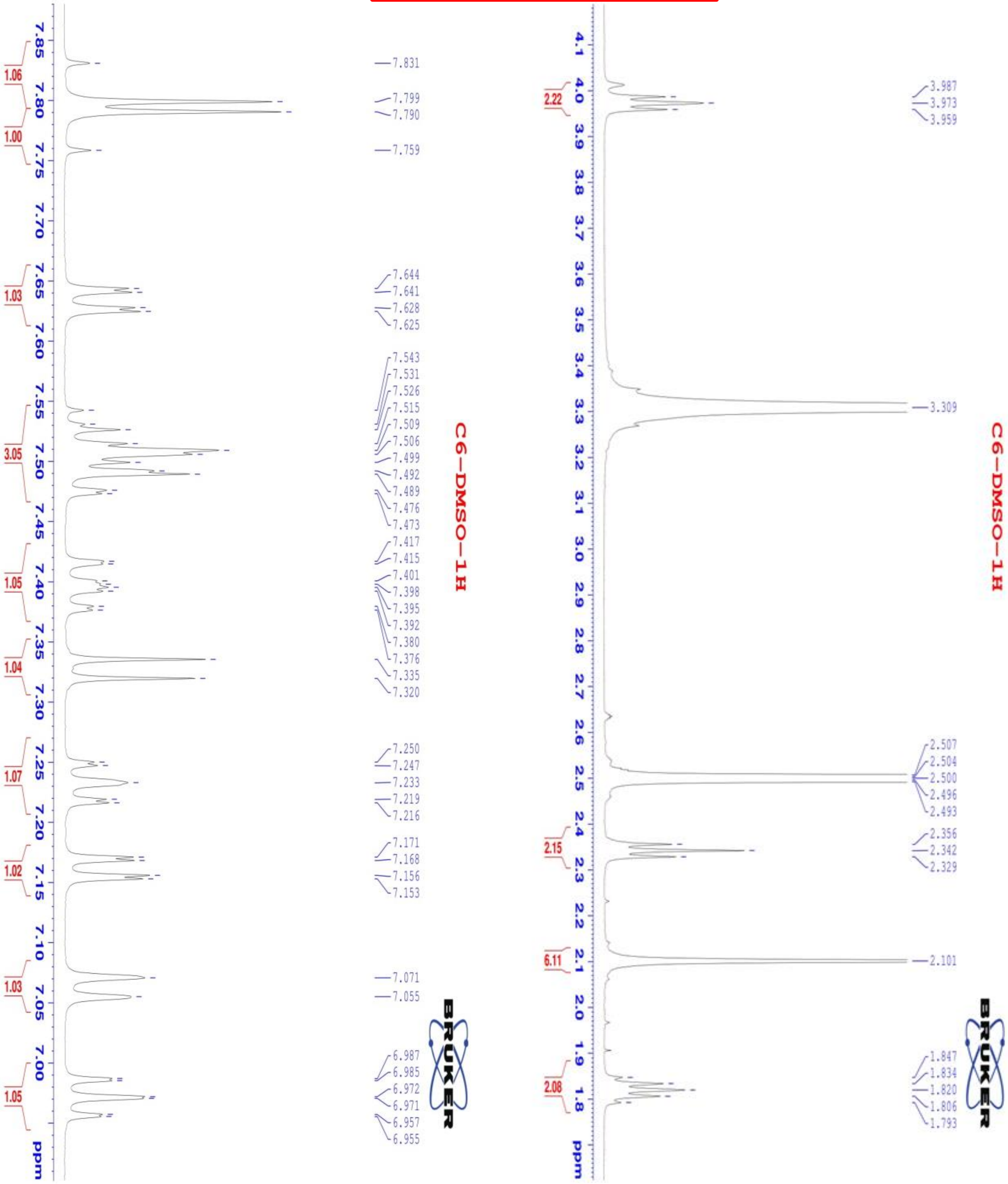

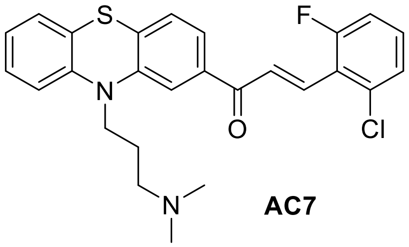

AC7

^{13}C-NMR

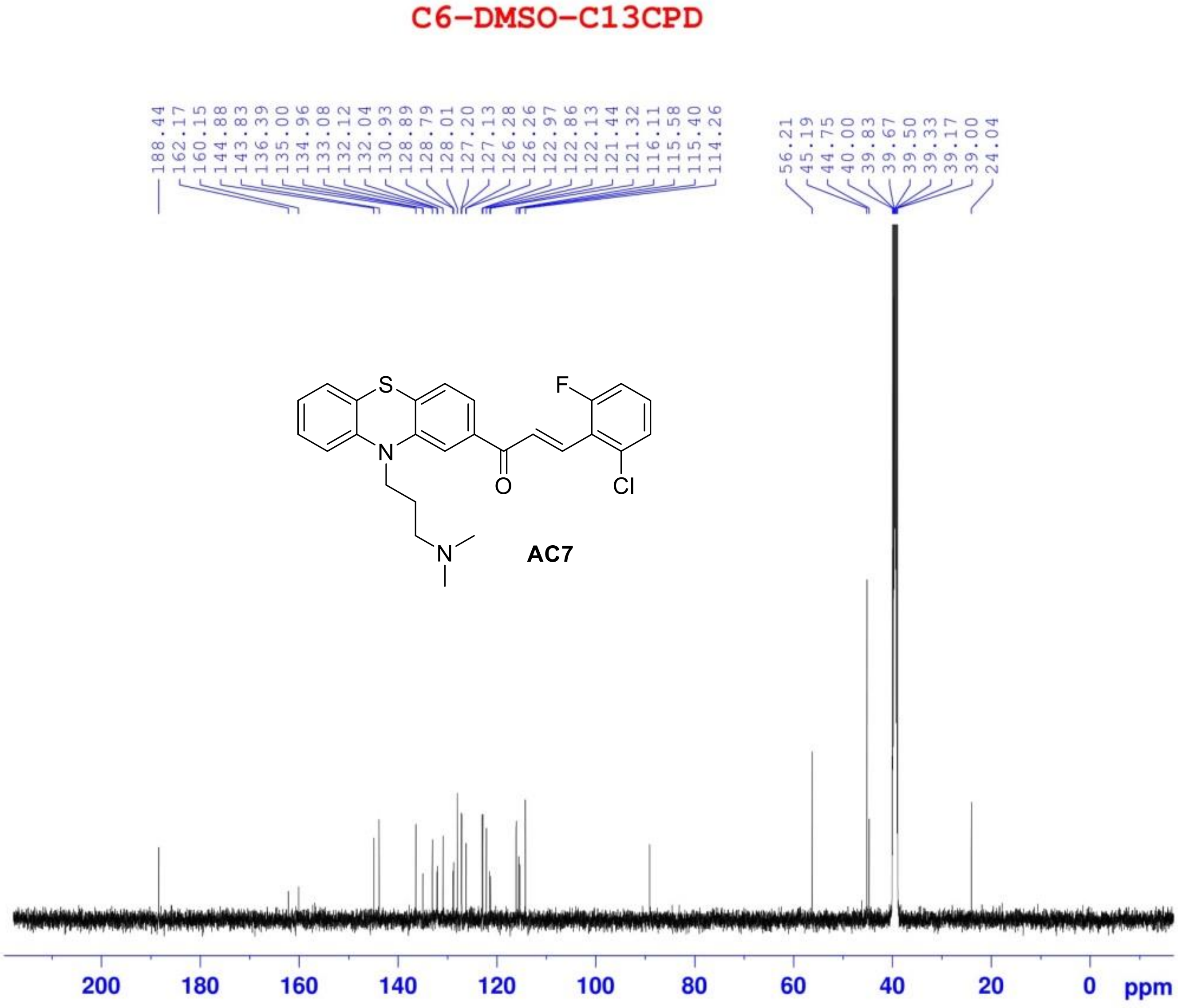

AC7

Current Data Parameters
NAME 113D_C6
EXPNO 2
PROCNO 1

F2 - Acquisition Parameters
Date_ 20170607
Time 1.15
INSTRUM spect
PROBHD 5 mm PABBO BB/
PULPROG zgpg30
TD 65536
SOLVENT DMSO
NS 3072
DS 4
SWH 29761.904 Hz
FIDRES 0.454131 Hz
AQ 1.1010048 sec
RG 198.57
DW 16.800 usec
DE 6.50 usec
TE 303.0 K
D1 2.00000000 sec
D11 0.03000000 sec
TD0 1

======== CHANNEL f1 ========
SFO1 125.7879670 MHz
NUC1 13C
P1 10.00 usec
PLW1 88.00000000 W

======== CHANNEL f2 ========
SFO2 500.2020008 MHz
NUC2 1H
CPDPRG[2 waltz16
PCPD2 80.00 usec
PLW2 22.00000000 W
PLW12 0.34375000 W
PLW13 0.22000000 W

F2 - Processing parameters
SI 32768
SF 125.7754563 MHz
WDW EM
SSB 0
LB 1.00 Hz
GB 0
PC 1.40

^{13}C-NMR

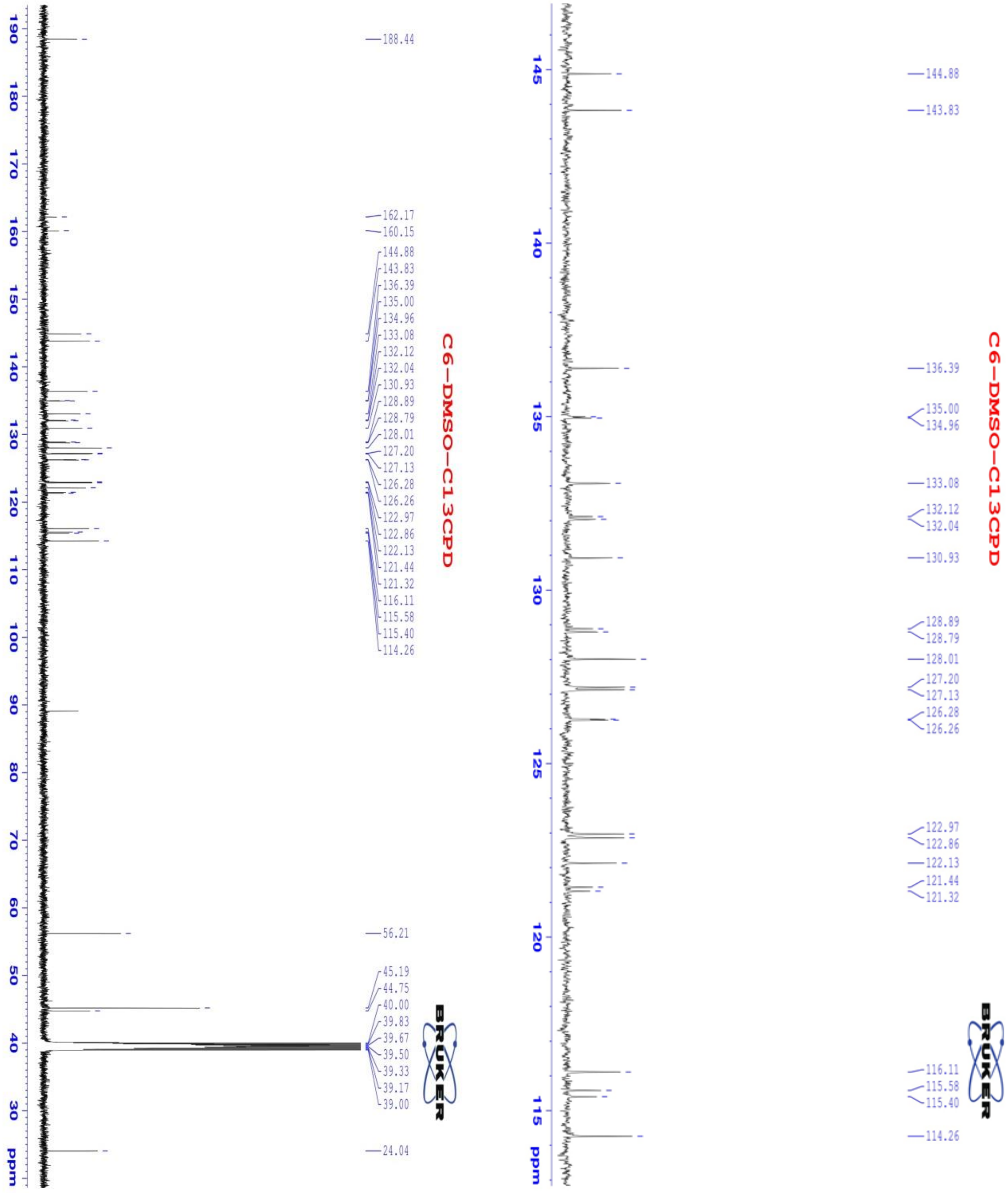
188.44
162.17
160.15
144.88
143.83
136.39
135.00
134.96
133.08
132.12
132.04
130.93
128.89
128.79
128.01
127.20
127.13
126.28
126.26
122.97
122.86
122.13
121.44
121.32
116.11
115.58
115.40
114.26
56.21
45.19
44.75
40.00
39.83
39.67
39.50
39.33
39.17
39.00
24.04
C6–DMSO–C13CPD
ppm

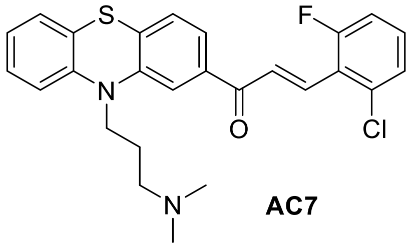
S
N
F
O
Cl
N

AC7

^{13}C-NMR

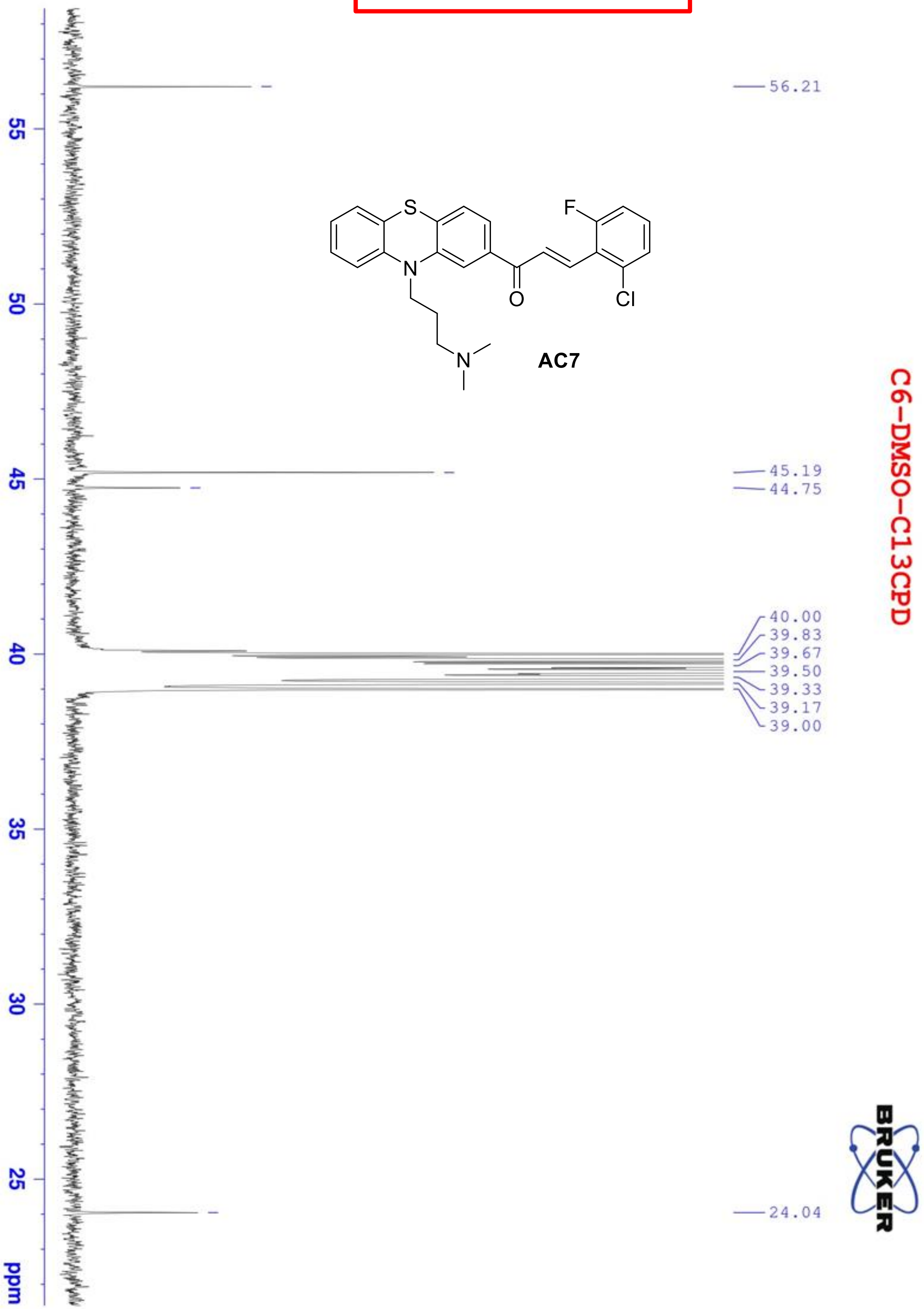
56.21
45.19
44.75
40.00
39.83
39.67
39.50
39.33
39.17
39.00
24.04
C6-DMSO-C13CPD
AC7
S
N
F
O
Cl
ppm
BRUKER

VIEN CONG NGHE HOA HOC
01 Mac Dinh Chi - Q.1 - Tp HCM

IR

Tel: 08.38296127
Fax: 08.38293889

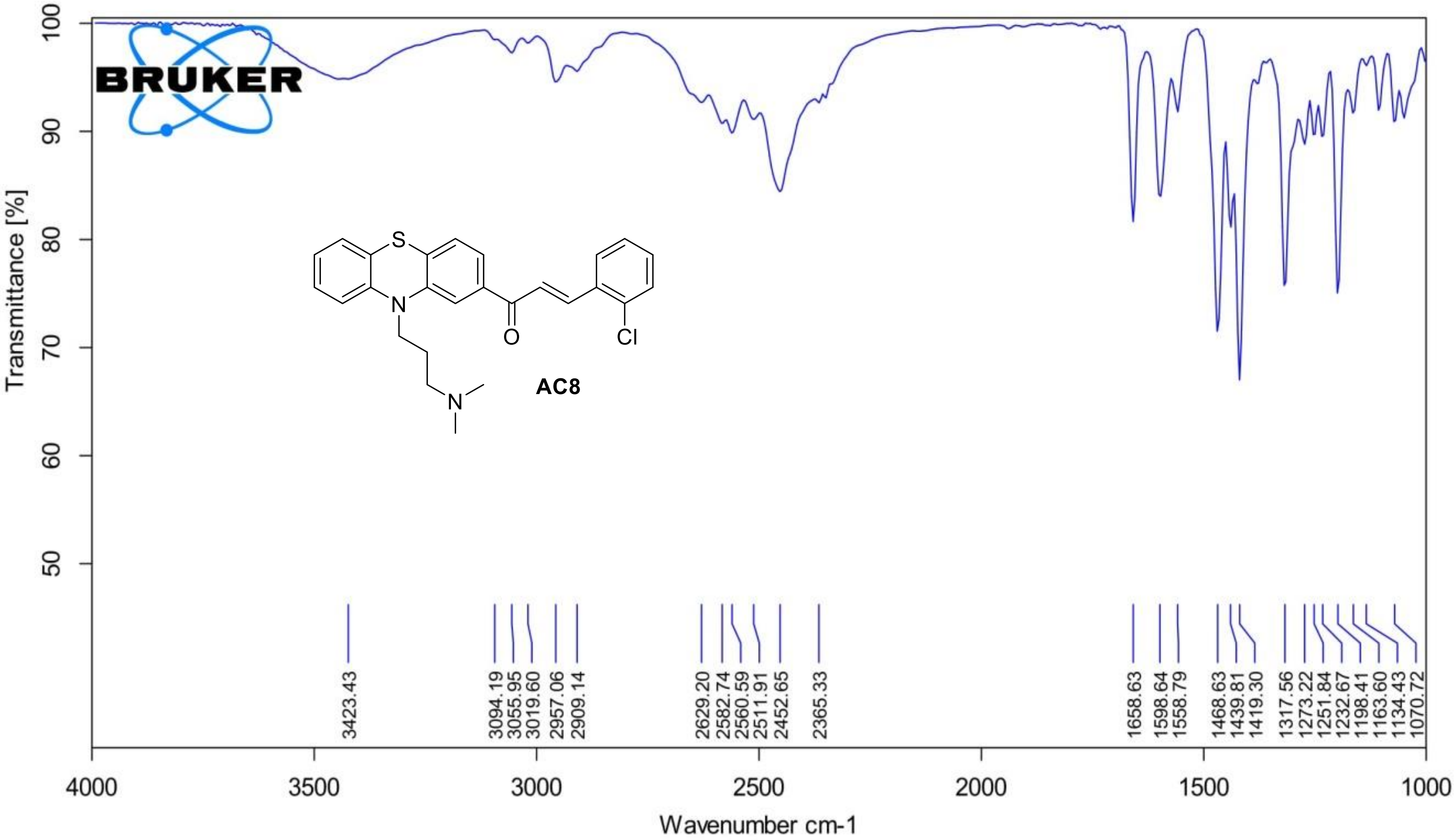

E:\OPUS 7\2017\THANG 6\20170606\C1.0	C1	TENSOR 27 - BRUKER - GERMANY	6/6/2017

MS

Qualitative Analysis Report

**Data Filename**	08-AC8.d	**Sample Name**	08-AC8
**Sample Type**	Sample	**Position**	P2-C5
**Instrument Name**	Instrument 1	**User Name**	
**Acq Method**	Cot ngan - MSMS_Pos.m	**Acquired Time**	22/08/2020 11:14:25 AM
**IRM Calibration Status**	Success	**DA Method**	COTNGAN.M.m
**Comment**			

**Sample Group**		**Info.**	
**Stream Name**	LC 1	**Acquisition SW Version**	6200 series TOF/6500 series Q-TOF B.06.01 (B6172 SP1)

User Chromatograms

Fragmentor Voltage 140 **Collision Energy** 0 **Ionization Mode** ESI

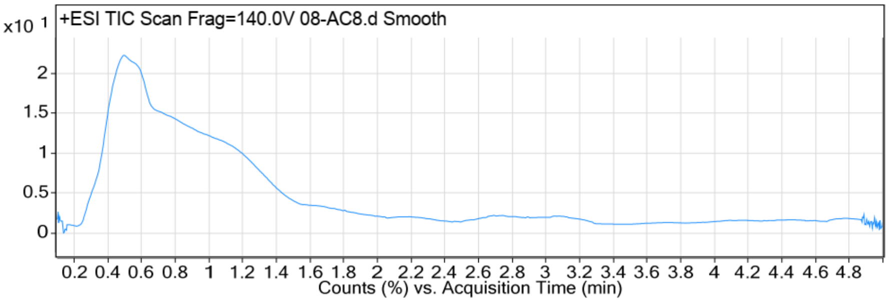

User Spectra

Fragmentor Voltage	Collision Energy	Ionization Mode
140	0	ESI

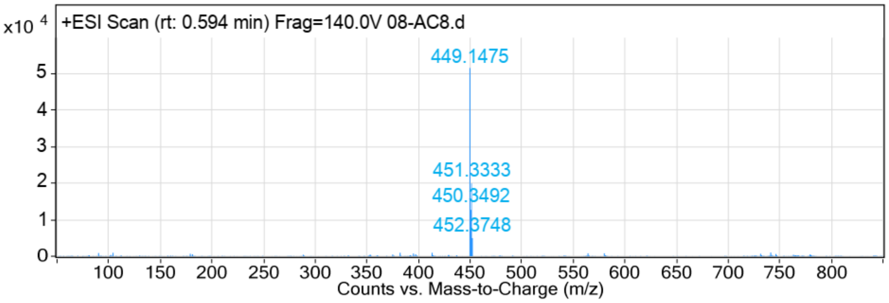

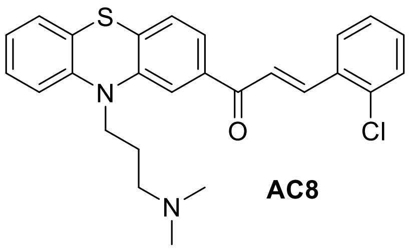

AC8

--- End Of Report ---

MS

**Sample Name**	08-AC8	**Position**	P2-C5	**Instrument Name**	Instrument 1	**User Name**	
**Inj Vol**	2	**InjPosition**		**SampleType**	Sample	**IRM Calibration Status**	Success
**Data Filename**	08-AC8.d	**ACQ Method**	Cot ngan - MSMS_Pos.	**Comment**		**Acquired Time**	22/08/2020 11:14:25 AM

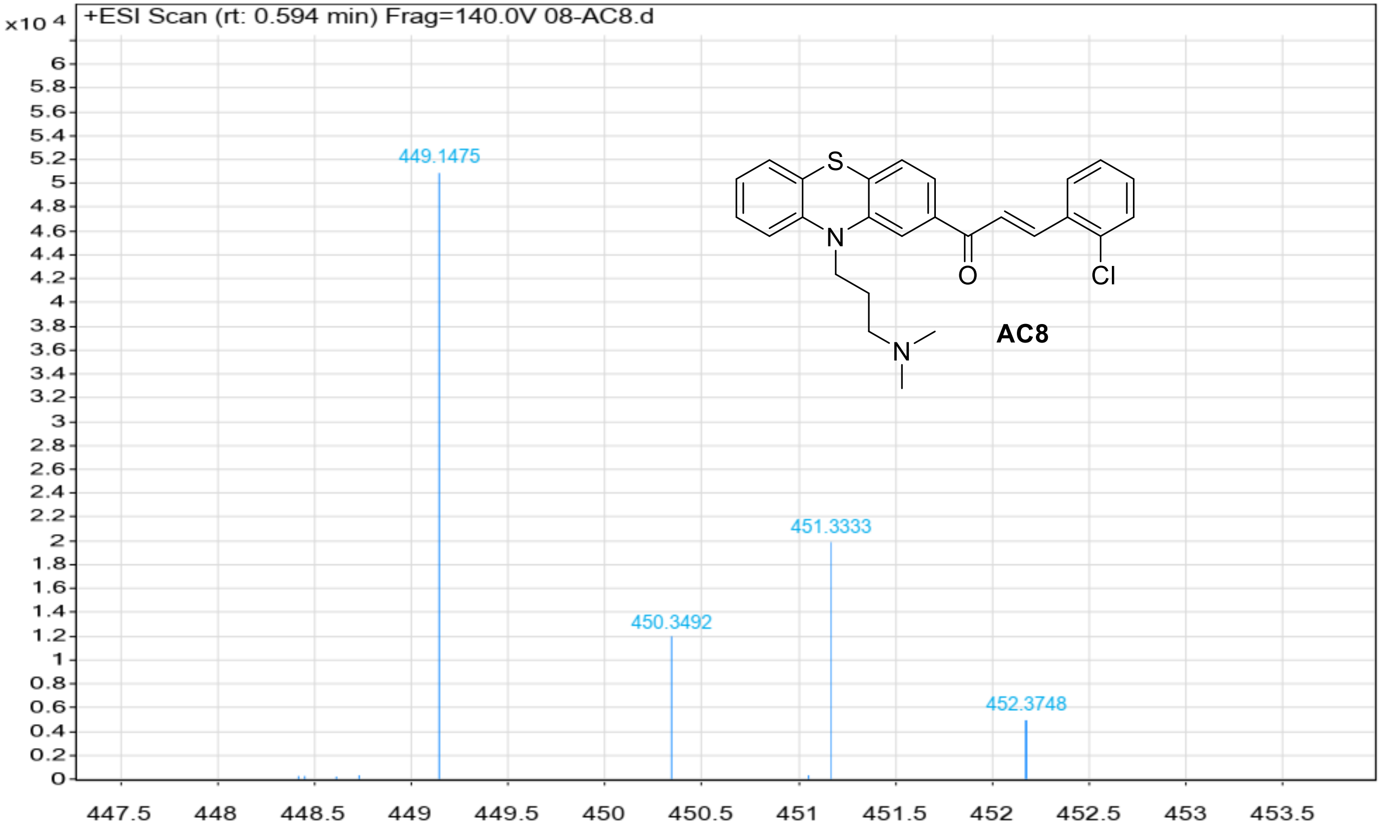

^{1}H-NMR

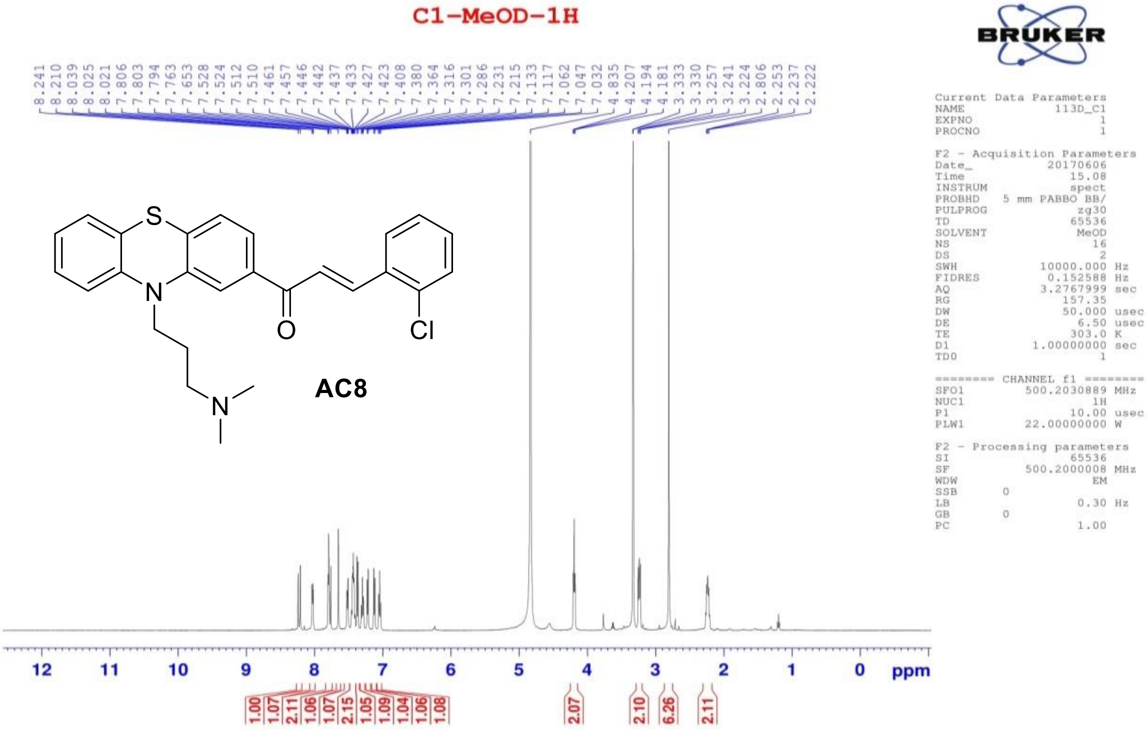
C1-MeOD-1H
BRUKER
AC8
Current Data Parameters
NAME 113D_C1
EXPNO 1
PROCNO 1
F2 - Acquisition Parameters
Date_ 20170606
Time 15.08
INSTRUM spect
PROBHD 5 mm PABBO BB/
PULPROG zg30
TD 65536
SOLVENT MeOD
NS 16
DS 2
SWH 10000.000 Hz
FIDRES 0.152588 Hz
AQ 3.2767999 sec
RG 157.35
DW 50.000 usec
DE 6.50 usec
TE 303.0 K
D1 1.00000000 sec
TD0 1
======== CHANNEL f1 ========
SFO1 500.2030889 MHz
NUC1 1H
P1 10.00 usec
PLW1 22.00000000 W
F2 - Processing parameters
SI 65536
SF 500.2000008 MHz
WDW EM
SSB 0
LB 0.30 Hz
GB 0
PC 1.00
ppm

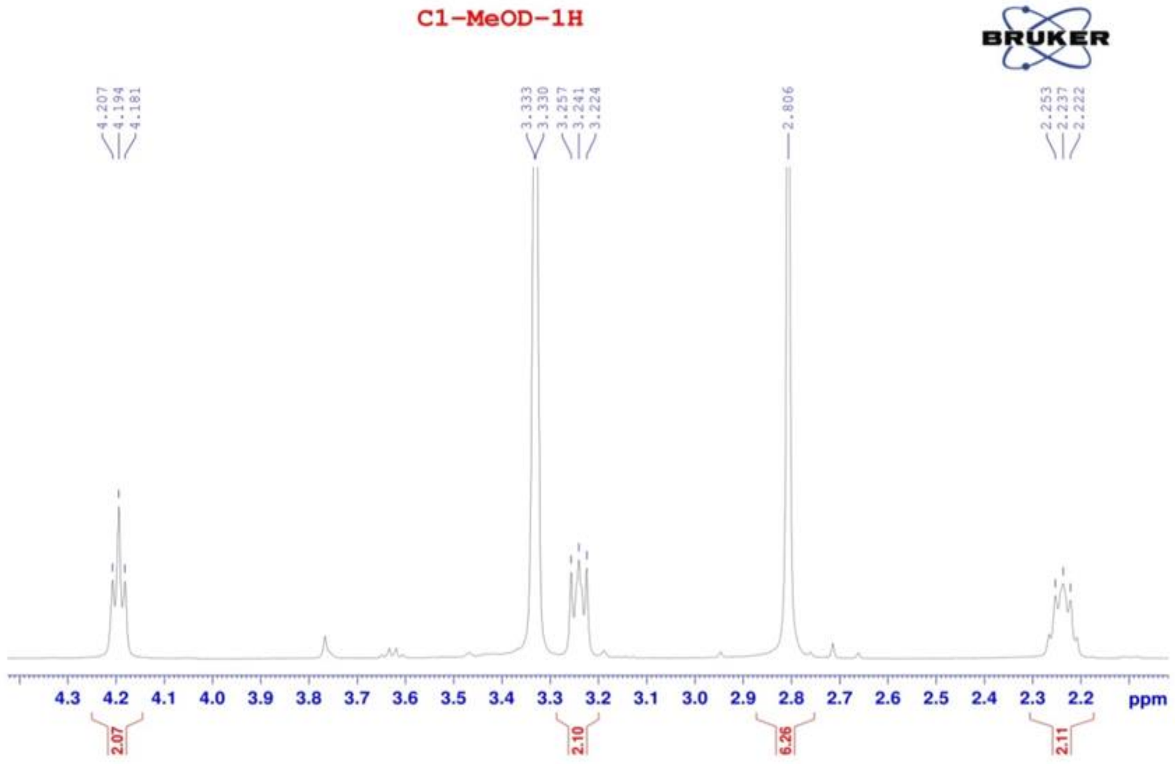

C1-MeOD-1H
BRUKER
4.207
4.194
4.181
3.333
3.330
3.257
3.241
3.224
2.806
2.253
2.237
2.222
4.3 4.2 4.1 4.0 3.9 3.8 3.7 3.6 3.5 3.4 3.3 3.2 3.1 3.0 2.9 2.8 2.7 2.6 2.5 2.4 2.3 2.2 ppm
2.07
2.10
6.26
2.11

^{1}H-NMR

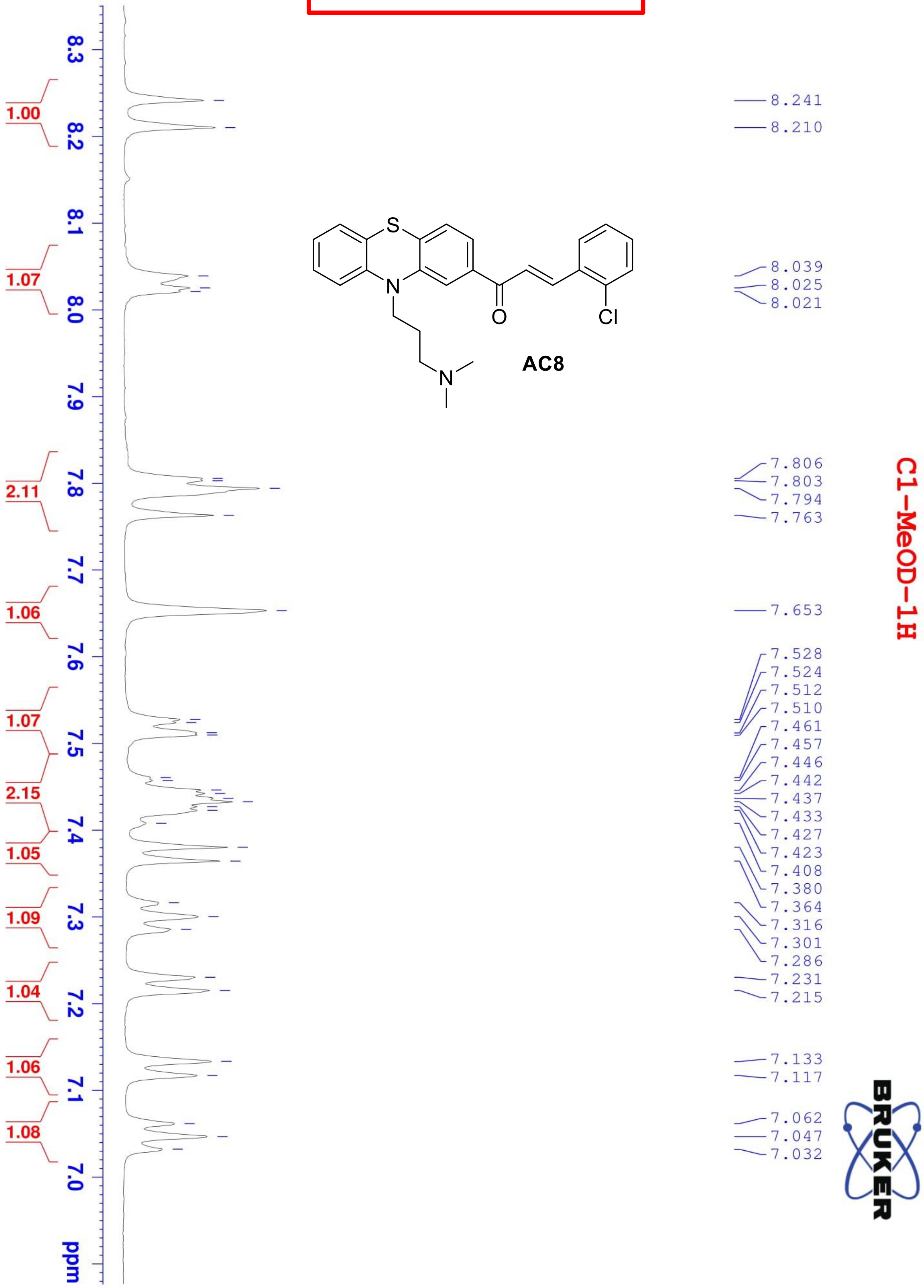

AC8
C1-MeOD-1H
8.241
8.210
8.039
8.025
8.021
7.806
7.803
7.794
7.763
7.653
7.528
7.524
7.512
7.510
7.461
7.457
7.446
7.442
7.437
7.433
7.427
7.423
7.408
7.380
7.364
7.316
7.301
7.286
7.231
7.215
7.133
7.117
7.062
7.047
7.032
1.00
1.07
2.11
1.06
1.07
2.15
1.05
1.09
1.04
1.06
1.08
8.3
8.2
8.1
8.0
7.9
7.8
7.7
7.6
7.5
7.4
7.3
7.2
7.1
7.0
ppm

^{13}C-NMR

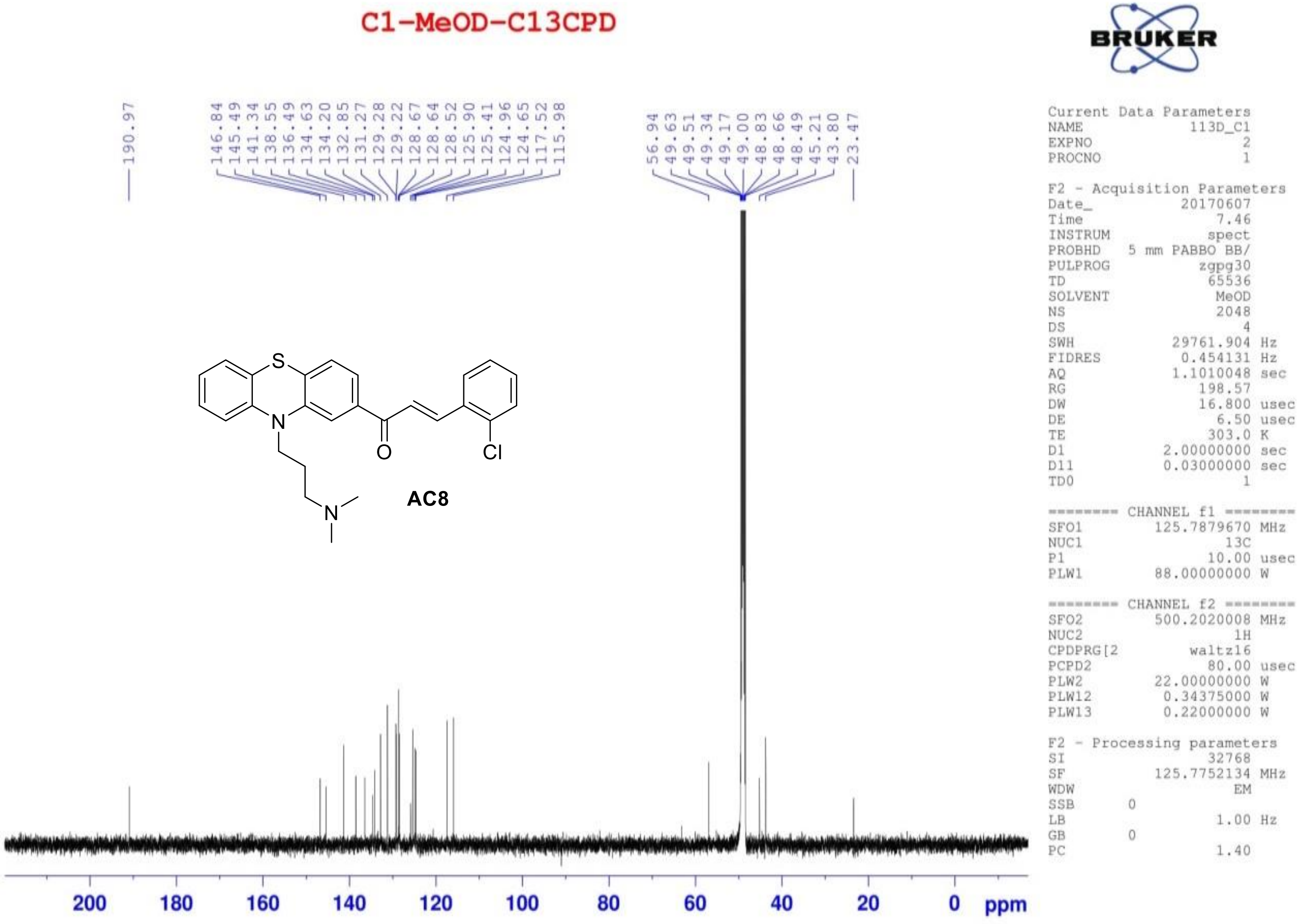
C1-MeOD-C13CPD
190.97
146.84
145.49
141.34
138.55
136.49
134.63
134.20
132.85
131.27
129.28
129.22
128.67
128.64
128.52
125.90
125.41
124.96
124.65
117.52
115.98
56.94
49.63
49.51
49.34
49.17
49.00
48.83
48.66
48.49
45.21
43.80
23.47
S
N
O
Cl
N
AC8
200
180
160
140
120
100
80
60
40
20
0
ppm
BRUKER
Current Data Parameters
NAME 113D_C1
EXPNO 2
PROCNO 1
F2 - Acquisition Parameters
Date_ 20170607
Time 7.46
INSTRUM spect
PROBHD 5 mm PABBO BB/
PULPROG zgpg30
TD 65536
SOLVENT MeOD
NS 2048
DS 4
SWH 29761.904 Hz
FIDRES 0.454131 Hz
AQ 1.1010048 sec
RG 198.57
DW 16.800 usec
DE 6.50 usec
TE 303.0 K
D1 2.00000000 sec
D11 0.03000000 sec
TD0 1
======== CHANNEL f1 ========
SFO1 125.7879670 MHz
NUC1 13C
P1 10.00 usec
PLW1 88.00000000 W
======== CHANNEL f2 ========
SFO2 500.2020008 MHz
NUC2 1H
CPDPRG[2 waltz16
PCPD2 80.00 usec
PLW2 22.00000000 W
PLW12 0.34375000 W
PLW13 0.22000000 W
F2 - Processing parameters
SI 32768
SF 125.7752134 MHz
WDW EM
SSB 0
LB 1.00 Hz
GB 0
PC 1.40

^{13}C-NMR

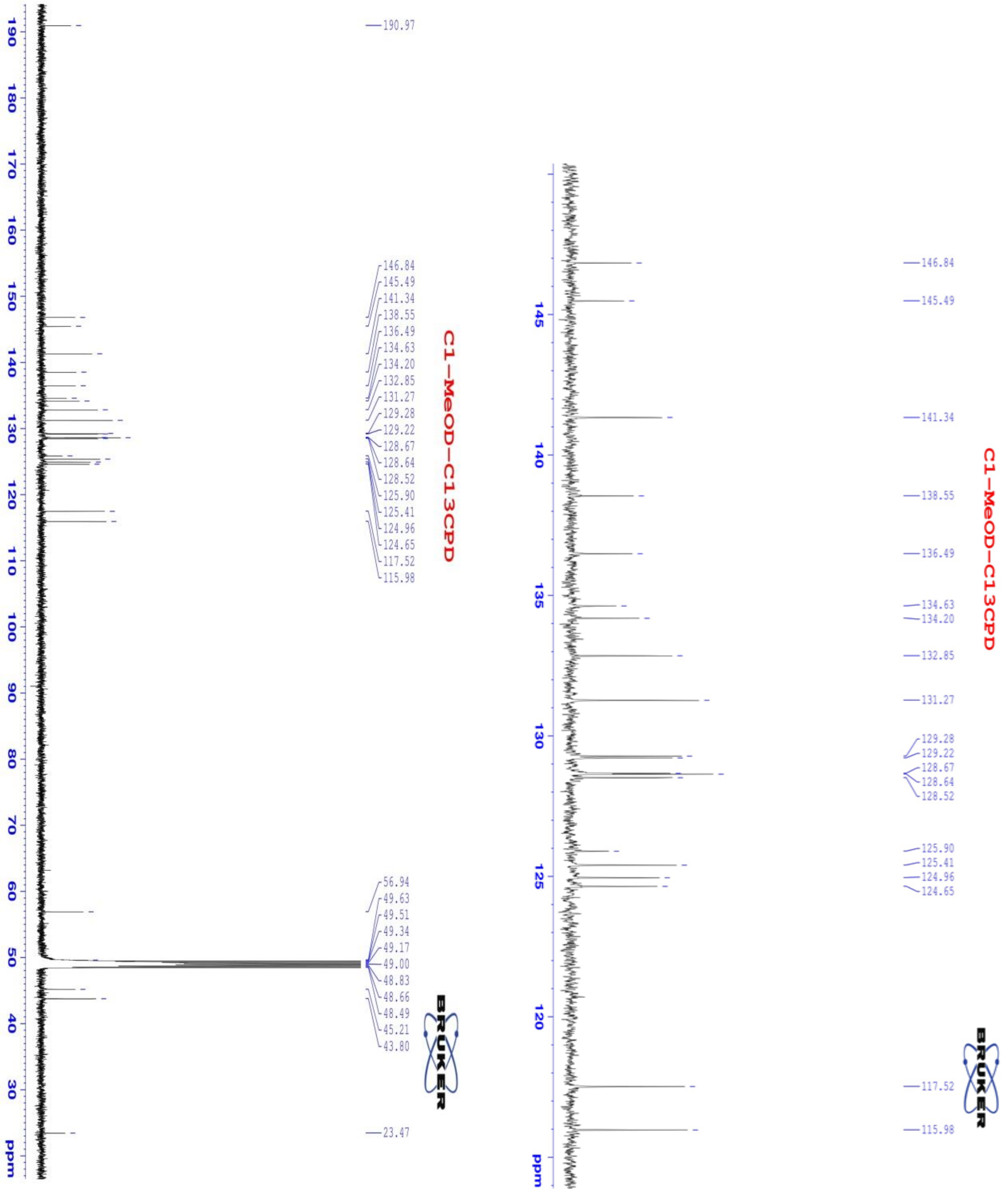

C1-MeOD-C13CPD
190.97
146.84
145.49
141.34
138.55
136.49
134.63
134.20
132.85
131.27
129.28
129.22
128.67
128.64
128.52
125.90
125.41
124.96
124.65
117.52
115.98
56.94
49.63
49.51
49.34
49.17
49.00
48.83
48.66
48.49
45.21
43.80
23.47
ppm
BRUKER

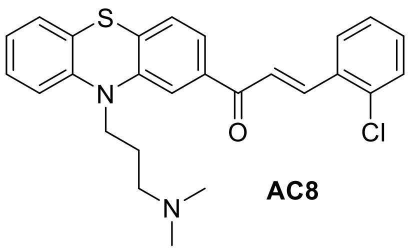

S
N
O
Cl
N

AC8

^{13}C-NMR

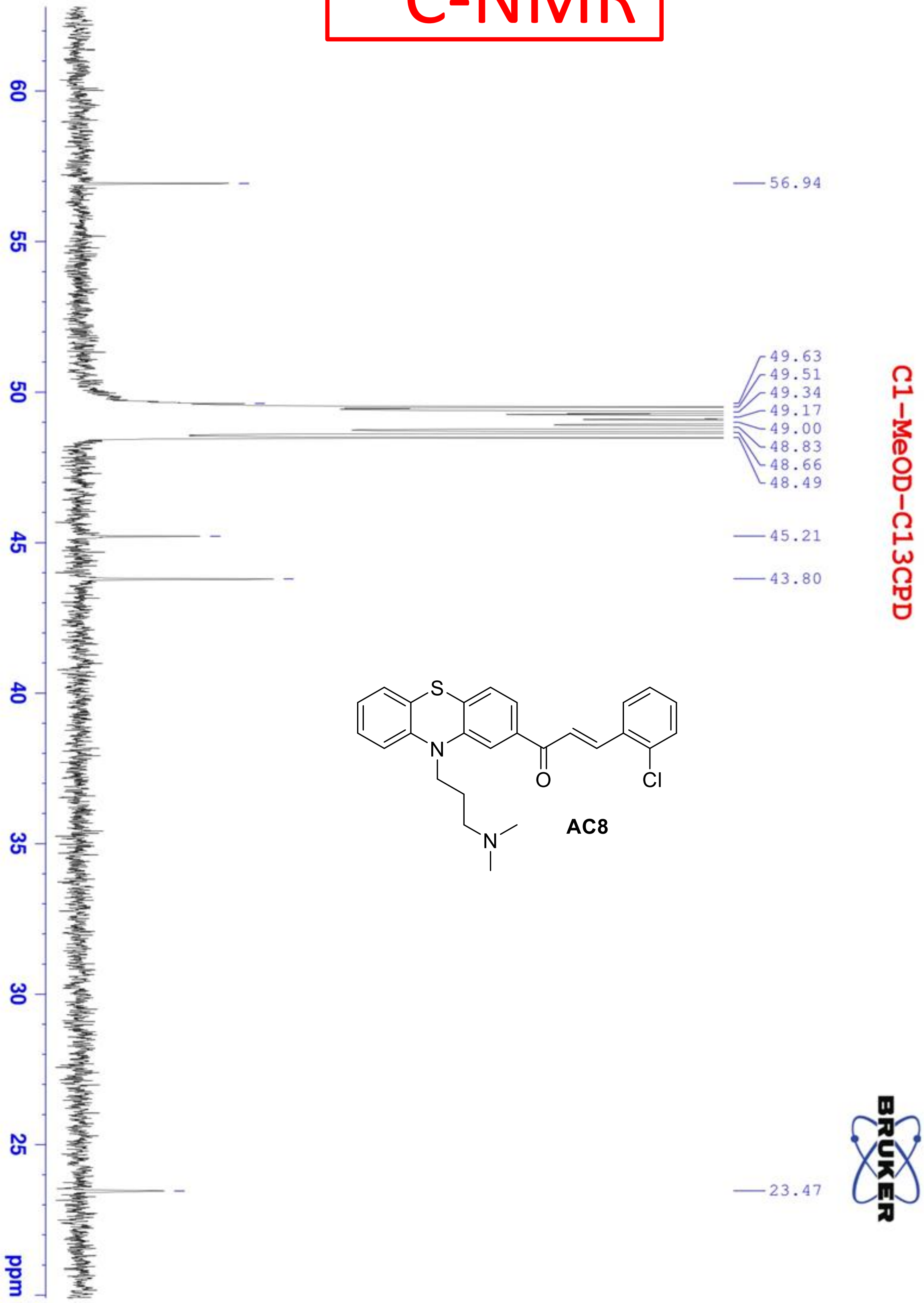

56.94
49.63
49.51
49.34
49.17
49.00
48.83
48.66
48.49
45.21
43.80
23.47
C1–MeOD–C13CPD
60
55
50
45
40
35
30
25
ppm
S
N
O
Cl
N

AC8

VIEN CONG NGHE HOA HOC
01 Mac Dinh Chi - Q.1 - Tp HCM

Tel: 08.38296127
Fax: 08.38293889

IR

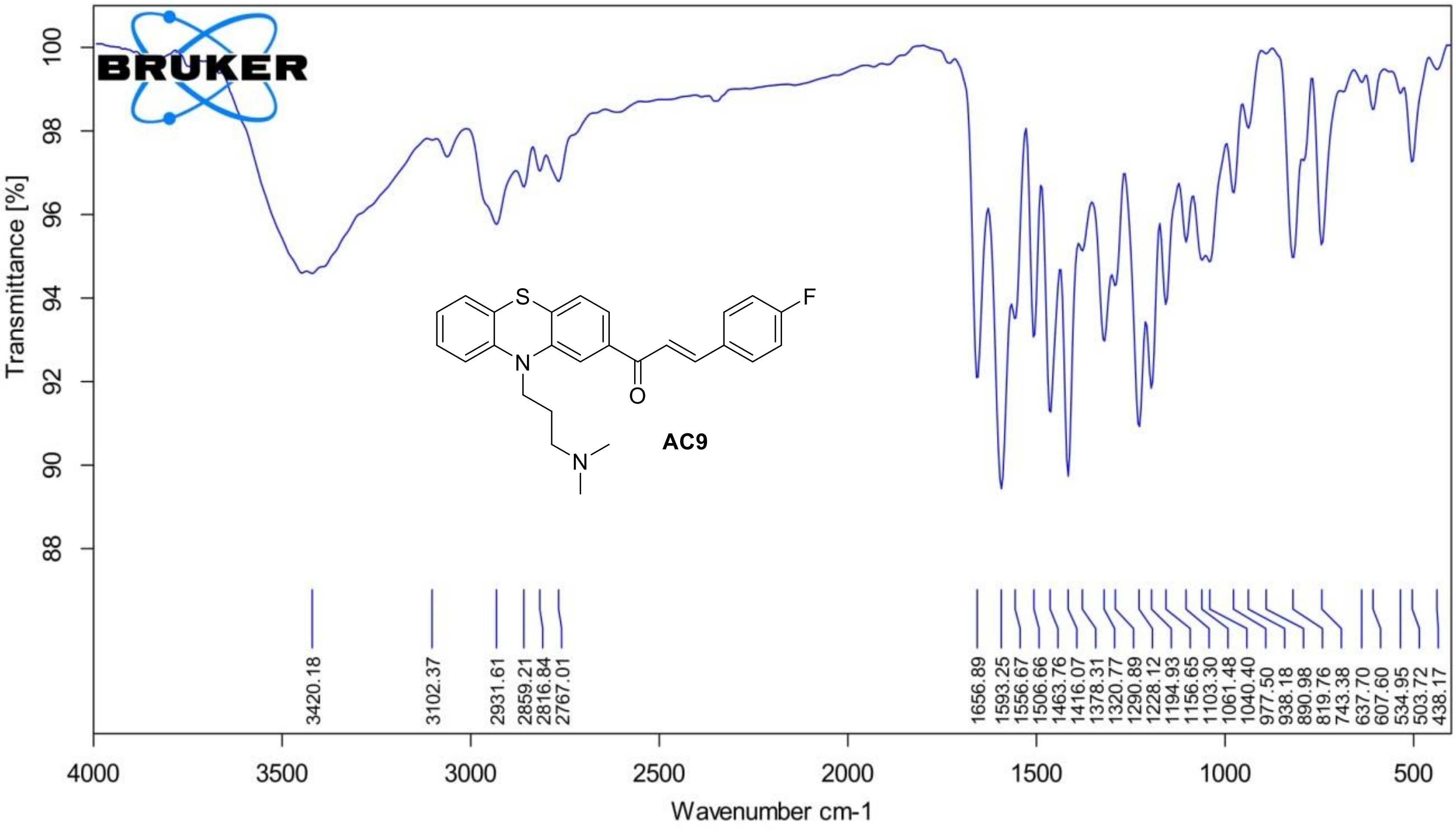

E:\OPUS 7\2017\THANG 7\20170714\C11.0 C11 TENSOR 27 - BRUKER - GERMANY	7/14/2017

MS

Qualitative Analysis Report

**Data Filename**	09-AC9.d	**Sample Name**	09-AC9
**Sample Type**	Sample	**Position**	P2-B2
**Instrument Name**	Instrument 1	**User Name**	
**Acq Method**	Cot ngan - MSMS_Pos.m	**Acquired Time**	22/08/2020 11:22:38 AM
**IRM Calibration Status**	Success	**DA Method**	COTNGAN.M.m
**Comment**			

**Sample Group**		**Info.**	
**Stream Name**	LC 1	**Acquisition SW Version**	6200 series TOF/6500 series Q-TOF B.06.01 (B6172 SP1)

User Chromatograms

Fragmentor Voltage 140 **Collision Energy** 0 **Ionization Mode** ESI

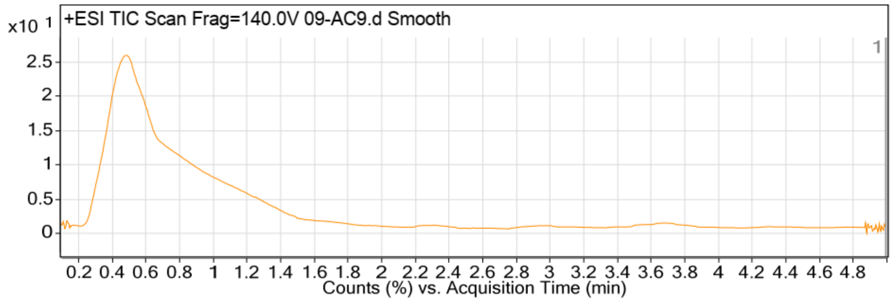

User Spectra

Fragmentor Voltage	Collision Energy	Ionization Mode
140	0	ESI

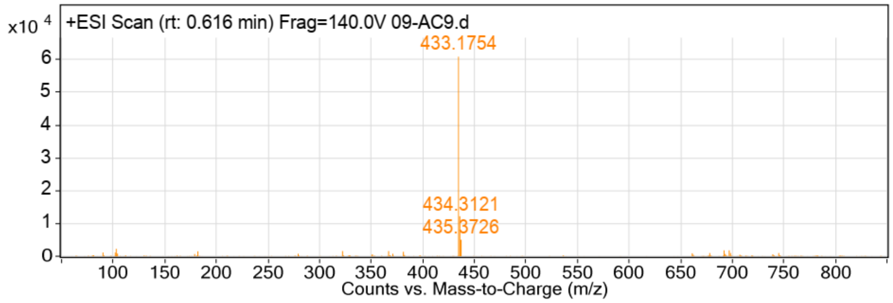

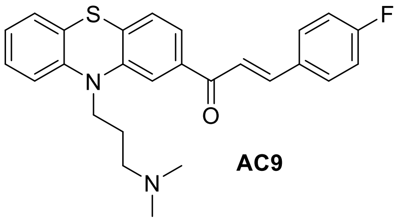

AC9

--- End Of Report ---

MS

+ESI Scan (rt: 0.616 min) Frag=140.0V BDE 4.d

**Sample Name**	09-AC9	**Position**	P2-B2	**Instrument Name**	Instrument 1	**User Name**	
**Inj Vol**	2	**InjPosition**		**SampleType**	Sample	**IRM Calibration Status**	Success
**Data Filename**	09-AC9.d	**ACQ Method**	Cot ngan - MSMS_Pos.	**Comment**		**Acquired Time**	22/08/2020 11:22:38 AM

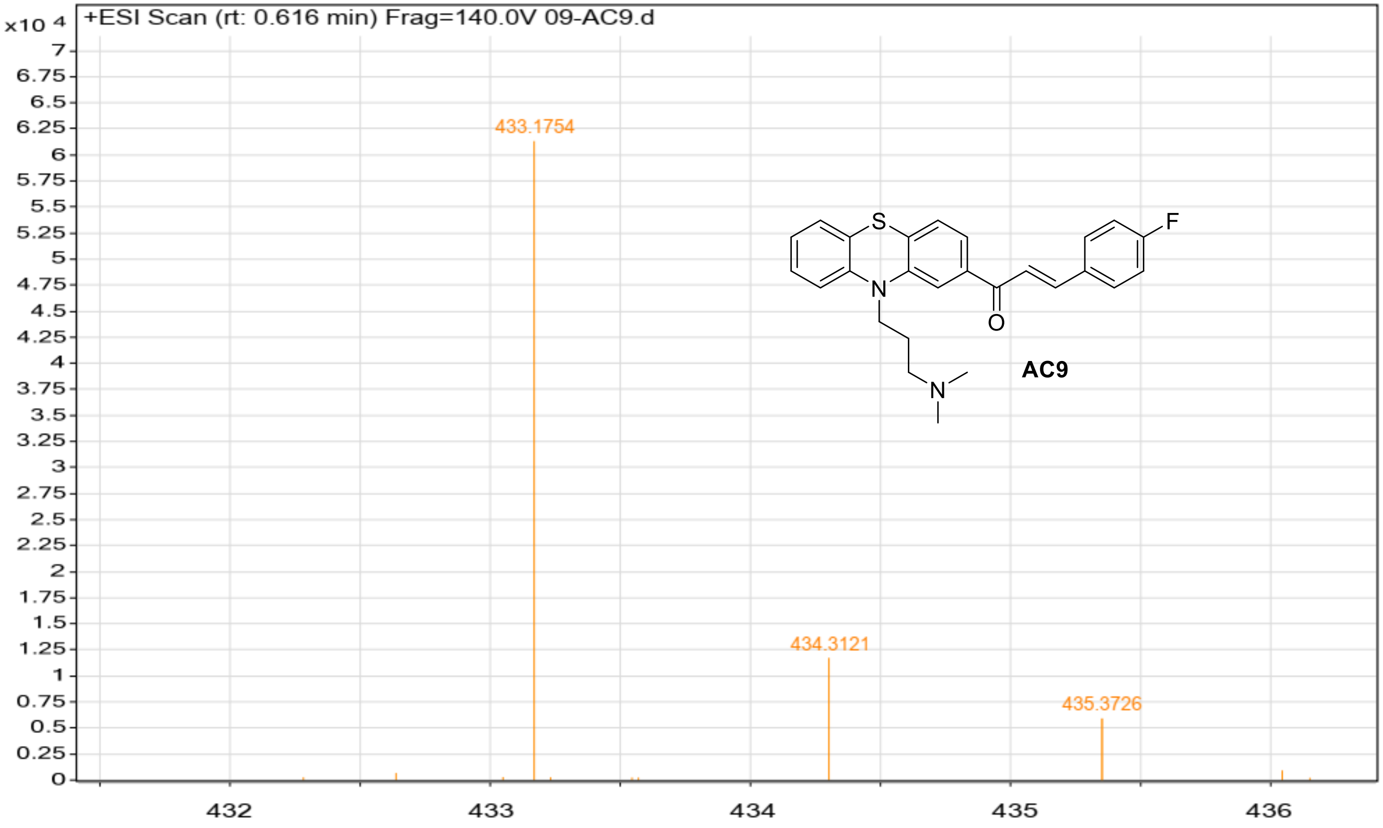

^{1}H-NMR

C11-MeOD-1H

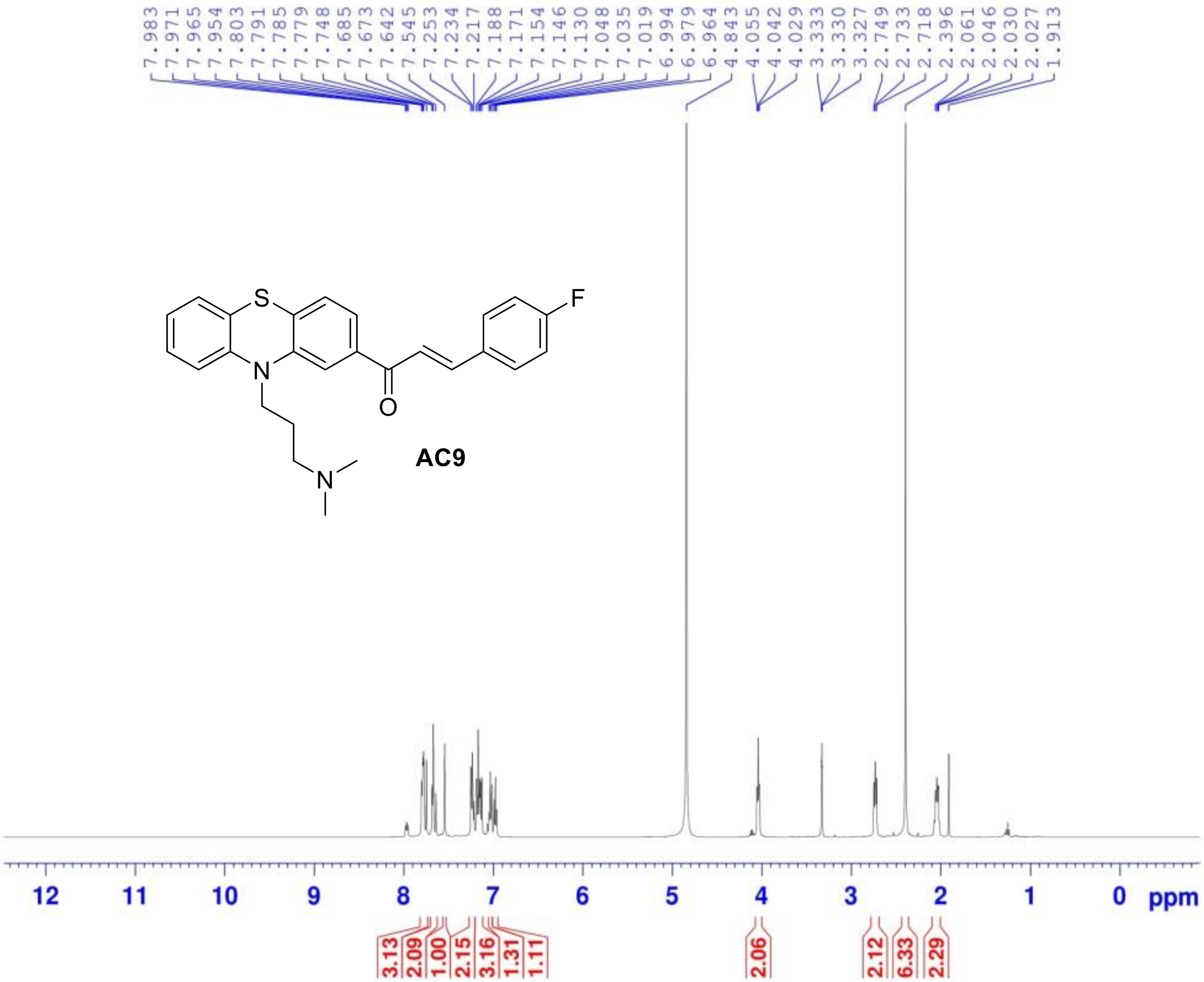

Current Data Parameters

NAME	114DAO_C11
EXPNO	1
PROCNO	1

F2 - Acquisition Parameters

Date_	20170704
Time	11.25
INSTRUM	spect
PROBHD	5 mm PABBO BB/
PULPROG	zg30
TD	65536
SOLVENT	MeOD
NS	16
DS	2
SWH	10000.000 Hz
FIDRES	0.152588 Hz
AQ	3.2767999 sec
RG	79.36
DW	50.000 usec
DE	6.50 usec
TE	303.0 K
D1	1.00000000 sec
TD0	1

======== CHANNEL f1 ========

SFO1	500.2030889 MHz
NUC1	1H
P1	10.00 usec
PLW1	22.00000000 W

F2 - Processing parameters

SI	65536
SF	500.2000016 MHz
WDW	EM
SSB	0
LB	0.30 Hz
GB	0
PC	1.00

^{1}H-NMR

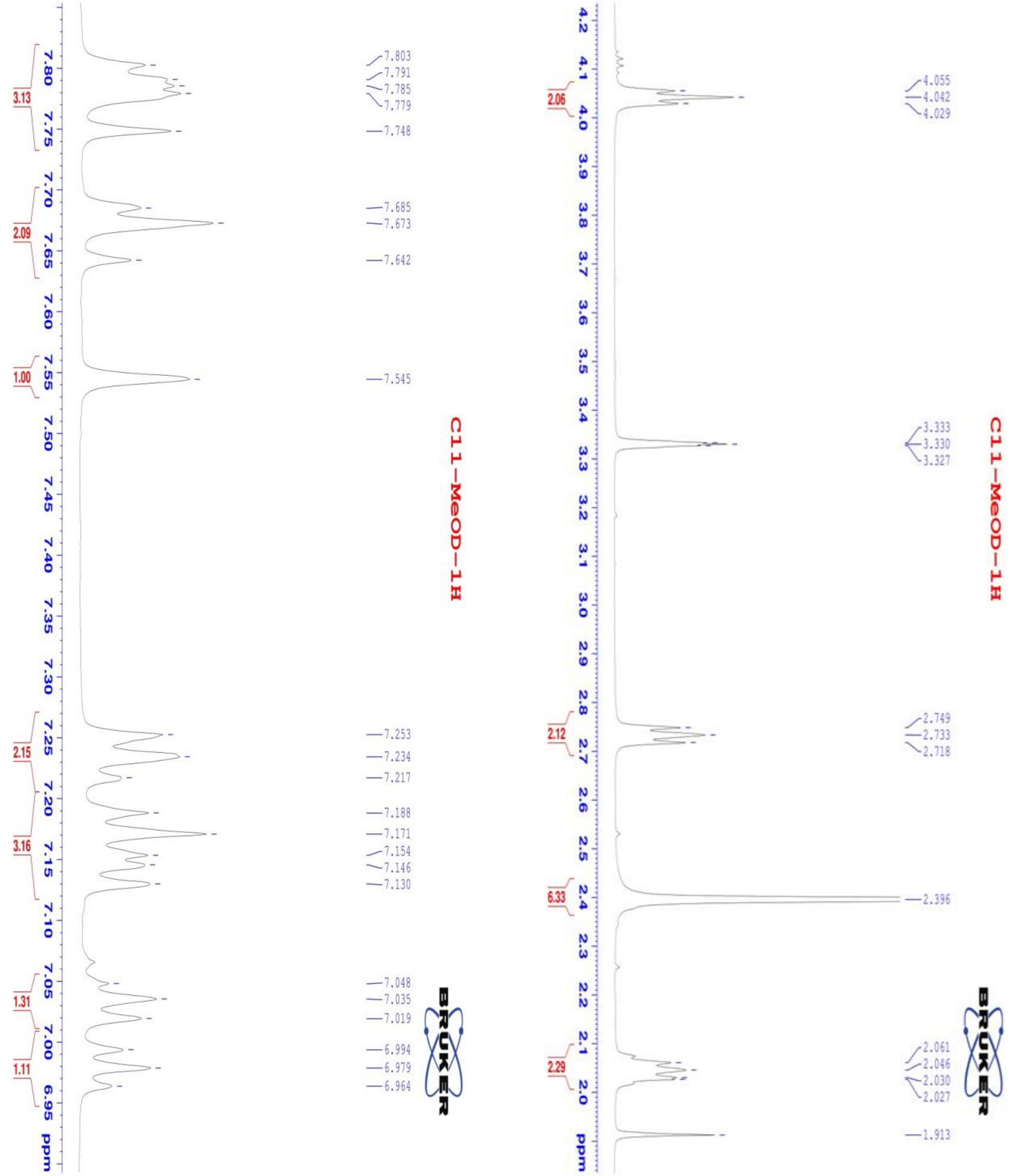
C11-MeOD-1H
7.803
7.791
7.785
7.779
7.748
7.685
7.673
7.642
7.545
7.253
7.234
7.217
7.188
7.171
7.154
7.146
7.130
7.048
7.035
7.019
6.994
6.979
6.964
3.13
2.09
1.00
2.15
3.16
1.31
1.11
ppm
C11-MeOD-1H
4.055
4.042
4.029
3.333
3.330
3.327
2.749
2.733
2.718
2.396
2.061
2.046
2.030
2.027
1.913
2.06
2.12
6.33
2.29
ppm

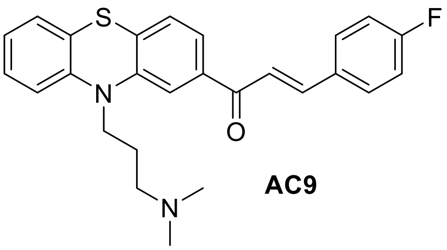
S
N
O
F
N

AC9

^{13}C-NMR

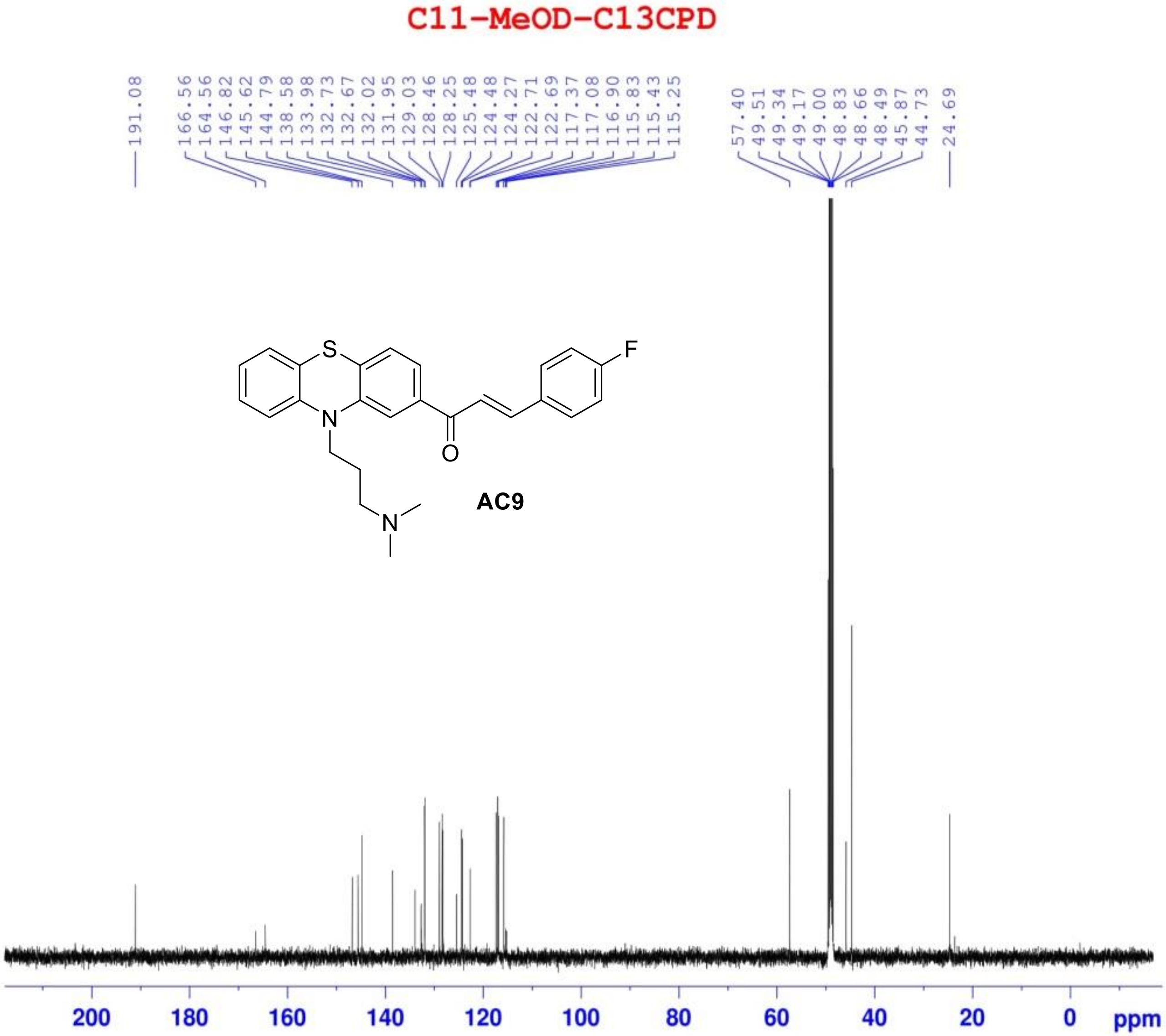

AC9

```
Current Data Parameters
NAME          113DAO_C11
EXPNO                  2
PROCNO                 1

F2 - Acquisition Parameters
Date_           20170705
Time               14.39
INSTRUM            spect
PROBHD   5 mm PABBO BB/
PULPROG          zgpg30
TD                 65536
SOLVENT             MeOD
NS                   128
DS                     4
SWH            29761.904 Hz
FIDRES          0.454131 Hz
AQ             1.1010048 sec
RG                198.57
DW                16.800 usec
DE                  6.50 usec
TE                 303.0 K
D1            2.00000000 sec
D11           0.03000000 sec
TD0                    1

======== CHANNEL f1 ========
SFO1         125.7879670 MHz
NUC1                 13C
P1                 10.00 usec
PLW1         88.00000000 W

======== CHANNEL f2 ========
SFO2         500.2020008 MHz
NUC2                  1H
CPDPRG[2         waltz16
PCPD2              80.00 usec
PLW2         22.00000000 W
PLW12         0.34375000 W
PLW13         0.22000000 W

F2 - Processing parameters
SI                 32768
SF           125.7752168 MHz
WDW                   EM
SSB      0
LB                  1.00 Hz
GB       0
PC                  1.40
```

^{13}C-NMR

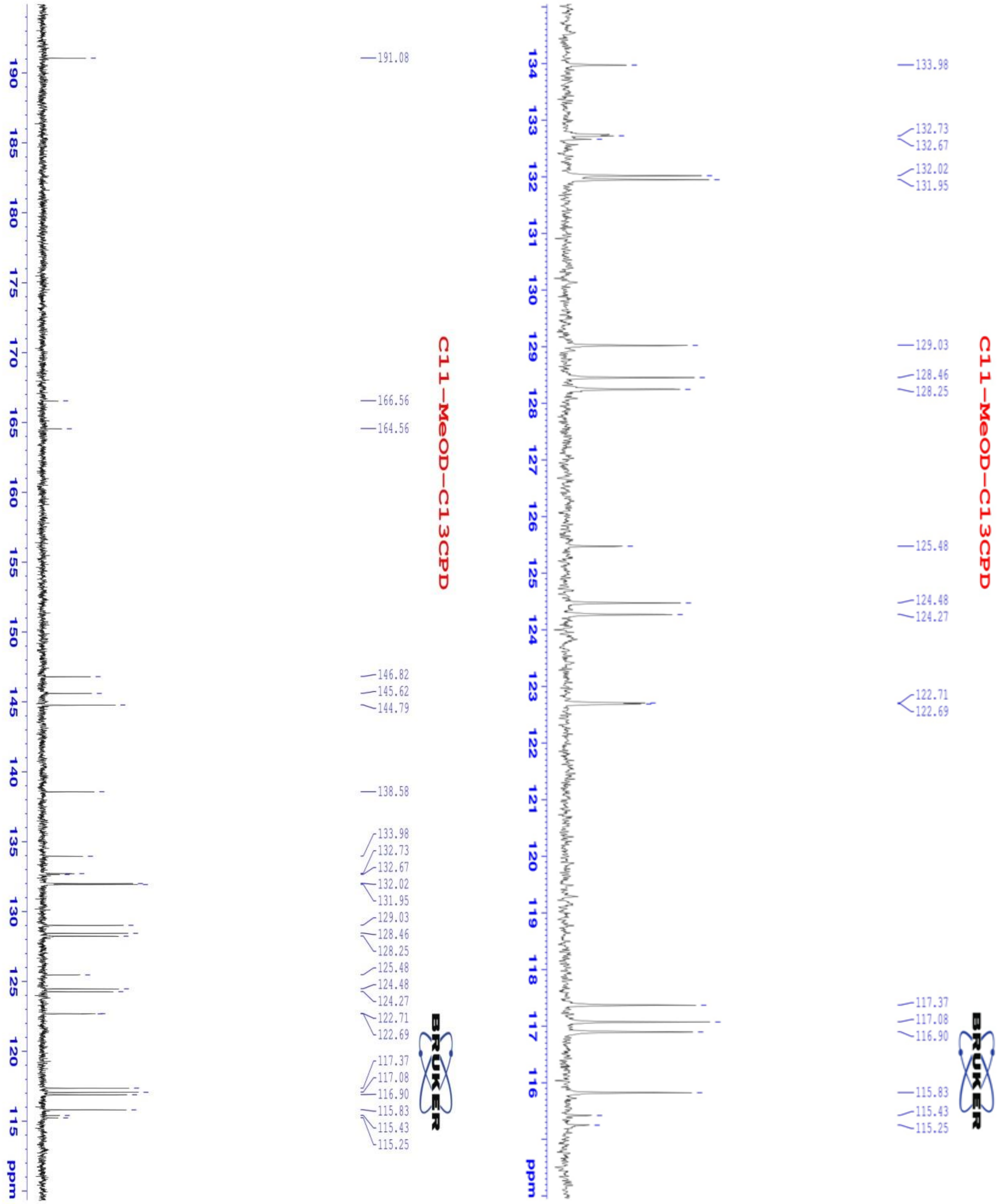
C11-MeOD-C13CPD
191.08
166.56
164.56
146.82
145.62
144.79
138.58
133.98
132.73
132.67
132.02
131.95
129.03
128.46
128.25
125.48
124.48
124.27
122.71
122.69
117.37
117.08
116.90
115.83
115.43
115.25
ppm

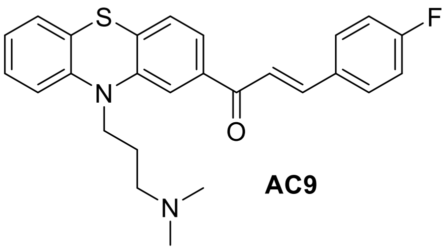
S
N
O
F
N

AC9

^{13}C-NMR

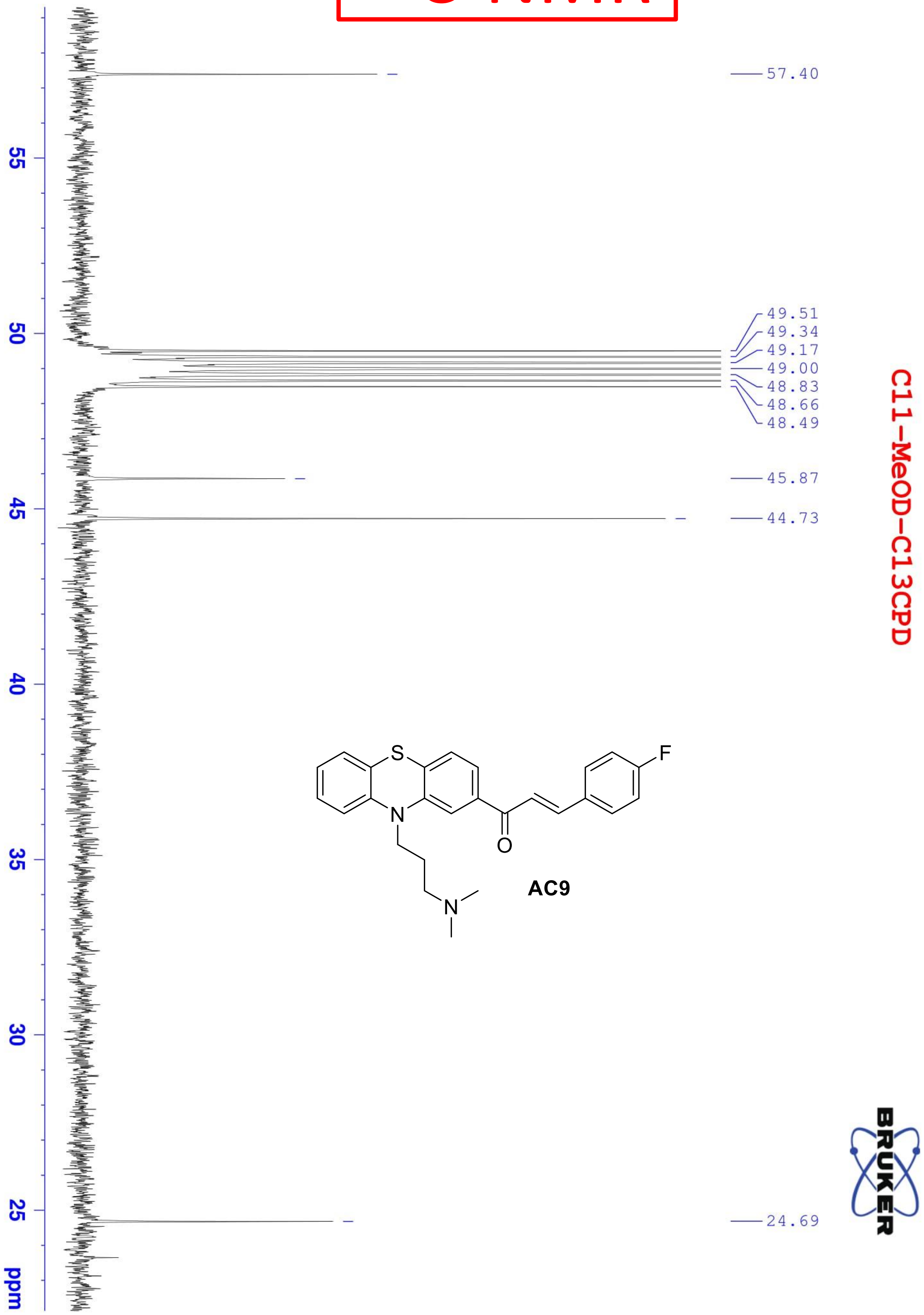
57.40
49.51
49.34
49.17
49.00
48.83
48.66
48.49
45.87
44.73
24.69
55
50
45
40
35
30
25
ppm
C11–MeOD–C13CPD
S
N
O
F
N
BRUKER

AC9

VIEN CONG NGHE HOA HOC
01 Mac Dinh Chi - Q.1 - Tp HCM

IR

Tel: 08.38296127
Fax: 08.38293889

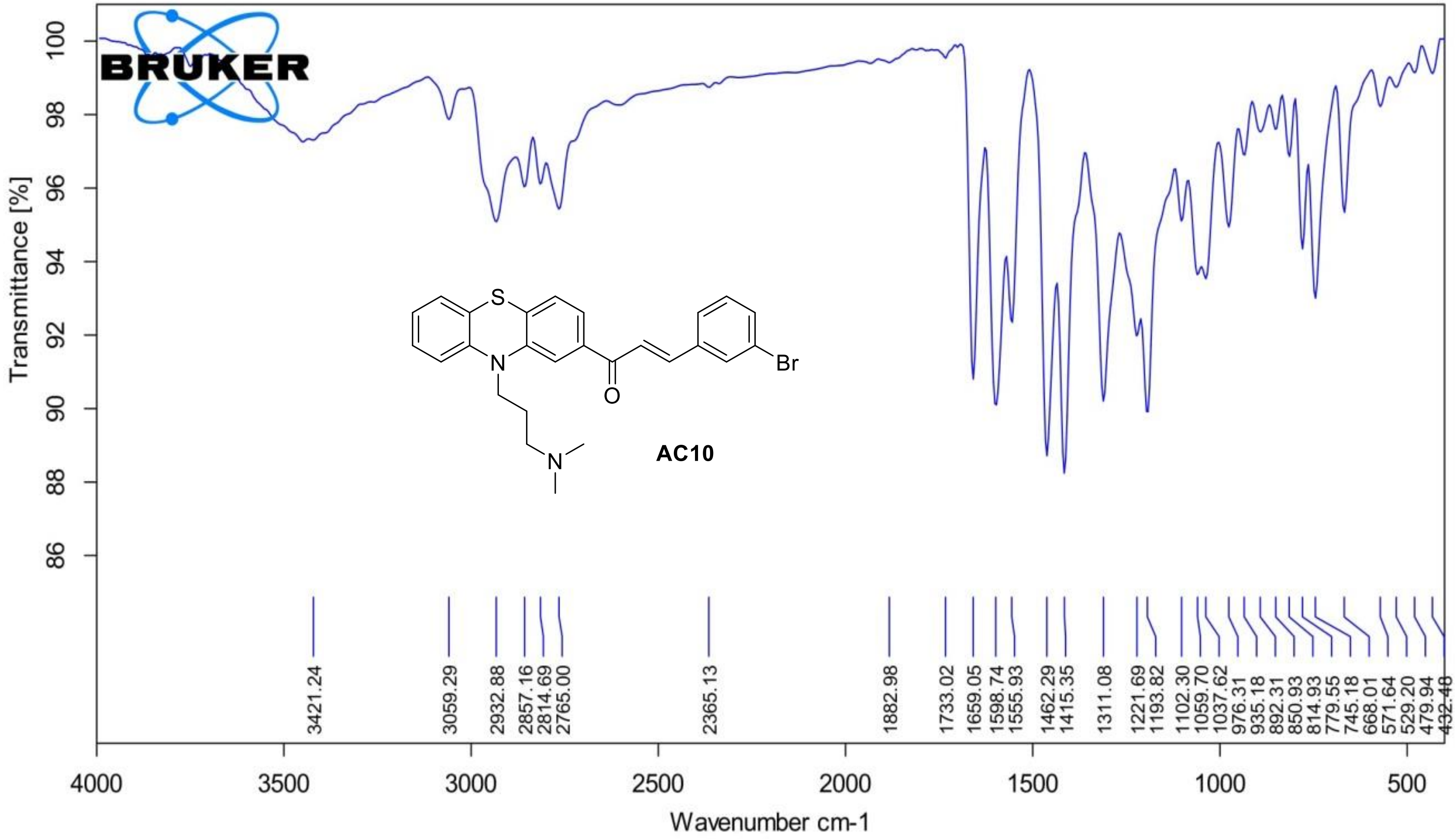

E:\OPUS 7\2017\THANG 7\20170714\C14.0	C14	TENSOR 27 - BRUKER - GERMANY	7/14/2017

MS

Qualitative Analysis Report

**Data Filename**	10-AC10.d	**Sample Name**	10-AC10
**Sample Type**	Sample	**Position**	P2-C1
**Instrument Name**	Instrument 1	**User Name**	
**Acq Method**	Cot ngan - MSMS_Pos.m	**Acquired Time**	22/08/2020 11:30:09 AM
**IRM Calibration Status**	Success	**DA Method**	COTNGAN.M.m
**Comment**			

**Sample Group**		**Info.**	
**Stream Name**	LC 1	**Acquisition SW Version**	6200 series TOF/6500 series Q-TOF B.06.01 (B6172 SP1)

User Chromatograms

Fragmentor Voltage 140 **Collision Energy** 0 **Ionization Mode** ESI

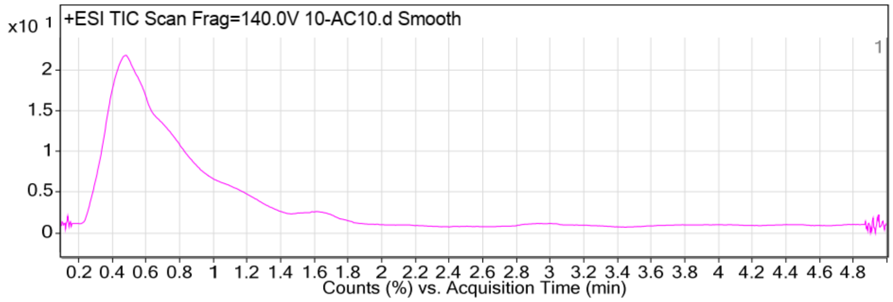

User Spectra

Fragmentor Voltage	Collision Energy	Ionization Mode
140	0	ESI

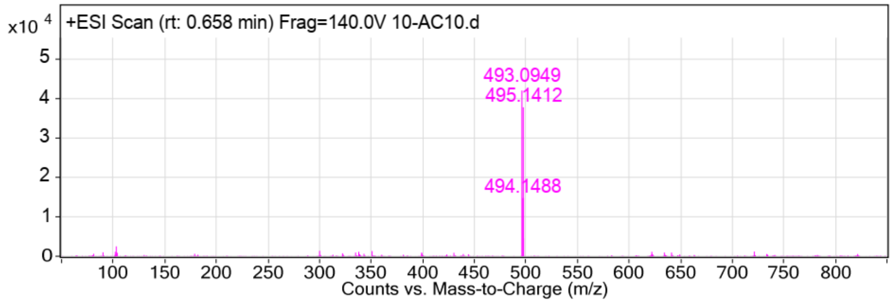

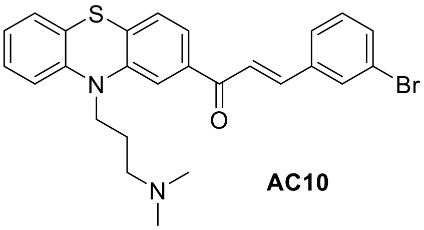

AC10

--- End Of Report ---

MS

Sample Name	10-AC10	Position	P2-C1	Instrument Name	Instrument 1	User Name	
Inj Vol	2	InjPosition		SampleType	Sample	IRM Calibration Status	Success
Data Filename	10-AC10.d	ACQ Method	Cot ngan - MSMS_Pos.	Comment		Acquired Time	22/08/2020 11:30:09 AM

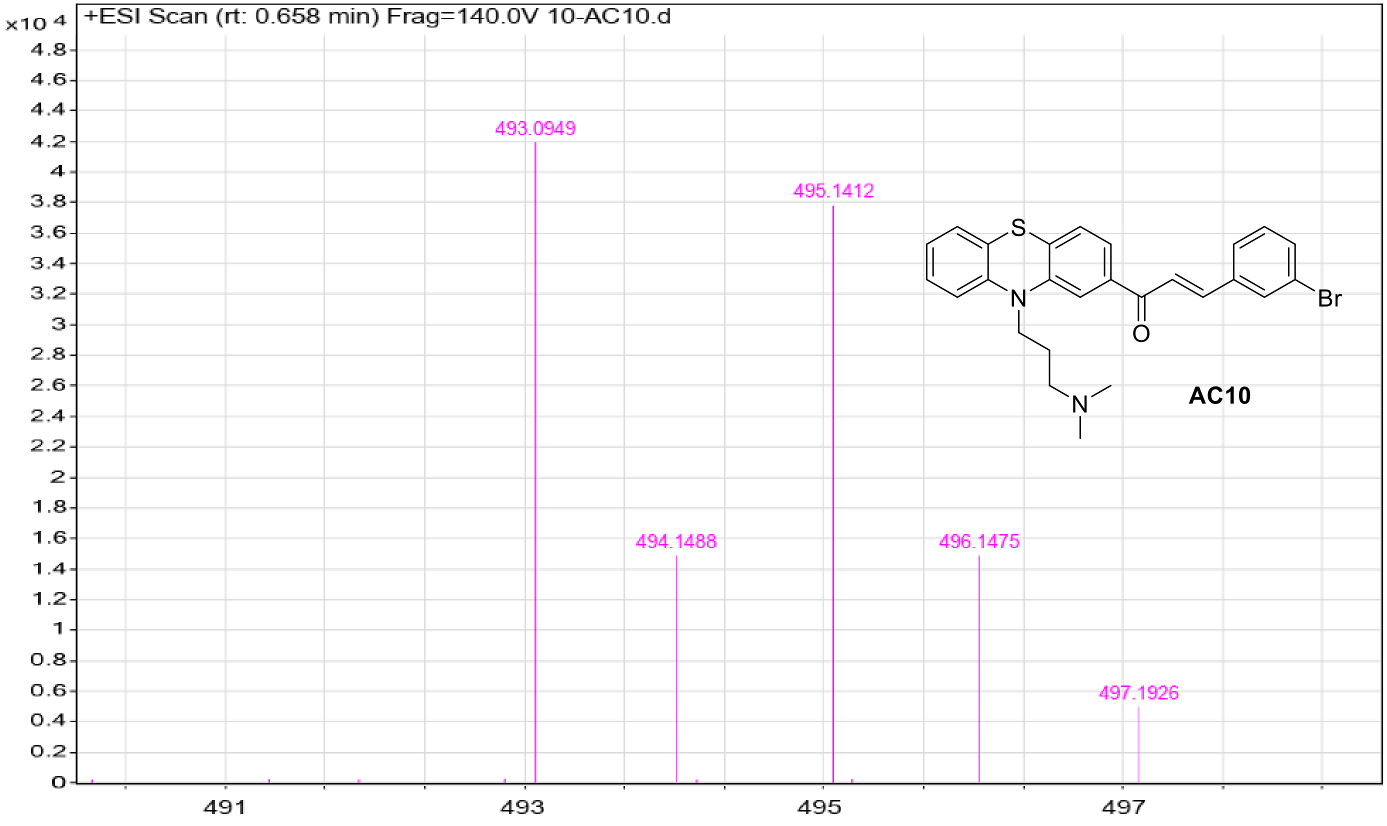

^{1}H-NMR

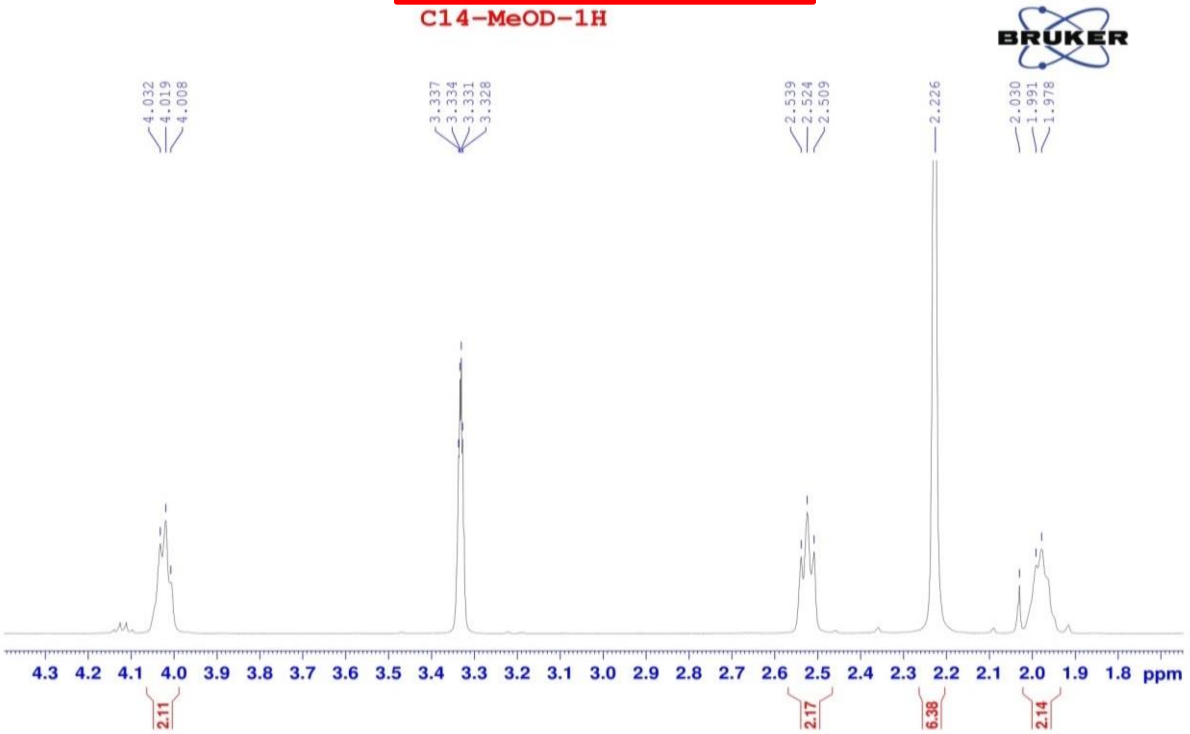

C14-MeOD-1H
BRUKER
4.032
4.019
4.008
3.337
3.334
3.331
3.328
2.539
2.524
2.509
2.226
2.030
1.991
1.978
4.3 4.2 4.1 4.0 3.9 3.8 3.7 3.6 3.5 3.4 3.3 3.2 3.1 3.0 2.9 2.8 2.7 2.6 2.5 2.4 2.3 2.2 2.1 2.0 1.9 1.8 ppm
2.11
2.17
6.38
2.14

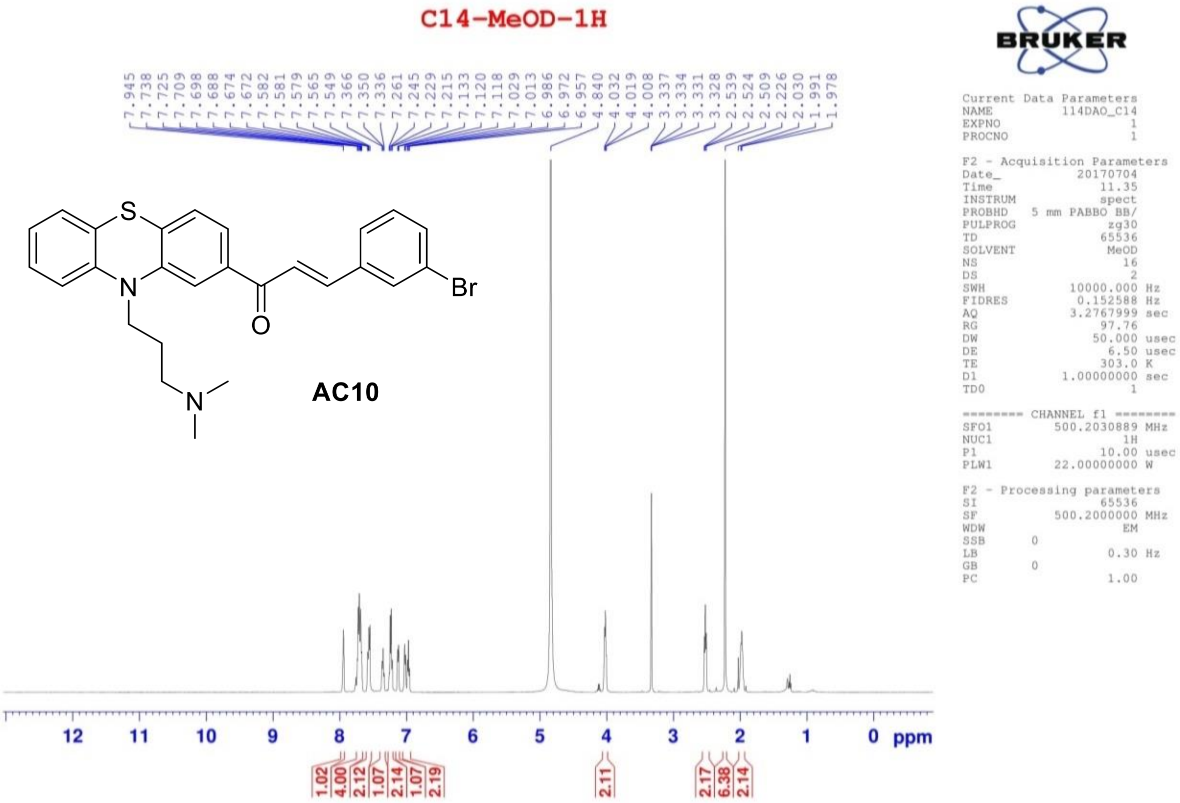

C14-MeOD-1H
BRUKER
7.945
7.738
7.725
7.709
7.698
7.688
7.674
7.672
7.582
7.581
7.579
7.565
7.549
7.366
7.350
7.336
7.261
7.245
7.229
7.215
7.133
7.120
7.118
7.029
7.013
6.986
6.972
6.957
4.840
4.032
4.019
4.008
3.337
3.334
3.331
3.328
2.539
2.524
2.509
2.226
2.030
1.991
1.978
S
N
O
Br
N
AC10
12 11 10 9 8 7 6 5 4 3 2 1 0 ppm
1.02
4.00
2.12
1.07
2.14
1.07
2.19
2.11
2.17
6.38
2.14
Current Data Parameters
NAME 114DAO_C14
EXPNO 1
PROCNO 1
F2 - Acquisition Parameters
Date_ 20170704
Time 11.35
INSTRUM spect
PROBHD 5 mm PABBO BB/
PULPROG zg30
TD 65536
SOLVENT MeOD
NS 16
DS 2
SWH 10000.000 Hz
FIDRES 0.152588 Hz
AQ 3.2767999 sec
RG 97.76
DW 50.000 usec
DE 6.50 usec
TE 303.0 K
D1 1.00000000 sec
TD0 1
======== CHANNEL f1 ========
SFO1 500.2030889 MHz
NUC1 1H
P1 10.00 usec
PLW1 22.00000000 W
F2 - Processing parameters
SI 65536
SF 500.2000000 MHz
WDW EM
SSB 0
LB 0.30 Hz
GB 0
PC 1.00

^{1}H-NMR

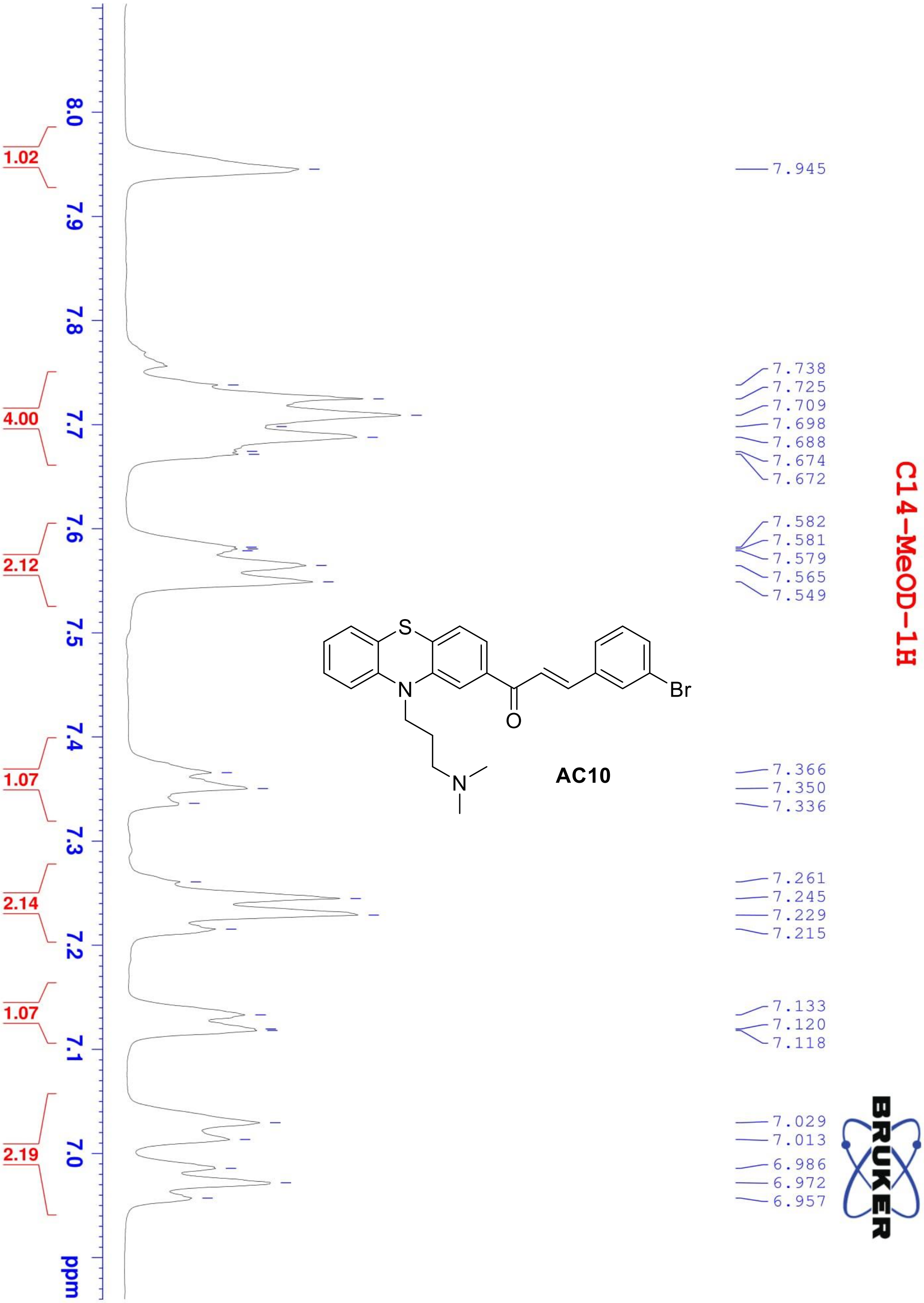
C14-MeOD-1H
AC10
S
N
O
Br
7.945
7.738
7.725
7.709
7.698
7.688
7.674
7.672
7.582
7.581
7.579
7.565
7.549
7.366
7.350
7.336
7.261
7.245
7.229
7.215
7.133
7.120
7.118
7.029
7.013
6.986
6.972
6.957
1.02
4.00
2.12
1.07
2.14
1.07
2.19
8.0
7.9
7.8
7.7
7.6
7.5
7.4
7.3
7.2
7.1
7.0
ppm
BRUKER

^{13}C-NMR

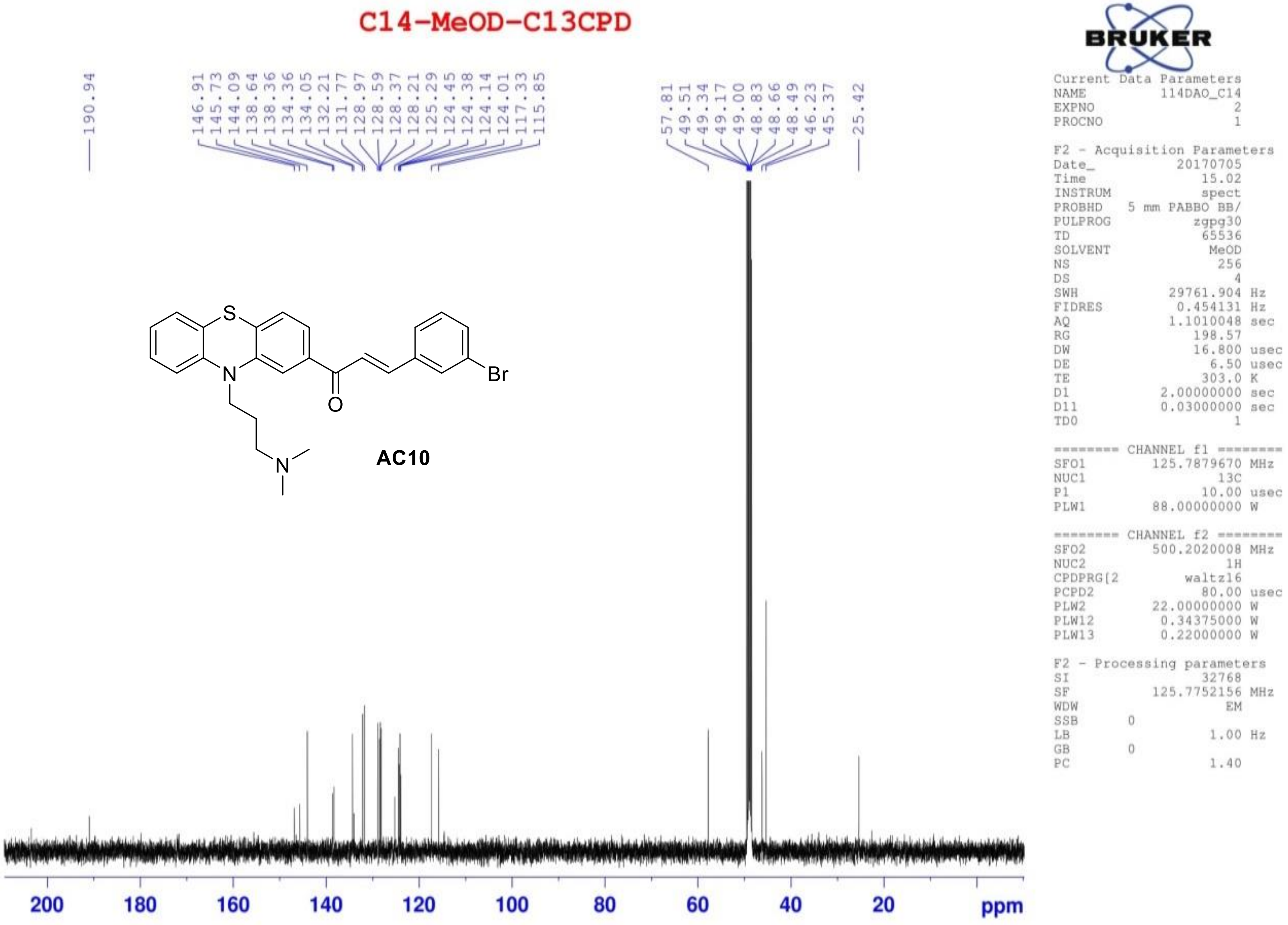
C14-MeOD-C13CPD
190.94
146.91
145.73
144.09
138.64
138.36
134.36
134.05
132.21
131.77
128.97
128.59
128.37
128.21
125.29
124.45
124.38
124.14
124.01
117.33
115.85
57.81
49.51
49.34
49.17
49.00
48.83
48.66
48.49
46.23
45.37
25.42
S
N
O
Br
AC10
200
180
160
140
120
100
80
60
40
20
ppm
BRUKER
Current Data Parameters
NAME 114DAO_C14
EXPNO 2
PROCNO 1
F2 - Acquisition Parameters
Date_ 20170705
Time 15.02
INSTRUM spect
PROBHD 5 mm PABBO BB/
PULPROG zgpg30
TD 65536
SOLVENT MeOD
NS 256
DS 4
SWH 29761.904 Hz
FIDRES 0.454131 Hz
AQ 1.1010048 sec
RG 198.57
DW 16.800 usec
DE 6.50 usec
TE 303.0 K
D1 2.00000000 sec
D11 0.03000000 sec
TD0 1
======== CHANNEL f1 ========
SFO1 125.7879670 MHz
NUC1 13C
P1 10.00 usec
PLW1 88.00000000 W
======== CHANNEL f2 ========
SFO2 500.2020008 MHz
NUC2 1H
CPDPRG[2 waltz16
PCPD2 80.00 usec
PLW2 22.00000000 W
PLW12 0.34375000 W
PLW13 0.22000000 W
F2 - Processing parameters
SI 32768
SF 125.7752156 MHz
WDW EM
SSB 0
LB 1.00 Hz
GB 0
PC 1.40

^{13}C-NMR

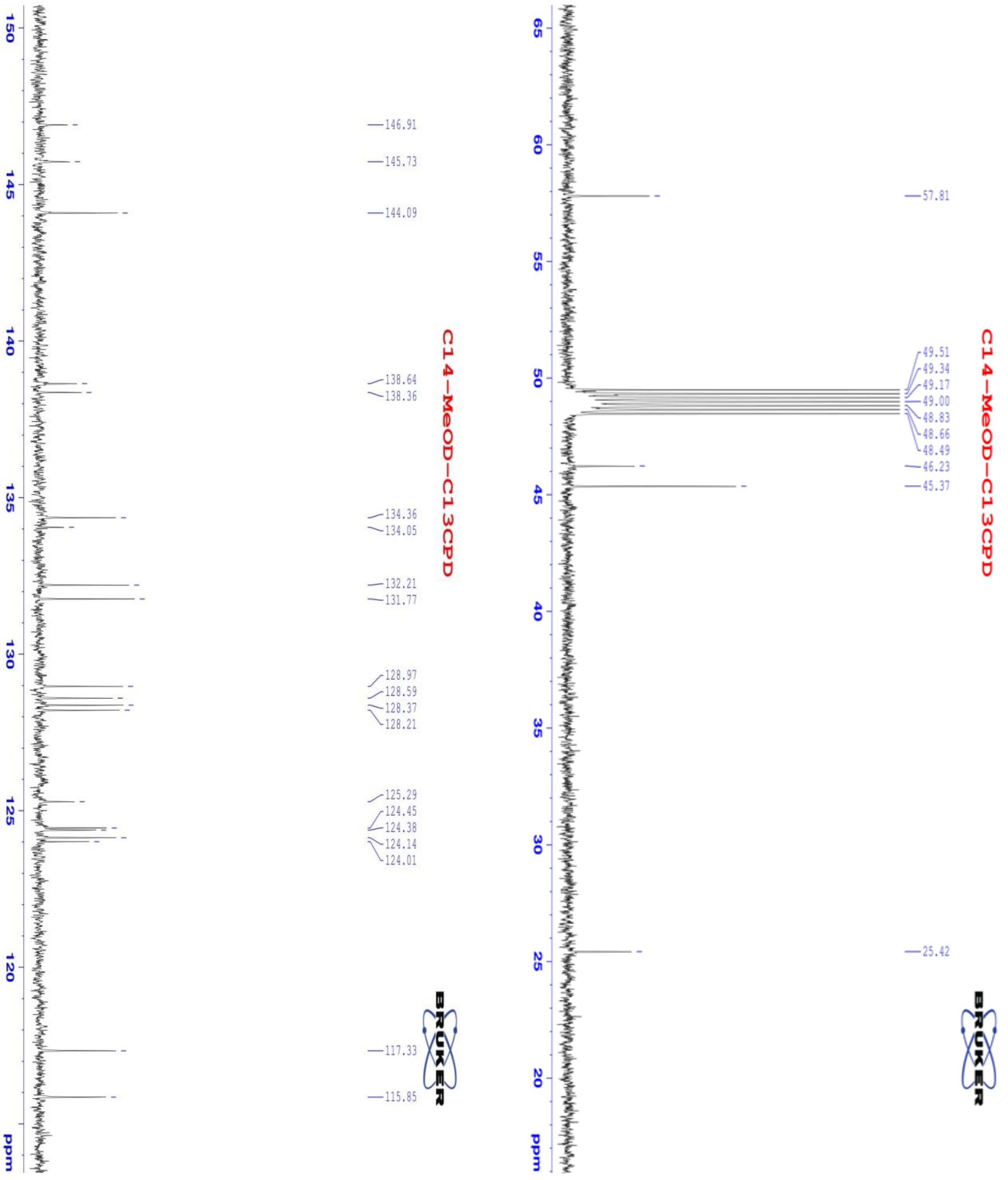

146.91
145.73
144.09
138.64
138.36
134.36
134.05
132.21
131.77
128.97
128.59
128.37
128.21
125.29
124.45
124.38
124.14
124.01
117.33
115.85
C14-MeOD-C13CPD
57.81
49.51
49.34
49.17
49.00
48.83
48.66
48.49
46.23
45.37
25.42
C14-MeOD-C13CPD
ppm
BRUKER

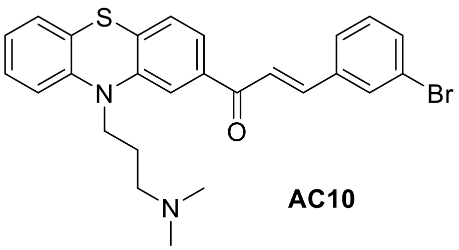

S
N
O
Br
N

AC10

IR

VIEN CONG NGHE HOA HOC
01 Mac Dinh Chi - Q.1 - Tp HCM

Tel: 08.38296127
Fax: 08.38293889

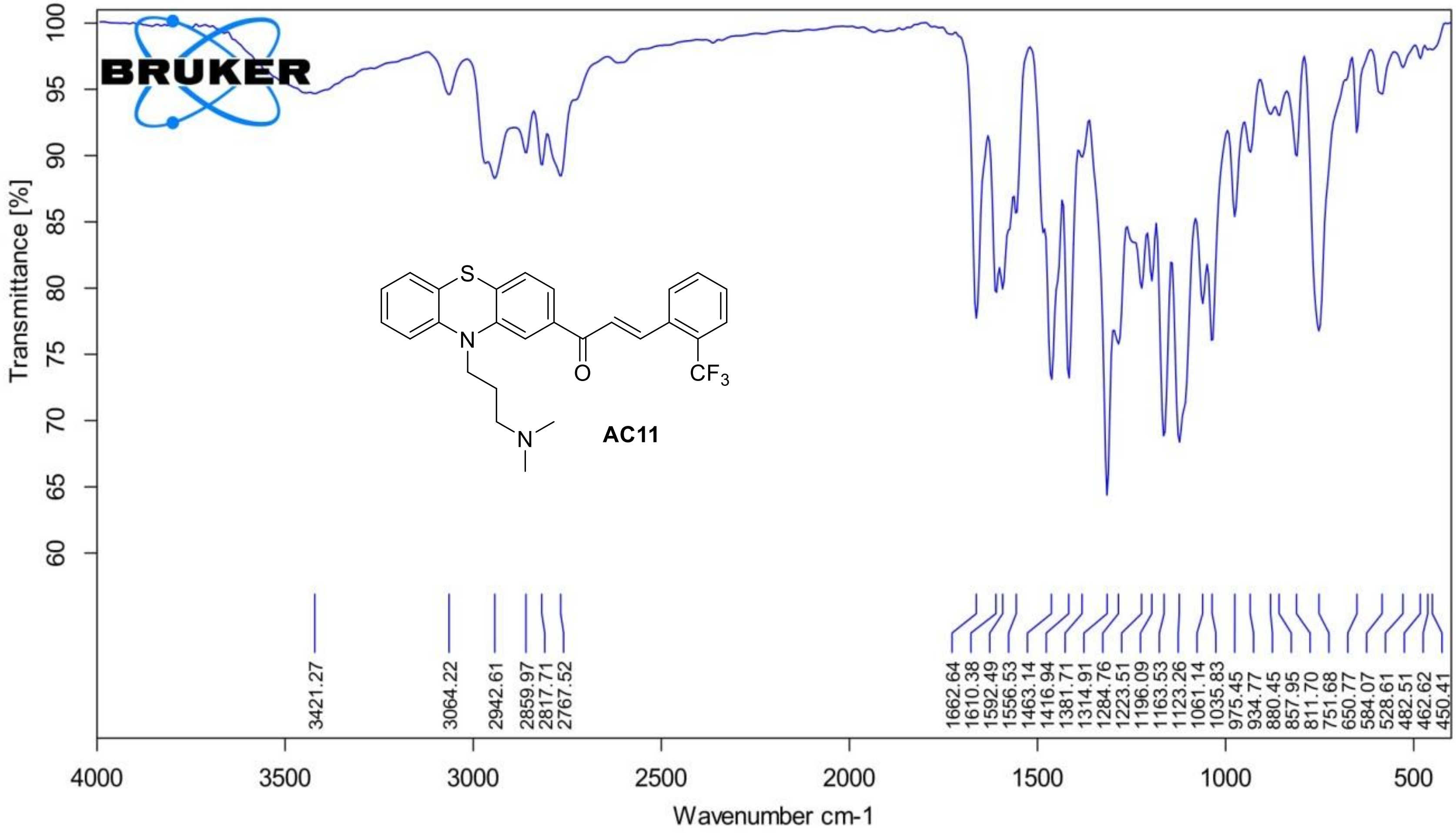

E:\OPUS 7\2017\THANG 7\20170714\C15.0	C15	TENSOR 27 - BRUKER - GERMANY	7/14/2017

MS

Qualitative Analysis Report

**Data Filename**	11-AC11.d	**Sample Name**	11-AC11
**Sample Type**	Sample	**Position**	P2-B1
**Instrument Name**	Instrument 1	**User Name**	
**Acq Method**	Cot ngan - MSMS_Pos.m	**Acquired Time**	22/08/2020 11:40:04 AM
**IRM Calibration Status**	Success	**DA Method**	COTNGAN.M.m
**Comment**			

**Sample Group**		**Info.**	
**Stream Name**	LC 1	**Acquisition SW Version**	6200 series TOF/6500 series Q-TOF B.06.01 (B6172 SP1)

User Chromatograms

Fragmentor Voltage 140 **Collision Energy** 0 **Ionization Mode** ESI

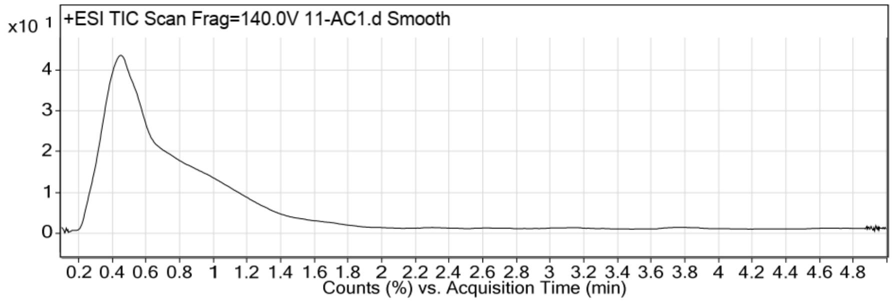

User Spectra

Fragmentor Voltage	Collision Energy	Ionization Mode
140	0	ESI

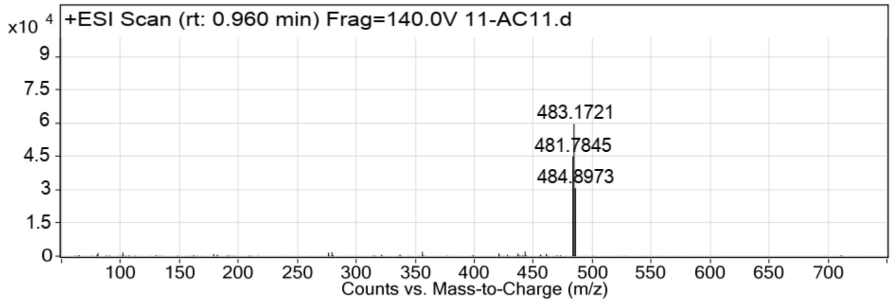

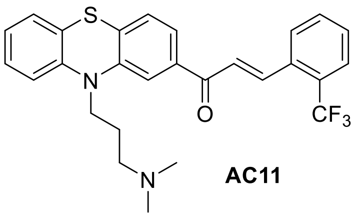

AC11

--- End Of Report ---

MS

**Sample Name**	11-AC11	**Position**	P2-B1	**Instrument Name**	Instrument 1	**User Name**	
**Inj Vol**	2	**InjPosition**		**SampleType**	Sample	**IRM Calibration Status**	Success
**Data Filename**	11-AC11.d	**ACQ Method**	Cot ngan - MSMS_Pos.	**Comment**		**Acquired Time**	22/08/2020 11:40:04 AM

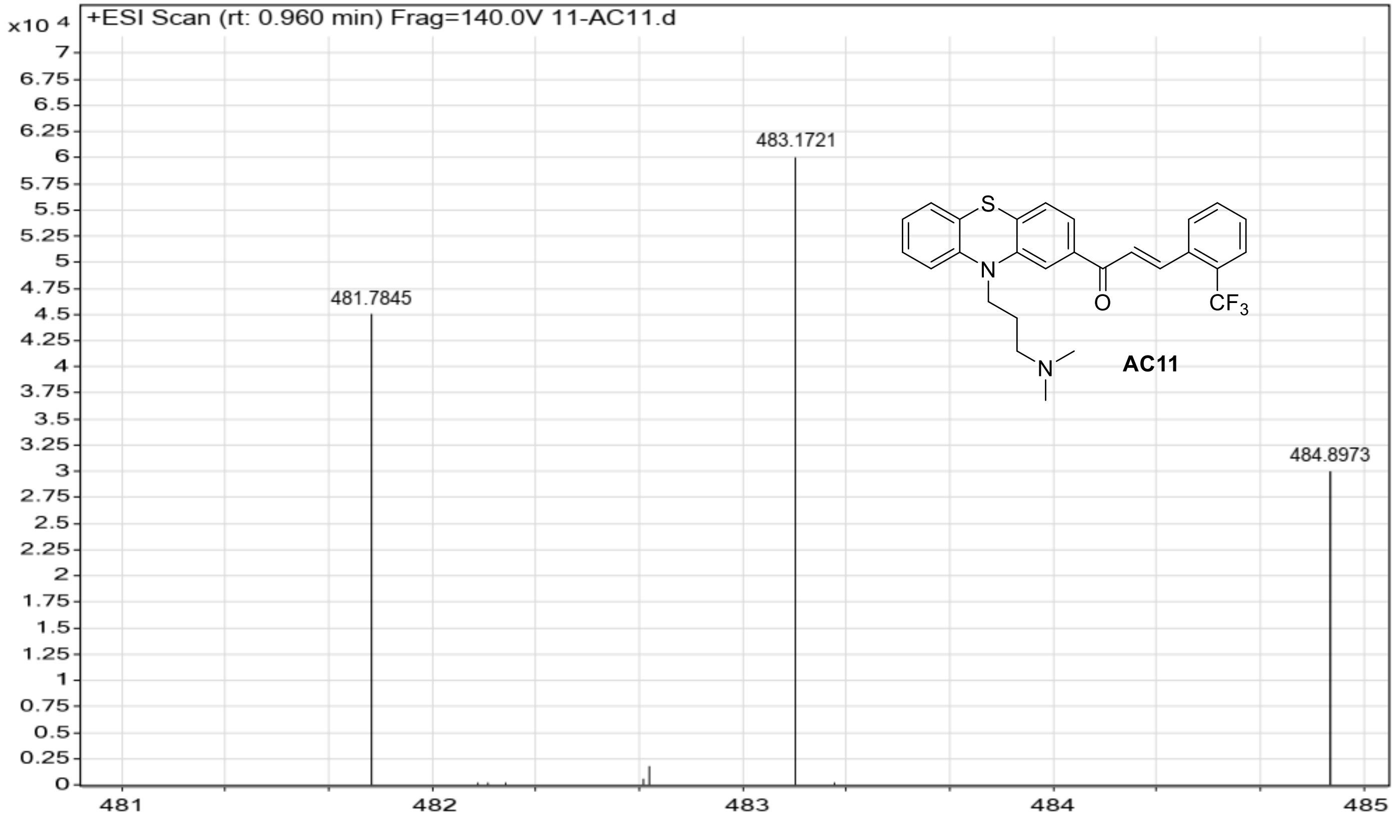

^{1}H-NMR

C15-MeOD-1H

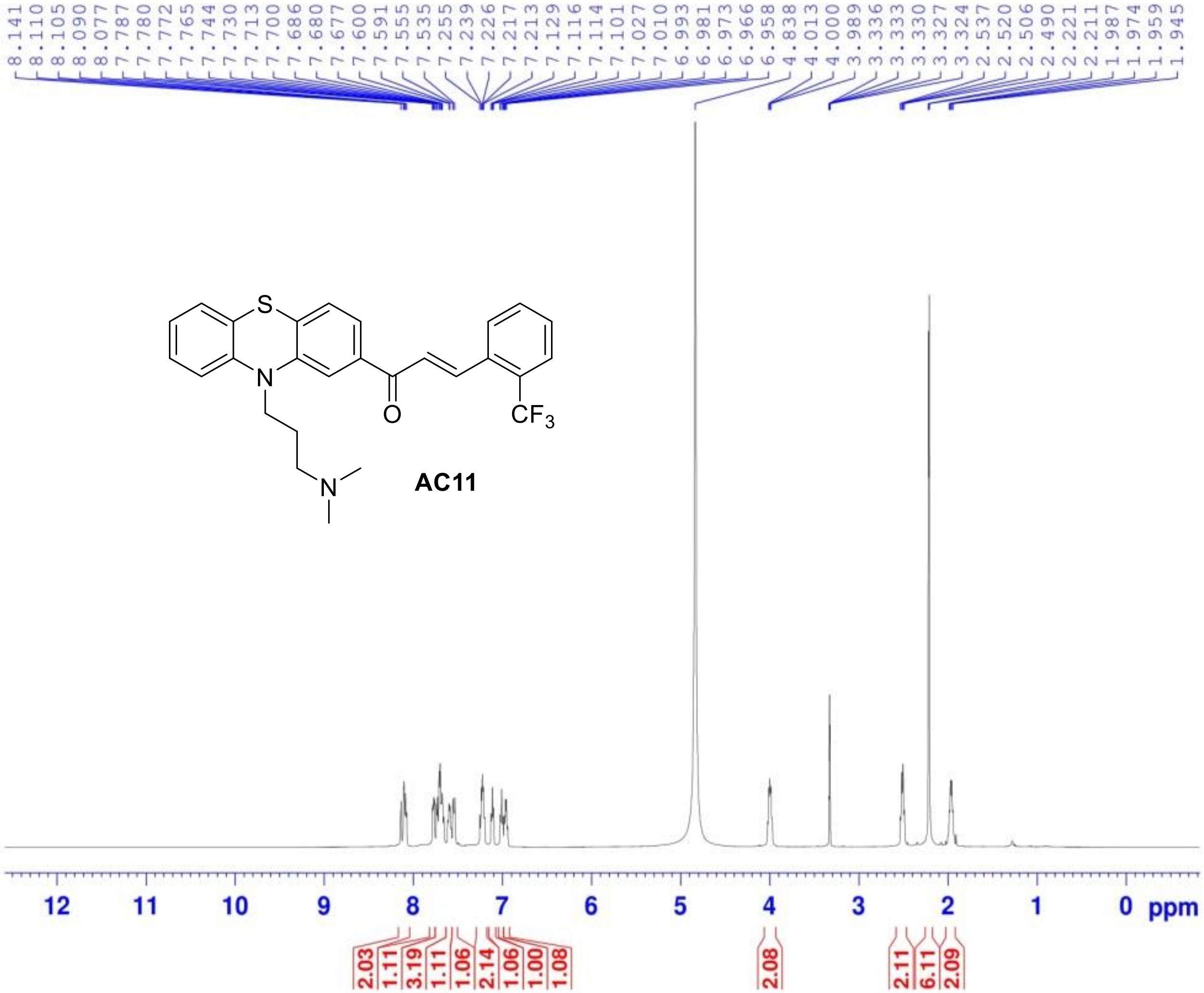


```
Current Data Parameters
NAME          113DAO_C15
EXPNO                  1
PROCNO                 1

F2 - Acquisition Parameters
Date_           20170705
Time               15.07
INSTRUM            spect
PROBHD   5 mm PABBO BB/
PULPROG             zg30
TD                 65536
SOLVENT             MeOD
NS                    16
DS                     2
SWH          10000.000 Hz
FIDRES        0.152588 Hz
AQ           3.2767999 sec
RG               79.36
DW              50.000 usec
DE               6.50 usec
TE              303.0 K
D1         1.00000000 sec
TD0                    1

======== CHANNEL f1 ========
SFO1       500.2030889 MHz
NUC1                  1H
P1               10.00 usec
PLW1       22.00000000 W

F2 - Processing parameters
SI                 65536
SF         500.2000011 MHz
WDW                   EM
SSB      0
LB                0.30 Hz
GB       0
PC                  1.00
```

^{1}H-NMR

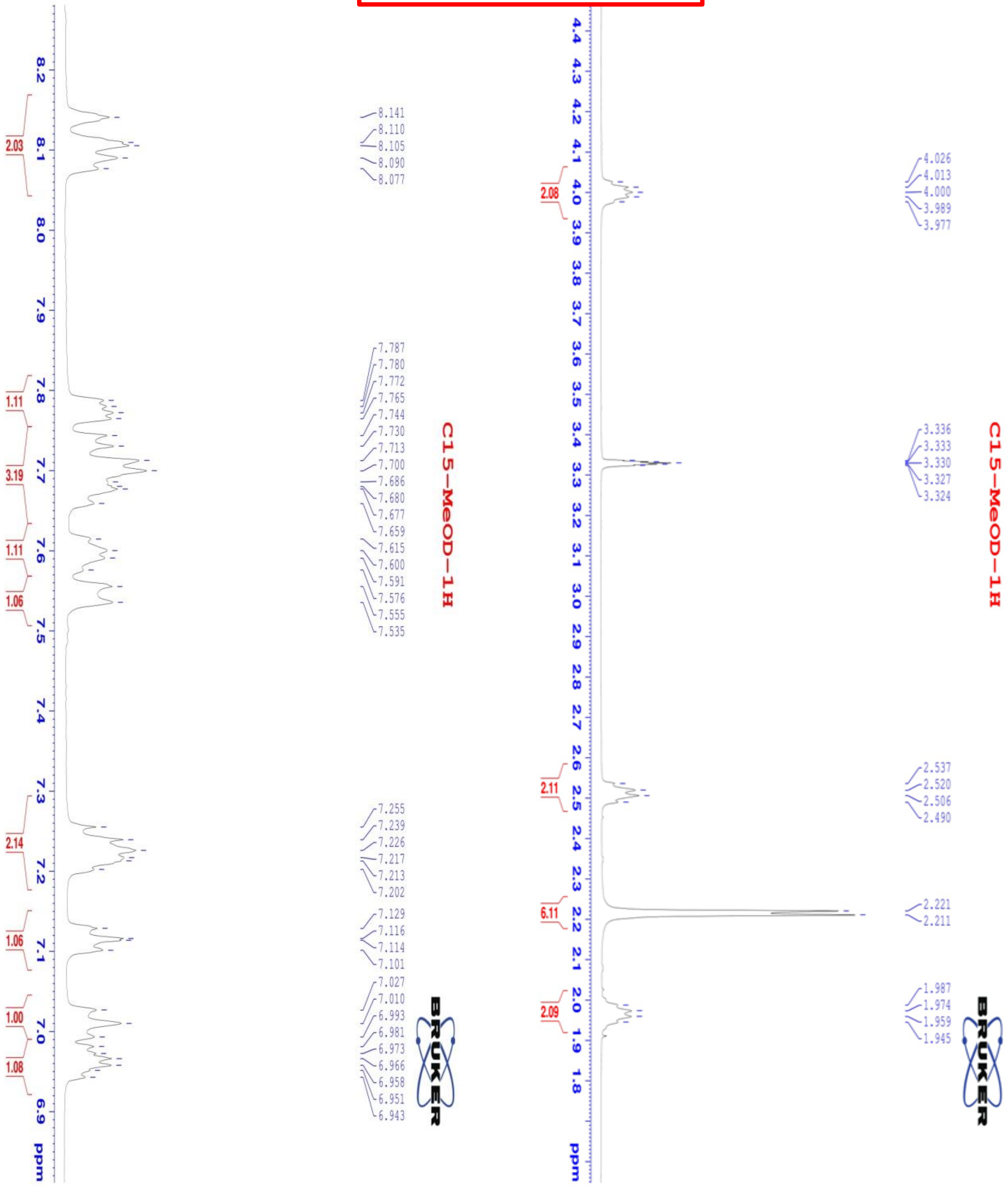
C15−MeOD−1H
8.141
8.110
8.105
8.090
8.077
7.787
7.780
7.772
7.765
7.744
7.730
7.713
7.700
7.686
7.680
7.677
7.659
7.615
7.600
7.591
7.576
7.555
7.535
7.255
7.239
7.226
7.217
7.213
7.202
7.129
7.116
7.114
7.101
7.027
7.010
6.993
6.981
6.973
6.966
6.958
6.951
6.943
4.026
4.013
4.000
3.989
3.977
3.336
3.333
3.330
3.327
3.324
2.537
2.520
2.506
2.490
2.221
2.211
1.987
1.974
1.959
1.945
ppm

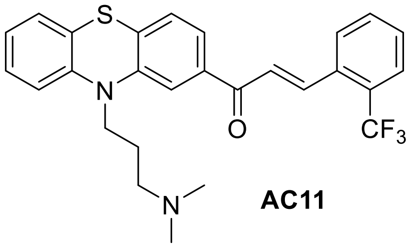
S
N
O
CF3
N

AC11

^{13}C-NMR

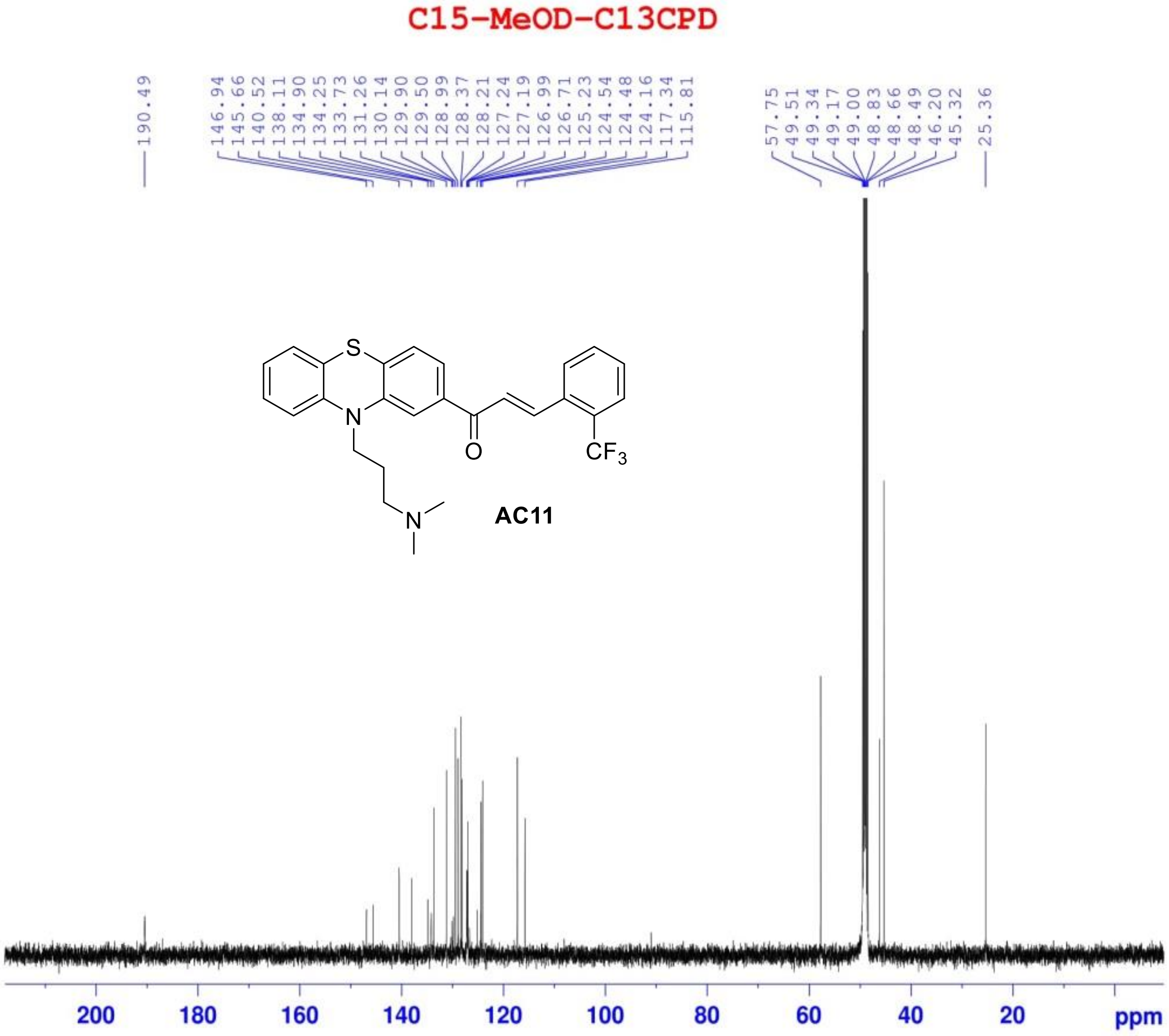

Current Data Parameters
NAME 113DAO_C15
EXPNO 2
PROCNO 1

F2 - Acquisition Parameters
Date_ 20170705
Time 15.23
INSTRUM spect
PROBHD 5 mm PABBO BB/
PULPROG zgpg30
TD 65536
SOLVENT MeOD
NS 256
DS 4
SWH 29761.904 Hz
FIDRES 0.454131 Hz
AQ 1.1010048 sec
RG 198.57
DW 16.800 usec
DE 6.50 usec
TE 303.0 K
D1 2.00000000 sec
D11 0.03000000 sec
TD0 1

======== CHANNEL f1 ========
SFO1 125.7879670 MHz
NUC1 13C
P1 10.00 usec
PLW1 88.00000000 W

======== CHANNEL f2 ========
SFO2 500.2020008 MHz
NUC2 1H
CPDPRG[2 waltz16
PCPD2 80.00 usec
PLW2 22.00000000 W
PLW12 0.34375000 W
PLW13 0.22000000 W

F2 - Processing parameters
SI 32768
SF 125.7752168 MHz
WDW EM
SSB 0
LB 1.00 Hz
GB 0
PC 1.40

^{13}C-NMR

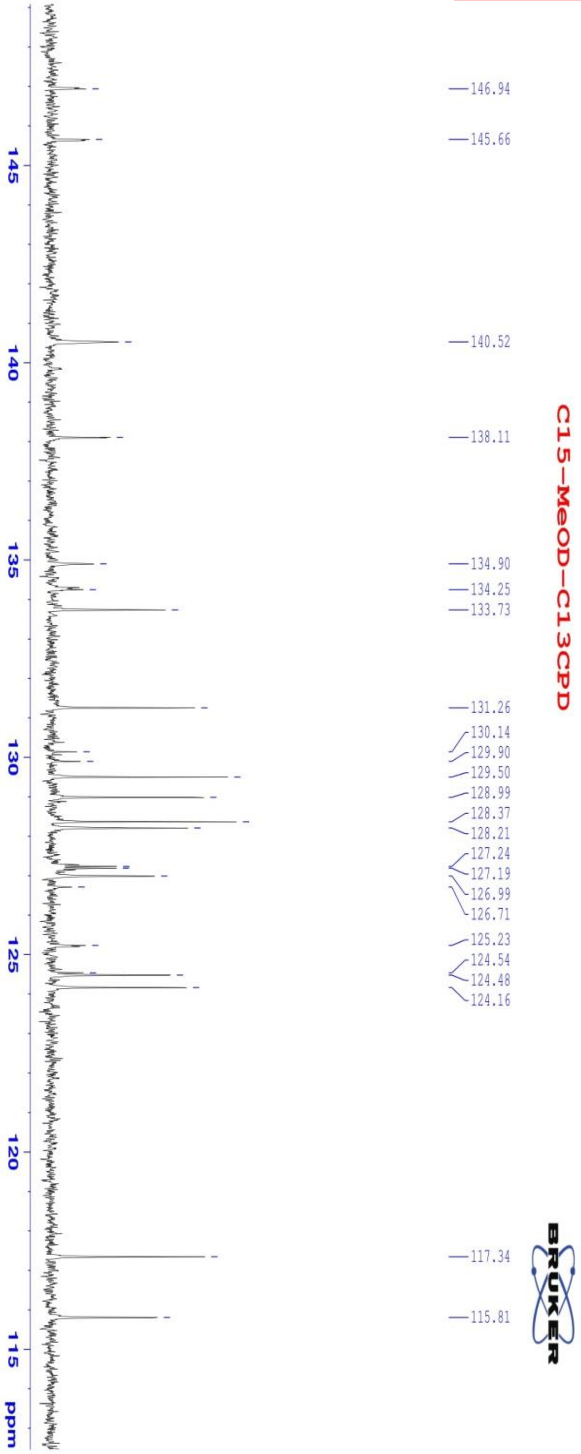
146.94
145.66
140.52
138.11
134.90
134.25
133.73
131.26
130.14
129.90
129.50
128.99
128.37
128.21
127.24
127.19
126.99
126.71
125.23
124.54
124.48
124.16
117.34
115.81
C15–MeOD–C13CPD
ppm

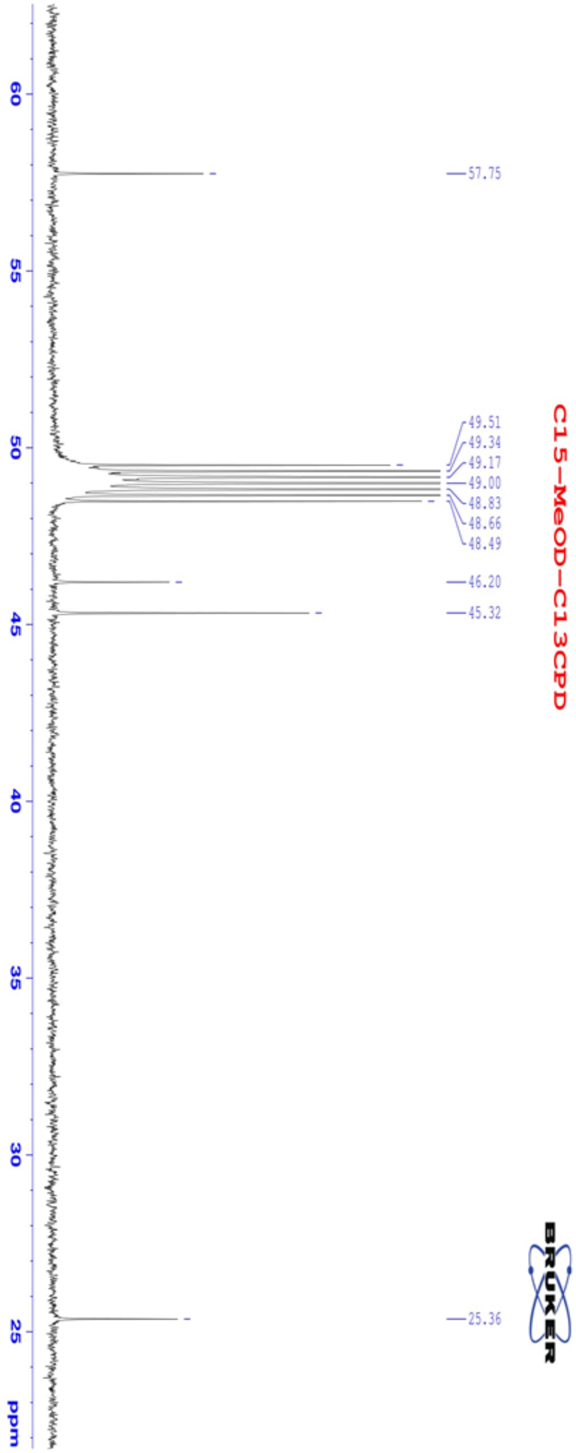
57.75
49.51
49.34
49.17
49.00
48.83
48.66
48.49
46.20
45.32
25.36
C15–MeOD–C13CPD
ppm

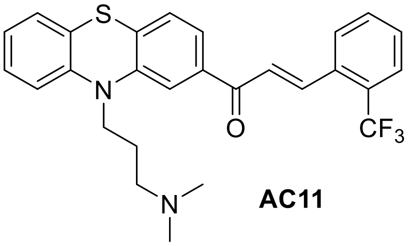
S
N
N
O
CF_3

AC11

VIEN CONG NGHE HOA HOC
01 Mac Dinh Chi - Q.1 - Tp HCM

Tel: 08.38296127
Fax: 08.38293889

IR

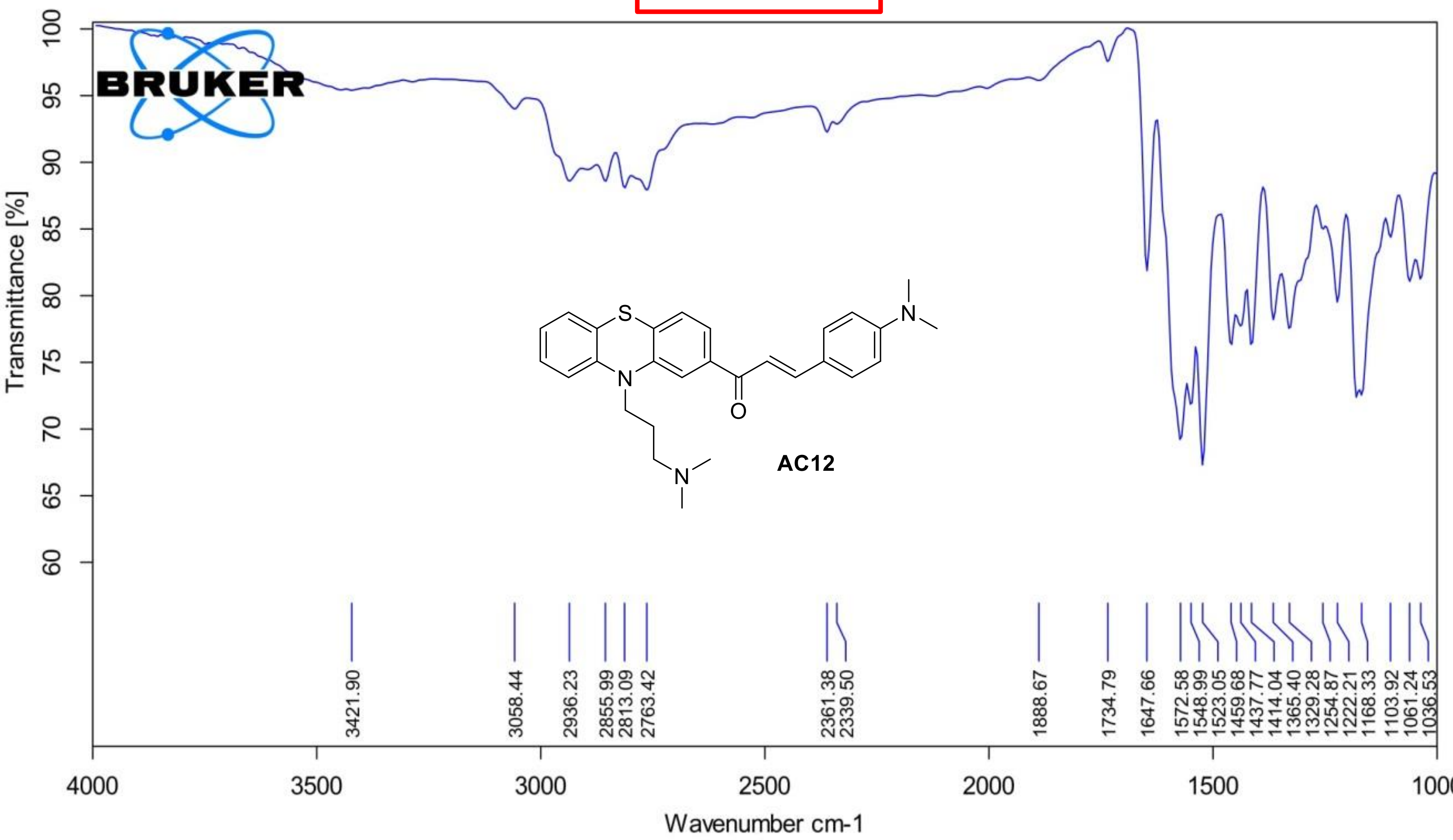

E:\OPUS 7\2017\THANG 6\20170606\C7.0	C7	TENSOR 27 - BRUKER - GERMANY	6/8/2017

MS

Qualitative Analysis Report

**Data Filename**	12-AC12.d	**Sample Name**	12-AC12
**Sample Type**	Sample	**Position**	P2-B9
**Instrument Name**	Instrument 1	**User Name**	
**Acq Method**	Cot ngan - MSMS_Pos.m	**Acquired Time**	22/08/2020 11:50:36 AM
**IRM Calibration Status**	Success	**DA Method**	COTNGAN.M.m
**Comment**			

**Sample Group**		**Info.**	
**Stream Name**	LC 1	**Acquisition SW Version**	6200 series TOF/6500 series Q-TOF B.06.01 (B6172 SP1)

User Chromatograms

Fragmentor Voltage 140 **Collision Energy** 0 **Ionization Mode** ESI

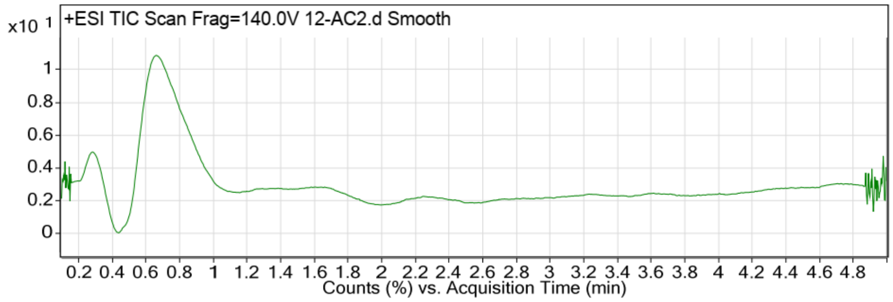

User Spectra

Fragmentor Voltage	Collision Energy	Ionization Mode
140	0	ESI

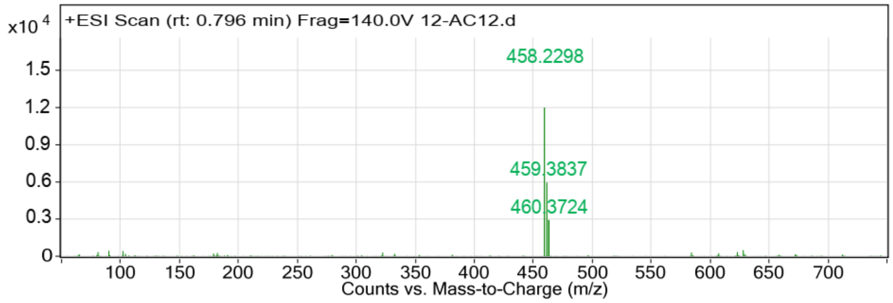

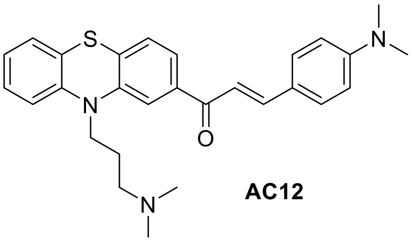

AC12

--- End Of Report ---

MS

**Sample Name**	12-AC12	**Position**	P2-B9	**Instrument Name**	Instrument 1	**User Name**	
**Inj Vol**	2	**InjPosition**		**SampleType**	Sample	**IRM Calibration Status**	Success
**Data Filename**	12-AC12.d	**ACQ Method**	Cot ngan - MSMS_Pos.	**Comment**		**Acquired Time**	22/08/2020 11:50:36 AM

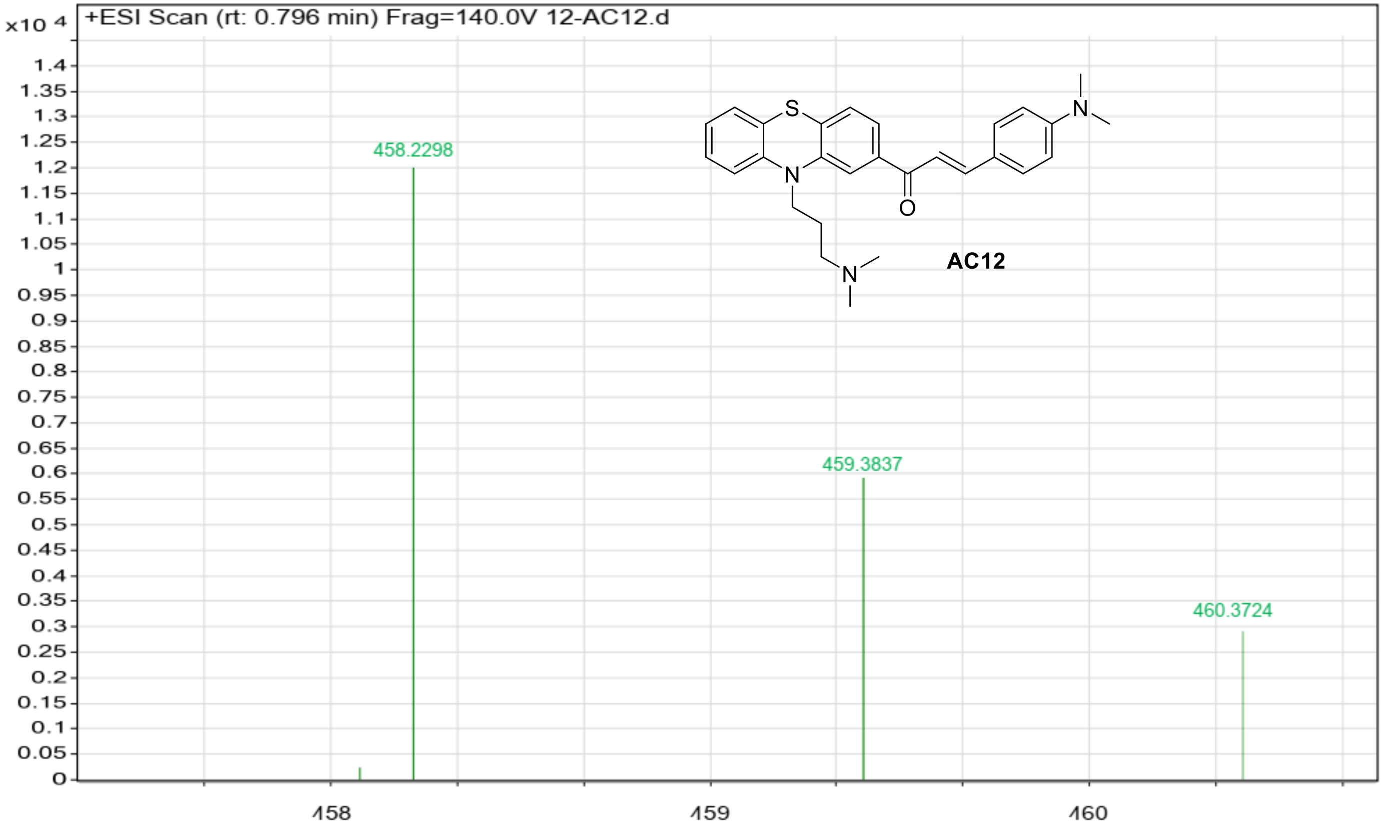

^{1}H-NMR

C7-MeOD-1H

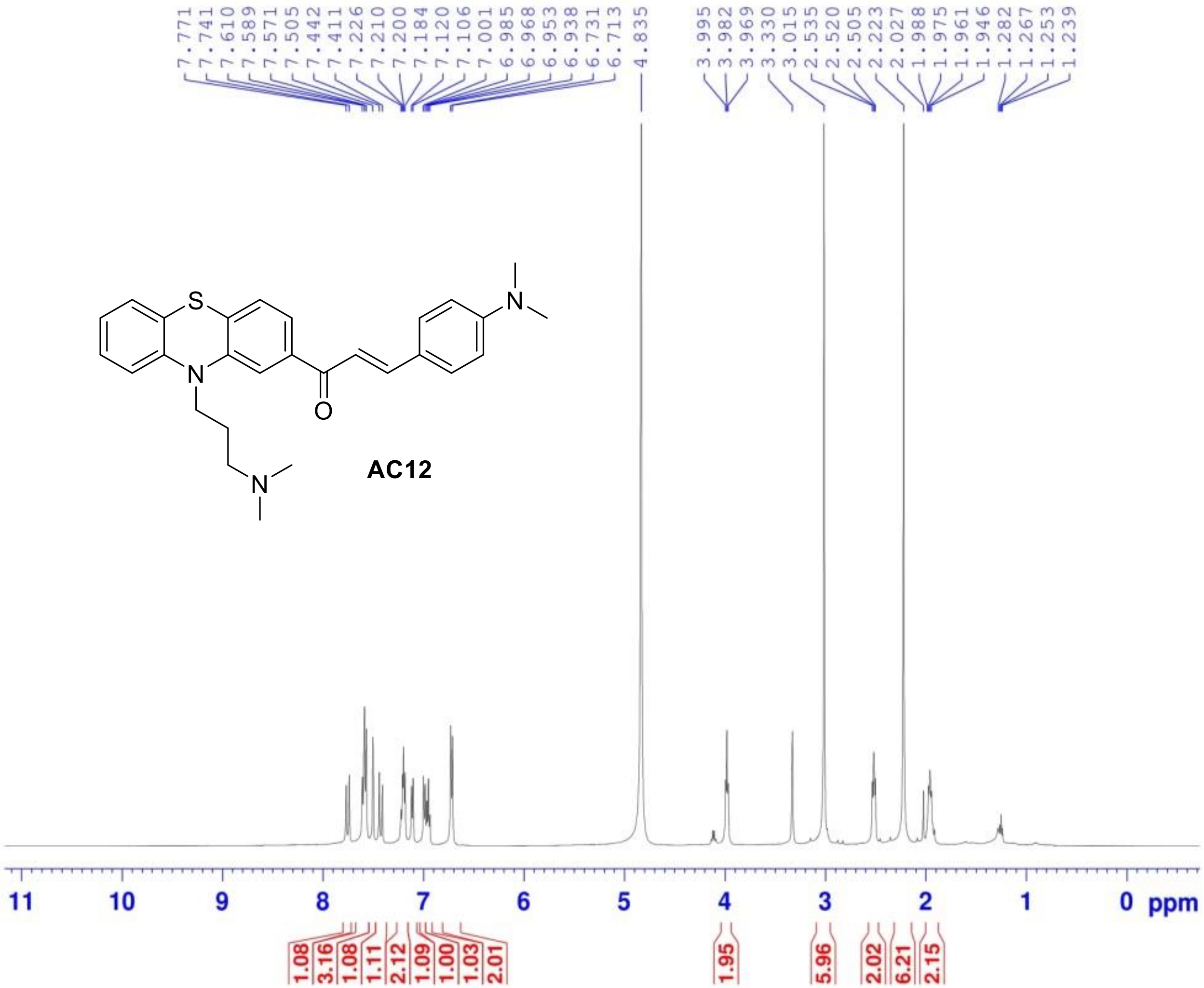


```
Current Data Parameters
NAME             113DAO_C7
EXPNO                    1
PROCNO                   1

F2 - Acquisition Parameters
Date_             20170704
Time                 16.50
INSTRUM              spect
PROBHD    5 mm PABBO BB/
PULPROG               zg30
TD                   65536
SOLVENT               MeOD
NS                      16
DS                       2
SWH             10000.000 Hz
FIDRES           0.152588 Hz
AQ              3.2767999 sec
RG                   79.36
DW                 50.000 usec
DE                   6.50 usec
TE                  303.0 K
D1             1.00000000 sec
TD0                      1

======== CHANNEL f1 ========
SFO1          500.2030889 MHz
NUC1                    1H
P1                   10.00 usec
PLW1           22.00000000 W

F2 - Processing parameters
SI                   65536
SF            500.2000020 MHz
WDW                     EM
SSB      0
LB                    0.30 Hz
GB       0
PC                    1.00
```

^{1}H-NMR

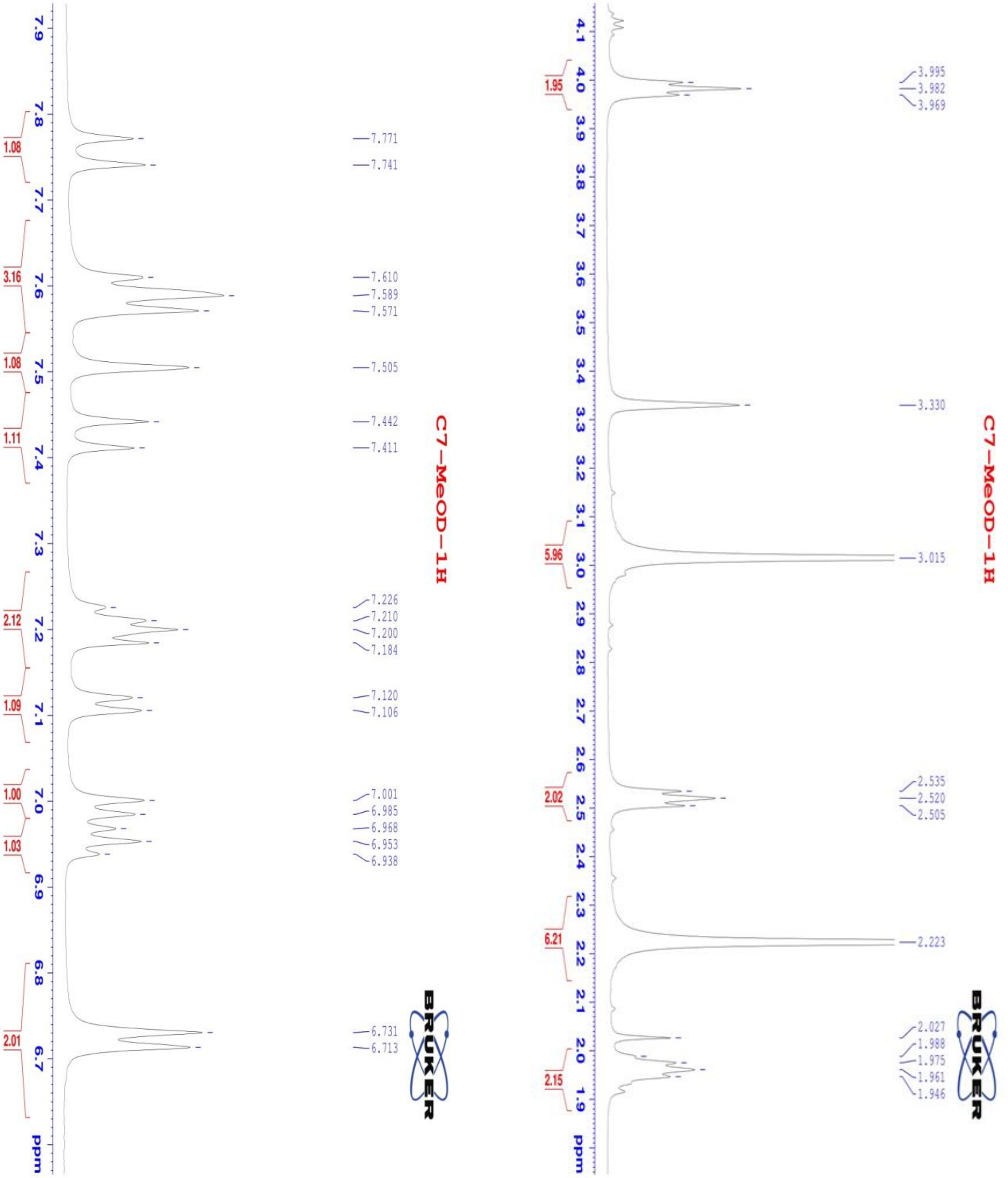
C7−MeOD−1H
7.771
7.741
7.610
7.589
7.571
7.505
7.442
7.411
7.226
7.210
7.200
7.184
7.120
7.106
7.001
6.985
6.968
6.953
6.938
6.731
6.713
1.08
3.16
1.08
1.11
2.12
1.09
1.00
1.03
2.01
ppm
C7−MeOD−1H
3.995
3.982
3.969
3.330
3.015
2.535
2.520
2.505
2.223
2.027
1.988
1.975
1.961
1.946
1.95
5.96
2.02
6.21
2.15
ppm

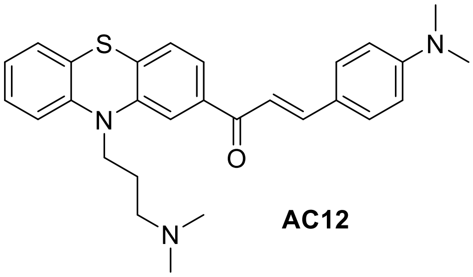
S
N
N
N
O

AC12

^{13}C-NMR

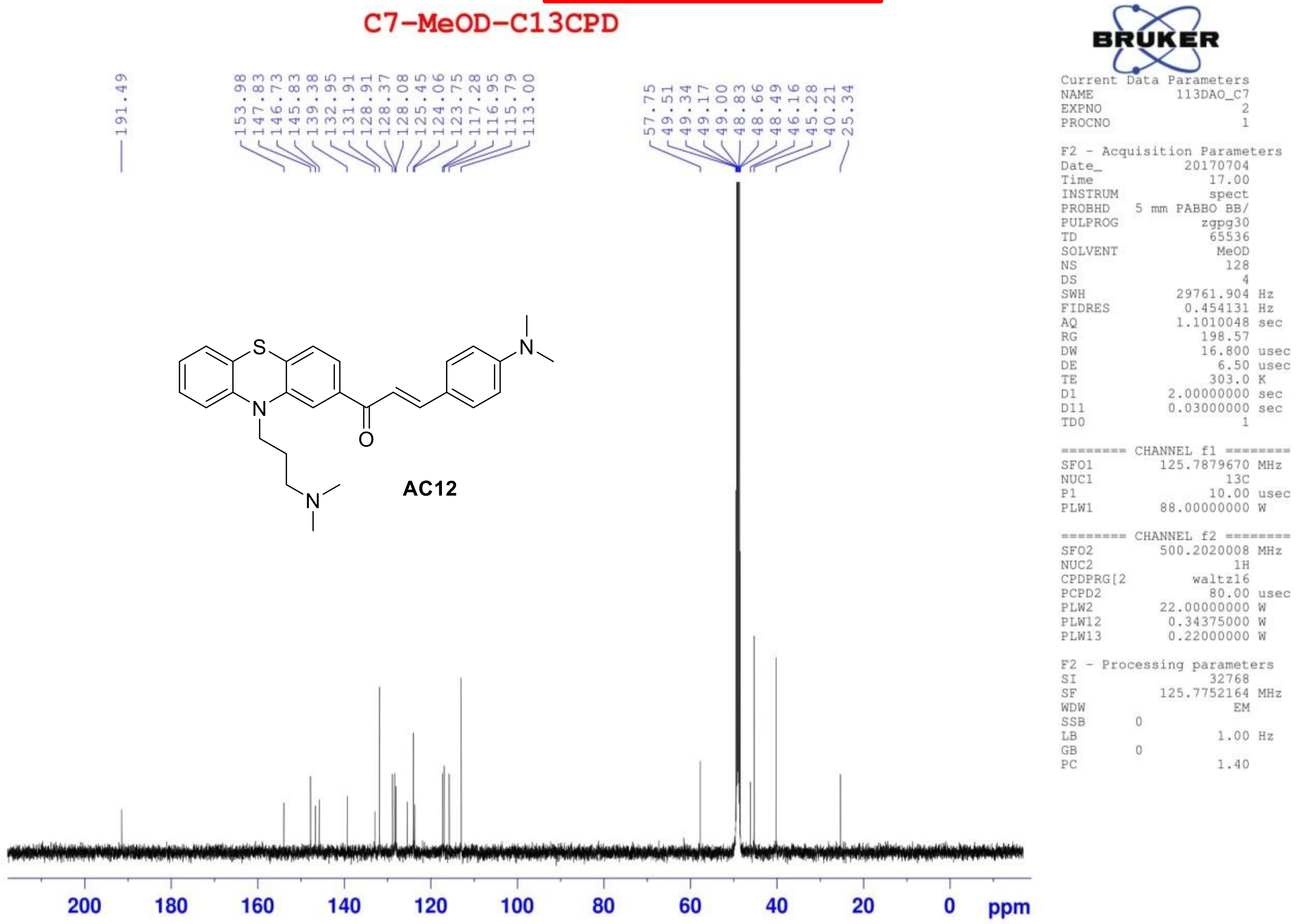

C7-MeOD-C13CPD
191.49
153.98
147.83
146.73
145.83
139.38
132.95
131.91
128.91
128.37
128.08
125.45
124.06
123.75
117.28
116.95
115.79
113.00
57.75
49.51
49.34
49.17
49.00
48.83
48.66
48.49
46.16
45.28
40.21
25.34
S
N
O
N
N
AC12
200 180 160 140 120 100 80 60 40 20 0 ppm
BRUKER
Current Data Parameters
NAME 113DAO_C7
EXPNO 2
PROCNO 1
F2 - Acquisition Parameters
Date_ 20170704
Time 17.00
INSTRUM spect
PROBHD 5 mm PABBO BB/
PULPROG zgpg30
TD 65536
SOLVENT MeOD
NS 128
DS 4
SWH 29761.904 Hz
FIDRES 0.454131 Hz
AQ 1.1010048 sec
RG 198.57
DW 16.800 usec
DE 6.50 usec
TE 303.0 K
D1 2.00000000 sec
D11 0.03000000 sec
TD0 1
======== CHANNEL f1 ========
SFO1 125.7879670 MHz
NUC1 13C
P1 10.00 usec
PLW1 88.00000000 W
======== CHANNEL f2 ========
SFO2 500.2020008 MHz
NUC2 1H
CPDPRG[2 waltz16
PCPD2 80.00 usec
PLW2 22.00000000 W
PLW12 0.34375000 W
PLW13 0.22000000 W
F2 - Processing parameters
SI 32768
SF 125.7752164 MHz
WDW EM
SSB 0
LB 1.00 Hz
GB 0
PC 1.40

^{13}C-NMR

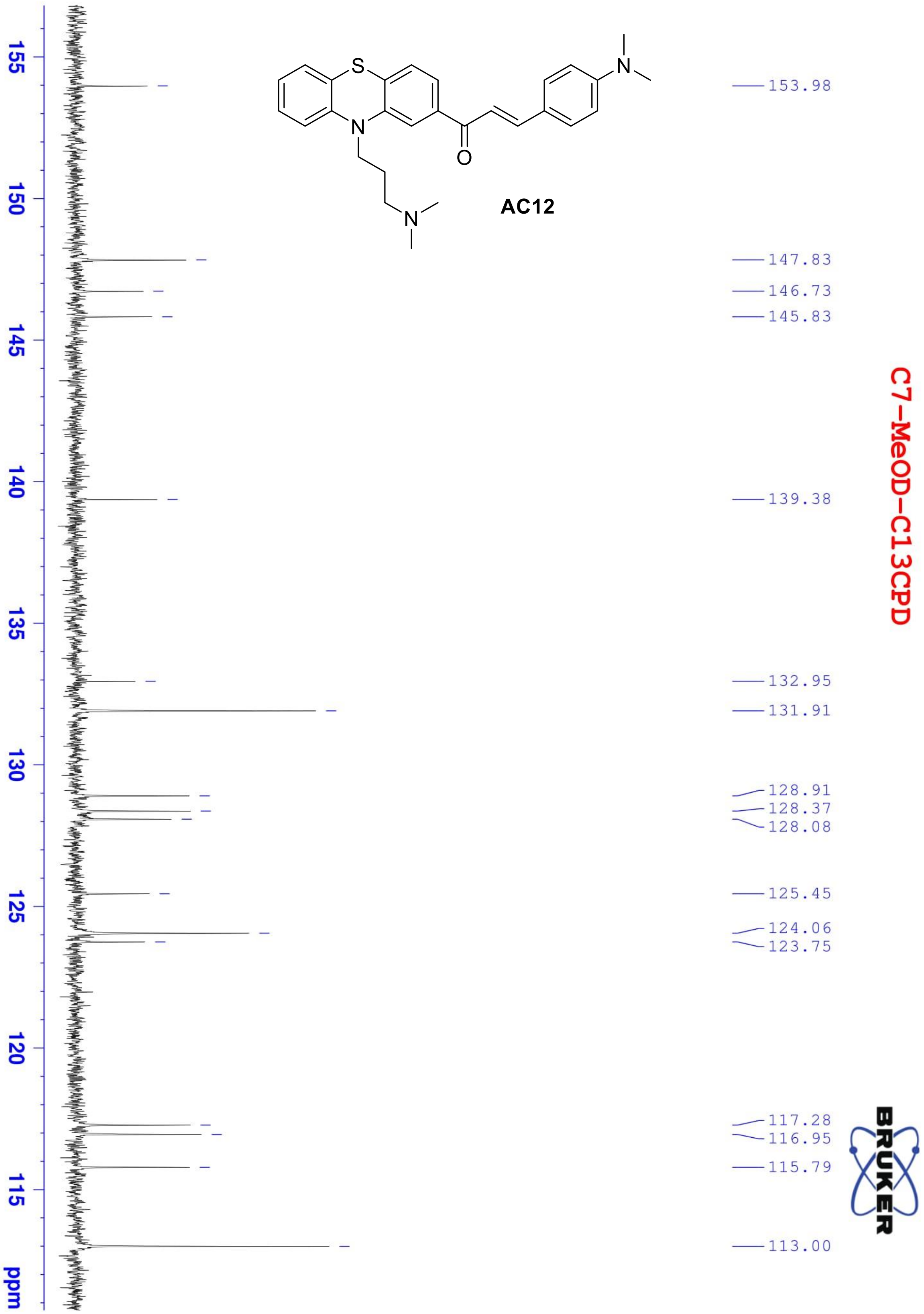

153.98
147.83
146.73
145.83
139.38
132.95
131.91
128.91
128.37
128.08
125.45
124.06
123.75
117.28
116.95
115.79
113.00
155
150
145
140
135
130
125
120
115
ppm
C7–MeOD–C13CPD
BRUKER

AC12

VIEN CONG NGHE HOA HOC
01 Mac Dinh Chi - Q.1 - Tp HCM

IR

Tel: 08.38296127
Fax: 08.38293889

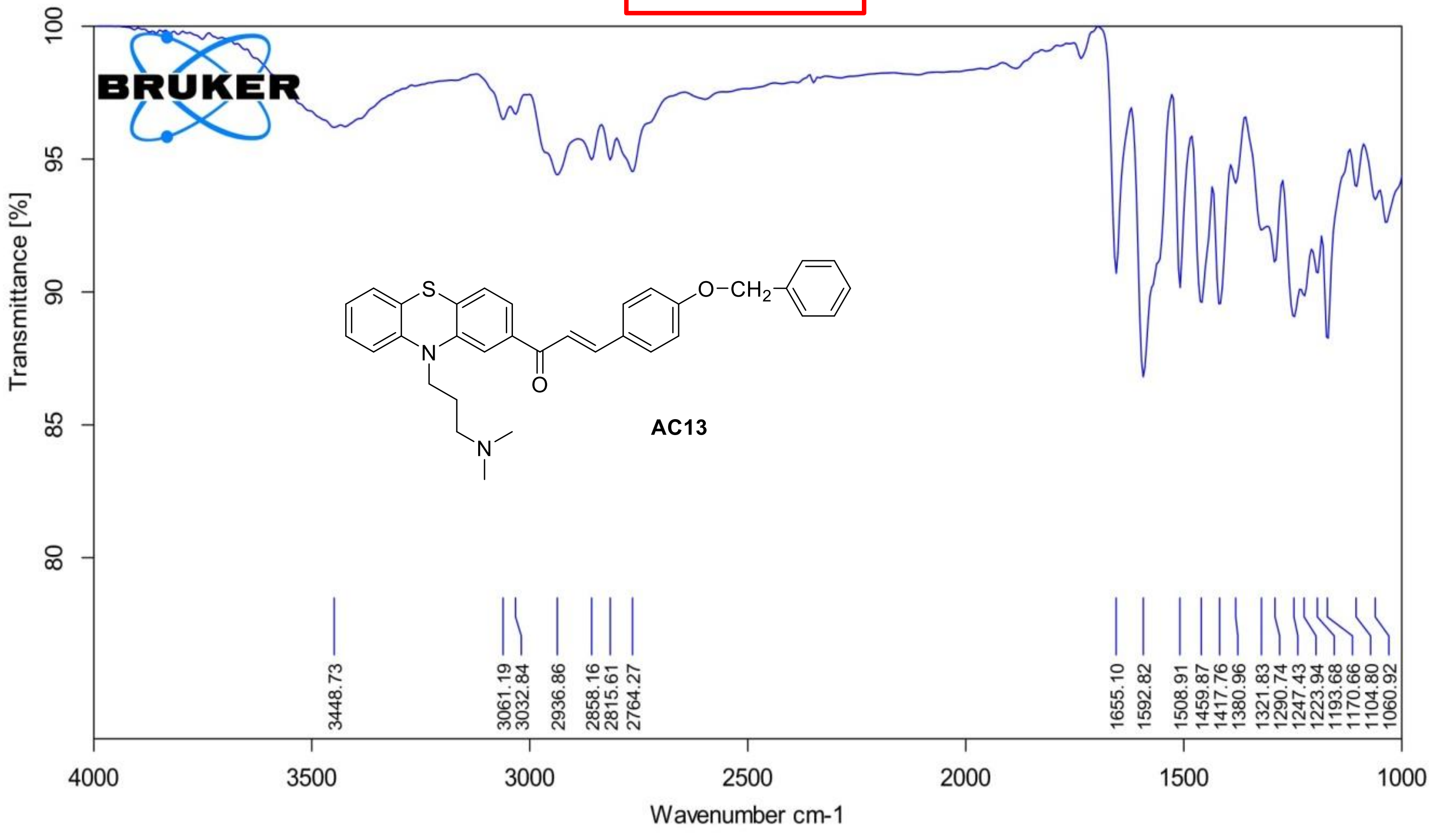

E:\OPUS 7\2017\THANG 6\20170606\C5.0	C5	TENSOR 27 - BRUKER - GERMANY	6/6/2017

MS

Qualitative Analysis Report

**Data Filename**	13-AC13.d	**Sample Name**	13-AC13
**Sample Type**	Sample	**Position**	P2-C2
**Instrument Name**	Instrument 1	**User Name**	
**Acq Method**	Cot ngan - MSMS_Pos.m	**Acquired Time**	22/08/2020 12:00:43 PM
**IRM Calibration Status**	Success	**DA Method**	COTNGAN.M.m
**Comment**			

**Sample Group**		**Info.**	
**Stream Name**	LC 1	**Acquisition SW Version**	6200 series TOF/6500 series Q-TOF B.06.01 (B6172 SP1)

+ESI TIC Scan Frag=140.0V BTBZ 9.d Smooth

User Chromatograms

Fragmentor Voltage 140 **Collision Energy** 0 **Ionization Mode** ESI

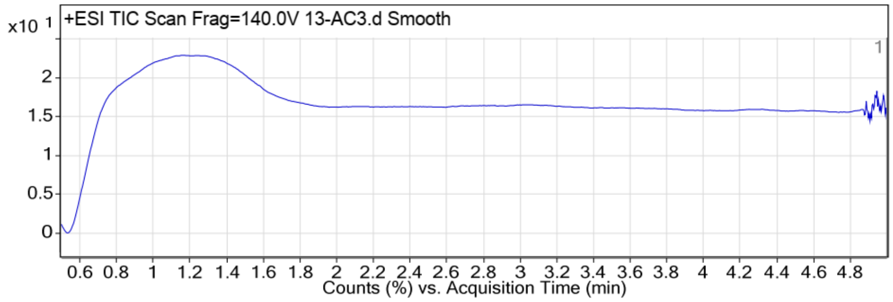

User Spectra

Fragmentor Voltage	Collision Energy	Ionization Mode
140	0	ESI

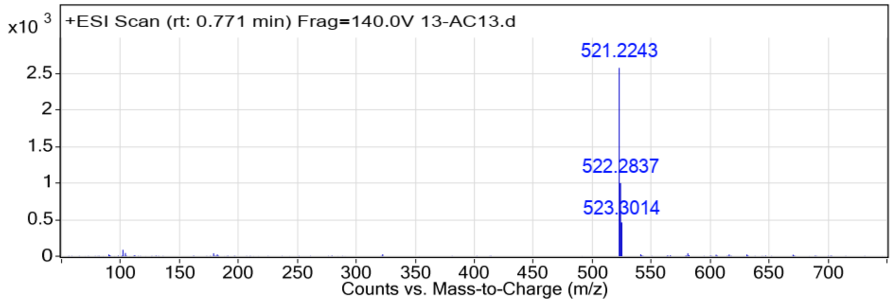

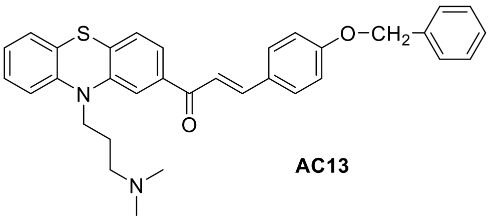

AC13

--- End Of Report ---

MS

**Sample Name**	13-AC13	**Position**	P2-C2	**Instrument Name**	Instrument 1	**User Name**	
**Inj Vol**	2	**InjPosition**		**SampleType**	Sample	**IRM Calibration Status**	Success
**Data Filename**	13-AC13.d	**ACQ Method**	Cot ngan - MSMS_Pos.	**Comment**		**Acquired Time**	22/08/2020 12:00:43 PM

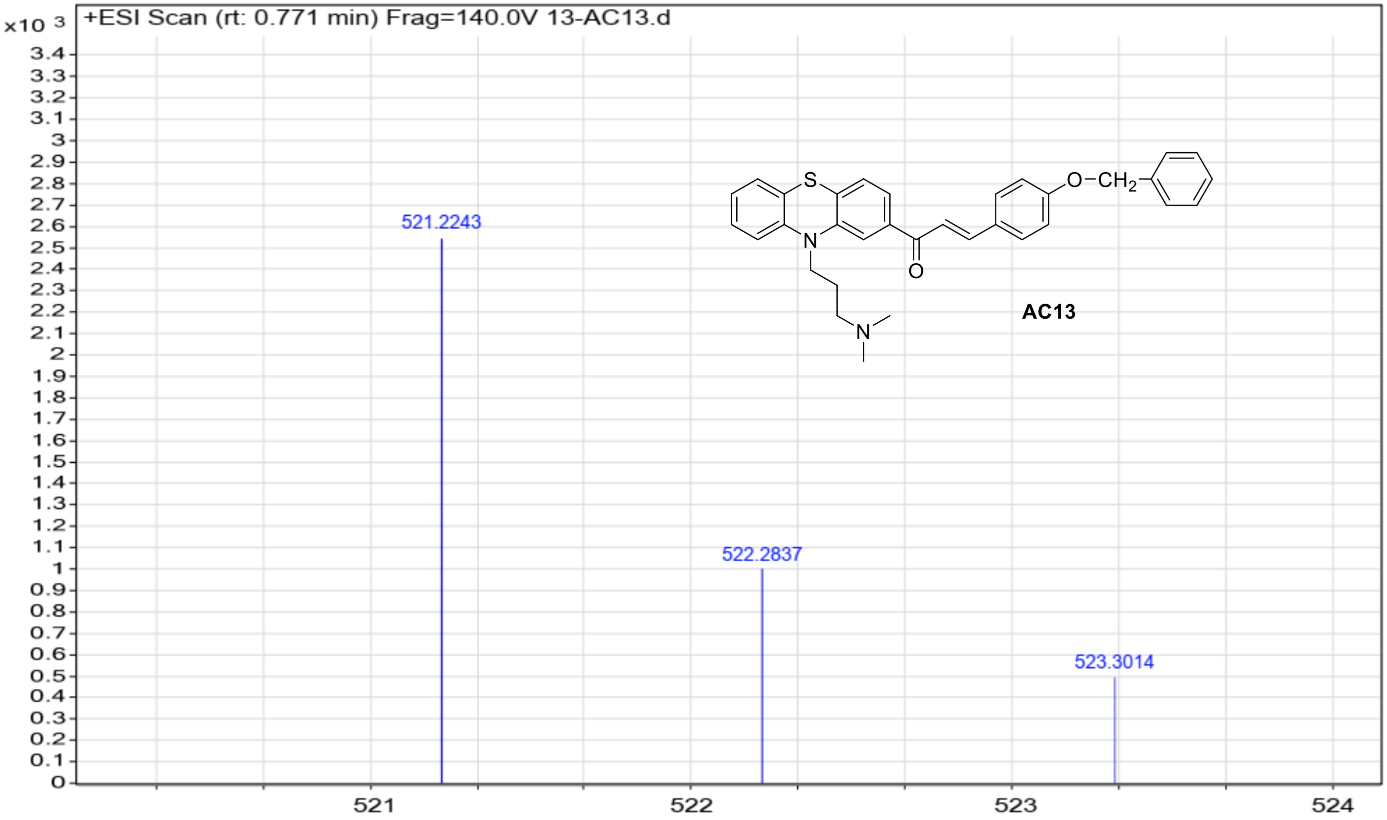

^{1}H-NMR

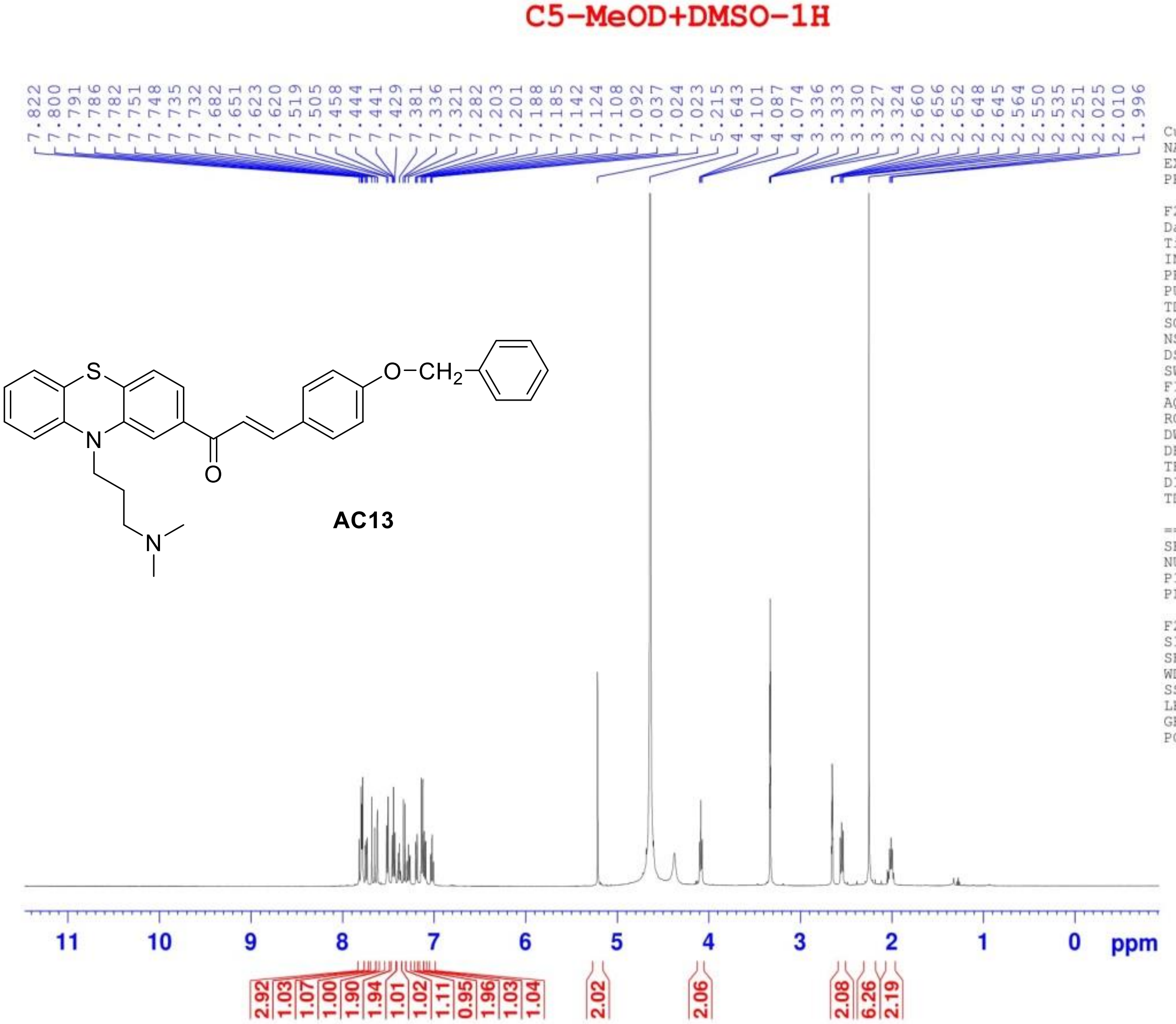

AC13

```
Current Data Parameters
NAME              113D_C5
EXPNO                   1
PROCNO                  1

F2 - Acquisition Parameters
Date_            20170607
Time                10.47
INSTRUM             spect
PROBHD   5 mm PABBO BB/
PULPROG              zg30
TD                  65536
SOLVENT              MeOD
NS                     16
DS                      2
SWH             10000.000 Hz
FIDRES           0.152588 Hz
AQ              3.2767999 sec
RG                 111.09
DW                 50.000 usec
DE                   6.50 usec
TE                  303.0 K
D1             1.00000000 sec
TD0                     1

======== CHANNEL f1 ========
SFO1          500.2030889 MHz
NUC1                   1H
P1                  10.00 usec
PLW1          22.00000000 W

F2 - Processing parameters
SI                  65536
SF            500.2000003 MHz
WDW                    EM
SSB      0
LB                   0.30 Hz
GB       0
PC                   1.00
```

^{1}H-NMR

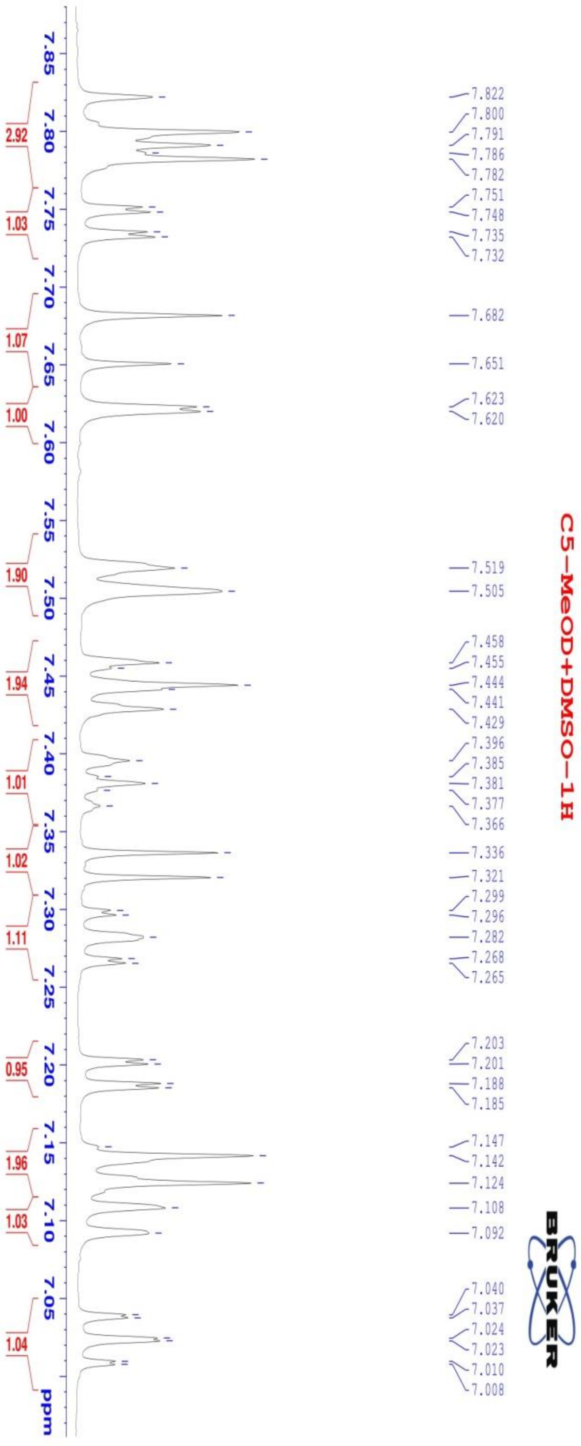

C5–MeOD+DMSO–1H
7.822
7.800
7.791
7.786
7.782
7.751
7.748
7.735
7.732
7.682
7.651
7.623
7.620
7.519
7.505
7.458
7.455
7.444
7.441
7.429
7.396
7.385
7.381
7.377
7.366
7.336
7.321
7.299
7.296
7.282
7.268
7.265
7.203
7.201
7.188
7.185
7.147
7.142
7.124
7.108
7.092
7.040
7.037
7.024
7.023
7.010
7.008
2.92
1.03
1.07
1.00
1.90
1.94
1.01
1.02
1.11
0.95
1.96
1.03
1.04
ppm

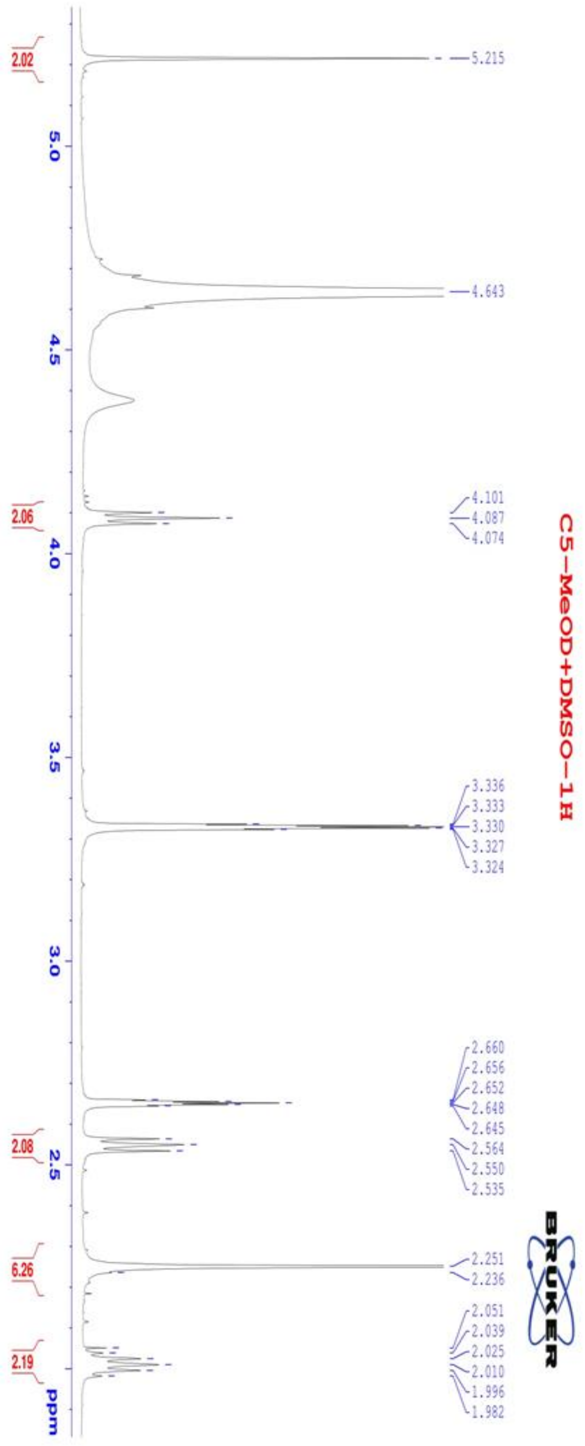

C5–MeOD+DMSO–1H
5.215
4.643
4.101
4.087
4.074
3.336
3.333
3.330
3.327
3.324
2.660
2.656
2.652
2.648
2.645
2.564
2.550
2.535
2.251
2.236
2.051
2.039
2.025
2.010
1.996
1.982
2.02
2.06
2.08
6.26
2.19
ppm

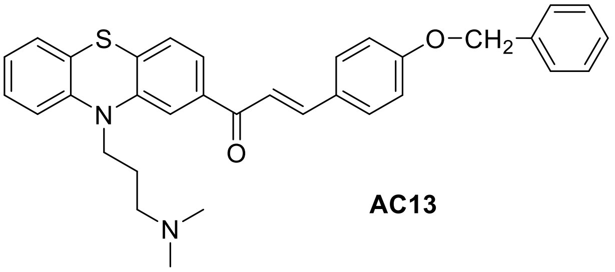

AC13

^{13}C-NMR

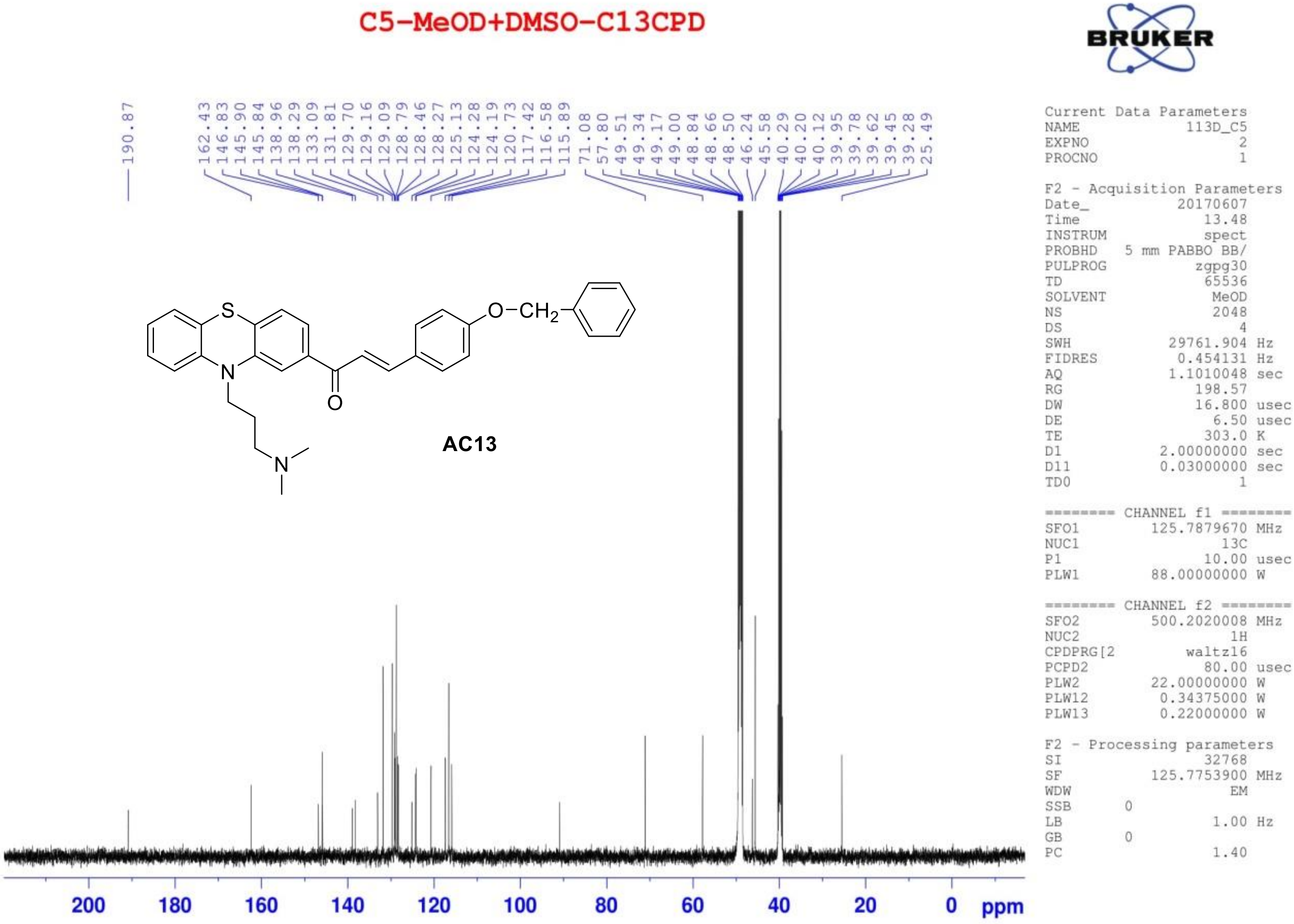
C5-MeOD+DMSO-C13CPD
BRUKER
190.87
162.43
146.83
145.90
145.84
138.96
138.29
133.09
131.81
129.70
129.16
129.09
128.79
128.46
128.27
125.13
124.28
124.19
120.73
117.42
116.58
115.89
71.08
57.80
49.51
49.34
49.17
49.00
48.84
48.66
48.50
46.24
45.58
40.29
40.20
40.12
39.95
39.78
39.62
39.45
39.28
25.49
S
N
O
O–CH2
N
AC13
Current Data Parameters
NAME 113D_C5
EXPNO 2
PROCNO 1
F2 - Acquisition Parameters
Date_ 20170607
Time 13.48
INSTRUM spect
PROBHD 5 mm PABBO BB/
PULPROG zgpg30
TD 65536
SOLVENT MeOD
NS 2048
DS 4
SWH 29761.904 Hz
FIDRES 0.454131 Hz
AQ 1.1010048 sec
RG 198.57
DW 16.800 usec
DE 6.50 usec
TE 303.0 K
D1 2.00000000 sec
D11 0.03000000 sec
TD0 1
======== CHANNEL f1 ========
SFO1 125.7879670 MHz
NUC1 13C
P1 10.00 usec
PLW1 88.00000000 W
======== CHANNEL f2 ========
SFO2 500.2020008 MHz
NUC2 1H
CPDPRG[2 waltz16
PCPD2 80.00 usec
PLW2 22.00000000 W
PLW12 0.34375000 W
PLW13 0.22000000 W
F2 - Processing parameters
SI 32768
SF 125.7753900 MHz
WDW EM
SSB 0
LB 1.00 Hz
GB 0
PC 1.40
200
180
160
140
120
100
80
60
40
20
0
ppm

^{13}C-NMR

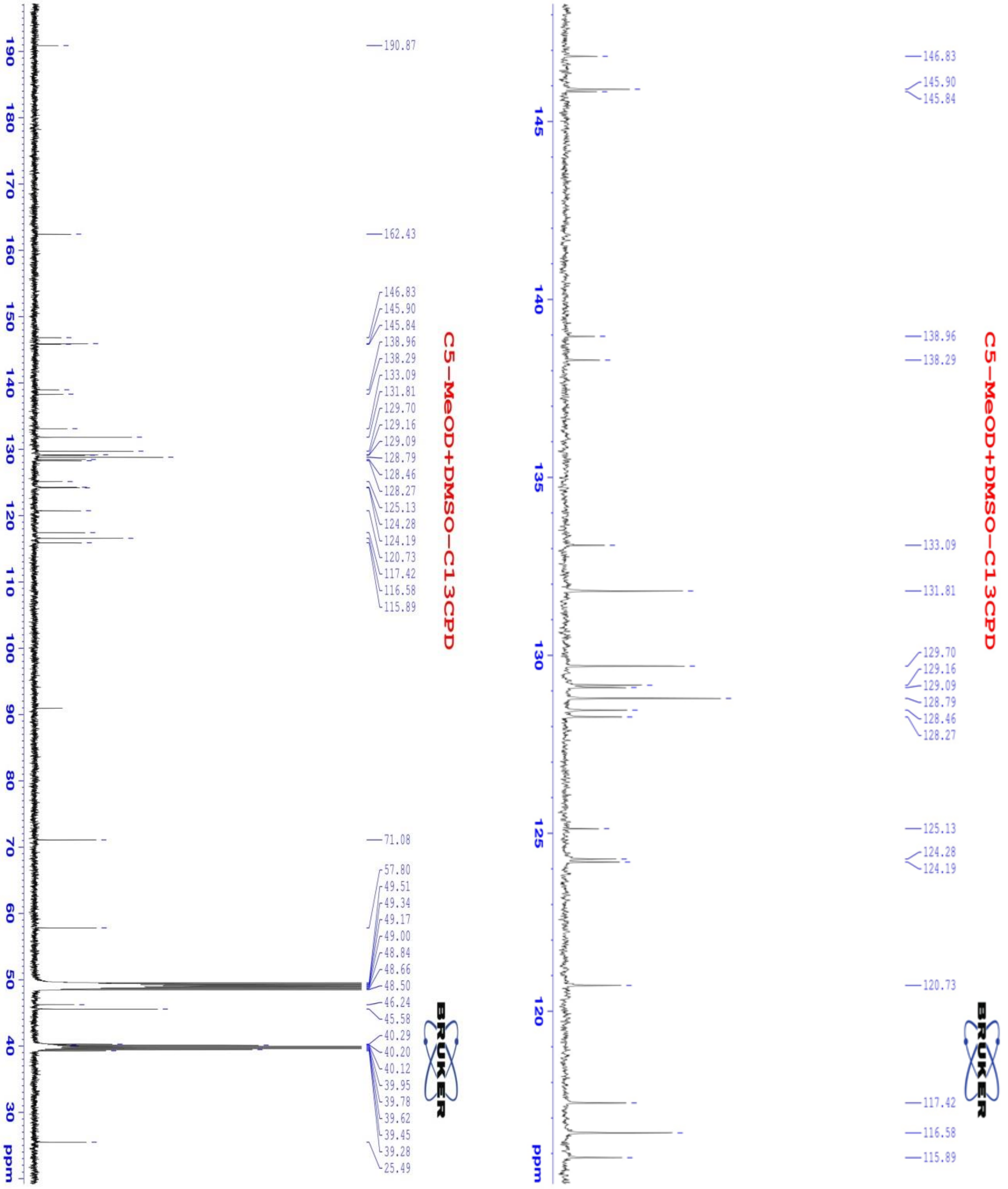
C5-MeOD+DMSO-C13CPD
190.87
162.43
146.83
145.90
145.84
138.96
138.29
133.09
131.81
129.70
129.16
129.09
128.79
128.46
128.27
125.13
124.28
124.19
120.73
117.42
116.58
115.89
71.08
57.80
49.51
49.34
49.17
49.00
48.84
48.66
48.50
46.24
45.58
40.29
40.20
40.12
39.95
39.78
39.62
39.45
39.28
25.49
ppm
C5-MeOD+DMSO-C13CPD
146.83
145.90
145.84
138.96
138.29
133.09
131.81
129.70
129.16
129.09
128.79
128.46
128.27
125.13
124.28
124.19
120.73
117.42
116.58
115.89
ppm
BRUKER

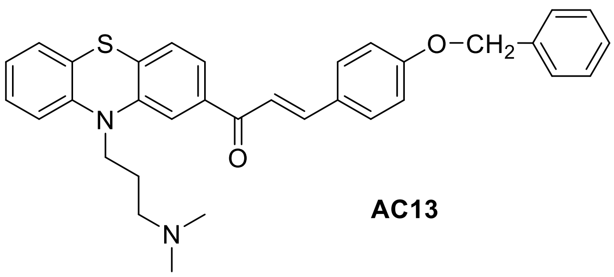
S
N
O
O–CH2
N

AC13

^{13}C-NMR

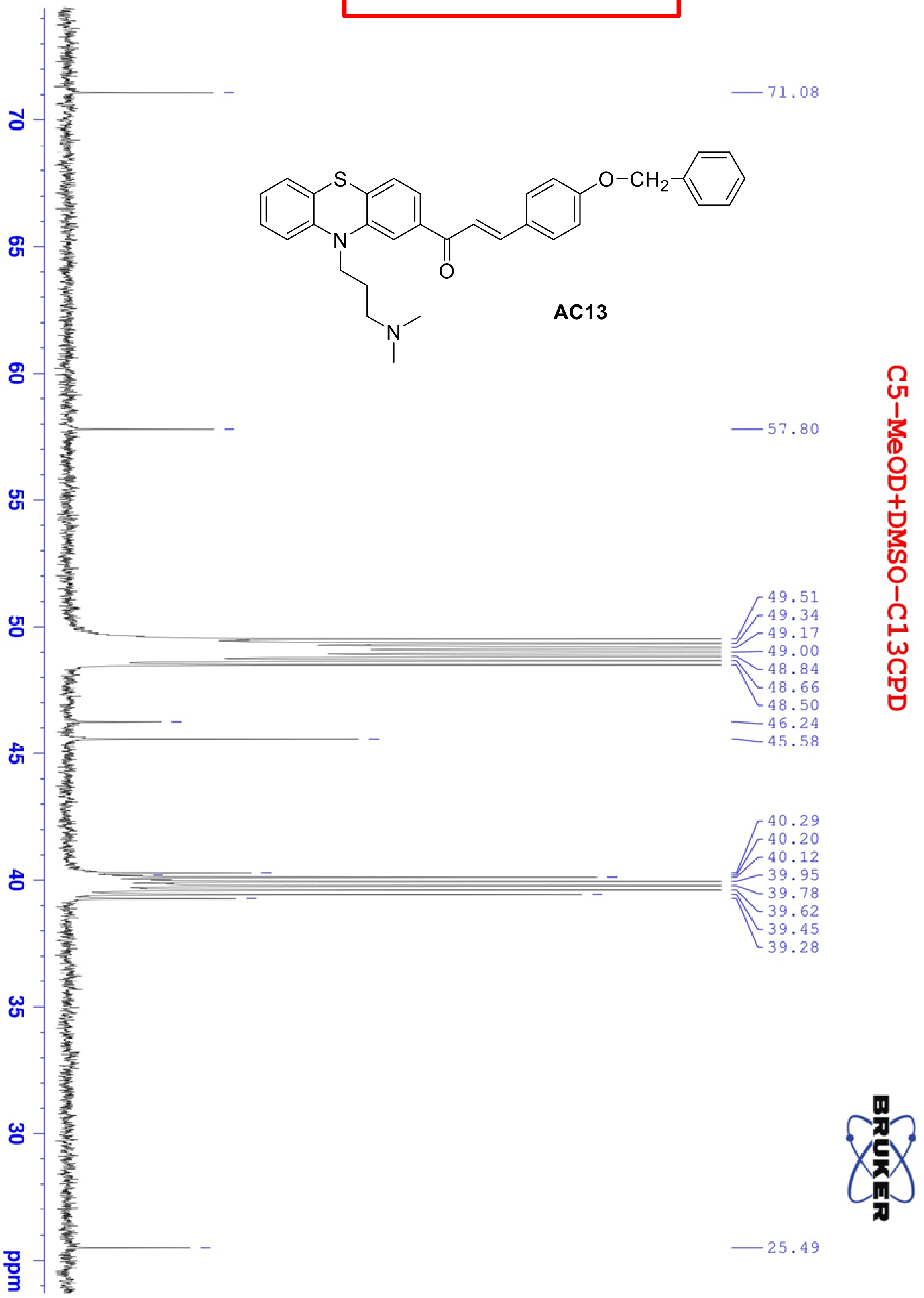

AC13
S
N
O
O−CH$_2$
N
71.08
57.80
49.51
49.34
49.17
49.00
48.84
48.66
48.50
46.24
45.58
40.29
40.20
40.12
39.95
39.78
39.62
39.45
39.28
25.49
70
65
60
55
50
45
40
35
30
ppm
C5−MeOD+DMSO−C13CPD
BRUKER

Reference

1. Thai, K.-M.; Bui, Q.-H.; Tran, T.-D.; Huynh, T.-N.-P. QSAR modeling on benzo[c]phenanthridine analogues as topoisomerase I inhibitors and anti-cancer agents. *Molecules* **2012**, *17*, 5690-5712, doi:10.3390/molecules17055690.
2. Chirico, N.; Gramatica, P. Real external predictivity of QSAR models: how to evaluate it? Comparison of different validation criteria and proposal of using the concordance correlation coefficient. *J Chem Inf Model* **2011**, *51*, 2320-2335, doi:10.1021/ci200211n.
3. Wildman, S.A.; Crippen, G.M. Prediction of Physicochemical Parameters by Atomic Contributions. *J Chem Inf Comput Sci* **1999**, *39*, 868-873, doi:10.1021/ci990307l.
4. Oprea, T.I. Property distribution of drug-related chemical databases. *Journal of computer-aided molecular design* **2000**, *14*, 251-264, doi:10.1023/a:1008130001697.
